# Tackling the Reactivity of Propadiene: Palladium Metallaphotoredox Dual Catalyzed Multi‐Component Allylation and Dienylation of Styrenes

**DOI:** 10.1002/anie.202518994

**Published:** 2025-11-21

**Authors:** Yi‐Fei Yang, Felix Bauer, Bernhard Breit

**Affiliations:** ^1^ Institut für Organische Chemie Albert‐Ludwigs‐Universität Freiburg Albertstraße 21 79104 Freiburg, im Breisgau Germany

**Keywords:** Allylation, Dienylation, Metallaphotoredox catalysis, Propadiene, Telomerization

## Abstract

Catalytic transformations of feedstock chemicals into value‐added products are a long‐standing challenge. A side product of the steam‐cracking process – propadiene – which is formed in 2.5 x 10^5^ t/y ‐ regardless of its potentially rich chemistry ‐ is usually wasted by burning it. To make more economical and sustainable use of valuable carbon atoms from feeds stocks, herein we report a palladium metallaphotoredox dual catalyzed multi‐component allylation and dienylation of styrene derivatives with propadiene as either an allylation or a dienylation reagent. The chemoselectivity for both directions are higher than 20:1 and pronounced functional group tolerance has been elucidated in both cases. Gram scale synthesis and orthogonal reactivities towards thermo allylation demonstrate the practicability of our protocols. Moreover, DFT calculation suggested an outer‐sphere reductive elimination at a Pd^I^ complex to be the most energetically favored route for both directions.

## Graphical abstract

We presented herein that propadiene can chemodivergently form either an allylic moiety or a dienylic moiety under Pd catalysis. Embedding this reactivity pattern into photocatalysis with DHP as the initial radical source and styrene derivatives as the initial acceptor leads to multi‐component allylation and dienylation.

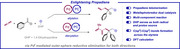

## Introduction

**Scheme 1:**
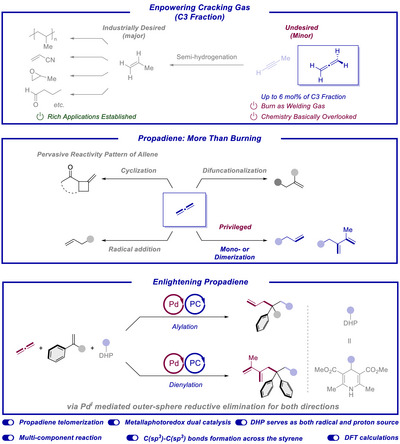
Introduction to propadiene and this work.

The steam cracking process is at the heart of the chemical industry transforming crude oil into valuable feedstock chemical building blocks for the production of the majority of chemical products ranging from functional materials to drug molecules among many others. In this regard an efficient use of all carbon atoms in this process for the formation of high‐value products is both of economical as well as sustainability interest. Among the most important fractions of the steam cracking process is the C3 fraction with propene being the mostly used major product (Scheme 1).^[^ ^1^ ^]^ However, a side product which constitutes 6 mol% of the C3‐fraction is propa‐1,2‐diene (also referred to as allene) together with its isomer propyne.^[^ ^2^, ^3^ ^]^ Today, the normal use of this fraction is mostly to burn it as the so‐called welding gas for thermal cutting and welding. However, this is unfortunate given the potentially rich chemistry of these highly‐unsaturated C3‐building blocks.

Several pioneering works on reactions of propadiene have been reported, such as [2 + 2] cyclization,^[^ ^4^, ^5^, ^6^ ^]^ difunctionalization,^[^ ^7^, ^8^, ^9^, ^10^, ^11^, ^12^, ^13^, ^14^ ^]^ radical addition,^[^ ^15^, ^16^, ^17^ ^]^ among others.^[^ ^18^, ^19^, ^20^, ^21^ ^]^ In addition to these common reactivities which can be found around other type of allenes, propadiene features a rather privileged reactivity fashion, known as telomerization, which includes monomerization as well as dimerization and higher order of oligomerization before interacting with pronucleophiles, first reported by Baker in 1973.^[^ ^22^ ^]^ However, the selectivity observed in these studies was typically low, and no significant improvements were reported since then.^[^ ^23^, ^24^, ^25^, ^26^ ^]^

Our group has a long‐standing research interest in applying allenes as building blocks in synthetic chemistry.^[^ ^27^, ^28^ ^]^ During the last decade a number of methods have been developed regarding the hydrofunctionalization of allenes under both thermal^[^ ^29^, ^30^, ^31^ ^]^ and photoredox conditions.^[^ ^32^, ^33^, ^34^, ^35^ ^]^ Following our continuous research focus and inspired by several seminal reports from other research groups,^[^ ^36^, ^37^, ^38^, ^39^ ^]^ our group recently realized a selective allylation reaction of a set of pronucleophiles with propadiene.^[^ ^40^ ^]^

The last several years have witnessed a flourish of metallaphotoredox dual catalysis, which integrates radical chemistry with transition metal catalysis and defines a new era of catalysis.^[^ ^41^, ^42^, ^43^, ^44^, ^45^ ^]^ In this context, we rationalized that it would be a great step forward if one could embed the telomerization of propadiene

Within the framework of metallaphotoredox catalysis in order to generate value‐added products from propadiene upon carbon‐carbon bond formation. We speculated that such a process could be initiated by a photoredox generated radical species, which could undergo an addition to a diphenylethylene generating a new more stable benzylic radical intermediate. At approximately the same time scale a palladium catalyst would be in charge of activating propadiene via telomerization. Finally, allylic and dienylic products could be delivered through an outer‐sphere reductive elimination step. We herein report the realization of such a unique three‐ or four‐component coupling reaction, respectively, in which two C(sp^3^)─C(sp^3^) bonds are formed across the alkene π‐bond and another C(sp^2^)─C(sp^2^) in the course of the dienylation direction. To the best of our knowledge, it represents the first example for the integration of propadiene telomerization with metallaphotoredox catalysis.

As the initial radical precursor, a DHP derivative^[^ ^46^, ^47^, ^48^, ^49^ ^]^ was selected not only because of its relatively low oxidation potential (*E*_ox_ = −1.05 V versus SCE)^[^ ^46^ ^]^ among other qualified candidates, but also attributed to its ability to act as a proton source, which is needed for the telomerization process (vide infra). Given the fact that interception of radical species with palladium was typically sluggish, a rather stable radical intermediate was chosen initially. Therefore, diphenylethylene was chosen as the initial radical acceptor since upon radical addition, the resulting diphenyl methyl radical would be one of the most stable radicals that can be accessed. Besides, considering that the commercial availability of propadiene may vary from region to region, a propadiene stock solution, which can be readily prepared in laboratory scale from 2,3‐dichloropropene,^[^ ^38^ ^]^ was used instead of a propadiene gas cylinder, which rendered our experimental setting more adaptable.

## Results and Discussion

**Scheme 2:**
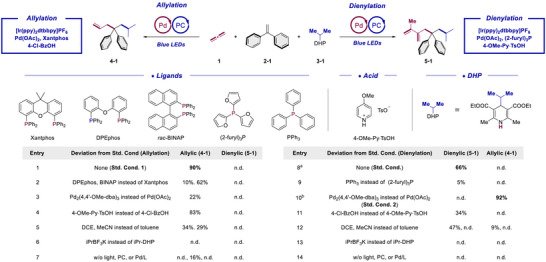
Optimization study. Std. Cond. (Allylation): Propa‐1,2‐diene (**1**) (0.1 mmol, 1.0 equiv.), 1,1‐diphenylethylene (**2–1**) (1.8 equiv.), iPr‐DHP (**3–1**) (1.5 equiv.), [Ir(ppy)_2_dtbbpy]PF_6_ (0.5 mol%), Pd(OAc)_2_ (7.5 mol%), Xantphos (9 mol%), and 4‐Cl‐BzOH (5 mol%) in toluene (1 mL) were irradiated with 16 W blue LEDs for 16 h under argon. Std. Cond. (Dienylation): Propa‐1,2‐diene (**1**) (0.2 mmol, 2*1.0 equiv.), 1,1‐diphenylethylene (**2–1**) (3.0 equiv.), iPr‐DHP (**3–1**) (1.5 equiv.), [Ir(ppy)_2_dtbbpy]PF_6_ (0.5 mol%), Pd(OAc)_2_ (7.5 mol%), (2‐furyl)_3_P (18 mol%), and 4‐OMe‐Py·TsOH (10 mol%) in toluene (1 mL) were irradiated with 16 W blue LEDs for 16 h under argon. a: w/ 1.2 equiv. DHP, 1.0 mol% PC and 10 mol% Pd/L instead. b: w/ propa‐1,2‐diene (**1**) (0.2 mmol, 1.0 equiv.), 1,1‐diphenylethylene (**2–1**) (1.8 equiv.), and PPTS (10 mol%) instead (Alternative Std. Cond. for the allylation direction, Std. Cond. 2). Yields were determined by ^1^H NMR with 2‐picoline as an internal standard. PC = Photocatalyst. DHP = Dihydropyridine. PPTS = Pyridinium *p*‐toluenesulfonate. Py = Pyridine.

With this rational design in mind, we launched our study from the allylation direction. Gratifyingly, a thorough survey of the reaction parameters led us to the standard condition (Scheme 2, entry 1, **Std. Cond. 1**) which can deliver the allylic product in 90% yield. Similar endeavors were devoted to the dienylation direction and we were delighted to identify the standard condition (entry 8, **Std. Cond**.) which led to the generation of the dienylic product in 66% yield. Remarkably, for both directions the chemoselectivity over the other isomer was exceedingly high under the optimal settings. Several rapid deviation experiments were conducted to ensure the superiority of the standard conditions. Thus, change of phosphine ligands to either DPEphos or BINAP proved to be inferior for the allylation (10% and 62%, respectively, entry 2) compared with Xantphos. As for dienylation, switching from (2‐furyl)_3_P to PPh_3_ also led to a detrimental result (5%, entry 9). Also, the palladium precursor had an influence. Hence, for the allylation direction, replacing Pd(OAc)_2_ with Pd_2_(4,4′‐OMe‐dba)_3_ deteriorated the desired transformation (22%, entry 3). However, it is interesting to note that for the dienylation direction, the same replacement led to a complete switch of the chemoselectivity, favoring the allylic product over the dienylic product with an impressive 92% yield. The dba ligand was assumed to act as a non‐innocent ligand in this case, and this condition was designated as an alternative choice for the allylation direction (entry 10, **Std. Cond. 2**). Empirically, we assume that a more congested coordination environment of Pd provided by either a rigid bidentate Xantphos ligand with a large bite angle or an additional non‐innocent dba ligand would favor the allylic direction. Conversely, a more flexible, less congested ligation environment would instead be beneficial for the approach and insertion of the secondary propadiene and favor the dienylation direction. The choice of Bronsted acid cocatalyst had also an influence on reaction yield (see entries 4 and 11). The same holds for changing the solvent from toluene to either DCE or MeCN (entries 5 and 12). Moreover, iPrBF_3_K proved to be an unsuitable radical precursor under our conditions (entries 6 and 13). Notably, the reaction can also be performed in 1 atm of propadiene. Upon a slight modification of the substrate loading the desired allylic and dienylic products can be delivered in a comparable yield with high chemoselectivity (87% yield, 17.4:1 selectivity based on Std. Cond. 2 for allylation and 61% yield, >20:1 selectivity based on Std. Cond. for dienylation, please refer to the Supporting Information for more details), leveraging our methods adaptable for the industrial usage where a propadiene gas cylinder would be more easily accessible. Finally, control experiments confirmed that continuous light irradiation, photocatalyst and Pd/L were all indispensable for efficient catalytic turnover (entries 7 and 14).

**Scheme 3:**
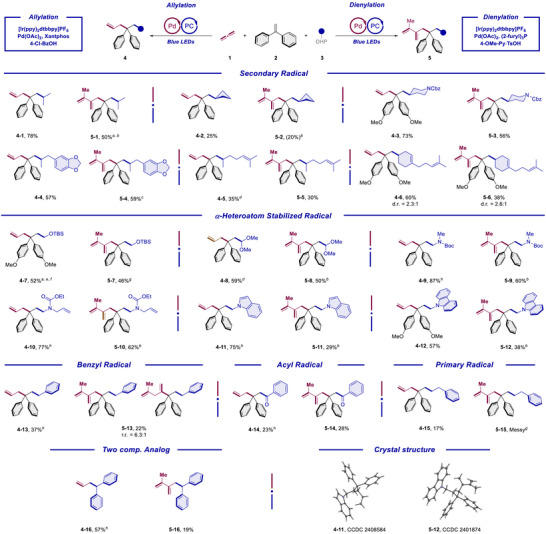
Substrate scope‐DHP. Reaction condition for allylation‐Std. Cond. 1: Propa‐1,2‐diene (**1**) (0.2 mmol, 1.0 equiv.), 1,1‐diphenylethylene (**2**) (1.8 equiv.), DHP derivatives (**3**) (1.5 equiv.), [Ir(ppy)_2_dtbbpy]PF_6_ (0.5 mol%), Pd(OAc)_2_ (7.5 mol%), Xantphos (9 mol%), and 4‐Cl‐BzOH (5 mol%) in toluene (2 mL) were irradiated with 16 W blue LEDs for 16 h under argon. Reaction condition for allylation‐Std. Cond. 2: Propa‐1,2‐diene (**1**) (0.2 mmol, 1.0 equiv.), 1,1‐diphenylethylene (**2**) (1.8 equiv.), DHP derivatives (**3**) (1.5 equiv.), [Ir(ppy)_2_dtbbpy]PF_6_ (0.5 mol%), Pd_2_(4,4′‐MeO‐dba)_3_ (3.75 mol%), (2‐furyl)_3_P (18 mol%), and PPTS (10 mol%) in toluene (2 mL) were irradiated with 16 W blue LEDs for 16 h under argon. Unless otherwise noted, the reaction for allylation was performed following the Std. Cond. 1. Reaction condition for dienylation: Propa‐1,2‐diene (**1**) (0.4 mmol, 2*1.0 equiv.), 1,1‐diphenylethylene (**2**) (3.0 equiv.), DHP derivatives (**3**) (1.5 equiv.), [Ir(ppy)_2_dtbbpy]PF_6_ (0.5 mol%), Pd(OAc)_2_ (7.5 mol%), (2‐furyl)_3_P (18 mol%), and 4‐OMe‐Py·TsOH (10 mol%) in toluene (2 mL) were irradiated with 16 W blue LEDs for 16 h under argon. Unless otherwise noted, the chemoselectivity for both the allylation and dienylation direction was higher than 19:1. a: w/ 1.2 equiv. DHP, b: w/ 1.0 mol% [Ir(ppy)_2_dtbbpy]PF_6_, 10 mol% Pd(OAc)_2_ and 24 mol% (2‐furyl)_3_P. c: w/ 4.0 equiv. 1,1‐diphenylethylene (**2**). d: w/ (*η* ^3^‐cinnamyl)PdCp. e: w/ 1.0 mol% [Ir(ppy)_2_dtbbpy]PF_6_, 10 mol% Pd(OAc)_2,_ and 12.5 mol% Xantphos. f: w/ 1.5 equiv. 1,1‐diphenylethylene (**2**). g: w/ 100 mg 3 Å MS. h: w/ Std. Cond. 2. PC = Photocatalyst. DHP = Dihydropyridine. PPTS = Pyridinium *p*‐toluenesulfonate. Py = Pyridine.

With the optimal conditions in hand, we set out to investigate the substrate scope. For a more integrated presentation, the results for allylation and dienylation are given in a comparative way, respectively. Namely the catalytic conditions for both directions were applied to typically the same array of starting materials and the corresponding allylic and dienylic products are listed together. The scope of DHP radical precursor component was explored first (Scheme 3).

Thus, several structurally diverse secondary alkyl radicals could be transferred smoothly in moderate to high yields for most cases (**3–1** to **3**–**6**). It is worth mention that two of them, which feature additional one and two internal olefin functions at the DHP partner, respectively (**3**–**5** and **3**–**6**), can be tolerated under the radical conditions to deliver the corresponding multi‐component products. The generation of a cyclobutyl radical was proved to be problematic, leading to lower yields for both direction (**3–2**). *α*‐Heteroatom substituted radical precursors are also compatible. Thus, TBS protected *α*‐hydroxy radical (**3**–**7**), acetal radical (**3**–**8**), Boc protected *α*‐amino radical (**3**–**9**), *α*‐amido radical tethered with an allylic motif (**3**–**10**), *N*‐methylene substituted indole and carbazole radical (**3**–**11** and **3**–**12**) were all proved to be amenable coupling partners in our protocols. Moreover, a benzyl radical can also be adopted, leading to **4**–**13** and **5–13**, respectfully. It is of further note that in the case of benzyl radical for the dienylation direction, a nonnegligible regioisomer was also observed. We propose that the secondary migratory insertion of the initially formed methylvinyl Pd^II^ intermediate to the propadiene with Pd attached to the central carbon forms an isomeric diene unit, which eventually leads to the minor regioisomer. To further push the scope, benzoyl substituted DHP derivatives (**3**–**14**) were subjected to both protocols. The catalytic cycles got less efficient, but still, the targeted products can be furnished in synthetically useful yields. In addition, the liberation of a primary alkyl radical was shown to be sluggish, only allylic product was collected in low yield (**4**–**15**). Intriguingly, when the reaction was initiated from diphenyl methyl radical generated directly from the corresponding DHP derivative (**3**–**16**) in the absence of diphenylethylene, the reactions can also proceed in a similar two‐component fashion. Finally, single crystal structure analysis proved the structural assignment for both allylation and dienylation products unambiguously.^[^ ^50^ ^]^

**Scheme 4:**
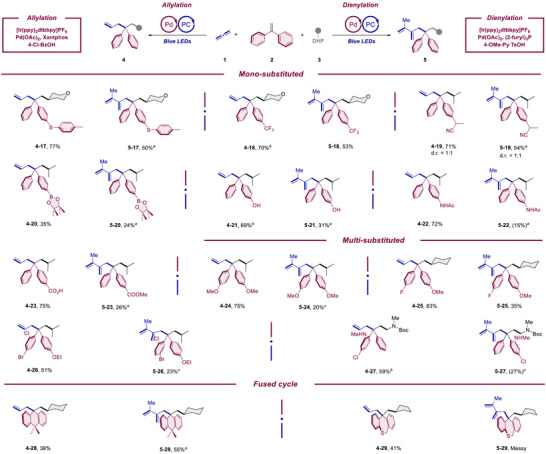
Substrate scope‐diphenylethylene. Reaction condition for allylation‐**Std. Cond. 1**: Propa‐1,2‐diene (**1**) (0.2 mmol, 1.0 equiv.), 1,1‐diphenylethylene derivatives (**2**) (1.8 equiv.), DHP derivatives (**3**) (1.5 equiv.), [Ir(ppy)_2_dtbbpy]PF_6_ (0.5 mol%), Pd(OAc)_2_ (7.5 mol%), Xantphos (9 mol%), and 4‐Cl‐BzOH (5 mol%) in toluene (2 mL) were irradiated with 16 W blue LEDs for 16 h under argon. Reaction condition for allylation‐Std. Cond. 2: Propa‐1,2‐diene (**1**) (0.2 mmol, 1.0 equiv.), 1,1‐diphenylethylene derivatives (**2**) (1.8 equiv.), DHP derivatives (**3**) (1.5 equiv.), [Ir(ppy)_2_dtbbpy]PF_6_ (0.5 mol%), Pd_2_(4,4′‐MeO‐dba)_3_ (3.75 mol%), (2‐furyl)_3_P (18 mol%), and PPTS (10 mol%) in toluene (2 mL) were irradiated with 16 W blue LEDs for 16 h under argon. Unless otherwise noted, the reaction for allylation was performed following the Std. Cond. 1. Reaction condition for dienylation: Propa‐1,2‐diene (**1**) (0.4 mmol, 2*1.0 equiv.), 1,1‐diphenylethylene derivatives (**2**) (3.0 equiv.), DHP derivatives (**3**) (1.5 equiv.), [Ir(ppy)_2_dtbbpy]PF_6_ (0.5 mol%), Pd(OAc)_2_ (7.5 mol%), (2‐furyl)_3_P (18 mol%), and 4‐OMe‐Py·TsOH (10 mol%) in toluene (2 mL) were irradiated with 16 W blue LEDs for 16 h under argon. Unless otherwise noted, the chemoselectivity for both the allylation and dienylation direction was higher than 19:1. a: w/ 100 mg 3 Å MS. b: w/ Std. Cond. 2, c: w/ 1.0 mol% [Ir(ppy)_2_dtbbpy]PF_6_, 10 mol% Pd(OAc)_2,_ and 24 mol% (2‐furyl)_3_P. PC = Photocatalyst. DHP = Dihydropyridine. PPTS = Pyridinium *p*‐toluenesulfonate. Py = Pyridine.

Next the structural variability of the diarylalkene reaction partner was explored (Scheme 4). Thus, a F (**2**–**25**), Cl (**2**–**26**), Br (**2**–**26**), thioether (**2**–**17**), CF_3_ (**2**–**18**), CN (**2**–**19**), Bpin (**2**–**20**), and OH (**2**–**21**) substituted diphenylethylenes proved all to be viable substrates for both directions. Particularly, in the allylation direction a better functional group tolerance was observed for a methyl aniline (**2**–**27**), an NHAc (**2**–**22**) as well as for a free carboxylic acid (**2**–**23**) function. The scope could be further extended to a carbocyclic (**2**–**28**) and heterocyclic system (**2**–**29**).

**Scheme 5:**
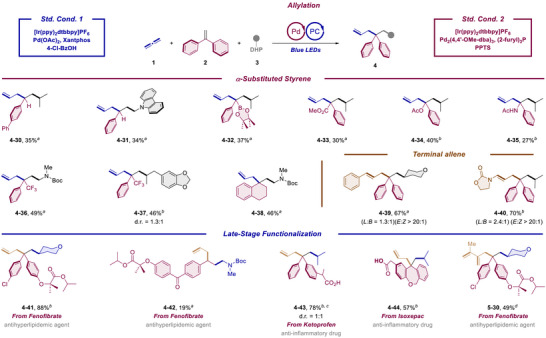
Substrate scope‐styrene, terminal allene and late‐stage functionalization. Reaction condition for allylation‐Std. Cond. 1: Propa‐1,2‐diene (**1**) (0.2 mmol, 1.0 equiv.), 1,1‐diphenylethylene derivatives (**2**) (1.8 equiv.), DHP derivatives (**3**) (1.5 equiv.), [Ir(ppy)_2_dtbbpy]PF_6_ (0.5 mol%), Pd(OAc)_2_ (7.5 mol%), Xantphos (9 mol%), and 4‐Cl‐BzOH (5 mol%) in toluene (2 mL) were irradiated with 16 W blue LEDs for 16 h under argon. Reaction condition for allylation‐Std. Cond. 2: Propa‐1,2‐diene (**1**) (0.2 mmol, 1.0 equiv.), 1,1‐diphenylethylene derivatives (**2**) (1.8 equiv.), DHP derivatives (**3**) (1.5 equiv.), [Ir(ppy)_2_dtbbpy]PF_6_ (0.5 mol%), Pd_2_(4,4′‐MeO‐dba)_3_ (3.75 mol%), (2‐furyl)_3_P (18 mol%), and PPTS (10 mol%) in toluene (2 mL) were irradiated with 16 W blue LEDs for 16 h under argon. Unless otherwise noted, the reaction for allylation was performed following the Std. Cond. 1. Reaction condition for dienylation: Propa‐1,2‐diene (**1**) (0.4 mmol, 2*1.0 equiv.), 1,1‐diphenylethylene derivatives (**2**) (3.0 equiv.), DHP derivatives (**3**) (1.2 equiv.), [Ir(ppy)_2_dtbbpy]PF_6_ (0.5 mol%), Pd(OAc)_2_ (7.5 mol%), (2‐furyl)_3_P (18  mol%), and 4‐OMe‐Py·TsOH (10 mol%) in toluene (2 mL) were irradiated with 16 W blue LEDs for 16 h under argon. Unless otherwise noted, the chemoselectivity for both the allylation and dienylation direction was higher than 19:1. a: The reaction was performed following the Std. Cond. 2. b: The reaction was performed following the Std. Cond. 1. c: w/ (*η* ^3^‐cinnamyl)PdCp. d: The reaction was performed following the Std. Cond. for dienylation. PC = Photocatalyst. DHP = Dihydropyridine. PPTS = Pyridinium *p*‐toluenesulfonate. Py = Pyridine.

Encouraged by these results, we envisioned that styrene and its *α*‐substituted analogs, may also be suitable for our protocols. While trials for dienylation typically afforded the products in less than 30% yields, we were delighted to find that the corresponding allylation manifold indeed afforded the desired products in acceptable yields (Scheme 5. Please refer to the Supporting Information for a full overview). In this context, styrene itself (**2**–**31**) and a number of functionalized styrenes with *α*‐Bpin (**2**–**32**), CO_2_Me (**2**–**33**), OAc (**2**–**34**), NHAc (**2**–**35**), and CF_3_ (**2–36**, **2**–**27**) functional groups reacted smoothly to furnish the three‐component allylation products. Besides, *α*‐Methylenetetralin (**2**–**38**), featuring a 1,1‐disubstitued styrene moiety, can also be adapted to our condition to construct a highly functionalized molecule. We also rationalized that it is not necessary to restrict the allylic coupling partner to propadiene. In fact, phenyl allene and alleneamide also exhibited excellent reactivity, albeit the regioselectivity was not high (**4**–**39** and **4**–**40**). Finally, late‐stage functionalization of pharmaceuticals was carried out. As depicted in Scheme 5, the vinyl group can be installed into two different sites of Fenofibrate through either Wittig olefination or Suzuki–Miyaura cross coupling, which in turn lead to the decoration at two distinct positions (**2**–**41** and **2**–**42**). The elaboration of Ketoprofen and Isoxepac tethered with a carboxylic group (**2**–**43** and **2**–**44**) was also achieved in high yield. Moreover, the introduction of a diene motif into Fenofibrate derivative was also realized (**5**–**30**).

**Scheme 6:**
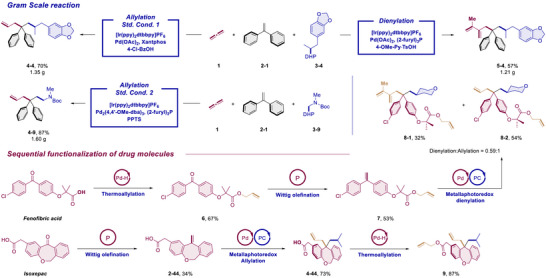
Gram scale reactions and sequential functionalization of drug molecules.

To demonstrate the practicality of our methods, gram scale reactions were conducted (Scheme 6). Gratifyingly, for all the established optimal conditions the products can be obtained on gram scale without loss of efficiency. Considering the fact that polar methods are typically orthogonal to the radical approaches, we conceived that it is rational to combine our previously established thermal palladium‐catalyzed allylation^[^ ^40^ ^]^ with this newly developed photo allylation to achieve a consecutive elaboration of complex molecules through divergent chemoselectivity. To elucidate this design, we started from the thermal allylation of Fenofibric acid to furnish an allylic ester (**6**). Upon wittig olefination the product was ready for the metallaphotoredox dual catalyzed dienylation. In this specific case the chemoselectivity between allylic and dienylic products was not high but both isomers (**8–1** and **8–2**) can be successfully isolated and separated. Another example for this orthogonal chemoselectivity started from Isoxepac. After methylenation, the product was subjected to the metallaphotoredox allylation condition to introduce an isopropyl and an allyl group across the double bond (**4**–**44**). A complementary thermal allylation with propadiene introduced another allylic group at the carboxyl terminus (**9**).

**Scheme 7:**
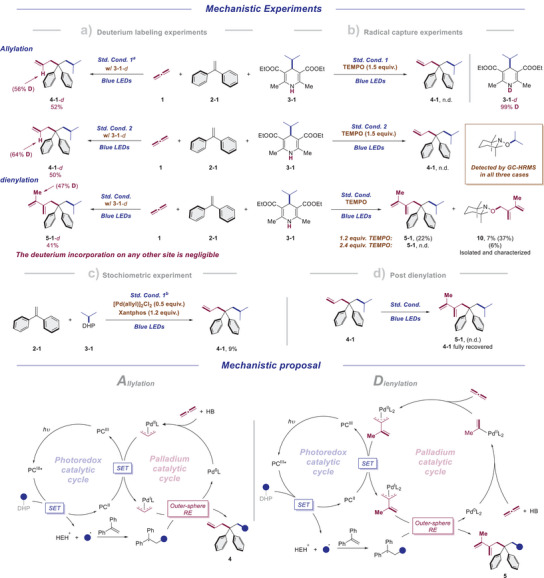
Mechanistic experiments and proposals. a) The reaction was performed for 72 h. b) w/ 4‐CF_3_‐BzOH instead, and c) w/ 1.5 equiv. of **2–1** instead. Please see the Supporting Information for experimental details. TEMPO = (2,2,6,6‐Tetramethylpiperidin‐1‐yl)oxyl.

Preliminary mechanistic experiments were conducted to shed light on the reaction mechanisms. We started with radical capture experiments using TEMPO as a radical scavenger (Scheme 7b). The catalytic cycles were ceased and the isopropyl‐TEMPO adduct was detected for every case, which suggested the involvement of radical intermediates during the reaction courses. Intriguingly, when 1.2 equiv. of TEMPO was subjected to the dienylation condition, diene‐TEMPO adduct was also observed and characterized, which may indicate a possible equivalence between a Pd^I^‐diene intermediate and Pd^0^ species along with a dienyl radical. Afterwards we conducted an array of deuterium labeling experiments (Scheme 7a). The deuterated *^i^* Pr‐DHP with deuterium labelling at N─H was used in the standard conditions for both allylation and dienylation directions. For both conditions optimized for allylation, the deuterium was selectively incorporated into the C2 position of allyl moiety with 56% and 64% D incorporation ratio, respectively. The deuterium incorporation for any other site was negligible. These results show that DHP derivatives not only serve as radical precursors, but also the major proton source. In the case of dienylation, the deuterium was specifically introduced into the 2‐methyl group of the diene motif. Besides, a stoichiometric experiment using 0.5 equiv. of [Pd(allyl)]_2_Cl_2_ in the absence of propadiene was conducted based on stand. Cond. 1 (Scheme 7c). Around 9% yield of allylic product was detected through crude NMR, which may support the involvement of π‐allyl Pd^II^ intermediate. Finally, we subjected the allylic product **4–1** into the dienylation condition to probe if the dienyl moiety was constructed first around the Pd complex or after the allylic product formation (Scheme 7d). No dienylic product was detected under such condition and the allylic compound was completely recovered, which suggested the post dienylation to be unlikely.

Based on the mechanistic experiments and literature reports,^[^ ^51^, ^52^, ^53^ ^]^ following mechanisms were proposed (Scheme 7). Regarding allylation, the photocatalyst was reductively quenched by DHP derivatives to release an active radical species along with a protonated pyridine derivative and reduced Ir^II^ catalyst. Given the unstable nature of the so formed radical, its direct engagement in palladium cycle was quite unlikely. Instead, the radical addition to the diphenylethylene would be an energetically more facile process, which lead to a rather stable benzyl radical intermediate. Meanwhile, the ligated Pd^0^ species would undergo an oxidative addition with acid additive to furnish a Pd^II−^‐H species, which can readily insert into propadiene to deliver a π–allyl Pd^II^ species. The reduction of which by the Ir^II^ catalyst would give a π–allyl Pd^I^ complex. It can be attacked by the previously described benzyl radical via an outer‐sphere mechanism to afford the allylic product and close the Pd catalytic cycle.

As for the dienylation direction, the photoredox catalyzed radical process would generally be the same. The difference exists in the palladium cycle. In this case, we assumed that the diene unit was formed through consecutive insertion of propadiene into a Pd–H species. Afterwards the π–allyl Pd^II^ species would be reduced by the photocatalyst and intercept the benzyl radical to furnish the dienylic product.

**Scheme 8:**
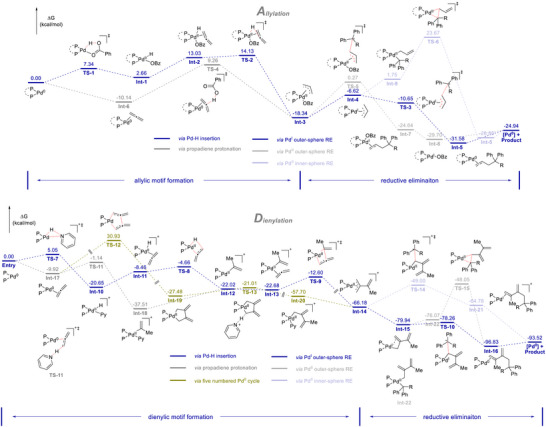
Calculated energy profiles for the allylation (top) and dienylation (bottom) (B3LYP/def2qzvp/SDD/D3BJ/SMD(toluene)//B3LYP/6–31G(d,p)/SDD/D3BJ).

To get deeper insight into the mechanistic details, we conducted DFT calculations for both directions and some representative results are illustrated here (Scheme 8. Please refer to the Supporting Information for a detailed discussion). We commenced our survey from the allylation condition under the Std. Cond. 1. As depicted in Scheme 8, regarding the formation of π–allyl palladium complex, the oxidative addition of benzoic acid to Pd (**TS‐1**) followed by propadiene hydrometallation (**TS‐2**) outcompeted the direct protonation of the coordinated propadiene (**TS‐4**). Upon the formation of π‐allyl palladium complex (**Int‐3**), three competing pathways were considered. The first alternative (blue line) started from the SET reduction of Pd^II^ complex (**Int‐3**) to Pd^I^ complex (**Int‐4**) by the reduced photocatalyst Ir^II^. An outer‐sphere reductive elimination of **Int‐4** with the benzyl radical can afford the Pd‐product complex, which, upon dissociation of Pd^0^ species, would deliver the product and close the Pd catalytic cycle. A direct outer‐sphere reductive elimination of Pd^II^ complex (**Int‐3**) with the radical species was then tested (gray line). It was found that the energetic span of this route was 18.6 kcal/mol, which is 3.9 kcal/mol higher than the Pd^I^ route. In addition, we considered the feasibility of an inner‐sphere reductive elimination mechanism (light blue line). In this scenario, Pd^II^ complex (**Int‐3**) was first reduced to Pd^I^ complex (**Int‐4**), followed by the coordination of benzyl radical to deliver the Pd^II^ species (**Int‐9**). Afterwards an inner‐sphere reductive elimination mechanism would proceed to give the product complex. However, this route was significantly less favorable compared to the outer‐sphere alternatives due to the significantly higher energetic barrier. An analogous Pd^III^ mediated process was also considered. However, we couldn't allocate the corresponding intermediate with both benzyl and allyl group attached to the Pd^III^ center, presumably because of the steric bulkiness. Since the location of Pd^III^‐allyl/benzyl complex was not successful, the following reductive eliminations were also considered unlikely.

We then turned our attention to the dienylation direction. Three possible mechanisms regarding the formation of diene unit were evaluated. The first route started from the oxidative addition of PPTS to the Pd° complex, leading to the Pd–H species (**Int‐10**) (blue line). The insertion of one equivalent of propadiene would give a methylvinyl‐Pd intermediate (**Int‐12**). Another round of propadiene insertion would deliver the π–allyl complex **Int‐14**. A mechanistic alternative initiated from the protonation of Pd‐coordinated propadiene, giving rise to **Int‐18**. Upon pyridine ligand dissociation **Int‐12** would be formed (gray line). The remaining steps of the process are similar to the first alternative described before. However, when comparing with the first route, the overall energetic span for this pathway is higher. The third pathway includes the formation of a key five membered palladacycle through the cyclization of Pd species with two equivalents of propadiene (yellow–green line). Afterwards a protodepalladation would proceed to generate the *σ*‐allyl Pd complex (**Int‐20**), which can easily tautomerize to the π–allyl complex **Int‐14**. However, the high activation energy of **TS‐12** indicates that this route is quite unlikely. Starting from the **Int‐14**, we investigated three viable pathways leading to the final product. We first considered two outer‐sphere reductive elimination pathways based on Pd^I^ intermediate **Int‐15** (blue line) and Pd^II^ intermediate **Int‐14** (light blue line). Intriguingly, the results favored the reductive elimination at the lower valent palladium complex. Alternatively, an inner‐sphere reductive elimination from Pd^II^ complex **Int‐22** was also tested (light gray line). However, it was also proved to be a more energetically demanding pathway. Additionally, endeavor was also devoted to the Pd^III^ mediated process. Similar results as described above in the allylation direction were observed and the corresponding route was thus considered unlikely.

Based on the current calculation results, several general conclusions can be deduced. To start, in the context of π–allyl complex formation, a Pd^II^–H mediated propadiene insertion was an energetically more feasible choice compared with the protonation of Pd coordinated propadiene. Additionally, regarding the reductive elimination, a low valent Pd (Pd^I^ complex) promoted outer‐sphere reductive elimination excelled the high valent Pd (Pd^II^ complex) promoted reductive elimination, either through an outer‐sphere or an inner‐sphere fashion.

## Conclusion

To conclude, we demonstrated herein that propadiene, although industrially considered as an undesired component of the C3 fraction of the steam cracking process, can undergo a chemo‐divergent mono‐addition or dimerization‐addition under Pd catalysis to afford either an allylic or a dienylic structural motif. With a DHP derivative as the initial radical precursor and styrene derivatives as the initial radical acceptor, we have successfully embedded the aforementioned propadiene chemistry into the framework of metallaphotoredox dual catalysis to construct a portfolio of allylic and dienylic products. Notably, the reactions proceed through a three‐ and four‐component fashion for the allylic and dienylic direction, respectively. In both protocols two C(sp^3^)─C(sp^3^) bonds were added across the alkene π−‐bond and another C(sp^2^)─C(sp^2^) bond was formed in the course of the dienylation process. The reactions feature a profound functional group tolerance and are capable of performing late‐stage functionalization of pharmaceutical derivatives. Moreover, the methods also demonstrated an orthogonal reactivity compared to the thermal allylation with propadiene. DFT calculations revealed that for both cases the reductive elimination proceeded through an outer‐sphere manner at a low valent Pd center (Pd^I^ complex). We hope that both methodologies developed and the mechanistic insight posed can intrigue further endeavors into this field.

## Supporting Information

The authors have cited additional references within the Supporting Information.^[^ ^54^, ^55^, ^56^, ^57^, ^58^, ^59^, ^60^, ^61^, ^62^, ^63^ ^]^

## Conflict of Interests

The authors declare no conflict of interest.

## Supporting information



Supporting Information

Supporting Information

## Data Availability

The data that support the findings of this study are available in the supplementary material of this article.
