# Supplementary material for: Tackling the Reactivity of Propadiene: Palladium Metallaphotoredox Dual Catalyzed Multi‐Component Allylation and Dienylation of Styrenes
Source: Angew Chem Int Ed Engl. 2025 Nov 21;65(4):e18994. doi: 10.1002/anie.202518994 (PMC12828483; doi:10.1002/anie.202518994)
Supplement: Supplementary file 1 — Supporting Information [file ANIE-65-e18994-s002.docx]

**Supporting information**

Tackling the Reactivity of Propadiene: Palladium Metallaphotoredox Dual Catalyzed Multi-Component Allylation and Dienylation of Styrenes

Yi-Fei Yang, Felix Bauer, Bernhard Breit*

Institut für Organische Chemie, Albert-Ludwigs-Universität Freiburg, Albertstraße 21, 79104 Freiburg im Breisgau, Germany

E-mail: [bernhard.breit@chemie.uni-freiburg.de](mailto:bernhard.breit@chemie.uni-freiburg.de)

Table of content

[1 General Consideration 3](#_Toc211550365)

[2 Selected optimization study 4](#_Toc211550366)

[2.1 Allylation direction-Std. Cond. 1 4](#_Toc211550367)

[2.2 Allylation direction-Std. Cond. 2 11](#_Toc211550368)

[2.3 Dienylation direction 14](#_Toc211550369)

[3 General procedures 20](#_Toc211550370)

[3.1 Synthesis of starting materials 20](#_Toc211550371)

[3.2 Catalytic reactions 22](#_Toc211550372)

[4 Characterization data 24](#_Toc211550373)

[4.1 Starting materials 24](#_Toc211550374)

[4.2 Products 35](#_Toc211550375)

[4.3 Crystal Structure and Crystallographic Data 85](#_Toc211550376)

[5 Extended scope 89](#_Toc211550377)

[5.1 Allylation direction 90](#_Toc211550378)

[5.2 Dienylation direction 93](#_Toc211550379)

[6 Practicability investigation 94](#_Toc211550380)

[6.1 Gram scale reactions 94](#_Toc211550381)

[6.2 Sequential functionalization of pharmaceuticals 96](#_Toc211550382)

[7 Mechanistic investigation 102](#_Toc211550383)

[7.1 Radical capture experiments 102](#_Toc211550384)

[7.2 Deuterium labeling experiments 104](#_Toc211550385)

[7.3 Stochiometric experiment 107](#_Toc211550386)

[7.4 Post dienylation 108](#_Toc211550387)

[8 DFT calculation 109](#_Toc211550388)

[8.1 General Remarks 109](#_Toc211550389)

[8.2 Mechanistic Discussion 110](#_Toc211550390)

[8.3 Atomic Coordinates 131](#_Toc211550391)

[9 Copy of NMR spectral 258](#_Toc211550392)

[9.1 Starting materials 258](#_Toc211550393)

[9.2 Allylic products 278](#_Toc211550394)

[9.3 Dienylic products 328](#_Toc211550395)

[9.4 Practicability investigation 354](#_Toc211550396)

[10 Reference 363](#_Toc211550397)

## General Consideration

All reactions were performed using Schlenk technique in oven dried glassware under argon (argon 5.0, *Sauerstoffwerk Friedrichshafen).* An oil bath was used as heating source for all reactions at elevated temperatures. Catalytic reactions were performed in 8 mL screw-capped tubes. Unless otherwise noted, reagents, catalysts and ligands were purchased from commercial suppliers and used without further purification. Solvents were purchased in p.a. grade and, if not stated otherwise, used without further purification.

**Chromatography**

Unless otherwise noted, flash columns were performed on an *Interchim* Puriflash system equipped with either a normal phase silica gel column (type PF-SIHP/12G, 30μm particle size) eluting with Petroleum ether/EtOAc or a reverse phase C18 column (type PF-30C18H/25G, 30μm particle size) eluting with acetonitrile (MeCN) / water. Alternatively, silica gel 60 (230–400 mesh, *Machery-Nagel)* was used for manual chromatography.

Thin layer chromatography (TLC) was performed on aluminum plates coated with silica-gel 60F_254_ *(Merck).* The TLC plates were visualized by UV fluorescence (λ_max_= 254 nm) and/or staining with 1% w/v KMnO_4_ solution in 0.5 M aqueous K_2_CO_3_ and heating.

**Nuclear Magnetic Resonance**

Nuclear magnetic resonance (NMR) spectra were measured on a *Bruker Avance 400* (^1^H-NMR: 400 MHz, ^13^C-NMR: 101 MHz, ^1^H decoupled), *Bruker Avance III HD 500* (^1^H-NMR: 500 MHz, ^13^C-NMR: 126 MHz, ^1^H decoupled, ^19^F-NMR: 471 MHz) or Bruker *Avance III Neo 700* (^1^H-NMR: 700 MHz, ^13^C-NMR: 176 MHz, ^1^H decoupled) by the analytic department at the Institut für Organische Chemie und Biochemie, Universität Freiburg. All NMR spectra were reported in parts per million (ppm) and measured relative to the residual solvent peaks (CHCl_3_: ^1^H-NMR δ = 7.26 ppm (s), ^13^C-NMR δ = 77.1 ppm (t)). The ^1^H-NMR data are described as follows: Chemical shift (δ in ppm), multiplicity (s, singlet; d, doublet; t, triplet; q, quartet; quint, quintet; m, multiplet; br. s., broad signal), coupling constant (Hz), integration. Data for ^13^C-NMR spectra are described in terms of chemical shift (δ in ppm).

**Mass Spectrometry**

Mass spectra were recorded by the analytic department at the Institut für Organische Chemie und Biochemie, Universität Freiburg. ESI and APCI high resolution mass spectra were obtained on an *Agilent* 6545B LC/QTOF instrument or a *Thermo Scientific* Exactive Orbitrap instrument (2.5 µl/min samplsolution in a flow of 100-400 µl/min MeOH/MeCN; spray voltage 4-5 kV, ion transfer tube: 250-300 °C, vaporizer: 300-400 °C).

## Selected optimization study

### Allylation direction-Std. Cond. 1

#### Ligand screening

| **Entry** | **Ligand/%** | **Allylic/%** | **Dienylic/%** | **HA /%** |
| --- | --- | --- | --- | --- |
| 1 | rac-BINAP | 42 | n.d. | ~10 |
| 2 | rac-Tol-BINAP | 31 | n.d. | 10 |
| 3 | DPEPhos | 51 | 13 | 6 |
| 4 | CyDPEPhos | 28 | 6 | 13 |
| **5** | **Xantphos** | **76** | **11** | **n.d.** |
| 6 | dppe | 15 | 1 | 51 |
| 7 | dppp | 14 | 4 | 15 |
| 8 | bis(diPhphosphino)benzene | 3 | 3 | 17 |
| 9 | X-Phos | 31 | 9 | ~20 |
| 10 | RuPhos | 49 | 31 | 26 |
| 11 | Cy-JohnPhos | 18 | 24 | 29 |
| 12 | S-Phos | 27 | 21 | 14 |
| 13 | BrettPhos | 9 | 5 | 12 |
| 14 | t-butyldiphenylphosphine | 10 | 3 | 71 |
| 15 | PPh_3_ | 16 | 22 | 32 |
| 16 | P(o-Tol)_3_ | 5 | n.d. | 94 |
| 17 | PCy_3_ | 9 | n.d. | 84 |
| 18 | P(OPh)_3_ | 57 | 27 | 20 |
| 19 | L1 | 26 | 12 | 12 |
| 20 | L2 | 6 | 11 | 23 |
| 21 | L3 | 11 | n.d. | 87 |
| 22 | L4 | 43 | 23 | 4 |
| 23 | L5 | 36 | 16 | 28 |
| 24 | L6 | n.d. | 7 | 34 |
| 25 | L7 | 14 | 13 | 65 |
| 26 | L8 | n.d. | n.d. | 40 |

Reaction condition: Propa-1,2-diene (5.0 equiv.), 1,1-diphenylethylene (1.5 equiv.), iPr-DHP (0.1 mmol, 1.0 equiv.), [Ir(ppy)_2_dtbbpy]PF_6_ (1 mol%), [Pd(allyl)Cl]_2_ (2.5 mol%), Ligand (6 mol% or 12 mol%), 4-CF_3_-BzOH (10 mol%) in toluene (2 mL) were irradiated with 16 W blue LEDs for 16 h under argon.

#### Loading screening

| **Entry** | **Propadiene** | **DHP** | **Allylic/%** | **Dienylic/%** | **DHP (Remain)/%** |
| --- | --- | --- | --- | --- | --- |
| 1 | 1.0 | 1.5 | 44 | n.d. | 27 |
| **2** | **1.0** | **1.2** | **68** | n.d. | **8** |
| 3 | 1.0 | 1.0 | 62 | 5 | n.d. |
| 4 | 3.0 | 1.0 | 65 | 12 | n.d. |
| **5** | **5.0** | **1.0** | **72** | **11** | **n.d.** |
| 6 | 7.0 | 1.0 | 72 | 11 | n.d. |

Reaction condition: Propa-1,2-diene, 1,1-diphenylethylene (1.5 equiv.), iPr-DHP, [Ir(ppy)_2_dtbbpy]PF_6_ (0.5 mol%), [Pd(allyl)]_2_Cl_2_ (2.5 mol%), Xantphos (6 mol%), 4-CF_3_-BzOH (10 mol%) in toluene (2 mL) were irradiated with 16 W blue LEDs for 16 h under argon. The reaction was performed on a 0.1 mmol scale.

#### Pd catalysis screening

| **Entry** | **[Pd]** | **Allylic/%** | **Dienylic/%** | **HA /%** | **DHP (Remain)/%** |
| --- | --- | --- | --- | --- | --- |
| **1** | **[Pd(allyl)]_2_Cl_2_** | **71** | **n.d.** | **n.d.** | **9** |
| 2 | Pd_2_(dba)_3_ | <5 | n.d. | n.d. | Quanti. |
| **3** | **Pd(OAc)_2_** | **81** | **n.d.** | **n.d.** | **n.d.** |
| 4 | PdCl_2_ | 50 | n.d. | 44 | 28 |
| 5 | Pd(TFA)_2_ | 55 | n.d. | 6 | n.d. |
| **6** | **Pd(OPiv)_2_** | **75** | **n.d.** | **n.d.** | **n.d.** |
| 7 | Pd(MeCN)_4_(BF_4_)_2_ | 56 | 3 | 12 | n.d. |
| **8** | **(η^3^-cinnamyl)PdCp** | **84** | **n.d.** | **n.d.** | **6** |
| 9 | Dichloro[2-(4,5-diH-2-oxazolyl)quinoline]Pd | n.d. | n.d. | 94 | 7 |
| 10 | Pd(PPh_3_)_4_ | 5 | n.d. | 12 | Quanti. |
| 11 | Pd(PPh_3_)_4_ | n.d. | 13 | n.d. | n.d. |

Reaction condition: Propa-1,2-diene (0.1 mmol, 1.0 equiv.), 1,1-diphenylethylene (1.5 equiv.), iPr-DHP (1.2 equiv.), [Ir(ppy)_2_dtbbpy]PF_6_ (0.5 mol%), [Pd] (5 or 10 mol%), Xantphos (12 mol%), 4-CF_3_-BzOH (20 mol%) in toluene (2 mL) were irradiated with 16 W blue LEDs for 16 h under argon.

#### Preliminary acid additives screening

| **Entry** | **Acid Addi.** | **Allylic/%** | **Dienylic/%** | **HA/%** | **DHP (Remain)/%** |
| --- | --- | --- | --- | --- | --- |
| **1** | **4-CF_3_-BzOH** | **77** | **n.d.** | **n.d.** | **n.d.** |
| 2 | BzOH | 74 | 2 | 7 | 4 |
| 3 | 4-OMe-BzOH | 64 | 3 | 7 | 11 |
| 4 | AcOH | 23 | n.d. | 9 | 67 |
| 5 | TFA | 48 | n.d. | 7 | n.d. |
| 6 | PPTS | 51 | 3 | 19 | n.d. |
| 7 | (PhO)_2_P(O)OH | 36 | n.d. | n.d. | Quanti. |

Reaction condition: Propa-1,2-diene (0.1 mmol, 1.0 equiv.), 1,1-diphenylethylene (1.5 equiv.), iPr-DHP (1.2 equiv.), [Ir(ppy)_2_dtbbpy]PF_6_ (0.5 mol%), Pd(OAc)_2_ (10 mol%), Xantphos (12 mol%), Acid Add. (20 mol%) in toluene (2 mL) were irradiated with 16 W blue LEDs for 16 h under argon.

#### Concentration screening

| **Entry** | **Con.(Allene)** | **Allylic/%** |
| --- | --- | --- |
| 1 | 0.2 | 77 |
| **2** | **0.1** | **82** |
| 3 | 0.05 | 78 |
| 4 | 0.033 | 77 |
| 5 | 0.025 | 72 |

Reaction condition: Propa-1,2-diene (0.1 mmol, 1.0 equiv.), 1,1-diphenylethylene (1.5 equiv.), iPr-DHP (1.2 equiv.), [Ir(ppy)_2_dtbbpy]PF_6_ (0.5 mol%), Pd(OAc)_2_ (5 mol%), Xantphos (6 mol%), 4-CF_3_-BzOH (20 mol%) in toluene were irradiated with 16 W blue LEDs for 16 h under argon.

#### Photocatalysis screening

| **Entry** | **PC** | **Allylic/%** | **HA/%** | **DHP/%** |
| --- | --- | --- | --- | --- |
| 1 | 4CzIPN | 78 | 6 | 8 |
| **2** | **[Ir(ppy)_2_dtbbpy]PF_6_** | **82** | **7** | **-** |
| 3 | [Ir(ppy)_2_bpy]PF_6_ | 78 | 6 | 2 |
| 4 | [Ir(dFCF_3_ppy)_2_dtbbpy]PF_6_ | 76 | 6 | - |
| 5 | [Ir(dFCF_3_ppy)_2_bpy]PF_6_ | 77 | 6 | 6 |

Reaction condition: Propa-1,2-diene (0.1 mmol, 1.0 equiv.), 1,1-diphenylethylene (1.5 equiv.), iPr-DHP (1.2 equiv.), PC (0.5 mol%), Pd(OAc)_2_ (5 mol%), Xantphos (6 mol%), 4-CF_3_-BzOH (20 mol%) in toluene (1 mL) were irradiated with 16 W blue LEDs for 16 h under argon.

#### In depth acid additive screening

| **Entry** | **Acid** | **Yield/%** | **HA/%** | **DHP (Remain)/%** |
| --- | --- | --- | --- | --- |
| **1** | **4-Cl-BzOH** | **83** | **n.d.** | **3** |
| **2** | **4-F-BzOH** | **81** | **n.d.** | **5** |
| 3 | 4-CN-BzOH | 66 | n.d. | 3 |
| 4 | 3,5-di-CF₃-BzOH | 67 | n.d. | n.d. |
| 5 | 4-Br-BzOH | 59 | 23 | 8 |
| 6 | 3,5-di-F-BzOH | 78 | n.d. | n.d. |
| 7 | 3,5-di-Cl-BzOH | 71 | n.d. | n.d.- |
| 8 | PerfluoroBzOH | 48 | 14 | n.d. |
| 9 | Palmitic acid | 44 | 24 | 33 |
| 10 | Ph_3_CCO_2_H | 71 | 17 | 13 |

Reaction condition: Propa-1,2-diene (0.1 mmol, 1.0 equiv.), 1,1-diphenylethylene (1.5 equiv.), iPr-DHP (1.2 equiv.), [Ir(ppy)_2_dtbbpy]PF_6_ (0.5 mol%), Pd(OAc)_2_ (5 mol%), Xantphos (6 mol%), Acid add. (20 mol%) in toluene (1 mL) were irradiated with 16 W blue LEDs for 16 h under argon.

#### Acid loading screening

| **Entry** | **Acid/%** | **Allylic/%** | **HA/%** | **DHP/%** |
| --- | --- | --- | --- | --- |
| 1 | - | 19 | 19 | 51 |
| **2** | **5 mol%** | **85** | **7** | **3** |
| 3 | 10 mol% | 83 | 7 | 3 |
| 4 | 20 mol% | 77 | 10 | 2 |
| 5 | 50 mol% | 74 | 6 | - |
| 6 | 100 mol% | 70 | 8 | - |
| **7^a^** | **5 mol%** | **86** | **7** | **5** |
| **8^a, b^** | **5 mol%** | **88** | **16** | **16** |
| 9^a^ | 20 mol% | 83 | n.d. | 3 |

Reaction condition: Propa-1,2-diene (0.1 mmol, 1.0 equiv.), 1,1-diphenylethylene (1.5 equiv.), iPr-DHP (1.2 equiv.), [Ir(ppy)_2_dtbbpy]PF_6_ (0.5 mol%), Pd(OAc)_2_ (5 mol%), Xantphos (6 mol%), 4-CF_3_-BzOH in toluene (1 mL) were irradiated with 16 W blue LEDs for 16 h under argon. a: w/ 4-Cl-BzOH as acid additive. b: Performed on 0.2 mmol scale. w/ 1,1-diphenylethylene (1.8 equiv.), iPr-DHP (1.5 equiv.), Pd(OAc)_2_ (7.5 mol%), Xantphos (9 mol%) instead.

#### Base screening

| **Entry** | **Base** | **Yield/%** | **HA/%** | **DHP/%** |
| --- | --- | --- | --- | --- |
| 1 | Et_3_N | n.d. | 26 | 72 |
| 2 | DABCO | n.d. | 26 | 68 |
| 3 | Cs_2_CO_3_ | n.d. | n.d. | Quanti. |

Reaction condition: Propa-1,2-diene (0.1 mmol, 1.0 equiv.), 1,1-diphenylethylene (1.5 equiv.), iPr-DHP (1.2 equiv.), [Ir(ppy)_2_dtbbpy]PF_6_ (0.5 mol%), Pd(OAc)_2_ (5 mol%), Xantphos (6 mol%), Base (20 mol%) in toluene (1 mL) were irradiated with 16 W blue LEDs for 16 h under argon.

#### Ligand screening-Xantphos scaffold

| **Entry** | **Ligand/%** | **Yield/%** | **HA/%** | **deproto/%** |
| --- | --- | --- | --- | --- |
| **1** | **L1** | **80** | **7** | **6** |
| 2 | L2 | 22 | 59 | 5 |
| 3 | L3 | n.d. | 92 | n.d. |
| 4 | L4 | 77 | 6 | 7 |
| 5 | L5 | n.d. | n.d. | 7 |

Reaction condition: Propa-1,2-diene (0.2 mmol, 1.0 equiv.), 1,1-diphenylethylene (1.5 equiv.), iPr-DHP (1.2 equiv.), [Ir(ppy)_2_dtbbpy]PF_6_ (0.5 mol%), Pd(OAc)_2_ (7.5 mol%), Xantphos (9 mol%), 4-Cl-BzOH (7.5 mol%) in toluene (1 mL) were irradiated with 16 W blue LEDs for 16 h under argon.

#### 1 atm. of propadiene

| **Entry** | **diPhethylene(eq.)** | **DHP(eq.)** | **Allylic/%** | **Dienylic/%** | | **HA/%** | **deproto/%** |
| --- | --- | --- | --- | --- | --- | --- | --- |
| 1 | 1.5 | 1.0 | 32 | 35 | 4 | | 4 |
| 2 | 1.0 | 1.2 | 34 | 27 | 4 | | 4 |

Reaction condition: Propa-1,2-diene (1 atm.), 1,1- diphenylethylene, iPr-DHP, [Ir(ppy)_2_dtbbpy]PF_6_ (0.5 mol%), Pd(OAc)_2_ (7.5 mol%), Xantphos (9 mol%), 4-Cl-BzOH (7.5 mol%) in toluene (1 mL) were irradiated with 16 W blue LEDs for 16 h under argon. Propadiene was bubbled into the Schlenk tube for 1 min to ensure a propadiene atmosphere. The reaction was performed on 0.2 mmol scale.

#### Control experiments

| **Entry** | **w/o** | **Allylic/%** | **Dienylic/%** | **HA/%** | **DHP (Remain)/%** |
| --- | --- | --- | --- | --- | --- |
| **1** | **-** | **88** | **n.d.** | **16** | **16** |
| 2 | Pd/L | n.d. | n.d. | Quanti. | n.d. |
| 3 | Pd | n.d. | 23 | n.d. | 53 |
| 4 | Acid | 29 | n.d. | 34 | 47 |
| 5 | PC | 16 | n.d. | n.d. | 90 |
| 6 | *hv* | n.d. | n.d. | n.d. | Quanti. |
| 7 | argon protection | ~14 | n.d. | n.d. | n.d. |

Reaction condition: Propa-1,2-diene (0.2 mmol, 1.0 equiv.), 1,1-diphenylethylene (1.8 equiv.), iPr-DHP (1.5 equiv.), [Ir(ppy)_2_dtbbpy]PF_6_ (0.5 mol%), Pd(OAc)_2_ (7.5 mol%), Xantphos (9 mol%), 4-Cl-BzOH (5 mol%) in toluene (2 mL) were irradiated with 16 W blue LEDs for 16 h under argon.

### Allylation direction-Std. Cond. 2

#### Pd catalyst screening

| **Entry** | **Pd** | **Allylic/%** | **Dienylic/%** | **deproto/%** |
| --- | --- | --- | --- | --- |
| 1 | Pd(OAc)_2_ | n.d. | 61 | 14 |
| 2 | Pd(OPiv)_2_ | n.d. | 60 | 17 |
| 3 | Pd(OTFA)_2_ | 65 | n.d. | 74 |
| 4 | (η^3^-cinnamyl)PdCp | 56 | n.d. | 22 |
| 5 | Pd_2_(dba)_3_ | 63 | n.d. | 12 |
| **6** | **Pd_2_(4,4’-MeO-dba)_3_** | **88** | **n.d.** | **15** |
| 7 | Pd_2_(dba)_3_·CHCl_3_ | 76 | n.d. | 39 |
| 8 | Pd(acac)_2_ | 70 | n.d. | 24 |
| 9 | [Pd(Ally)]_2_Cl_2_ | 51 | n.d. | 66 |

Reaction condition: Propa-1,2-diene (0.4 mmol, 2*1.0 equiv.), 1,1-diphenylethylene (4.0 equiv.), iPr-DHP (1.2 equiv.), [Ir(ppy)_2_dtbbpy]PF_6_ (0.5 mol%), [Pd] (7.5 mol%), (2-furyl)_3_P (18 mol%), PPTS (10 mol%) in toluene (1 mL) were irradiated with 16 W blue LEDs for 16 h under argon.

#### Parallel screening of ligand with different acid additives

| **Entry** | **Ligand** | **Acid** | **Allylic/%** | **Dienylic/%** | **HA/%** | | **Deproto/%** |
| --- | --- | --- | --- | --- | --- | --- | --- |
| **1** | **(2-furyl)_3_P** | **PPTS** | **92** | **n.d.** | **15** | **15** | |
| 2 | (4-CF_3_Ph)_3_P | PPTS | n.d. | n.d. | Quanti. | 19 | |
| 3 | Xantphos | PPTS | 52 | n.d. | 40 | n.d. | |
| 4 | (2-furyl)_3_P | 4-CF_3_-BzOH | n.d. | 20 | 34 | 30 | |
| 5 | (4-CF_3_Ph)_3_P | 4-CF_3_-BzOH | n.d. | n.d. | 95 | 11 | |
| 6 | Xantphos | 4-CF_3_-BzOH | 93 | n.d. | 9 | n.d. | |

Reaction condition: Propa-1,2-diene (0.1 mmol, 1.0 equiv.), 1,1-diphenylethylene (1.8 equiv.), iPr-DHP (1.5 equiv.), [Ir(ppy)_2_dtbbpy]PF_6_ (0.5 mol%), Pd_2_(4,4’-MeO-dba)_3_ (3.75 mol%), Ligand (9 or 18 mol%), Acid add. (10 mol%) in toluene (1 mL) were irradiated with 16 W blue LEDs for 16 h under argon.

#### Rapid deviation study

| **Entry** | **Ligand** | **Allylic/%** | **HA/%** | **Deproto/%** | **DHP** |
| --- | --- | --- | --- | --- | --- |
| 1 | P(OPh)_3_ | n.d. | Quanti. | n.d. | n.d. |
| 2 | (2-thienyl)_3_P | 12 | 65 | n.d. | n.d. |
| **3^a^** | **(2-furyl)_3_P** | **83** | **21** | **16** | **6** |
| 4^b^ | (2-furyl)_3_P | 31 | 31 | 10 | n.d. |
| 5^a, c^ | (2-furyl)_3_P | 80 | 21 | 19 | 9 |
| 6^a, d^ | (2-furyl)_3_P | 82 | 27 | 16 | 8 |

Reaction condition: Propa-1,2-diene (0.1 mmol, 1.0 equiv.), 1,1-diphenylethylene (1.8 equiv.), iPr-DHP (1.5 equiv.), [Ir(ppy)_2_dtbbpy]PF_6_ (0.5 mol%), Pd_2_(4,4’-MeO-dba)_3_ (3.75 mol%), Ligand (18 mol%), Acid add. (10 mol%) in toluene (1 mL) were irradiated with 16 W blue LEDs for 16 h under argon. a: w/ TCE (CHClCCl_2_) as the internal standard. b: w/ P(O)(OPh)_2_OH. c: w/ 5 mol% acid. d: w/ Pd_2_(dba)_3_.

#### 1 atm. of propadiene

| **Entry** | **diPhethylene(eq.)** | **DHP(eq.)** | **Allylic/%** | **Dienylic/%** | | **HA/%** | **deproto/%** |
| --- | --- | --- | --- | --- | --- | --- | --- |
| 1 | 1.5 | 1.0 | 75 | 7 | trace | | 16 |
| 2 | 1.0 | 1.2 | 87 | 5 | trace | | 13 |

Reaction condition: Propa-1,2-diene, 1,1-diphenylethylene, iPr-DHP, [Ir(ppy)_2_dtbbpy]PF_6_ (0.5 mol%), Pd_2_(MeO-dba)_3_ (3.75 mol%), (2-furyl)_3_P (18 mol%), PPTS (10 mol%) in toluene (2 mL) were irradiated with 16 W blue LEDs for 16 h under argon. Propadiene was bubbled into the Schlenk tube for 1 min to ensure a propadiene atmosphere. The reaction was performed on 0.2 mmol scale.

#### Control experiments

| **Entry** | **w/o** | **Allylic/%** | **Dienylic/%** | **HA/%** | **DHP/%** |
| --- | --- | --- | --- | --- | --- |
| 1 | - | **92** | **n.d.** | **15** | **n.d.** |
| 2 | Pd/L | n.d. | n.d. | 92 | n.d. |
| 3 | Pd | n.d. | n.d. | 89 | n.d. |
| 4 | Acid | n.d. | 23 | 94 | n.d. |
| 5 | PC | n.d. | n.d. | n.d. | Quanti. |
| 6 | *hv* | n.d. | n.d. | n.d. | Quanti. |
| 7 | argon protection | n.d. | ~18 | n.d. | n.d. |

Reaction condition: Propa-1,2-diene (0.2 mmol, 1.0 equiv.), 1,1-diphenylethylene (1.8 equiv.), iPr-DHP (1.5 equiv.), [Ir(ppy)_2_dtbbpy]PF_6_ (0.5 mol%), Pd_2_(MeO-dba)_3_ (3.75 mol%), (2-furyl)_3_P (18 mol%), PPTS (10 mol%) in toluene (2 mL) were irradiated with 16 W blue LEDs for 16 h under argon.

### Dienylation direction

#### Ligand screening

| **Entry** | **Ligand** | **Dienylic/%** | **Deproto/%** |
| --- | --- | --- | --- |
| 1 | (4-CF_3_Ph)_3_P | 27 | 33 |
| 2 | (perfluoroPh)_3_P | 28 | 7 |
| **3** | **(2-furyl)_3_P** | **42** | **35** |
| 4 | (2-thienyl)_3_P | 32 | 24 |
| 5 | *^t^*Bu-DavePhos | trace | - |
| 6 | L1 | 12 | 14 |

Reaction condition: Propa-1,2-diene (0.2 mmol, 2*1.0 equiv.), 1,1-diphenylethylene (1.5 equiv.), iPr-DHP (1.2 equiv.), [Ir(ppy)_2_dtbbpy]PF_6_ (0.5 mol%), Pd(OAc)_2_ (7.5 mol%), Ligand (18 mol%), 4-CF_3_BzOH (10 mol%) in toluene (1 mL) were irradiated with 16 W blue LEDs for 16 h under argon.

#### Acid screening

| **Entry** | **Acid add.** | **Dienylic/%** | **Allylic/%** | **deproto/%** |
| --- | --- | --- | --- | --- |
| 1 | 4OMeBzOH | 25 | n.d. | 16 |
| 2 | 4-CF_3_BzOH | 25 | n.d. | 25 |
| 3 | 3,5-diCF_3_BzOH | 27 | n.d. | 23 |
| 4 | AcOH | 24 | n.d. | 17 |
| 5 | TFA | 29 | 17 | 25 |
| **6** | **PPTS** | **44** | **n.d.** | **11** |
| 7 | (PhO)_2_P(O)OH | 40 | n.d. | 18 |
| 8 | - | 37 | n.d. | 26 |

Reaction condition: Propa-1,2-diene (0.2 mmol, 2*1.0 equiv.), 1,1-diphenylethylene (1.5 equiv.), iPr-DHP (1.2 equiv.), [Ir(ppy)_2_dtbbpy]PF_6_ (0.5 mol%), Pd(OAc)_2_ (7.5 mol%), (2-furyl)_3_P (18 mol%), acid add. (10 mol%) in toluene (1 mL) were irradiated with 16 W blue LEDs for 16 h under argon.

#### Acid screening

| **Entry** | **Base add.** | **Yield/%** | **HA/%** | **deproto/%** |
| --- | --- | --- | --- | --- |
| 1 | DABCO | 23 | - | 10 |
| 2 | Et_3_N | 25 | - | 7 |
| 3 | Pyidine | 22 | - | 14 |
| 4 | Cs_2_CO_3_ | 12 | 63 | 4 |

Reaction condition: Propa-1,2-diene (0.2 mmol, 2*1.0 equiv.), 1,1-diphenylethylene (1.5 equiv.), iPr-DHP (1.2 equiv.), [Ir(ppy)_2_dtbbpy]PF_6_ (0.5 mol%), Pd(OAc)_2_ (7.5 mol%), (2-furyl)_3_P (18 mol%), base add. (10 mol%) in toluene (1 mL) were irradiated with 16 W blue LEDs for 16 h under argon.

#### Deviation from standard condition

| **Entry** | **Deviation from Std. Cond.** | **Dienylic/%** | **Allylic/%** | **deproto/%** |
| --- | --- | --- | --- | --- |
| 1 | - | 46 | n.d. | 10 |
| 2 | w/ 5 equiv. PDE and 1 equiv. DHP | 21 | 5 | 8 |
| 3 | w/ 2 equiv. diPhethylene | 52 | n.d. | 12 |
| **4** | **w/ 3 equiv. diPhethylene** | **60** | **n.d.** | **14** |
| 5 | w/ Styrene instead of diPhethylene | 28 | n.d. | 3 |
| 6 | w/ (2-thienyl)_3_P | 35 | n.d. | 10 |
| 7 | w/ 20 mol% PPTS | 40 | 8 | 14 |
| 8 | w/ DCE as Sol. | 35 | 3 | 12 |

Reaction condition: Propa-1,2-diene (0.2 mmol, 2*1.0 equiv.), 1,1-diphenylethylene (1.5 equiv.), iPr-DHP (1.2 equiv.), [Ir(ppy)_2_dtbbpy]PF_6_ (0.5 mol%), Pd(OAc)_2_ (7.5 mol%), (2-furyl)_3_P (18 mol%), PPTS (10 mol%) in toluene (1 mL) were irradiated with 16 W blue LEDs for 16 h under argon.

#### Photocatalyst screening

| **Entry** | **PC** | **Dienylic/%** | **Deproto/%** |
| --- | --- | --- | --- |
| 1 | 4CzIPN | 57 | 25 |
| 2 | Ir[(ppy)_2_bpy]PF_6_ | 48 | 33 |
| 3 | Ir[(dFCF_3_ppy)_2_dtbbpy]PF_6_ | 52 | 25 |
| 4 | Ir[(dFCF_3_ppy)_2_bpy]PF_6_ | 56 | 28 |
| **5** | **Ir[(ppy)_2_dtbbpy]PF_6_** | **60** | **14** |
| 6 | Ir(ppy)_3_ | 5 | 31 + 77 DHP |

Reaction condition: Propa-1,2-diene (0.2 mmol, 2*1.0 equiv.), 1,1-diphenylethylene (4.0 equiv.), iPr-DHP (1.2 equiv.), [Ir(ppy)_2_dtbbpy]PF_6_ (0.5 mol%), Pd(OAc)_2_ (7.5 mol%), (2-furyl)_3_P (18 mol%), PPTS (10 mol%) in toluene (1 mL) were irradiated with 16 W blue LEDs for 16 h under argon.

#### Pd catalyst screening

| **Entry** | **Pd** | **Dienylic/%** | **Allylic/%** | **Deproto/%** |
| --- | --- | --- | --- | --- |
| **1** | **Pd(OAc)_2_** | **61** | **n.d.** | **14** |
| 2 | Pd(OPiv)_2_ | 60 | n.d. | 17 |
| 3 | Pd(OTFA)_2_ | n.d. | 65 | 74 |
| 4 | (η^3^-cinnamyl)PdCp | n.d. | 56 | 22 |
| 5 | Pd_2_(dba)_3_ | n.d. | 63 | 12 |
| 6 | Pd_2_(4,4’-MeO-dba)_3_ | n.d. | 88 | 15 |
| 7 | Pd_2_(dba)_3_·CHCl_3_ | n.d. | 76 | 39 |
| 8 | Pd(acac)_2_ | n.d. | 70 | 24 |
| 9 | [Pd(Ally)]_2_Cl_2_ | n.d. | 51 | 66 |

Reaction condition: Propa-1,2-diene (0.2 mmol, 2*1.0 equiv.), 1,1-diphenylethylene (4.0 equiv.), iPr-DHP (1.2 equiv.), [Ir(ppy)_2_dtbbpy]PF_6_ (0.5 mol%), [Pd] (7.5 mol%), (2-furyl)_3_P (18 mol%), PPTS (10 mol%) in toluene (1 mL) were irradiated with 16 W blue LEDs for 16 h under argon.

#### Acid additive screening-Counter anion

| **Entry** | **Acid add.** | **Dienylic/%** | **Allylic/%** | **Deproto/%** |
| --- | --- | --- | --- | --- |
| 1 | PPTS | 63 | n.d. | 20 |
| 2 | Py·TFA | 28 | 14 | 63 |
| 3 | Py·TfOH | 44 | n.d. | 40 |
| 4 | Collidine·TfOH | 61 | n.d. | 12 |
| 5 | Py·MsOH | 67 | n.d. | 23 |
| 6 | Py·HBF_4_ | 50 | n.d. | 39 |
| 7 | Py·HCl | 21 | 27 | 38 |

Reaction condition: Propa-1,2-diene (0.4 mmol, 2*1.0 equiv.), 1,1-diphenylethylene (3.0 equiv.), iPr-DHP (1.2 equiv.), [Ir(ppy)_2_dtbbpy]PF_6_ (1.0 mol%), Pd(OAc)_2_ (10 mol%), (2-furyl)_3_P (24 mol%), Acid add. (10 mol%) in toluene (2 mL) were irradiated with 16 W blue LEDs for 16 h under argon.

#### Acid additive screening-Pyridine core

| **Entry** | **Acid add.** | **Dienylic/%** | **deproto/%** |
| --- | --- | --- | --- |
| 1 | PPTS | 63 | 20 |
| 4 | Py·TFA | 28 | 63 |
| 5 | 2-Picoline·TsOH | 63 | 12 |
| 6 | 2,6-Lutidine·TsOH | 61 | 17 |
| 7 | 2,4,6-Colidine·TsOH | 59 | 12 |
| 8 | Py·MsOH | 56 | 26 |
| 9 | 2-Picoline·MsOH | 61 | 14 |
| 10 | 4-CN-Py·TsOH | 42 | 18 |
| 11 | 4-CF_3_-Py·TsOH | 50 | 13 |
| 12 | DMAP·TsOH | 53 | 27 |
| **13** | **4-OMe-Py·TsOH** | **68** | **18** |

Reaction condition: Propa-1,2-diene (0.4 mmol, 2*1.0 equiv.), 1,1-diphenylethylene (3.0 equiv.), iPr-DHP (1.2 equiv.), [Ir(ppy)_2_dtbbpy]PF_6_ (1.0 mol%), Pd(OAc)_2_ (10 mol%), (2-furyl)_3_P (24 mol%), Acid add. (10 mol%) in toluene (2 mL) were irradiated with 16 W blue LEDs for 16 h under argon.

#### 1 atm of propadiene

| **Entry** | **diPhethylene(eq.)** | **DHP(eq.)** | **Allylic/%** | **Dienylic/%** | | **HA/%** | **deproto/%** |
| --- | --- | --- | --- | --- | --- | --- | --- |
| 1 | 3.0 | 1.0 | trace | 61 | 4 | | 14 |
| 2 | 1.0 | 1.2 | 6 | 30 | 4 | | 8 |

Reaction condition: Propa-1,2-diene (1 atm.), 1,1-diphenylethylene, iPr-DHP, [Ir(ppy)_2_dtbbpy]PF_6_ (0.5 mol%), Pd(OAc)_2_ (7.5 mol%), (2-furyl)_3_P (18 mol%), 4-OMe-Py·TsOH (10 mol%) in toluene (2 mL) were irradiated with 16 W blue LEDs for 16 h under argon. Propadiene was bubbled into the Schlenk tube for 1 min to ensure a propadiene atmosphere. The reaction was performed on 0.2 mmol scale.

#### Control experiments

| **Entry** | **w/o** | **Dienylic/%** | **HA/%** | **DHP (Remain)/%** |
| --- | --- | --- | --- | --- |
| 1 | - | 63 | n.d. | n.d. |
| 2 | Pd/L | n.d. | 89 | n.d. |
| 3 | Pd | 15 | 80 | n.d. |
| 4 | Acid | 37 | 21 | 20 |
| 5 | PC | n.d. | n.d. | Quanti. |
| 6 | *hv* | n.d. | n.d. | Quanti. |
| 7 | argon protection | 34 | n.d. | n.d. |

Reaction condition: Propa-1,2-diene (0.4 mmol, 2*1.0 equiv.), 1,1-diphenylethylene (3.0 equiv.), iPr-DHP (1.2 equiv.), [Ir(ppy)_2_dtbbpy]PF_6_ (0.5 mol%), Pd(OAc)_2_ (7.5 mol%), (2-furyl)_3_P (18 mol%), 4-OMe-Py·TsOH (10 mol%) in toluene (2 mL) were irradiated with 16 W blue LEDs for 16 h under argon.

## General procedures

### Synthesis of starting materials

#### Propadiene stock solution

Propadiene was prepared according to a slightly modified procedure of Doye.^[1]^ To a 500 mL flask equipped with dropping funnel, reflux condenser, magnetic stirring bar and magnesium turnings (4.86 g, 12.4 mL, 0.200 mol), tetrahydrofuran (200 mL) was added. All joints were tightly secured with fork clamps. A solution of 2,3-dichloropropene (15.0 g, 0.135 mol) in tetrahydrofuran (60 mL) was added for 40 min. Off-gassing product was allowed to escape through a rubber tube and cannula attached to the reflux condenser. Once the tubing and cannula were thoroughly flushed, the cannula was inserted into a Schlenk tube with dried, degassed toluene (80 mL) and the allene was bubbled through the solvent at room temperature. The concentration of the resulting saturated Propa-1,2-diene solution was found to be 0.90 M according to ^1^H NMR.

*Note 1: The stock solution of propadiene in other common solvents can also be prepared in this way.*

*Note 2: Upon properly* *sealing, the concentration of propadiene stock solution typically can remain steady for a rather long time. A slight decrease of concentration along time is normal and unavoidable based on our experience. The rate of deceasing to certain extend depended on the property of solvent used. Toluene and acetonitrile were proved to be two very robust choices.*

*Note 3: Although the concentration is typically stable, it is still recommended to renew the rubber septum regularly and test the concentration before setting up a reaction.*

*Note 4: Propadiene stock solution prepared this way inevitably contains trace amount of THF vaporized from the reaction flask. Empirically, the existence of trace amount of THF won’t affect the desired reactivity of propadiene for the current manifolds.*

#### Styrene derivatives (*GP 1*)

The reaction was performed following a modified literature procedure.^[2]^ To an oven dried round bottom flask equipped with a magnetic stirring bar was charged with Ph_3_PMeBr (1.2 equiv.). The flask was sealed with a rubber septum, evacuated and refilled with argon for three times before THF (0.2 M) was added. The mixture was cooled to 0 °C and n-BuLi (2.5 M in hexane, 1.2 equiv.) was added dropwise over 10 min. Then the reaction system was stirred at 0 °C for 30 min before a THF solution of the corresponding ketone (1 M, 1.0 equiv.) was added dropwise. The flask was warmed up to room temperature and stirred overnight. Upon completion, the crude mixture was quenched with saturated aqueous NH_4_Cl and the water phase was extracted with ethyl acetate twice. The organic phases were combined, dried with Na_2_SO_4_, filtered and evaporated under reduced pressure. The residue was then subjected to flash column chromatography on silica gel (PE:EtOAc = 100:1) to afford the title compound.

#### DHP derivatives (*GP 2*)

The reaction was performed following a modified literature procedure.^[3]^ To an oven dried round bottom flask equipped with a magnetic stirring bar was charged with Bu_4_NHSO_4_ (12 mol%). The flask was sealed with a rubber septum, evacuated and refilled with argon for three times before the corresponding aldehyde (1.0 equiv.), ethyl acetoacetate (1.0 equiv.), ethyl 3-aminocrotonate (1.0 equiv.) and ethylene glycol (2.5 M) was added sequentially. The resulting reaction mixture was then allowed to be heated at 80 °C for 3-4 hours until one of the starting materials was completely consumed. The mixture was then cooled to room temperature, diluted with EtOAc and washed with brine. The aqueous phase was extracted two times with EtOAc before the organic phases were combined, dried with Na_2_SO_4_ and evaporated under reduced pressure. The residue was then subjected to flash column chromatography on silica gel (PE:EtOAc gradient from 95:5 to 60:40) to afford the title compound. Optional further purification can be conducted through recrystallization from PE/EtOAc (v(PE):v(EtOAc) = 8:1) at -20 °C.

*Note: The 1,4-DHP itself is stable and can be stored at 4 °C for a rather long time. However, it is* *prone to decompose in CDCl_3_. Therefore, it is recommended to measure the NMR sample as soon as possible. The stability of 1,4-DHP in other common deuterated solvents was not tested.*

### Catalytic reactions

#### Catalytic allylation-Stand. Condi. 1 (*GP 3*)

To an oven dried 8 mL Schlenk tube equipped with a magnetic stirring bar was charged with Pd(OAc)_2_ (3.4 mg, 0.015 mmol, 7.5 mol%), Xantphos (10.4 mg, 0.018 mmol, 9 mol%) and [Ir(ppy)_2_dtbbpy]PF_6_ (0.9 mg, 0.001 mmol, 0.5 mol%) in air. The tube was evacuated and refilled with argon for three times before toluene was added. The tube was then sealed with a screw cap and the mixture was allowed to stir for 30 min before 4-Cl-BzOH (1.6 mg, 0.01 mmol, 5 mol%), styrene (0.36 mmol, 1.8 equiv.), DHP derivative (0.3 mmol, 1.5 equiv.) and the stock solution of Propa-1,2-diene (0.2 mmol, 1.0 equiv.) was subjected to the reaction mixture under an argon counterflow. The overall concentration of propadiene set to be 0.1 M *via* adjusting the volume of toluene added during pre-string. The resulting mixture was allowed to stir for 16 h under the irradiation of a 16 W Blue LEDs strip. A cooling fan was place above the tubes to keep the temperature at room temperature during the reaction. Upon completion, the crude mixture was evaporated under reduced pressure and subjected to flash column chromatography to afford the pure product.

#### Catalytic allylation-Stand. Condi. 2 (*GP 4*)

To an oven dried 8 mL Schlenk tube equipped with a magnetic stirring bar was charged with Pd_2_(4,4'-OMe-dba)_3_ (8.2 mg, 0.0075 mmol, 3.75 mol%), (2-furyl)_3_P (8.4 mg, 0.036 mmol, 18 mol%) and [Ir(ppy)_2_dtbbpy]PF_6_ (0.9 mg, 0.001 mmol, 0.5 mol%) in air. The tube was evacuated and refilled with argon for three times before toluene was added. The tube was then sealed with a screw cap and the mixture was allowed to stir for 30 min before PPTS (5.0 mg, 0.02 mmol, 10 mol%), styrene (0.36 mmol, 1.8 equiv.), DHP derivative (0.3 mmol, 1.5 equiv.) and the stock solution of Propa-1,2-diene (1.0 equiv.) was subjected to the reaction mixture under an argon counterflow. The overall concentration of propadiene set to be 0.1 M *via* adjusting the volume of toluene added during pre-string. The resulting mixture was allowed to stir for 16 h under the irradiation of a 16 W Blue LEDs strip. A cooling fan was place above the tubes to keep the temperature at room temperature during the reaction. Upon completion, the crude mixture was evaporated under reduced pressure and subjected to flash column chromatography to afford the pure product.

#### Catalytic Dienylation (*GP 5*)

To an oven dried 8 mL Schlenk tube equipped with a magnetic stirring bar was charged with Pd(OAc)_2_ (3.4 mg, 0.015 mmol, 7.5 mol%), (2-furyl)_3_P (8.4 mg, 0.036 mmol, 18 mol%) and [Ir(ppy)_2_dtbbpy]PF_6_ (0.9 mg, 0.001 mmol, 0.5 mol%) in air. The tube was evacuated and refilled with argon for three times before toluene was added. the tube was sealed with a screw cap and the mixture was allowed to stir for 30 min before 4-OMe-Py·TsOH (5.6 mg, 0.02 mmol, 10 mol%), diphenylethylene (0.6 mmol, 3.0 equiv.), DHP derivative (0.30 mmol, 1.5 equiv.) and the stock solution of Propa-1,2-diene (0.4 mmol, 2*1.0 equiv.) was subjected to the reaction mixture under an argon counterflow. The overall concentration of propadiene set to be 0.2 M *via* adjusting the volume of toluene added during pre-string. The resulting mixture was allowed to stir for 16 h under the irradiation of a 16 W Blue LEDs strip. A cooling fan was place above the tubes to keep the temperature at room temperature during the reaction. Upon completion, the crude mixture was evaporated under reduced pressure and subjected to flash column chromatography to afford the final product.

## Characterization data

### Starting materials

##### (4-(1-phenylvinyl)phenyl)(*p*-tolyl)sulfane (2-17)

([See Spectra](#_1H_NMR_Spectrum_78))

The reaction was performed following the ***GP 1*** on a 10 mmol scale. The residue was purified by flash column chromatography on silica gel (PE:EtOAc = 100:1) to afford the title compound as a white solid (2.79 g, 92%).

**TLC** (SiO_2_): R*_f_* = 0.20 (PE)

**^1^H NMR (400 MHz, CDCl_3_)** δ 7.35 – 7.27 (m, 7H), 7.24 – 7.21 (m, 2H), 7.21 – 7.17 (m, 2H), 7.15 – 7.14 (m, 1H), 7.14 – 7.11 (m, 1H), 5.43 (d, *J* = 1.2 Hz, 1H), 5.40 (d, *J* = 1.2 Hz, 1H), 2.33 (t, *J* = 0.7 Hz, 3H).

**^13^C NMR (101 MHz, CDCl_3_)** δ 149.5, 141.4, 139.6, 137.9, 136.9, 132.6, 131.0, 130.2, 129.3, 128.9, 128.4, 128.3, 127.9, 114.3, 21.2.

**HRMS (pos. APCI)** m/z: [M+H]^+^ Calcd for C_21_H_19_S 303.1202. Found 303.1200.

**Melting point:** 51-52 °C

##### 2-(3-(1-phenylvinyl)phenyl)propanenitrile (2-19)

([See Spectra](#_1H_NMR_Spectrum_79))

The reaction was performed following the ***GP 1*** on a 10 mmol scale. The residue was purified by flash column chromatography on silica gel (PE:EtOAc = 20:1) to afford the title compound as a yellowish oil (2.79 g, 92%).

**TLC** (SiO_2_): R*_f_* = 0.21 (PE:EtOAc = 20:1)

**^1^H NMR (400 MHz, CDCl_3_)** δ 7.37 – 7.26 (m, 9H), 5.50 (d, *J* = 1.1 Hz, 1H), 5.47 (d, *J* = 1.1 Hz, 1H), 3.86 (q, *J* = 7.3 Hz, 1H), 1.62 (d, *J* = 7.3 Hz, 3H).

**^13^C NMR (101 MHz, CDCl_3_)** δ 149.5, 142.6, 141.1, 137.2, 129.1, 128.3, 128.2, 128.1, 128.0, 126.7, 126.0, 121.6, 115.0, 31.3, 21.5.

**HRMS (pos. APCI)** m/z: [M+H]^+^ Calcd for C_17_H_16_N 234.1277. Found 234.1277.

##### 4,4,5,5-tetramethyl-2-(4-(1-phenylvinyl)phenyl)-1,3,2-dioxaborolane (2-20)

([See Spectra](#_1H_NMR_Spectrum_81))

The reaction was performed following the ***GP 1*** on a 3.3 mmol scale. The residue was purified by flash column chromatography on silica gel (PE:EtOAc = 100:1) to afford the title compound as a white solid (0.72 g, 71%).

**TLC** (SiO_2_): R*_f_* = 0.38 (PE:EtOAc = 100:1)

**^1^H NMR (500 MHz, CDCl_3_)** δ 7.80 – 7.75 (m, 2H), 7.36 – 7.33 (m, 2H), 7.33 – 7.29 (m, 5H), 5.49 (d, *J* = 1.2 Hz, 1H), 5.48 (d, *J* = 1.2 Hz, 1H), 1.35 (s, 12H).

**^13^C NMR (126 MHz, CDCl_3_)** δ 150.1, 144.4, 141.4, 134.7, 128.34, 128.26, 127.8, 127.7, 114.9, 83.9, 25.0.

**HRMS (pos. APCI)** m/z: [M+H]^+^ Calcd for C_20_H_24_O_2_B 307.1864. Found 307.1860.

**Melting point:** 93-95 °C

##### 4-bromo-1-chloro-2-(1-(4-ethoxyphenyl)vinyl)benzene (2-26)

([See Spectra](#_1H_NMR_Spectrum_80))

The reaction was performed following the ***GP 1*** on a 10 mmol scale. The residue was purified by flash column chromatography on silica gel (PE:EtOAc = 40:1) to afford the title compound as a colorless viscous oil (2.85 g, 84%).

**TLC** (SiO_2_): R*_f_* = 0.44 (PE:EtOAc = 40:1)

**^1^H NMR (400 MHz, CDCl_3_)** δ 7.45 (d, *J* = 2.4 Hz, 1H), 7.39 (dd, *J* = 8.5, 2.5 Hz, 1H), 7.24 (d, *J* = 8.5 Hz, 1H), 7.18 – 7.14 (m, 2H), 6.84 – 6.80 (m, 2H), 5.73 (d, *J* = 0.9 Hz, 1H), 5.16 (d, *J* = 0.9 Hz, 1H), 4.02 (q, *J* = 7.0 Hz, 2H), 1.40 (t, *J* = 7.0 Hz, 3H).

**^13^C NMR (101 MHz, CDCl_3_)** δ 159.0, 145.9, 143.0, 134.2, 132.5, 131.8, 131.6, 131.2, 127.7, 120.3, 114.9, 114.4, 63.5, 14.9.

**HRMS (pos. APCI)** m/z: [M+H]^+^ Calcd for C_16_H_15_^81^Br^35^ClO 338.9969. Found 338.9970.

##### 9,9-dimethyl-10-methylene-9,10-dihydroanthracene (2-28)

([See Spectra](#_1H_NMR_Spectrum_83))

The reaction was performed following the ***GP 1*** on a 10 mmol scale. The residue was purified by flash column chromatography on silica gel (PE) to afford the title compound as a white solid (1.46 g, 66%).

**TLC** (SiO_2_): R*_f_* = 0.40 (PE)

**^1^H NMR (400 MHz, CDCl_3_)** δ 7.68 (ddd, *J* = 7.5, 1.6, 0.5 Hz, 2H), 7.55 (ddd, *J* = 7.8, 1.4, 0.5 Hz, 2H), 7.34 – 7.29 (m, 2H), 7.28 – 7.22 (m, 2H), 5.65 (s, 2H), 1.60 (s, 6H).

**^13^C NMR (101 MHz, CDCl_3_)** δ 144.0, 142.7, 135.5, 127.9, 126.3, 124.5, 123.9, 109.5, 39.2, 30.0.

**HRMS (pos. APCI)** m/z: [M+H]^+^ Calcd for C_17_H_17_ 221.1325. Found 221.1320.

**Melting point:** 67-68 °C

##### 11-methylene-6,11-dihydrodibenzo[b,e]thiepine (2-29)

([See Spectra](#_1H_NMR_Spectrum_84))

The reaction was performed following the ***GP 1*** on a 10 mmol scale. The residue was purified by flash column chromatography on silica gel (PE) to afford the title compound as a yellowish solid (2.13 g, 95%).

**TLC** (SiO_2_): R*_f_* = 0.46 (PE)

**^1^H NMR (400 MHz, CDCl_3_)** δ 7.36 (ddd, *J* = 7.0, 2.4, 0.8 Hz, 1H), 7.30 – 7.18 (m, 4H), 7.11 – 7.02 (m, 3H), 5.60 (d, *J* = 1.7 Hz, 1H), 5.24 (d, *J* = 1.6 Hz, 1H), 4.17 (s, 2H).

**^13^C NMR (101 MHz, CDCl_3_)** δ 150.7, 142.4, 136.9, 136.4, 134.3, 130.9, 128.4, 128.1, 128.0, 127.8, 127.7, 126.1, 125.1, 119.5, 33.6.

**HRMS (pos. APCI)** m/z: [M+H]^+^ Calcd for C_15_H_13_S 225.0732. Found 225.0728.

**Melting point:** 56-57 °C

##### 2-(11-methylene-6,11-dihydrodibenzo[b,e]oxepin-2-yl)acetic acid (2-44)

([See Spectra](#_1H_NMR_Spectrum_85))

The reaction was performed following a slightly modified ***GP 1*** using 2.2 equiv. n-BuLi instead of 1.2 equiv. on a 10 mmol scale. The residue was purified by flash column chromatography on silica gel (PE:EtOAc = 2:1, acidified with 1 mol% HCO_2_H) to afford the title compound as a white solid (0.91 g, 34%).

**TLC** (SiO_2_): R*_f_* = 0.23 (PE:EtOAc = 2:1, acidified with 1 mol% HCO_2_H)

**^1^H NMR (400 MHz, CDCl_3_)** δ 7.37 – 7.25 (m, 5H), 7.08 (dd, *J* = 8.4, 2.3 Hz, 1H), 6.79 (d, *J* = 8.4 Hz, 1H), 5.69 (d, *J* = 1.1 Hz, 1H), 5.27 (d, *J* = 1.0 Hz, 1H), 5.15 (s, 2H), 3.57 (s, 2H).

**^13^C NMR (101 MHz, CDCl_3_)** δ 177.6, 155.4, 148.1, 143.9, 134.3, 131.1, 130.6, 129.2, 128.2, 127.6, 127.0, 126.0, 125.7, 120.1, 117.6, 70.9, 40.2.

**HRMS (neg. APCI)** m/z: [M-H]^-^ Calcd for C_17_H_13_O_3_ 265.0870. Found 265.0870.

**Melting point:** 164-165 °C

##### 1-chloro-2-(1-(4-fluorophenyl)vinyl)-4-iodobenzene (A-1)

([See Spectra](#_1H_NMR_Spectrum_86))

The reaction was performed following the ***GP 1*** on a 10 mmol scale. The residue was purified by flash column chromatography on silica gel (PE) to afford the title compound as a colorless oil (2.58 g, 72%).

**TLC** (SiO_2_): R*_f_* = 0.44 (PE)

**^1^H NMR (500 MHz, CDCl_3_)** δ 7.65 (d, *J* = 2.2 Hz, 1H), 7.59 (dd, *J* = 8.4, 2.2 Hz, 1H), 7.23 – 7.18 (m, 2H), 7.11 (d, *J* = 8.4 Hz, 1H), 7.00 – 6.95 (m, 2H), 5.75 (t, *J* = 0.6 Hz, 1H), 5.25 (d, *J* = 0.6 Hz, 1H).

**^19^F NMR (471 MHz, CDCl_3_)** δ -113.90 – -114.04 (m, 1F).

**^13^C NMR (126 MHz, CDCl_3_)** δ 162.6 (d, *J* = 247.8 Hz), 145.4, 142.6, 140.0, 138.0, 135.4 (d, *J* = 3.3 Hz), 133.5, 131.5, 128.2 (d, *J* = 8.2 Hz), 116.8 (d, *J* = 1.6 Hz), 115.4 (d, *J* = 21.8 Hz), 91.5.

**HRMS (pos. APCI)** m/z: [M+H]^+^ Calcd for C_14_H_10_^35^ClFI 358.9494. Found 358.9486.

##### 1-chloro-9-methylene-4-propoxy-9*H*-thioxanthene (A-2)

([See Spectra](#_1H_NMR_Spectrum_87))

The reaction was performed following the ***GP 1*** on a 10 mmol scale. The residue was purified by flash column chromatography on silica gel (PE) to afford the title compound as a greenish viscous oil (2.58 g, 72%).

**TLC** (SiO_2_): R*_f_* = 0.33 (PE)

**^1^H NMR (400 MHz, CDCl_3_)** δ 7.60 – 7.57 (m, 1H), 7.44 – 7.41 (m, 1H), 7.31 – 7.23 (m, 3H), 6.71 (d, *J* = 8.7 Hz, 1H), 5.81 (d, *J* = 0.8 Hz, 1H), 5.77 (d, *J* = 0.8 Hz, 1H), 4.02 (t, *J* = 6.4 Hz, 2H), 1.89 (qt, *J* = 7.4, 6.4 Hz, 2H), 1.11 (t, *J* = 7.4 Hz, 3H).

**^13^C NMR (101 MHz, CDCl_3_)** δ 153.3, 139.3, 137.2, 132.7, 131.5, 128.6, 127.6, 127.0, 126.3, 125.8, 124.6, 123.2, 121.1, 110.5, 71.0, 22.6, 10.7.

**HRMS (pos. APCI)** m/z: [M+H]^+^ Calcd for C_17_H_16_^35^ClOS 303.0605. Found 303.0606.

##### 9-methylene-2-(trifluoromethyl)-9*H*-thioxanthene (A-3)

([See Spectra](#_1H_NMR_Spectrum_88))

The reaction was performed following the ***GP 1*** on a 10 mmol scale. The residue was purified by flash column chromatography on silica gel (PE) to afford the title compound as a greenish solid (0.55 g, 20%).

**TLC** (SiO_2_): R*_f_* = 0.55 (PE)

**^1^H NMR (500 MHz, CDCl_3_)** δ 7.85 (dd, *J* = 1.8, 0.8 Hz, 1H), 7.65 – 7.59 (m, 1H), 7.47 (dd, *J* = 8.3, 1.9 Hz, 1H), 7.44 (dt, *J* = 8.3, 0.7 Hz, 1H), 7.37 – 7.32 (m, 1H), 7.31 – 7.25 (m, 2H), 5.63 (s, 1H), 5.59 (s, 1H).

**^19^F NMR (471 MHz, CDCl_3_)** δ -62.44 (s, 3F).

**^13^C NMR (126 MHz, CDCl_3_)** δ 141.5, 136.0, 135.0, 134.0, 130.2, 129.1 (q, *J* = 32.8 Hz), 128.2, 127.3, 126.4, 126.2, 126.0, 124.20 (q, *J* = 3.8 Hz), 124.17 (q, *J* = 272.0 Hz), 123.0 (q, *J* = 4.0 Hz), 115.4.

**HRMS (pos. APCI)** m/z: [M+H]^+^ Calcd for C_15_H_10_F_3_S 279.0450. Found 279.0452.

**Melting point:** 35-36 °C

##### 3-chloro-6-methyl-11-methylene-6,11-dihydrodibenzo[*c,f*][1,2]thiazepine 5,5-dioxide (A-4)

([See Spectra](#_1H_NMR_Spectrum_89))

The reaction was performed on a 10 mmol scale following a slightly modified ***GP 1*** with the reaction mixture being heated to reflux instead of room temperature. The residue was purified by flash column chromatography on silica gel (PE:EtOAc = 4:1) to afford the title compound as a white solid (0.25 g, 8%).

*Note: A significant amount of starting material still remained after 16 h. Further optimization was not conducted.*

**TLC** (SiO_2_): R*_f_* = 0.28 (PE:EtOAc = 4:1)

**^1^H NMR (400 MHz, CDCl_3_)** δ 7.91 (dd, *J* = 1.9, 0.6 Hz, 1H), 7.50 (dd, *J* = 8.3, 2.0 Hz, 1H), 7.48 – 7.43 (m, 2H), 7.42 – 7.37 (m, 1H), 7.35 (ddd, *J* = 7.9, 1.8, 0.5 Hz, 1H), 7.31 (ddd, *J* = 7.6, 7.0, 1.8 Hz, 1H), 5.65 (d, *J* = 0.8 Hz, 1H), 5.63 (d, *J* = 0.7 Hz, 1H), 3.26 (s, 3H).

**^13^C NMR (101 MHz, CDCl_3_)** δ 146.9, 140.1, 138.3, 137.6, 135.1, 134.9, 132.7, 131.3, 130.1, 129.6, 128.4, 127.3, 124.0, 39.6.

**HRMS (pos. APCI)** m/z: [M+H]^+^ Calcd for C_15_H_13_^35^ClNO_2_S 306.0350. Found 306.0349.

**Melting point:** 229-230 °C

##### diethyl 4-(dimethoxymethyl)-2,6-dimethyl-1,4-dihydropyridine-3,5-dicarboxylate (3-8)

([See Spectra](#_1H_NMR_Spectrum_77))

The reaction was performed following a modified literature procedure. To an oven dried round bottom flask equipped with a magnetic stirring bar was charged with the aldehyde (1.13 g, 4 mmol , 1.0 equiv.) and *p*-TsOH•H_2_O (15.2 mg, 0.08 mmol, 2 mol%). The flask was sealed with a rubber septum, evacuated and refilled with argon for three times before MeOH (5 mL, 0.8 M)and trimethyl orthoformate (1.75 mL, 16 mmol, 4.0 equiv.) was added sequentially. The resulting reaction mixture was then allowed to be heated at 60 °C overnight. The mixture was then cooled to room temperature, diluted with EtOAc and washed with brine. The aqueous phase was extracted two times with EtOAc before the organic phases were combined, dried with Na_2_SO_4_ and evaporated under reduced pressure. The residue was then subjected to flash column chromatography on silica gel (PE:EtOAc gradient from 75:25 to 25:75) to afford the title compound. Optional further purification can be conducted through recrystallization from PE/EtOAc (v(PE):v(EtOAc) = 8:1) at -20 °C.

**TLC** (SiO_2_): R*_f_* = 0.24 (PE:EtOAc = 1:1)

^1^H NMR (500 MHz, CDCl_3_) δ 5.75 (s, 1H), 4.35 (d, *J* = 5.2 Hz, 1H), 4.21 (dq, *J* = 10.8, 7.2 Hz, 2H), 4.16 (dq, *J* = 10.8, 7.1 Hz, 2H), 3.95 (d, *J* = 5.1 Hz, 1H), 3.29 (s, 6H), 2.28 (s, 6H), 1.29 (t, *J* = 7.1 Hz, 6H).

^13^C NMR (126 MHz, CDCl_3_) δ 168.3, 145.0, 107.9, 99.2, 59.7, 55.2, 36.9, 19.4, 14.4.

**HRMS (pos. ESI)** m/z: [M+Na]^+^ Calcd for C_16_H_25_O_6_NNa 350.1574. Found 350.1573.

**Melting point:** 83-85 °C

##### diethyl 4-(((*tert*-butoxycarbonyl)(methyl)amino)methyl)-2,6-dimethyl-1,4-dihydropyridine-3,5-dicarboxylate (3-9)

([See Spectra](#_1H_NMR_Spectrum_71))

The reaction was performed following the ***GP 2*** on a 15 mmol scale. The product was collected as a white solid (4.22 g, 71%) after flash column chromatography on silica gel (PE:EtOAc gradient from 95:5 to 50:50) and recrystallization from PE/EtOAc (v(PE):v(EtOAc) = 8:1) at -20 °C. The NMR spectra showed two set of picks with a ratio of 1.1:1. The formation of atropisomers was proposed to rationalize the observation.

**TLC** (SiO_2_): R*_f_* = 0.26 (PE:EtOAc = 2:1)

**^1^H NMR (500 MHz, CDCl_3_)** **(Major)** δ 7.39 (s, 1H), 4.23 – 4.11 (m, 5H), 3.04 (d, *J* = 7.2 Hz, 2H), 2.88 (s, 3H), 2.27 (s, 6H), 1.41 (s, 9H), 1.30 (t, *J* = 7.1 Hz, 6H).

**^1^H NMR (500 MHz, CDCl_3_)** **(Minor)** δ 6.19 (s, 1H), 4.23 – 4.10 (m, 5H), 3.12 (d, *J* = 5.8 Hz, 2H), 2.86 (s, 3H), 2.31 (s, 6H), 1.39 (s, 9H), 1.31 (t, *J* = 7.1 Hz, 6H).

**^13^C NMR (126 MHz, CDCl_3_) (Major)** δ 167.64, 156.14, 147.07, 100.10, 78.96, 59.62, 50.93, 34.32, 31.36, 28.43, 19.19, 14.50.

**^13^C NMR (126 MHz, CDCl_3_)** **(Minor)** δ 167.50, 155.88, 146.08, 101.10, 78.79, 59.83, 52.92, 34.81, 32.80, 28.35, 19.47, 14.46.

**HRMS (pos. ESI)** m/z: [M+Na]^+^ Calcd for C_20_H_32_O_6_N_2_Na 419.2153. Found 419.2159.

**Melting point:** 131-132 °C

##### diethyl 4-((allyl(ethoxycarbonyl)amino)methyl)-2,6-dimethyl-1,4-dihydropyridine-3,5-dicarboxylate (3-10)

([See Spectra](#_1H_NMR_Spectrum_72))

The reaction was performed following the ***GP 2*** on a 6 mmol scale. The product was collected as a white solid (1.52 g, 67%) after flash column chromatography on silica gel (PE:EtOAc gradient from 80:20 to 40:60) and recrystallization from PE/EtOAc (v(PE):v(EtOAc) = 8:1) at -20 °C. The NMR spectra showed two set of picks with a ratio of 1:1. The formation of atropisomers was proposed to rationalize the observation.

**TLC** (SiO_2_): R*_f_* = 0.22 (PE:EtOAc = 1:1)

**^1^H NMR (500 MHz, CDCl_3_)** δ 7.19 (s, 0.50H) (Set of isomer peak 1), 6.24 (s, 0.47H) (Set of isomer peak 1), 5.82 – 5.64 (m, 1H), 5.16 – 5.02 (m, 2H), 4.26 – 4.10 (m, 5H), 4.05 (q, *J* = 7.1 Hz, 2H) (Set of isomer peak 2), 4.05 (q, *J* = 7.1 Hz, 2H) (Set of isomer peak 2), 3.88 (d, *J* = 4.8 Hz, 2H) (Set of isomer peak 3), 3.86 (d, *J* = 5.1 Hz, 2H) (Set of isomer peak 3), 3.20 – 3.02 (m, 2H), 2.29 (s, 6H), 1.30 (t, *J* = 7.1 Hz, 6H), 1.22 (t, *J* = 7.1 Hz, 3H) (Set of isomer peak 4), 1.20 (t, *J* = 7.1 Hz, 3H) (Set of isomer peak 4).

**^13^C NMR (126 MHz, CDCl_3_)** δ 167.73 (Set of isomer peak 1), 167.53 (Set of isomer peak 1), 156.72 (Set of isomer peak 2), 156.66 (Set of isomer peak 2), 146.91 (Set of isomer peak 3), 146.13 (Set of isomer peak 3), 133.74 (Set of isomer peak 4), 133.68 (Set of isomer peak 4), 116.29 (Set of isomer peak 5), 116.22 (Set of isomer peak 5), 100.87 (Set of isomer peak 6), 100.08 (Set of isomer peak 6), 61.21 (Set of isomer peak 7), 61.13 (Set of isomer peak 7), 59.81 (Set of isomer peak 8), 59.63, (Set of isomer peak 8) 49.92 (Set of isomer peak 9), 49.19 (Set of isomer peak 9), 48.66 (Set of isomer peak 10), 48.62 (Set of isomer peak 10), 33.05 (Set of isomer peak 11), 31.55 (Set of isomer peak 11), 19.31 (Set of isomer peak 12), 19.08 (Set of isomer peak 12), 14.73 (Set of isomer peak 13), 14.64 (Set of isomer peak 13), 14.48.

**HRMS (pos. ESI)** m/z: [M+Na]^+^ Calcd for C_20_H_30_O_6_N_2_Na 417.1996. Found 417.1988.

**Melting point:** 125-126 °C

##### diethyl 4-((1*H*-indol-1-yl)methyl)-2,6-dimethyl-1,4-dihydropyridine-3,5-dicarboxylate (3-11)

([See Spectra](#_1H_NMR_Spectrum_73))

The reaction was performed following the ***GP 2*** on a 11 mmol scale. The product was collected as a white solid (1.74 g, 41%) after flash column chromatography on silica gel (PE:EtOAc gradient from 80:20 to 40:60) and recrystallization from PE/EtOAc (v(PE):v(EtOAc) = 8:1) at -20 °C. The product was stored at -20 °C.

**TLC** (SiO_2_): R*_f_* = 0.21 (PE:EtOAc = 4:1)

**^1^H NMR (500 MHz, CDCl_3_)** δ 7.53 (dt, *J* = 7.9, 1.0 Hz, 1H), 7.35 (dq, *J* = 8.3, 0.9 Hz, 1H), 7.13 (ddd, *J* = 8.3, 7.0, 1.2 Hz, 1H), 7.01 (ddd, *J* = 7.9, 7.0, 1.0 Hz, 1H), 6.82 (d, *J* = 3.1 Hz, 1H), 6.35 (dd, *J* = 3.1, 0.9 Hz, 1H), 5.39 (s, 1H), 4.38 (t, *J* = 4.9 Hz, 1H), 4.10 (dq, *J* = 10.8, 7.1 Hz, 3H), 4.05 (d, *J* = 4.9 Hz, 2H), 3.96 (dq, *J* = 10.8, 7.2 Hz, 2H), 2.07 (s, 6H), 1.15 (t, *J* = 7.1 Hz, 6H).

**^13^C NMR (126 MHz, CDCl_3_)** δ 167.4, 146.6, 137.1, 129.3, 128.5, 121.1, 120.5, 118.9, 109.9, 100.17, 100.14, 60.0, 50.7, 35.4, 19.3, 14.2.

**HRMS (pos. ESI)** m/z: [M+Na]^+^ Calcd for C_22_H_26_O_4_N_2_Na 405.1785. Found 405.1779.

**Melting point:** 109-111 °C

##### diethyl 4-benzhydryl-2,6-dimethyl-1,4-dihydropyridine-3,5-dicarboxylate (3-16)

([See Spectra](#_1H_NMR_Spectrum_74))

The reaction was performed following the ***GP 2*** on a 25 mmol scale. The product was collected as a white solid (1.73 g, 16%) after flash column chromatography on silica gel (PE:EtOAc gradient from 93:7 to 45:55) and recrystallization from PE/EtOAc (v(PE):v(EtOAc) = 8:1) at -20 °C.

**TLC** (SiO_2_): R*_f_* = 0.22 (PE:EtOAc = 3:1)

**^1^H NMR (500 MHz, CDCl_3_)** δ 7.34 – 7.27 (m, 4H), 7.22 – 7.17 (m, 4H), 7.15 – 7.09 (m, 2H), 5.82 (s, 1fH), 4.91 (d, *J* = 8.6 Hz, 1H), 3.93 (dq, *J* = 10.7, 7.1 Hz, 2H), 3.76 (dq, *J* = 10.7, 7.1 Hz, 2H), 3.69 (d, *J* = 8.5 Hz, 1H), 2.20 (s, 6H), 1.17 (t, *J* = 7.1 Hz, 6H).

**^13^C NMR (126 MHz, CDCl_3_)** δ 167.9, 144.3, 141.5, 129.6, 127.6, 126.2, 102.4, 59.6, 57.7, 39.4, 19.2, 14.2.

**HRMS (pos. ESI)** m/z: [M+Na]^+^ Calcd for C_26_H_29_O_4_NNa 442.1989. Found 442.1983.

**Melting point:** 153-154 °C

##### diethyl 4-(hydroxymethyl)-2,6-dimethyl-1,4-dihydropyridine-3,5-dicarboxylate (B-1)

([See Spectra](#_1H_NMR_Spectrum_75))

The reaction was performed following a modified literature procedure. To an oven dried round bottom flask was charged with a magnetic stirring bar. The flask was sealed with a rubber septum, evacuated and refilled with argon for three times before THF (2 mL) and TBS protected alcohol (1.59 g, 4 mmol, 1.0 equiv.) was added sequentially. TBAF (1 M in THF, 12.8 mL , 12.8 mmol, 3.2 equiv.) was subjected to the solution and the reaction mixture was allowed to be heated at 50 °C for 3-4 hours until the starting material was completely consumed. The mixture was then cooled to room temperature and evaporated under reduced pressure. The residue was then subjected to flash column chromatography on silica gel (PE:EtOAc gradient from 75:25 to 25:75) to afford the title compound. Optional further purification can be conducted through recrystallization from PE/EtOAc (v(PE):v(EtOAc) = 8:1) at -20 °C.

*Note: Some* *lactonized byproduct was also observed under the current condition. The separation was easy.*

**TLC** (SiO_2_): R*_f_* = 0.18 (PE:EtOAc = 1:1)

**^1^H NMR (700 MHz, CDCl_3_)** δ 5.96 (s, 1H), 4.22 (dq, *J* = 11.0, 7.1 Hz, 2H), 4.19 (dq, *J* = 10.8, 7.1 Hz, 2H), 4.01 (t, *J* = 5.6 Hz, 1H), 3.45 (d, *J* = 5.6 Hz, 2H), 2.40 (br, s, 1H), 2.29 (s, 6H), 1.30 (t, *J* = 7.1 Hz, 6H).

**^13^C NMR (176 MHz, CDCl_3_)** δ 168.2, 146.2, 100.2, 67.0, 60.2, 36.7, 19.6, 14.5.

**HRMS (neg. ESI)** m/z: [M-H]^-^ Calcd for C_14_H_20_O_5_N 282.1347. Found 282.1349.

**Melting point:** 134-136 °C

##### diethyl 4-((benzoyloxy)methyl)-2,6-dimethyl-1,4-dihydropyridine-3,5-dicarboxylate (B-2)

([See Spectra](#_1H_NMR_Spectrum_76))

The reaction was performed following the ***GP 2*** on a 11 mmol scale. The product was collected as a yellow solid (1.52 g, 36%) after flash column chromatography on silica gel (PE:EtOAc gradient from 92:8 to 50:50) and recrystallization from PE/EtOAc (v(PE):v(EtOAc) = 8:1) at -20 °C.

**TLC** (SiO_2_): R*_f_* = 0.20 (PE:EtOAc = 2:1)

**^1^H NMR (700 MHz, CDCl_3_)** δ 7.98 – 7.95 (m, 2H), 7.54 – 7.50 (m, 1H), 7.42 – 7.38 (m, 2H), 5.80 (s, 1H), 4.37 (t, *J* = 5.1 Hz, 1H), 4.19 (d, *J* = 5.1 Hz, 2H), 4.16 (qd, *J* = 7.1, 2.6 Hz, 4H), 2.30 (s, 6H), 1.27 (t, *J* = 7.1 Hz, 6H).

**^13^C NMR (176 MHz, CDCl_3_)** δ 167.5, 166.8, 146.4, 132.8, 130.7, 129.6, 128.3, 99.7, 67.6, 60.0, 33.9, 19.5, 14.4.

**HRMS (pos. ESI)** m/z: [M+Na]^+^ Calcd for C_21_H_25_O_6_NNa 410.1574. Found 410.1577.

**Melting point:** 102-103 °C

### Products

#### Allylic products

##### (6-methylhept-1-ene-4,4-diyl)dibenzene (4-1) ([See Spectra](#_1H_NMR_Spectrum))

The reaction was performed following the ***GP 3***. The crude mixture was first roughly purified though normal phase column chromatography on silica gel (Pure PE) to get rid of the majority of polar side products. The residue was then carefully purified by a reverse phase column chromatography on C18 to afford the title compound as a colorless oil (41.5 mg, 79%).

**TLC** (SiO_2_): R*_f_* = 0.50 (PE)

**^1^H NMR** (400 MHz, CDCl_3_) δ 7.27 – 7.20 (m, 4H), 7.19 – 7.11 (m, 6H), 5.34 (ddt, *J* = 17.1, 10.1, 7.0 Hz, 1H), 5.01 – 4.88 (m, 2H), 2.93 (dt, *J* = 7.0, 1.4 Hz, 2H), 2.02 (d, *J* = 5.1 Hz, 2H), 1.58 – 1.47 (m, 1H), 0.61 (d, *J* = 6.7 Hz, 6H).

**^13^C NMR** (101 MHz, CDCl_3_) δ 148.8, 135.2, 128.2, 127.8, 125.7, 117.5, 49.6, 46.2, 43.0, 24.9, 24.2.

**HRMS (pos. APCI)** m/z: [M+NO]^+^ Calcd for C_20_H_24_NO 294.1852. Found 294.1848.

##### (1-cyclobutylpent-4-ene-2,2-diyl)dibenzene (4-2) ([See Spectra](#_1H_NMR_Spectrum_1))

The reaction was performed following the ***GP 3***. The crude mixture was first roughly purified though normal phase column chromatography on silica gel (Pure PE) to get rid of the majority of polar side products. The residue was then carefully purified by a reverse phase column chromatography on C18 to afford the title compound as a colorless oil (14.0 mg, 25%).

**TLC** (SiO_2_): R*_f_* = 0.48 (PE)

^1^**H NMR (400 MHz, CDCl_3_)** δ 7.26 – 7.20 (m, 4H), 7.17 – 7.11 (m, 6H), 5.34 (ddt, *J* = 17.2, 10.2, 7.0 Hz, 1H), 4.99 – 4.87 (m, 2H), 2.86 (dt, *J* = 7.0, 1.3 Hz, 2H), 2.19 (d, *J* = 6.8 Hz, 2H), 2.17 – 2.06 (m, 1H), 1.69 – 1.52 (m, 4H), 1.49 – 1.35 (m, 2H).

**^13^C NMR** **(101 MHz, CDCl_3_)** δ 148.6, 135.2, 128.1, 125.7, 117.4, 49.4, 45.0, 42.6, 32.7, 30.1, 19.3.

**HRMS (pos. APCI)** m/z: [M+NH_4_]^+^ Calcd for C_21_H_28_N 294.2216. Found 294.2218.

##### benzyl 4-(2,2-bis(4-methoxyphenyl)pent-4-en-1-yl)piperidine-1-carboxylate (4-3)

([See Spectra](#_1H_NMR_Spectrum_2))

The reaction was performed following the ***GP 3***. The crude mixture was first roughly purified though normal phase column chromatography on silica gel (PE:EtOAc = 1:1) to get rid of the majority of side products. The residue was then carefully purified by a reverse phase column chromatography on C18 to afford the title compound as a colorless oil (73.1 mg, 73%).

**TLC** (SiO_2_): R*_f_* = 0.23 (PE:EtOAc = 1:1)

**^1^H NMR (500 MHz, CDCl_3_)** δ 7.36 – 7.24 (m, 5H), 7.09 – 7.03 (m, 4H), 6.81 – 6.74 (m, 4H), 5.33 (ddt, *J* = 17.1, 10.1, 6.9 Hz, 1H), 5.07 (s, 2H), 4.99 – 4.89 (m, 2H), 4.06 – 3.82 (br, s, 2H), 3.77 (s, 6H), 2.85 (d, *J* = 6.9 Hz, 2H), 2.57 (ddd, *J* = 13.3, 12.0, 3.0 Hz, 2H), 2.03 – 1.89 (br, s, 2H), 1.41 – 1.30 (m, 1H), 1.24 – 0.80 (br, 4H).

**^13^C NMR (126 MHz, CDCl_3_)** δ 157.5, 155.2, 140.6, 137.0, 135.2, 128.9, 128.5, 127.9, 127.8, 117.6, 113.1, 66.9, 55.2, 48.1, 44.3, 44.2, 43.4, 33.8, 32.0.

**HRMS (pos. APCI)** m/z: [M+H]^+^ Calcd for C_32_H_38_O_4_N 500.2795. Found 500.2795.

##### 5-(2-methyl-4,4-diphenylhept-6-en-1-yl)benzo[d][1,3]dioxole (4-4)

([See spectra](#_1H_NMR_Spectrum_3)) ([See gram scale reaction](#_Allylation-Std._Cond._1))

The reaction was performed following the ***GP 3***. The crude mixture was first roughly purified though column chromatography on silica gel (PE:EtOAc = 100:1) to get rid of the majority of polar side products. The residue was then carefully purified by a reverse phase column chromatography on C18 to afford the title compound as a yellowish oil (43.4 mg, 57%).

**TLC** (SiO_2_): R*_f_* = 0.22 (PE)

**^1^H NMR (400 MHz, CDCl_3_)** δ 7.29 – 7.21 (m, 4H), 7.20 – 7.12 (m, 6H), 6.63 (dd, *J* = 7.8, 0.5 Hz, 1H), 6.39 – 6.31 (m, 2H), 5.89 – 5.84 (m, 2H), 5.29 (ddt, *J* = 17.2, 10.1, 6.9 Hz, 1H), 4.98 – 4.85 (m, 2H), 3.00 – 2.84 (m, 2H), 2.19 – 2.10 (m, 2H), 2.08 – 1.95 (m, 2H), 1.65 – 1.49 (m, 1H), 0.56 (d, *J* = 6.7 Hz, 3H).

**^13^C NMR (101 MHz, CDCl_3_)** δ 148.7, 148.5, 147.4, 145.5, 135.5, 135.1, 128.35, 128.25, 127.9, 127.8, 125.9, 125.8, 122.1, 117.6, 109.6, 107.8, 100.7, 49.8, 45.2, 44.3, 43.0, 31.5, 21.3.

**HRMS (pos. APCI)** m/z: [M+H]^+^ Calcd for C_27_H_29_O_2_ 385.2162. Found 385.2158.

##### (6,10-dimethylundeca-1,9-diene-4,4-diyl)dibenzene (4-5)

([See spectra](#_1H_NMR_Spectrum_4))

The reaction was performed following a slightly modified ***GP 3*** using (η^3^-cinnamyl)PdCp instead of Pd(OAc)_2_. The crude mixture was first roughly purified though column chromatography on silica gel (PE) to get rid of the majority of polar side products. The residue was then carefully purified by a reverse phase column chromatography on C18 to afford the title compound as a colorless oil (23.2 mg, 35%).

**TLC** (SiO_2_): R*_f_* = 0.64 (PE)

**^1^H NMR (400 MHz, CDCl_3_)** δ 7.26 – 7.20 (m, 4H), 7.18 – 7.12 (m, 6H), 5.35 (ddt, *J* = 17.1, 10.1, 6.9 Hz, 1H), 5.00 – 4.84 (m, 3H), 2.97 – 2.86 (m, 2H), 2.10 (dd, *J* = 13.7, 3.9 Hz, 1H), 1.95 (dd, *J* = 13.7, 5.8 Hz, 1H), 1.86 – 1.66 (m, 2H), 1.64 (q, *J* = 1.3 Hz, 3H), 1.52 (d, *J* = 1.2 Hz, 3H), 1.44 – 1.32 (m, 1H), 1.07 – 0.92 (m, 2H), 0.56 (d, *J* = 6.7 Hz, 3H).

**^13^C NMR (101 MHz, CDCl_3_)** δ 148.9, 148.8, 135.2, 131.0, 128.32, 128.28, 127.78, 127.77, 125.71, 125.69, 125.0, 117.5, 49.7, 44.9, 42.8, 39.2, 28.4, 25.7, 25.5, 21.4, 17.7.

**HRMS (pos. APCI)** m/z: [M+NH_4_]^+^ Calcd for C_25_H_36_N 350.2842. Found 350.2850.

##### 4,4'-(1-(4-(4-methylpent-3-en-1-yl)cyclohex-3-en-1-yl)pent-4-ene-2,2-diyl)bis(methoxybenzene) (4-6)

([See spectra](#_1H_NMR_Spectrum_5))

The reaction was performed following the ***GP 3***. The crude mixture was first roughly purified though column chromatography on silica gel (PE:EtOAc = 40:1) to get rid of the majority of polar side products. The residue was then carefully purified by a reverse phase column chromatography on C18 to afford the title compound as a colorless oil (53.8 mg, 60%). The NMR spectra showed two set of picks with a ratio of 2.3:1 while the GC-HRMS gave only one absorption pick. The formation of atropisomers was proposed to rationalize the observed phenomena.

**TLC** (SiO_2_): R*_f_* = 0.28 (PE:EtOAc = 40:1)

**^1^H NMR (400 MHz, CDCl_3_)** δ 7.12 – 7.04 (m, 4H), 6.82 – 6.73 (m, 4H), 5.44 – 5.29 (m, 1H), 5.29 – 5.24 (m, 1H) (Minor), 5.22 – 5.17 (m, 1H) (Major), 5.11 – 4.98 (m, 1H), 5.02 – 4.87 (m, 2H), 3.80 – 3.74 (m, 6H), 2.93 – 2.79 (m, 2H), 2.07 – 1.72 (m, 8H), 1.66 (q, *J* = 1.3 Hz, 4H), 1.58 – 1.55 (m, 3H), 1.53 – 1.38 (m, 2H), 1.22 – 1.12 (m, 1H), 1.11 – 0.92 (m, 1H).

**^13^C NMR (101 MHz, CDCl_3_)** **(Major)** δ 157.47, 141.11, 141.01, 137.43, 135.49, 131.28, 129.07, 124.59, 120.60, 117.39, 113.09, 55.22, 48.33, 44.54, 43.47, 37.63, 34.00, 31.09, 29.71, 28.73, 26.55, 25.76, 17.74.

**^13^C NMR (101 MHz, CDCl_3_) (Minor)** δ 157.54, 157.50, 141.05, 137.62, 135.51, 131.24, 129.10, 124.56, 120.33, 117.39, 113.12, 55.22, 48.36, 44.64, 43.50, 37.91, 37.01, 30.64, 30.05, 28.73, 26.51, 25.44, 17.74.

**HRMS (pos. APCI)** m/z: [M+H]^+^ Calcd for C_31_H_41_O_2_ 445.3101. Found 445.3098.

##### ((3,3-bis(4-methoxyphenyl)hex-5-en-1-yl)oxy)(tert-butyl)dimethylsilane (4-7)

([See spectra](#_1H_NMR_Spectrum_6))

The reaction was performed following a slightly modified ***GP 3*** using the combination of 1.2 equiv. of DHP derivative, 1.5 equiv. of 4,4'-(ethene-1,1-diyl)bis(methoxybenzene), 1.0 mol% of [Ir(ppy)_2_dtbbpy]PF_6_, 10 mol% of Pd(OAc)_2_ and 12 mol% of Xantphos. The crude mixture was first roughly purified though column chromatography on silica gel (PE:EtOAc = 40:1) to get rid of the majority of polar side products. The residue was then carefully purified by a reverse phase column chromatography on C18 to afford the title compound as a colorless oil (44.4 mg, 52%).

**TLC** (SiO_2_): R*_f_* = 0.38 (PE:EtOAc = 40:1)

**^1^H NMR (500 MHz, CDCl_3_)** δ 7.11 – 7.04 (m, 4H), 6.82 – 6.75 (m, 4H), 5.40 (ddt, *J* = 17.2, 10.2, 7.0 Hz, 1H), 5.03 – 4.92 (m, 2H), 3.40 – 3.33 (m, 2H), 2.80 (dt, *J* = 7.0, 1.3 Hz, 2H), 2.32 – 2.26 (m, 2H), 0.83 (s, 9H), -0.06 (s, 6H).

**^13^C NMR (126 MHz, CDCl_3_)** δ 157.5, 140.4, 134.9, 128.7, 117.5, 113.2, 60.1, 55.2, 46.9, 43.3, 40.5, 26.1, 18.4, -5.2.

**HRMS (pos. ESI)** m/z: [M+Na]^+^ Calcd for C_26_H_38_NaO_3_Si 449.2482. Found 449.2485.

##### (1,1-dimethoxyhex-5-ene-3,3-diyl)dibenzene (4-8)

([See spectra](#_1H_NMR_Spectrum_7))

The reaction was performed following a slightly modified ***GP 3*** using (η^3^-cinnamyl)PdCp instead of Pd(OAc)_2_. The crude mixture was first roughly purified though column chromatography on silica gel (PE:EtOAc = 40:1) to get rid of the majority of polar side products. The residue was then carefully purified by a reverse phase column chromatography on C18 to afford the title compound as a colorless oil (34.9 mg, 59%).

**TLC** (SiO_2_): R*_f_* = 0.26 (PE:EtOAc = 40:1)

**^1^H NMR (400 MHz, CDCl_3_)** δ 7.28 – 7.23 (m, 4H), 7.19 – 7.14 (m, 6H), 5.38 (ddt, *J* = 17.2, 10.2, 7.0 Hz, 1H), 5.00 – 4.89 (m, 2H), 3.91 (t, *J* = 4.9 Hz, 1H), 3.13 (s, 6H), 2.96 (dt, *J* = 7.0, 1.2 Hz, 2H), 2.42 (d, *J* = 4.9 Hz, 2H).

**^13^C NMR (101 MHz, CDCl_3_)** δ 148.1, 134.8, 128.0, 127.9, 125.9, 118.2, 103.8, 53.1, 48.1, 42.9, 40.7.

**HRMS (pos. APCI)** m/z: [M+NH_4_]^+^ Calcd for C_20_H_28_NO_2_ 314.2115. Found 314.2112.

##### *tert*-butyl (3,3-diphenylhex-5-en-1-yl)(methyl)carbamate (4-9)

([See spectra](#_1H_NMR_Spectrum_8)) ([See gram scale reaction](#_Allylation-Std._Cond._2))

The reaction was performed following the ***GP 4***. The crude mixture was first roughly purified though column chromatography on silica gel (PE:EtOAc = 40:1) to get rid of the majority of polar side products. The residue was then carefully purified by a reverse phase column chromatography on C18 to afford the title compound as a white solid (61.0 mg, 87%).

**TLC** (SiO_2_): R*_f_* = 0.24 (PE:EtOAc = 40:1)

**^1^H NMR (400 MHz, CDCl_3_)** δ 7.29 – 7.22 (m, 4H), 7.20 – 7.13 (m, 6H), 5.39 (ddt, *J* = 17.1, 10.1, 7.0 Hz, 1H), 5.04 (ddt, *J* = 17.1, 2.5, 1.3 Hz, 1H), 4.98 (ddd, *J* = 10.3, 2.2, 1.1 Hz, 1H), 2.99 – 2.82 (m, 4H), 2.69 (s, 3H), 2.33 – 2.24 (m, 2H), 1.40 (s, 9H).

**^13^C NMR (101 MHz, CDCl_3_)** δ 155.7, 147.6, 134.4, 128.1, 127.7, 126.0, 117.8, 79.3, 47.9, 45.2, 42.6, 34.8, 34.1, 28.6.

**HRMS (pos. ESI)** m/z: [M+Na]^+^ Calcd for C_24_H_31_O_2_NNa 388.2247. Found 388.2247.

**Melting point**: 76-78 °C.

##### ethyl allyl(3,3-diphenylhex-5-en-1-yl)carbamate (4-10)

([See spectra](#_1H_NMR_Spectrum_9))

The reaction was performed following the ***GP 4***. The crude mixture was first roughly purified though column chromatography on silica gel (PE:EtOAc = 10:1) to get rid of the majority of side products. The residue was then carefully purified by a reverse phase column chromatography on C18 to afford the title compound as a colorless oil (56.2 mg, 77%).

**TLC** (SiO_2_): R*_f_* = 0.28 (PE:EtOAc = 10:1)

**^1^H NMR (400 MHz, CDCl_3_)** δ 7.29 – 7.22 (m, 4H), 7.21 – 7.12 (m, 6H), 5.72 – 5.56 (m, 1H), 5.46 – 5.30 (m, 1H), 5.09 – 4.95 (m, 3H), 4.94 – 4.81 (m, 1H), 4.08 (q, *J* = 7.2 Hz, 2H), 3.68 (d, *J* = 33.2 Hz, 2H), 3.01 – 2.76 (m, 4H), 2.40 – 2.21 (m, 2H), 1.20 (t, *J* = 7.1 Hz, 3H).

**^13^C NMR (101 MHz, CDCl_3_)** δ 156.2, 147.6, 134.4, 133.9, 128.0, 127.7, 126.0, 117.8, 116.9 (d, *J* = 51.7 Hz), 61.2, 49.7, 42.7 (d, *J* = 82.1 Hz), 42.5, 35.2, 14.7.

**HRMS (pos. APCI)** m/z: [M+H]^+^ Calcd for C_24_H_30_NO_2_ 364.2271. Found 364.2265.

##### 1-(3,3-diphenylhex-5-en-1-yl)-1H-indole (4-11)

([See spectra](#_1H_NMR_Spectrum_10)) ([See crystal structure](#_Allylic_product_4-11))

The reaction was performed following the ***GP 4***. The crude mixture was first roughly purified though column chromatography on silica gel (PE) to get rid of the majority of polar side products. The residue was then carefully purified by a reverse phase column chromatography on C18 to afford the title compound as a white solid (52.9 mg, 75%).

**TLC** (SiO_2_): R*_f_* = 0.26 (PE)

**^1^H NMR (400 MHz, CDCl_3_)** δ 7.59 (ddd, *J* = 7.7, 1.3, 0.8 Hz, 1H), 7.36 – 7.26 (m, 4H), 7.25 – 7.19 (m, 6H), 7.11 (ddd, *J* = 8.2, 7.0, 1.3 Hz, 1H), 7.05 (ddd, *J* = 7.7, 7.0, 1.2 Hz, 1H), 6.97 (dq, *J* = 8.1, 0.9 Hz, 1H), 6.90 (d, *J* = 3.1 Hz, 1H), 6.44 (dd, *J* = 3.1, 0.9 Hz, 1H), 5.43 (ddt, *J* = 17.0, 10.1, 7.0 Hz, 1H), 5.19 (ddt, *J* = 17.1, 2.2, 1.3 Hz, 1H), 5.09 (ddt, *J* = 10.1, 2.1, 1.0 Hz, 1H), 3.88 – 3.79 (m, 2H), 3.01 (dt, *J* = 7.0, 1.3 Hz, 2H), 2.62 – 2.53 (m, 2H).

**^13^C NMR (101 MHz, CDCl_3_)** δ 147.4, 135.9, 134.5, 128.7, 128.3, 127.7, 127.6, 126.4, 121.4, 121.0, 119.3, 118.0, 109.3, 101.3, 48.3, 42.5, 42.4, 37.9.

**HRMS (pos. ESI)** m/z: [M+H]^+^ Calcd for C_26_H_26_N 352.2060. Found 352.2063.

**Melting point**: 131-133 °C.

##### 9-(3,3-bis(4-methoxyphenyl)hex-5-en-1-yl)-9*H*-carbazole (4-12)

([See spectra](#_1H_NMR_Spectrum_11))

The reaction was performed following the ***GP 3***. The crude mixture was first roughly purified though column chromatography on silica gel (PE:EtOAc = 40:1) to get rid of the majority of polar side products. The residue was then carefully purified by a reverse phase column chromatography on C18 to afford the title compound as a white solid (52.6 mg, 57%).

**TLC** (SiO_2_): R*_f_* = 0.26 (PE:EtOAc = 40:1)

**^1^H NMR (500 MHz, CDCl_3_)** δ 8.06 (dt, *J* = 7.6, 1.0 Hz, 2H), 7.36 (ddd, *J* = 8.2, 7.2, 1.2 Hz, 2H), 7.18 (td, *J* = 7.5, 0.9 Hz, 2H), 7.14 – 7.09 (m, 4H), 7.03 – 6.97 (m, 2H), 6.87 – 6.80 (m, 4H), 5.49 (ddt, *J* = 16.9, 9.9, 6.8 Hz, 1H), 5.36 – 5.29 (m, 1H), 5.20 – 5.14 (m, 1H), 4.01 – 3.94 (m, 2H), 3.78 (s, 6H), 3.02 – 2.97 (m, 2H), 2.49 – 2.42 (m, 2H).

**^13^C NMR (126 MHz, CDCl_3_)** δ 157.9, 140.1, 139.8, 135.0, 128.6, 125.6, 122.9, 120.4, 118.8, 117.8, 113.6, 108.5, 55.3, 47.0, 42.5, 38.7, 35.8.

**HRMS (pos. APCI)** m/z: [M+H]^+^ Calcd for C_32_H_32_O_2_N 462.2428. Found 462.2435.

**Melting point**: 175-177 °C.

##### hex-5-ene-1,3,3-triyltribenzene (4-13)

([See spectra](#_1H_NMR_Spectrum_12))

The reaction was performed following the ***GP 4***. The crude mixture was first roughly purified though column chromatography on silica gel (PE) to get rid of the majority of polar side products. The residue was then carefully purified by a reverse phase column chromatography on C18 to afford the title compound as a white solid (23.4 mg, 37%).

**TLC** (SiO_2_): R*_f_* = 0.38 (PE)

**^1^H NMR (400 MHz, CDCl_3_)** δ 7.30 – 7.24 (m, 4H), 7.24 – 7.12 (m, 9H), 7.08 – 7.03 (m, 2H), 5.40 (ddt, *J* = 17.1, 10.1, 7.0 Hz, 1H), 5.15 – 5.05 (m, 1H), 5.00 (ddt, *J* = 10.1, 2.2, 1.0 Hz, 1H), 2.98 (dt, *J* = 7.1, 1.2 Hz, 2H), 2.41 – 2.34 (m, 2H), 2.30 – 2.22 (m, 2H).

**^13^C NMR (101 MHz, CDCl_3_)** δ 148.2, 143.0, 134.8, 128.5, 128.4, 128.0, 128.0, 125.9, 125.8, 117.5, 49.2, 42.3, 40.1, 30.6.

**HRMS (pos. APCI)** m/z: [M+H]^+^ Calcd for C_24_H_25_ 313.1951. Found 313.1958.

**Melting point**: 96-97 °C.

##### 1,3,3-triphenylhex-5-en-1-one (4-14)

([See spectra](#_1H_NMR_Spectrum_13))

The reaction was performed following the ***GP 4***. The crude mixture was first roughly purified though column chromatography on silica gel (PE:EtOAc = 40:1) to get rid of the majority of polar side products. The residue was then carefully purified by a reverse phase column chromatography on C18 to afford the title compound as a white solid (15.0 mg, 23%).

**TLC** (SiO_2_): R*_f_* = 0.30 (PE:EtOAc = 40:1)

**^1^H NMR (400 MHz, CDCl_3_)** δ 7.78 – 7.70 (m, 2H), 7.49 – 7.40 (m, 1H), 7.35 – 7.30 (m, 2H), 7.23 (d, *J* = 4.3 Hz, 8H), 7.17 – 7.10 (m, 2H), 5.43 (ddt, *J* = 17.2, 10.1, 7.2 Hz, 1H), 5.07 (ddt, *J* = 17.1, 2.5, 1.3 Hz, 1H), 4.98 (ddt, *J* = 10.1, 2.1, 1.0 Hz, 1H), 3.82 (s, 2H), 3.28 (dt, *J* = 7.2, 1.2 Hz, 2H).

**^13^C NMR (101 MHz, CDCl_3_)** δ 198.6, 147.6, 138.5, 134.8, 132.5, 128.3, 128.0, 127.9, 127.8, 126.1, 118.7, 48.8, 44.5, 42.6.

**HRMS (pos. APCI)** m/z: [M+H]^+^ Calcd for C_24_H_23_O 327.1743. Found 327.1744.

**Melting point**: 107-109 °C.

##### hept-6-ene-1,4,4-triyltribenzene (4-15)

([See spectra](#_1H_NMR_Spectrum_14))

The reaction was performed following the ***GP 3***. The crude mixture was first roughly purified though column chromatography on silica gel (PE) to get rid of the majority of polar side products. The residue was then carefully purified by a reverse phase column chromatography on C18 to afford the title compound as a colorless oil (10.9 mg, 17%).

**TLC** (SiO_2_): R*_f_* = 0.44 (PE)

**^1^H NMR (400 MHz, CDCl_3_)** δ 7.26 – 7.19 (m, 6H), 7.17 – 7.10 (m, 7H), 7.07 – 7.03 (m, 2H), 5.31 (ddt, *J* = 17.1, 10.1, 7.0 Hz, 1H), 4.99 – 4.85 (m, 2H), 2.86 (dt, *J* = 7.1, 1.2 Hz, 2H), 2.52 (t, *J* = 7.6 Hz, 2H), 2.14 – 2.06 (m, 2H), 1.39 – 1.29 (m, 2H).

**^13^C NMR (101 MHz, CDCl_3_)** δ 148.4, 142.4, 134.9, 128.5, 128.3, 128.0, 127.9, 125.7, 117.3, 49.2, 42.5, 37.1, 36.5, 25.6.

**HRMS (pos. APCI)** m/z: [M+NH_4_]^+^ Calcd for C_25_H_30_N 344.2373. Found 344.2373.

##### but-3-ene-1,1-diyldibenzene (4-16)

([See spectra](#_1H_NMR_Spectrum_15))

The reaction was performed following the ***GP 4***. The crude mixture was first roughly purified though column chromatography on silica gel (PE) to get rid of the majority of polar side products. The residue was then carefully purified by a reverse phase column chromatography on C18 to afford the title compound as a colorless oil (23.9 mg, 57%).

**TLC** (SiO_2_): R*_f_* = 0.40 (PE)

**^1^H NMR (400 MHz, CDCl_3_)** δ 7.30 – 7.21 (m, 8H), 7.19 – 7.14 (m, 2H), 5.72 (ddt, *J* = 17.0, 10.2, 6.8 Hz, 1H), 5.02 (ddt, *J* = 17.2, 2.1, 1.5 Hz, 1H), 4.96 – 4.92 (m, 1H), 4.01 (t, *J* = 7.9 Hz, 1H), 2.82 (ddt, *J* = 8.0, 6.8, 1.3 Hz, 2H).

**^13^C NMR (101 MHz, CDCl_3_)** δ 144.6, 136.9, 128.5, 128.1, 126.3, 116.3, 51.4, 40.0.

**HRMS (pos. APCI)** m/z: [M+H]^+^ Calcd for C_16_H_17_ 209.1325. Found 209.1325.

##### 4-(2-phenyl-2-(4-(*p*-tolylthio)phenyl)pent-4-en-1-yl)tetrahydro-2*H*-pyran (4-17)

([See spectra](#_1H_NMR_Spectrum_16))

The reaction was performed following the ***GP 3***. The crude mixture was first roughly purified though column chromatography on silica gel (PE:EtOAc = 20:1) to get rid of the majority of polar side products. The residue was then carefully purified by a reverse phase column chromatography on C18 to afford the title compound as a colorless oil (66.0 mg, 77%).

**TLC** (SiO_2_): R*_f_* = 0.18 (PE:EtOAc = 20:1)

**^1^H NMR (400 MHz, CDCl_3_)** δ 7.27 – 7.20 (m, 4H), 7.19 – 7.12 (m, 5H), 7.12 – 7.05 (m, 4H), 5.32 (ddt, *J* = 17.2, 10.1, 7.0 Hz, 1H), 4.99 – 4.90 (m, 2H), 3.78 – 3.68 (m, 2H), 3.22 – 3.10 (m, 2H), 2.96 – 2.82 (m, 2H), 2.32 (d, *J* = 0.7 Hz, 3H), 2.01 (d, *J* = 4.9 Hz, 2H), 1.49 – 1.35 (m, 1H), 1.24 – 1.07 (m, 2H), 1.05 – 0.92 (m, 2H).

**^13^C NMR (101 MHz, CDCl_3_)** δ 148.0, 147.1, 137.4, 134.8, 133.9, 131.83, 131.76, 130.0, 129.8, 128.9, 128.0, 127.9, 126.0, 117.9, 68.1, 49.2, 44.5, 43.1, 35.0, 34.9, 31.2, 21.1.

**HRMS (pos. ESI)** m/z: [M+H]^+^ Calcd for C_29_H_33_OS 429.2247. Found 429.2248.

##### 4-(2-phenyl-2-(4-(trifluoromethyl)phenyl)pent-4-en-1-yl)tetrahydro-2*H*-pyran (4-18)

([See spectra](#_1H_NMR_Spectrum_17))

The reaction was performed following the ***GP 4***. The crude mixture was first roughly purified though column chromatography on silica gel (PE:EtOAc = 10:1) to get rid of the majority of polar side products. The residue was then carefully purified by a reverse phase column chromatography on C18 to afford the title compound as a colorless oil (51.9 mg, 70%).

**TLC** (SiO_2_): R*_f_* = 0.28 (PE:EtOAc = 10:1)

**^1^H NMR (500 MHz, CDCl_3_)** δ 7.50 (d, *J* = 8.3 Hz, 2H), 7.32 – 7.23 (m, 4H), 7.20 – 7.16 (m, 1H), 7.16 – 7.12 (m, 2H), 5.30 (ddt, *J* = 17.1, 10.1, 7.0 Hz, 1H), 5.01 – 4.93 (m, 2H), 3.79 – 3.68 (m, 2H), 3.18 (ddd, *J* = 11.7, 2.4 Hz, 1H), 3.15 (ddd, *J* = 11.7, 2.3 Hz, 1H), 2.97 (ddt, *J* = 13.7, 7.2, 1.3 Hz, 1H), 2.92 (ddt, *J* = 13.7, 6.8, 1.3 Hz, 1H), 2.12 – 2.02 (m, 2H), 1.48 – 1.38 (m, 1H), 1.25 – 1.09 (m, 2H), 1.04 (ddq, *J* = 13.3, 4.4, 2.3 Hz, 1H), 0.93 (ddq, *J* = 13.4, 4.4, 2.3 Hz, 1H).

**^19^F NMR (471 MHz, CDCl_3_)** δ -62.31 (s, 3F).

**^13^C NMR (126 MHz, CDCl_3_)** δ 152.7, 147.5, 134.2, 128.4, 128.2 (q, *J* = 32.4 Hz), 128.1, 128.0, 126.3, 124.8 (q, *J* = 3.8 Hz), 124.3 (q, *J* = 271.9 Hz), 118.4, 68.1, 49.6, 44.3, 42.9, 35.0, 34.8, 31.2.

**HRMS (pos. APCI)** m/z: [M+H]^+^ Calcd for C_23_H_25_F_3_O 375.1930. Found 375.1928.

##### 2-(4-(6-methyl-4-phenylhept-1-en-4-yl)phenyl)propanenitrile (4-19)

([See spectra](#_1H_NMR_Spectrum_18))

The reaction was performed following the ***GP 3***. The crude mixture was first roughly purified though column chromatography on silica gel (PE:EtOAc = 20:1) to get rid of the majority of polar side products. The residue was then carefully purified by a reverse phase column chromatography on C18 to afford the title compound as a colorless oil (45.2 mg, 71%). The diastereoselectivity was determined to be 1:1 based on both ^1^H NMR and ^13^C NMR.

**TLC** (SiO_2_): R*_f_* = 0.20 (PE:EtOAc = 20:1)

**^1^H NMR (400 MHz, CDCl_3_)** δ 7.30 – 7.20 (m, 3H), 7.21 – 7.08 (m, 6H), 5.33 (ddt, *J* = 17.1, 10.1, 7.0 Hz, 1H), 5.00 – 4.89 (m, 2H), 3.82 (q, *J* = 7.3 Hz, 1H) (*set of isomer picks 1*), 3.82 (q, *J* = 7.3 Hz, 1H) (*set of isomer picks 1*), 2.99 – 2.85 (m, 2H), 2.09 – 1.95 (m, 2H), 1.58 (d, *J* = 7.3 Hz, 3H) (*set of isomer picks 2*), 1.57 (d, *J* = 7.3 Hz, 3H) (*set of isomer picks 2*), 1.56 – 1.46 (m, 1H), 0.63 (d, *J* = 6.7 Hz, 3H) (*set of isomer picks 3*), 0.61 (d, *J* = 6.7 Hz, 3H) (*set of isomer picks 4*), 0.60 (d, *J* = 6.7 Hz, 3H) (*set of isomer picks 3*), 0.59 (d, *J* = 6.7 Hz, 3H) (*set of isomer picks 4*).

**^13^C NMR (101 MHz, CDCl_3_)** δ 150.00 (*set of isomer picks 1*), 149.97 (*set of isomer picks 1*), 148.12 (*set of isomer picks 2*), 148.09 (*set of isomer picks 2*), 136.6, 134.78 (*set of isomer picks* *3*), 134.76 (*set of isomer picks 3*), 128.60 (*set of isomer picks 4*), 128.59 (*set of isomer picks 4*), 128.18 (*set of isomer picks 5*), 128.16, 128.09 (*set of isomer picks 5*), 127.9, 126.7 (*set of isomer picks 6*), 126.6 (*set of isomer picks 6*), 125.9, 124.0, 121.7, 117.9, 49.8, 46.3, 43.00 (*set of isomer picks 7*), 42.99 (*set of isomer picks 7*), 31.4, 24.9, 24.8, 24.8, 24.1, 21.7 (*set of isomer picks 8*), 21.6 (*set of isomer picks 8*).

**HRMS (pos. APCI)** m/z: [M+H]^+^ Calcd for C_23_H_28_N 318.2216. Found 318.2218.

##### 4,4,5,5-tetramethyl-2-(4-(6-methyl-4-phenylhept-1-en-4-yl)phenyl)-1,3,2-dioxaborolane (4-20)

([See spectra](#_1H_NMR_Spectrum_19))

The reaction was performed following the ***GP 3***. The crude mixture was first roughly purified though column chromatography on silica gel (PE) to get rid of the majority of polar side products. The residue was then carefully purified by a reverse phase column chromatography on C18 to afford the title compound as a colorless oil (27.4 mg, 35%).

**TLC** (SiO_2_): R*_f_* = 0.25 (PE)

**^1^H NMR (500 MHz, CDCl_3_)** δ 7.72 – 7.66 (m, 2H), 7.25 – 7.21 (m, 2H), 7.21 – 7.17 (m, 2H), 7.17 – 7.12 (m, 3H), 5.33 (ddt, *J* = 17.1, 10.1, 7.0 Hz, 1H), 4.99 – 4.94 (m, 1H), 4.92 (ddt, *J* = 10.2, 2.2, 1.1 Hz, 1H), 2.93 (dt, *J* = 7.0, 1.4 Hz, 2H), 2.03 (d, *J* = 5.0 Hz, 2H), 1.56 – 1.47 (m, 1H), 1.34 (s, 12H), 0.62 (d, *J* = 6.7 Hz, 3H), 0.61 (d, *J* = 6.6 Hz, 3H).

**^13^C NMR (126 MHz, CDCl_3_)** δ 152.2, 148.6, 135.1, 134.3, 128.2, 127.8, 127.7, 125.7, 117.6, 83.7, 49.8, 46.0, 42.8, 24.99, 24.98, 24.9, 24.1.

**HRMS (pos. APCI)** m/z: [M+NH_4_]^+^ Calcd for C_26_H_39_BNO_2_ 408.3068. Found 408.3067.

##### 4-(6-methyl-4-phenylhept-1-en-4-yl)phenol (4-21)

([See spectra](#_1H_NMR_Spectrum_20))

The reaction was performed following the ***GP 4***. The crude mixture was first roughly purified though column chromatography on silica gel (PE:EtOAc = 9:1) to get rid of the majority of side products. The residue was then carefully purified by a reverse phase column chromatography on C18 to afford the title compound as a yellow oil (38.6 mg, 69%).

**TLC** (SiO_2_): R*_f_* = 0.25 (PE:EtOAc = 9:1)

**^1^H NMR (400 MHz, CDCl_3_)** δ 7.26 – 7.20 (m, 2H), 7.19 – 7.11 (m, 3H), 7.05 – 6.99 (m, 2H), 6.73 – 6.67 (m, 2H), 5.35 (ddt, *J* = 17.1, 10.1, 7.0 Hz, 1H), 5.00 – 4.88 (m, 2H), 4.72 (br, s, 1H), 2.93 – 2.82 (m, 2H), 1.98 (d, *J* = 5.0 Hz, 2H), 1.57 – 1.44 (m, 1H), 0.62 (d, *J* = 7.9 Hz, 3H), 0.60 (d, *J* = 7.9 Hz, 3H).

**^13^C NMR (101 MHz, CDCl_3_)** δ 153.3, 148.9, 141.2, 135.3, 129.4, 128.2, 127.8, 125.6, 117.5, 114.6, 49.0, 46.4, 43.1, 24.93, 24.88, 24.2.

**HRMS (pos. APCI)** m/z: [M+H]^+^ Calcd for C_20_H_25_O 281.1900. Found 281.1901.

##### *N*-(4-(6-methyl-4-phenylhept-1-en-4-yl)phenyl)acetamide (4-22)

([See spectra](#_1H_NMR_Spectrum_21))

The reaction was performed following the ***GP 3***. The crude mixture was first roughly purified though column chromatography on silica gel (PE:EtOAc = 2:1) to get rid of the majority of side products. The residue was then carefully purified by a reverse phase column chromatography on C18 to afford the title compound as a white solid (46.2 mg, 72%).

**TLC** (SiO_2_): R*_f_* = 0.26 (PE:EtOAc = 2:1)

**^1^H NMR (400 MHz, CDCl_3_)** δ 7.43 (br, s, 1H), 7.41 – 7.35 (m, 2H), 7.25 – 7.19 (m, 2H), 7.18 – 7.06 (m, 5H), 5.34 (ddt, *J* = 17.1, 10.1, 7.0 Hz, 1H), 5.00 – 4.88 (m, 2H), 2.97 – 2.83 (m, 2H), 2.13 (s, 3H), 1.99 (d, *J* = 5.1 Hz, 2H), 1.58 – 1.44 (m, 1H), 0.65 – 0.57 (m, 6H).

**^13^C NMR (101 MHz, CDCl_3_)** δ 168.4, 148.6, 144.8, 135.6, 135.1, 128.7, 128.2, 127.8, 125.7, 119.2, 117.6, 49.2, 46.2, 42.9, 24.9, 24.8, 24.6, 24.1.

**HRMS (pos. ESI)** m/z: [M+H]^+^ Calcd for C_22_H_28_ON 322.2165. Found 322.2169.

**Melting point**: 95-97 °C.

##### 4-(6-methyl-4-phenylhept-1-en-4-yl)benzoic acid (4-23)

([See spectra](#_1H_NMR_Spectrum_22))

The reaction was performed following the ***GP 3***. The crude mixture was first roughly purified though column chromatography on silica gel (PE:EtOAc = 4:1, acidified with 1 mol% HCO_2_H) to get rid of the majority of side products. The residue was then carefully purified by a reverse phase column chromatography on C18 to afford the title compound as a colorless oil (46.4 mg, 75%).

**^1^H NMR (400 MHz, CDCl_3_)** δ 8.03 – 7.96 (m, 2H), 7.32 – 7.22 (m, 4H), 7.19 – 7.12 (m, 3H), 5.33 (ddt, *J* = 17.1, 10.1, 7.0 Hz, 1H), 5.01 – 4.90 (m, 2H), 3.02 – 2.89 (m, 2H), 2.06 (d, *J* = 5.1 Hz, 2H), 1.59 – 1.46 (m, 1H), 0.63 (d, *J* = 6.7 Hz, 3H), 0.62 (d, *J* = 6.6 Hz, 3H).

**^13^C NMR (101 MHz, CDCl_3_)** δ 172.2, 155.5, 147.8, 134.5, 129.8, 128.4, 128.1, 128.0, 126.9, 126.1, 118.1, 50.1, 46.1, 42.8, 24.9, 24.8, 24.2.

**TLC** (SiO_2_): R*_f_* = 0.30 (PE:EtOAc = 4:1, acidified with 1 mol% HCO_2_H)

**HRMS (neg. ESI)** m/z: [M-H]^-^ Calcd for C_21_H_23_O_2_ 307.1704. Found 307.1702.

##### 4,4'-(6-methylhept-1-ene-4,4-diyl)bis(methoxybenzene) (4-24)

([See spectra](#_1H_NMR_Spectrum_23))

The reaction was performed following the ***GP 3***. The crude mixture was first roughly purified though column chromatography on silica gel (PE:EtOAc = 40:1) to get rid of the majority of polar side products. The residue was then carefully purified by a reverse phase column chromatography on C18 to afford the title compound as a colorless oil (48.3 mg, 75%).

**TLC** (SiO_2_): R*_f_* = 0.33 (PE:EtOAc = 40:1)

**^1^H NMR (400 MHz, CDCl_3_)** δ 7.13 – 7.03 (m, 4H), 6.82 – 6.73 (m, 4H), 5.36 (ddt, *J* = 17.1, 10.1, 7.0 Hz, 1H), 4.99 – 4.88 (m, 2H), 3.77 (s, 6H), 2.86 (dt, *J* = 6.9, 1.3 Hz, 2H), 1.95 (d, *J* = 5.1 Hz, 2H), 1.57 – 1.45 (m, 1H), 0.62 (d, *J* = 6.7 Hz, 6H).

**^13^C NMR (101 MHz, CDCl_3_)** δ 157.5, 141.1, 135.5, 129.1, 117.3, 113.1, 55.2, 48.3, 46.6, 43.3, 24.9, 24.2.

**HRMS (pos. APCI)** m/z: [M+H]^+^ Calcd for C_22_H_29_O_2_ 325.2162. Found 325.2165.

##### 1-fluoro-4-(4-(4-methoxyphenyl)-6-methylhept-1-en-4-yl)benzene (4-25)

([See spectra](#_1H_NMR_Spectrum_24))

The reaction was performed following the ***GP 3***. The crude mixture was first roughly purified though column chromatography on silica gel (PE:EtOAc = 100:1) to get rid of the majority of polar side products. The residue was then carefully purified by a reverse phase column chromatography on C18 to afford the title compound as a colorless oil (58.5 mg, 83%).

**TLC** (SiO_2_): R*_f_* = 0.32 (PE:EtOAc = 100:1)

**^1^H NMR (500 MHz, CDCl_3_)** δ 7.15 – 7.07 (m, 2H), 7.08 – 7.01 (m, 2H), 6.95 – 6.87 (m, 2H), 6.81 – 6.75 (m, 2H), 5.34 (ddt, *J* = 17.1, 10.1, 6.9 Hz, 1H), 4.97 – 4.88 (m, 2H), 3.78 (s, 3H), 2.89 – 2.80 (m, 2H), 1.91 (d, *J* = 4.2 Hz, 2H), 1.54 – 1.43 (m, 3H), 1.21 – 1.11 (m, 3H), 1.09 – 0.97 (m, 3H), 0.86 – 0.72 (m, 2H).

**^19^F NMR (471 MHz, CDCl_3_)** δ -117.88 – -118.05 (m,1F).

**^13^C NMR (126 MHz, CDCl_3_)** δ 160.9 (d, *J* = 244.1 Hz), 157.5, 144.8 (d, *J* = 3.3 Hz), 140.6, 135.1, 129.6 (d, *J* = 7.6 Hz), 129.0, 117.7, 114.4 (d, *J* = 20.8 Hz), 113.1, 55.2, 48.6, 45.2, 43.3, 35.34, 35.31, 33.7, 26.6, 26.3.

**HRMS (pos. APCI)** m/z: [M+H]^+^ Calcd for C_24_H_30_FO 353.2275. Found 353.2274.

##### 4-bromo-2-chloro-1-(4-(4-ethoxyphenyl)-6-methylhept-1-en-4-yl)benzene (4-26)

([See spectra](#_1H_NMR_Spectrum_25))

The reaction was performed following the ***GP 3***. The crude mixture was first roughly purified though column chromatography on silica gel (PE:EtOAc = 100:1) to get rid of the majority of polar side products. The residue was then carefully purified by a reverse phase column chromatography on C18 to afford the title compound as a colorless oil (42.8 mg, 51%).

**TLC** (SiO_2_): R*_f_* = 0.18 (PE:EtOAc = 100:1)

**^1^H NMR (400 MHz, CDCl_3_)** δ 7.68 (d, *J* = 2.4 Hz, 1H), 7.29 (dd, *J* = 8.4, 2.4 Hz, 1H), 7.09 (d, *J* = 8.4 Hz, 1H), 6.97 – 6.90 (m, 2H), 6.79 – 6.72 (m, 2H), 5.29 (dddd, *J* = 17.5, 10.0, 7.5, 6.4 Hz, 1H), 5.03 – 4.91 (m, 2H), 3.99 (q, *J* = 7.0 Hz, 2H), 3.19 (dd, *J* = 13.4, 7.6 Hz, 1H), 2.80 – 2.71 (m, 1H), 2.32 (dd, *J* = 13.5, 5.3 Hz, 1H), 1.92 (dd, *J* = 13.5, 4.5 Hz, 1H), 1.52 – 1.42 (m, 1H), 1.38 (t, *J* = 7.0 Hz, 3H), 0.68 (d, *J* = 6.7 Hz, 3H), 0.62 (d, *J* = 6.7 Hz, 3H).

**^13^C NMR (101 MHz, CDCl_3_)** δ 157.0, 147.1, 138.5, 134.6, 134.1, 133.2, 132.4, 130.6, 128.2, 120.4, 118.1, 113.8, 63.4, 49.4, 43.6, 41.6, 25.1, 24.5, 24.3, 15.0.

**HRMS (pos. APCI)** m/z: [M+H]^+^ Calcd for C_22_H_27_^81^Br^35^ClO 423.0908. Found 423.0911.

##### *tert*-butyl (3-(4-chloro-2-(methylamino)phenyl)-3-phenylhex-5-en-1-yl)(methyl)carbamate (4-27)

([See spectra](#_1H_NMR_Spectrum_26))

The reaction was performed following the ***GP 4***. The crude mixture was first roughly purified though column chromatography on silica gel (PE:EtOAc = 10:1) to get rid of the majority of polar side products. The residue was then carefully purified by a reverse phase column chromatography on C18 to afford the title compound as a colorless oil (48.8 mg, 59%).

**TLC** (SiO_2_): R*_f_* = 0.20 (PE:EtOAc = 10:1)

**^1^H NMR (400 MHz, CDCl_3_)** δ 7.30 (tt, *J* = 6.9, 1.1 Hz, 3H), 7.25 – 7.18 (m, 3H), 7.15 (dd, *J* = 8.6, 2.4 Hz, 1H), 6.45 (d, *J* = 8.7 Hz, 1H), 5.31 (ddt, *J* = 17.1, 10.0, 7.0 Hz, 1H), 5.08 – 4.95 (m, 2H), 3.20 (br, s, 1H), 3.05 (dd, *J* = 13.4, 7.1 Hz, 1H), 3.01 – 2.87 (m, 1H), 2.81 – 2.56 (m, 5H), 2.47 (td, *J* = 12.0, 4.6 Hz, 1H), 2.42 (s, 3H), 2.13 (td, *J* = 12.0, 4.8 Hz, 1H), 1.38 (s, 9H).

**^13^C NMR (101 MHz, CDCl_3_)** δ 155.6, 145.7, 145.5, 134.0, 131.8, 128.9, 127.6, 127.4, 127.0, 126.7, 121.8, 118.2, 112.6, 79.3, 46.9, 45.3, 40.3, 34.0, 31.8, 30.9, 28.5.

**HRMS (pos. ESI)** m/z: [M+Na]^+^ Calcd for C_25_H_33_O_2_N_2_ClNa 451.2123. Found 451.2116.

##### 9-allyl-9-(cyclohexylmethyl)-10,10-dimethyl-9,10-dihydroanthracene (4-28)

([See spectra](#_1H_NMR_Spectrum_27))

The reaction was performed following the ***GP 3***. The crude mixture was first roughly purified though column chromatography on silica gel (PE) to get rid of the majority of polar side products. The residue was then carefully purified by a reverse phase column chromatography on C18 to afford the title compound as a colorless oil (26.3 mg, 38%).

**TLC** (SiO_2_): R*_f_* = 0.56 (PE)

**^1^H NMR (400 MHz, CDCl_3_)** δ 7.48 – 7.43 (m, 2H), 7.42 – 7.37 (m, 2H), 7.24 – 7.17 (m, 4H), 5.04 (ddt, *J* = 16.7, 10.7, 7.1 Hz, 1H), 4.57 – 4.50 (m, 2H), 2.75 (dt, *J* = 7.1, 1.2 Hz, 2H), 2.05 (d, *J* = 5.4 Hz, 2H), 1.64 (s, 3H), 1.61 (s, 3H), 1.39 – 1.28 (m, 3H), 1.02 – 0.94 (m, 2H), 0.94 – 0.72 (m, 4H), 0.64 – 0.52 (m, 2H).

**^13^C NMR (101 MHz, CDCl_3_)** δ 144.0, 137.8, 134.8, 126.73, 126.67, 126.0, 125.9, 116.6, 53.4, 52.3, 45.4, 36.9, 36.0, 35.1, 34.7, 34.6, 26.33, 26.25.

**HRMS (pos. APCI)** m/z: [M+H]^+^ Calcd for C_26_H_33_ 345.2577. Found 345.2572.

##### 11-allyl-11-(cyclopentylmethyl)-6,11-dihydrodibenzo[*b,e*]thiepine (4-29)

([See spectra](#_1H_NMR_Spectrum_28))

The reaction was performed following the ***GP 3***. The crude mixture was first roughly purified though column chromatography on silica gel (PE) to get rid of the majority of polar side products. The residue was then carefully purified by a reverse phase column chromatography on C18 to afford the title compound as a colorless oil (27.1 mg, 41%).

**TLC** (SiO_2_): R*_f_* = 0.39 (PE)

**^1^H NMR (400 MHz, CDCl_3_)** δ 7.52 (dd, *J* = 8.2, 1.4 Hz, 1H), 7.50 (dd, *J* = 8.2, 1.3 Hz, 1H), 7.25 (dd, *J* = 7.7, 1.6 Hz, 1H), 7.19 (ddd, *J* = 8.1, 7.2, 1.7 Hz, 1H), 7.14 – 7.11 (m, 1H), 7.11 – 7.07 (m, 1H), 7.02 (dd, *J* = 7.5, 1.6 Hz, 1H), 7.00 (ddd, *J* = 7.7, 7.2, 1.4 Hz, 1H), 5.71 – 5.56 (m, 1H), 4.99 – 4.90 (m, 2H), 4.25 (d, *J* = 14.1 Hz, 1H), 4.04 (d, *J* = 14.0 Hz, 1H), 3.26 (dd, *J* = 15.4, 6.6 Hz, 1H), 3.06 (ddt, *J* = 15.3, 6.6, 1.5 Hz, 1H), 2.79 (dd, *J* = 14.4, 6.4 Hz, 1H), 2.38 (dd, *J* = 14.4, 5.1 Hz, 1H), 1.79 – 1.65 (m, 1H), 1.57 – 1.38 (m, 4H), 1.38 – 1.27 (m, 2H), 0.98 – 0.78 (m, 2H).

**^13^C NMR (101 MHz, CDCl_3_)** δ 144.4, 143.9, 135.2, 131.8, 129.6, 129.5, 129.1, 127.4, 126.5, 126.1, 125.9, 117.5, 40.5, 37.5, 34.4, 33.8, 29.8, 25.1, 24.7.

**HRMS (pos. APCI)** m/z: [M+H]^+^ Calcd for C_23_H_27_S 335.1828. Found 335.1827.

##### 4-(4-methylhept-1-en-4-yl)-1,1'-biphenyl (4-30)

([See spectra](#_1H_NMR_Spectrum_29))

The reaction was performed following the ***GP 4***. The crude mixture was first roughly purified though column chromatography on silica gel (PE) to get rid of the majority of polar side products. The residue was then carefully purified by a reverse phase column chromatography on C18 to afford the title compound as a colorless oil (18.7 mg, 35%).

**TLC** (SiO_2_): R*_f_* = 0.52 (PE)

**^1^H NMR (400 MHz, CDCl_3_)** δ 7.61 – 7.56 (m, 2H), 7.54 – 7.49 (m, 2H), 7.44 – 7.38 (m, 2H), 7.33 – 7.28 (m, 1H), 7.24 – 7.19 (m, 2H), 5.69 (ddt, *J* = 17.1, 10.1, 7.0 Hz, 1H), 4.97 (ddt, *J* = 17.2, 2.3, 1.5 Hz, 1H), 4.93 (ddt, *J* = 10.2, 2.2, 1.1 Hz, 1H), 2.74 (dtd, *J* = 10.0, 7.2, 5.0 Hz, 1H), 2.38 – 2.31 (m, 2H), 1.58 (ddd, *J* = 13.2, 9.9, 4.8 Hz, 1H), 1.50 – 1.36 (m, 2H), 0.88 (d, *J* = 6.3 Hz, 3H), 0.84 (d, *J* = 6.4 Hz, 3H).

**^13^C NMR (101 MHz, CDCl_3_)** δ 144.8, 141.2, 138.9, 137.3, 128.8, 128.2, 127.04, 127.02, 115.9, 45.4, 43.3, 42.0, 25.4, 23.7, 21.9.

**HRMS (pos. APCI)** m/z: [M+H]^+^ Calcd for C_20_H_25_ 265.1951. Found 265.1954.

##### 9-(3-phenylhex-5-en-1-yl)-9*H*-carbazole (4-31)

([See spectra](#_1H_NMR_Spectrum_34))

The reaction was performed following the ***GP 4***. The crude mixture was first roughly purified though column chromatography on silica gel (PE:EtOAc = 100:1) to get rid of the majority of polar side products. The residue was then carefully purified by a reverse phase column chromatography on C18 to afford the title compound as a colorless oil (22.3 mg, 34%).

**TLC** (SiO_2_): R*_f_* = 0.02 (PE:EtOAc = 100:1)

**^1^H NMR (700 MHz, CDCl_3_)** δ 8.10 – 8.03 (m, 2H), 7.42 – 7.36 (m, 4H), 7.31 – 7.27 (m, 1H), 7.26 – 7.23 (m, 2H), 7.21 – 7.18 (m, 2H), 7.16 – 7.14 (m, 2H), 5.62 (dddd, *J* = 16.9, 10.2, 7.4, 6.6 Hz, 1H), 4.97 – 4.90 (m, 2H), 4.16 (ddd, *J* = 14.9, 10.0, 6.6 Hz, 1H), 4.06 (ddd, *J* = 15.0, 10.3, 4.8 Hz, 1H), 2.78 – 2.71 (m, 1H), 2.42 – 2.32 (m, 2H), 2.28 (dddd, *J* = 13.8, 10.3, 6.6, 4.3 Hz, 1H), 2.07 (dtd, *J* = 13.9, 10.2, 4.8 Hz, 1H).

**^13^C NMR (176 MHz, CDCl_3_)** δ 144.0, 140.2, 136.3, 128.8, 127.8, 126.8, 125.6, 123.0, 120.4, 118.8, 116.6, 108.6, 43.7, 41.7, 41.3, 34.1.

**HRMS (pos. ESI)** m/z: [M+H]^+^ Calcd for C_24_H_24_N 326.1903. Found 326.1905.

**Melting point:** 68-70 °C

##### 4,4,5,5-tetramethyl-2-(6-methyl-4-phenylhept-1-en-4-yl)-1,3,2-dioxaborolane (4-32)

([See spectra](#_1H_NMR_Spectrum_30))

The reaction was performed following the ***GP 4***. The crude mixture was first roughly purified though column chromatography on silica gel (PE:EtOAc = 40:1) to get rid of the majority of polar side products. The residue was then carefully purified by a reverse phase column chromatography on C18 to afford the title compound as a colorless oil (23.2 mg, 37%).

**TLC** (SiO_2_): R*_f_* = 0.15 (PE:EtOAc = 40:1)

**^1^H NMR (500 MHz, CDCl_3_)** δ 7.37 – 7.33 (m, 2H), 7.28 – 7.23 (m, 2H), 7.13 – 7.09 (m, 1H), 5.63 (ddt, *J* = 17.2, 10.2, 7.0 Hz, 1H), 5.03 (ddd, *J* = 17.1, 2.5, 1.3 Hz, 1H), 4.94 (ddt, *J* = 10.2, 2.4, 1.2 Hz, 1H), 2.73 (ddt, *J* = 14.4, 6.9, 1.4 Hz, 1H), 2.64 (ddt, *J* = 14.4, 7.2, 1.4 Hz, 1H), 1.83 – 1.74 (m, 2H), 1.63 – 1.55 (m, 1H), 1.19 (s, 6H), 1.16 (s, 6H), 0.84 (d, *J* = 6.7 Hz, 3H), 0.72 (d, *J* = 6.6 Hz, 3H).

**^13^C NMR (126 MHz, CDCl_3_)** δ 145.6, 136.2, 128.0, 127.6, 125.1, 116.7, 83.4, 43.7, 38.8, 25.2, 24.9, 24.8, 24.4, 24.2.

**HRMS (pos. APCI)** m/z: [M+H]^+^ Calcd for C_20_H_32_^11^BO_2_ 315.2490. Found 315.2493.

##### methyl 2-phenyl-2-propylpent-4-enoate (4-33)

([See spectra](#_1H_NMR_Spectrum_31))

The reaction was performed following the ***GP 4***. The crude mixture was first roughly purified though column chromatography on silica gel (PE:EtOAc = 40:1) to get rid of the majority of polar side products. The residue was then carefully purified by a reverse phase column chromatography on C18 to afford the title compound as a colorless oil (14.9 mg, 30%).

**TLC** (SiO_2_): R*_f_* = 0.20 (PE:EtOAc = 40:1)

**^1^H NMR (400 MHz, CDCl_3_)** δ 7.34 – 7.29 (m, 2H), 7.28 – 7.25 (m, 2H), 7.25 – 7.20 (m, 1H), 5.48 (ddt, *J* = 17.2, 10.2, 7.2 Hz, 1H), 5.05 – 4.97 (m, 2H), 3.62 (s, 3H), 2.85 (dt, *J* = 7.2, 1.3 Hz, 2H), 1.98 (d, *J* = 5.9 Hz, 2H), 1.69 – 1.58 (m, 1H), 0.83 (d, *J* = 6.7 Hz, 3H), 0.76 (d, *J* = 6.6 Hz, 3H).

**^13^C NMR (101 MHz, CDCl_3_)** δ 176.5, 142.8, 133.9, 128.3, 126.8, 126.6, 118.3, 53.5, 51.9, 43.1, 39.8, 24.7, 24.4, 23.6.

**HRMS (pos. APCI)** m/z: [M+H]^+^ Calcd for C_16_H_23_O_2_ 247.1693. Found 247.1690.

##### 6-methyl-4-phenylhept-1-en-4-yl acetate (4-34)

([See spectra](#_1H_NMR_Spectrum_32))

The reaction was performed following the ***GP 3***. The crude mixture was first roughly purified though column chromatography on silica gel (PE:EtOAc = 40:1) to get rid of the majority of polar side products. The residue was then carefully purified by a reverse phase column chromatography on C18 to afford the title compound as a colorless oil (19.5 mg, 40%).

**TLC** (SiO_2_): R*_f_* = 0.28 (PE:EtOAc = 40:1)

**^1^H NMR (400 MHz, CDCl_3_)** δ 7.36 – 7.31 (m, 4H), 7.25 – 7.20 (m, 1H), 5.44 (dddd, *J* = 17.1, 10.2, 8.1, 6.3 Hz, 1H), 5.03 – 4.95 (m, 2H), 3.26 (ddt, *J* = 14.3, 8.1, 1.1 Hz, 1H), 2.83 (ddt, *J* = 14.3, 6.3, 1.5 Hz, 1H), 2.43 (dd, *J* = 14.6, 7.2 Hz, 1H), 1.89 (dd, *J* = 14.7, 5.1 Hz, 1H), 1.51 – 1.40 (m, 1H), 0.79 (d, *J* = 6.6 Hz, 3H), 0.69 (d, *J* = 6.7 Hz, 3H).

**^13^C NMR (101 MHz, CDCl_3_)** δ 169.7, 143.6, 132.8, 128.1, 126.8, 125.2, 118.3, 86.9, 46.3, 43.4, 24.3, 24.1, 23.4, 22.4.

**HRMS (pos. ESI)** m/z: [M+NH_4_]^+^ Calcd for C_16_H_26_O_2_N 264.1958. Found 264.1961.

##### *N*-(4-phenylhept-1-en-4-yl)acetamide (4-35)

([See spectra](#_1H_NMR_Spectrum_33))

The reaction was performed following the ***GP 3***. The crude mixture was first roughly purified though column chromatography on silica gel (PE:EtOAc = 40:1) to get rid of the majority of side products. The residue was then carefully purified by a reverse phase column chromatography on C18 to afford the title compound as a white solid (19.5 mg, 40%).

**TLC** (SiO_2_): R*_f_* = 0.28 (PE:EtOAc = 40:1)

**^1^H NMR (400 MHz, CDCl_3_)** δ 7.35 – 7.27 (m, 4H), 7.24 – 7.19 (m, 1H), 5.64 – 5.52 (m, 2H), 5.12 – 5.04 (m, 2H), 3.04 (ddt, *J* = 13.8, 6.5, 1.3 Hz, 1H), 2.84 (ddt, *J* = 13.7, 8.0, 1.0 Hz, 1H), 2.04 – 1.98 (m, 4H), 1.92 (dd, *J* = 14.1, 6.2 Hz, 1H), 1.57 – 1.46 (m, 1H), 0.75 (d, *J* = 6.7 Hz, 6H).

**^13^C NMR (101 MHz, CDCl_3_)** δ 169.2, 144.2, 133.9, 128.3, 126.6, 125.6, 118.8, 61.9, 47.5, 42.1, 24.6, 24.5, 24.4, 24.0.

**HRMS (pos. APCI)** m/z: [M+H]^+^ Calcd for C_16_H_24_NO 246.1852. Found 246.1855.

**Melting point:** 125-127 °C

##### *tert*-butyl methyl(3-phenyl-3-(trifluoromethyl)hex-5-en-1-yl)carbamate (4-36)

([See spectra](#_1H_NMR_Spectrum_69))

The reaction was performed following the ***GP 4***. The crude mixture was first roughly purified though column chromatography on silica gel (PE:EtOAc = 20:1) to get rid of the majority of polar side products. The residue was then carefully purified by a reverse phase column chromatography on C18 to afford the title compound as a colorless oil (34.7 mg, 49%).

**TLC** (SiO_2_): R*_f_* = 0.32 (PE:EtOAc = 20:1)

^1^**H NMR (500 MHz, CDCl_3_, 333 K)** δ 7.52 – 7.46 (m, 2H), 7.38 – 7.33 (m, 2H), 7.31 – 7.27 (m, 1H), 5.80 – 5.68 (m, 1H), 5.16 (dq, *J* = 17.0, 1.6 Hz, 1H), 5.11 (dq, *J* = 10.2, 1.3 Hz, 1H), 3.25 (ddd, *J* = 13.8, 11.1, 5.6 Hz, 1H), 3.16 (ddd, *J* = 13.9, 10.9, 5.7 Hz, 1H), 2.86 (ddt, *J* = 15.0, 7.1, 1.4 Hz, 1H), 2.80 (s, 3H), 2.78 – 2.72 (m, 1H), 2.31 – 2.17 (m, 2H), 1.46 (s, 9H).

**^19^F NMR (471 MHz, CDCl_3_****, 333 K)** δ -69.93 (s, 3F).

**^13^C NMR (126 MHz, CDCl_3_, 333 K)** δ 155.6, 137.1, 132.7, 128.5, 128.2 (q, *J* = 285.1 Hz), 128.0, 127.8, 119.0, 79.7, 49.8 (q, *J* = 23.2 Hz), 44.6, 38.2 (q, *J* = 2.2 Hz), 34.4, 30.5, 28.7.

**HRMS (pos. APCI)** m/z: [M+H]^+^ Calcd for C_19_H_27_O_2_NF_3_ 358.1988. Found 358.1990.

##### 5-(2-methyl-4-phenyl-4-(trifluoromethyl)hept-6-en-1-yl)benzo[*d*][1,3]dioxole (4-37)

([See spectra](#_1H_NMR_Spectrum_70))

The reaction was performed following a slightly modified ***GP 3*** using (η^3^-cinnamyl)PdCp instead of Pd(OAc)_2_. The crude mixture was first roughly purified though column chromatography on silica gel (PE:EtOAc = 40:1) to get rid of the majority of polar side products. The residue was then carefully purified by a reverse phase column chromatography on C18 to afford the title compound as a colorless oil (35.2 mg, 48%). The diastereoselectivity was determined to be 1.3:1 based on both ^1^H NMR and ^19^F NMR.

**TLC** (SiO_2_): R*_f_* = 0.28 (PE:EtOAc = 40:1)

**^1^H NMR (500 MHz, CDCl_3_)** δ 7.48 – 7.41 (m, 2H), 7.36 – 7.31 (m, 2H), 7.30 – 7.26 (m, 1H), 6.68 (dd, *J* = 7.8, 0.5 Hz, 0.58H) (major), 6.67 – 6.64 (m, 0.43H) (Minor), 6.50 – 6.46 (m, 1H), 6.43 – 6.39 (m, 1H), 5.90 (d, *J* = 1.5 Hz, 0.58H) (major), 5.89 (d, *J* = 1.5 Hz, 0.58H) (major), 5.89 (d, *J* = 1.6 Hz, 0.43H) (Minor), 5.88 (d, *J* = 1.4 Hz, 0.43H) (Minor), 5.82 – 5.69 (m, 1H), 5.17 – 5.12 (m, 1H), 5.09 – 5.05 (m, 1H), 2.97 – 2.87 (m, 1H), 2.78 (ddt, *J* = 15.3, 7.0, 1.5 Hz, 0.43H) (Minor), 2.72 (ddt, *J* = 15.1, 7.0, 1.4 Hz, 0.58H) (major), 2.51 (dd, *J* = 13.4, 5.3 Hz, 0.58H) (major), 2.37 (dd, *J* = 13.4, 5.2 Hz, 0.46H) (Minor), 2.17 (dd, *J* = 13.4, 9.0 Hz, 0.60H) (major), 2.14 – 2.04 (m, 1.45H) (Major + Minor), 1.94 (dd, *J* = 14.8, 7.0 Hz, 0.60H) (major), 1.89 (dd, *J* = 14.8, 6.5 Hz, 0.45H) (Minor), 1.85 – 1.75 (m, 0.59H) (major), 1.75 – 1.66 (m, 0.44H) (Minor), 0.77 (d, *J* = 6.6 Hz, 1.33H) (Minor), 0.56 (d, *J* = 6.6 Hz, 1.73H) (major).

*Note: Unless otherwise noted, the integration refers to the sum of both isomers.*

**^19^F NMR (471 MHz, CDCl_3_) (Major)** δ -68.48 (s, 3F).

**^19^F NMR (471 MHz, CDCl_3_) (Minor)** δ -68.25 (s, 3F).

**^13^C NMR (126 MHz, CDCl_3_) (Major)** δ 147.50, 145.73, 137.98, 134.77, 133.41, 128.47 (q, *J* = 285.6 Hz), 128.24, 128.10, 127.51, 122.09, 118.42, 109.51, 107.99, 100.80, 51.21 (q, *J* = 22.3 Hz), 45.07, 40.63, 38.49 (q, *J* = 2.2 Hz), 31.16, 20.99.

**^13^C NMR (126 MHz, CDCl_3_) (Minor)** δ 147.46, 145.69, 137.78, 134.80, 133.41, 128.41 (d, *J* = 285.7 Hz), 128.28, 128.10, 127.51, 122.04, 118.42, 109.44, 107.95, 100.78, 51.59 (q, *J* = 22.1 Hz), 44.91, 40.67, 38.60 (d, *J* = 2.2 Hz), 31.13, 21.44.

**HRMS (pos. APCI)** m/z: [M+H]^+^ Calcd for C_22_H_24_F_3_O_2_ 377.1723. Found 377.1719.

##### tert-butyl (2-(1-allyl-1,2,3,4-tetrahydronaphthalen-1-yl)ethyl)(methyl)carbamate (4-38)

([See spectra](#_1H_NMR_Spectrum_36))

The reaction was performed following the ***GP 4***. The crude mixture was first roughly purified though column chromatography on silica gel (PE:EtOAc = 10:1) to get rid of the majority of side products. The residue was then carefully purified by a reverse phase column chromatography on C18 to afford the title compound as a colorless oil (30.1 mg, 46%).

**TLC** (SiO_2_): R*_f_* = 0.25 (PE:EtOAc = 10:1)

**^1^H NMR (500 MHz, CDCl_3_, 333 K)** δ 7.25 (dd, *J* = 7.8, 1.3 Hz, 1H), 7.12 (dddd, *J* = 7.8, 6.9, 1.8, 0.8 Hz, 1H), 7.08 – 7.00 (m, 2H), 5.70 – 5.61 (m, 1H), 5.03 – 4.97 (m, 2H), 3.18 (br, s, 1H), 2.90 (ddd, *J* = 13.7, 11.4, 4.8 Hz, 1H), 2.74 (s, 3H), 2.74 – 2.68 (m, 2H), 2.50 (ddt, *J* = 14.0, 6.8, 1.4 Hz, 1H), 2.30 (ddt, *J* = 14.0, 7.8, 1.1 Hz, 1H), 1.98 (ddd, *J* = 13.4, 11.8, 5.0 Hz, 1H), 1.87 – 1.68 (m, 5H), 1.43 (s, 9H).

**^13^C NMR (126 MHz, CDCl_3_, 333 K)** δ 155.7, 142.1, 137.6, 135.2, 129.3, 126.9, 125.9, 125.6, 117.4, 79.2, 47.7, 45.5, 39.3, 39.0, 34.2, 32.3, 30.6, 28.6, 19.8.

**HRMS (pos. ESI)** m/z: [M+Na]^+^ Calcd for C_21_H_31_O_2_NNa 352.2247. Found 352.2244.

(4-39)

##### (*E*)-4-(2,2,5-triphenylpent-4-en-1-yl)tetrahydro-2*H*-pyran (4-39*L-E*)

([See spectra](#_1H_NMR_Spectrum_38))

##### 4-(2,2,3-triphenylpent-4-en-1-yl)tetrahydro-2H-pyran (4-39*B*)

([See spectra](#_1H_NMR_Spectrum_37))

The reaction was performed following the ***GP 4***. The crude mixture was first roughly purified though column chromatography on silica gel (PE:EtOAc = 10:1) to get rid of the majority of side products. The residue was then carefully purified by a reverse phase column chromatography on C18 to afford the title compounds as a colorless oil (Combined yield: 51.3 mg, 67%). The *L:B* selectivity was determined to be 1.3:1 and the *E:Z* selectivity for the linear product was determined to be over 20:1 based on crude ^1^H NMR (*L:B* = 1.3:1)(*E:Z* > 20:1).

**Characterization data for 4-39*L-E***

**TLC** (SiO_2_): R*_f_* = 0.18 (PE:EtOAc = 10:1)

**^1^H NMR (500 MHz, CDCl_3_)** δ 7.29 – 7.22 (m, 5H), 7.22 – 7.13 (m, 10H), 6.29 (dt, *J* = 15.8 Hz, *J* = 1.5 Hz, 1H), 5.70 (dt, *J* = 15.9, 7.2 Hz, 1H), 3.73 (ddd, *J* = 11.7, 3.8, 1.8 Hz, 2H), 3.18 (td, *J* = 11.6, 2.1 Hz, 2H), 3.06 (dd, *J* = 7.2, 1.4 Hz, 2H), 2.07 (d, *J* = 4.9 Hz, 2H), 1.55 – 1.43 (m, 1H), 1.16 (dtd, *J* = 13.4, 11.6, 4.3 Hz, 2H), 0.98 (ddd, *J* = 13.2, 4.2, 2.0 Hz, 2H).

**^13^C NMR (126 MHz, CDCl_3_)** δ 148.4, 137.7, 132.9, 128.5, 128.1, 128.0, 127.1, 127.0, 126.05, 126.00, 68.2, 50.0, 44.8, 42.5, 35.0, 31.3.

**HRMS (pos. APCI)** m/z: [M+H]^+^ Calcd for C_28_H_31_O 383.2369. Found 383.2372.

**Characterization data for 4-39*B*** (Containing 8% linear isomer)

**TLC** (SiO_2_): R*_f_* = 0.18 (PE:EtOAc = 10:1)

**^1^H NMR (500 MHz, CDCl_3_)** δ 7.40 – 7.32 (m, 2H), 7.32 – 7.25 (m, 5H), 7.23 – 7.19 (m, 1H), 7.16 – 7.10 (m, 3H), 7.09 – 7.05 (m, 2H), 6.51 – 6.40 (m, 2H), 6.16 (ddd, *J* = 17.1, 10.4, 7.8 Hz, 1H), 5.04 (ddd, *J* = 10.4, 1.7, 1.2 Hz, 1H), 4.95 (dt, *J* = 17.1, 1.5 Hz, 1H), 4.44 (dt, *J* = 7.9, 1.4 Hz, 1H), 3.68 – 3.58 (m, 2H), 3.09 (td, *J* = 11.7, 2.2 Hz, 1H), 3.05 (td, *J* = 11.7, 2.2 Hz, 1H), 1.85 (dd, *J* = 14.3, 4.5 Hz, 1H), 1.70 (dd, *J* = 14.3, 4.9 Hz, 1H), 1.45 – 1.34 (m, 1H), 1.00 (dtd, *J* = 13.4, 11.7, 4.3 Hz, 1H), 0.91 (dtd, *J* = 13.4, 11.8, 4.3 Hz, 1H), 0.73 (ddt, *J* = 13.3, 4.6, 2.3 Hz, 1H), 0.46 (dtd, *J* = 13.4, 4.6, 2.4 Hz, 1H).

**^13^C NMR (126 MHz, CDCl_3_)** δ 145.3, 142.5, 140.6, 139.2, 131.0, 130.7, 130.1, 127.6, 127.3, 127.0, 126.60, 126.57, 126.2, 117.2, 68.2, 57.2, 55.6, 47.9, 34.8, 34.7, 31.5.

**HRMS (pos. APCI)** m/z: [M+H]^+^ Calcd for C_28_H_31_O 383.2369. Found 383.2373.

(4-40)

##### (*E*)-3-(6-methyl-4,4-diphenylhept-1-en-1-yl)oxazolidin-2-one (4-40*L-E*)

([See spectra](#_1H_NMR_Spectrum_39))

##### 3-(6-methyl-4,4-diphenylhept-1-en-3-yl)oxazolidin-2-one (4-40*B*)

([See spectra](#_1H_NMR_Spectrum_40))

The reaction was performed following the ***GP 3***. The crude mixture was first roughly purified though column chromatography on silica gel (PE:EtOAc = 4:1) to get rid of the majority of nonpolar side products. The residue was then carefully purified by a reverse phase column chromatography on C18 to afford both isomers in pure form (Combined yield: 48.8 mg, 70%). The *L:B* selectivity was determined to be 2.1:1 and the *E:Z* selectivity for the linear product was determined to be over 20:1 based on crude ^1^H NMR (*L:B* = 1.3:1)(*E:Z* > 20:1).

**Characterization data for 4-40*L-E*** (Colorless oil, 33.4 mg, 48%)

**TLC** (SiO_2_): R*_f_* = 0.33 (PE:EtOAc = 4:1)

**^1^H NMR (400 MHz, CDCl_3_)** δ 7.27 – 7.21 (m, 4H), 7.18 – 7.13 (m, 6H), 6.57 (dt, *J* = 14.4, 1.3 Hz, 1H), 4.35 – 4.29 (m, 2H), 4.23 (dt, *J* = 14.5, 7.3 Hz, 1H), 3.48 – 3.39 (m, 2H), 2.91 (dd, *J* = 7.3, 1.3 Hz, 2H), 1.99 (d, *J* = 5.1 Hz, 2H), 1.56 – 1.45 (m, 1H), 0.60 (d, *J* = 6.7 Hz, 6H).

**^13^C NMR (101 MHz, CDCl_3_)** δ 155.3, 148.4, 128.2, 127.8, 125.85, 125.80, 107.3, 62.0, 50.1, 46.4, 42.5, 39.3, 24.9, 24.2.

**HRMS (pos. APCI)** m/z: [M+H]^+^ Calcd for C_23_H_28_NO_2_ 350.2115. Found 350.2117.

**Characterization data for 4-40*B*** (White solid, 15.4 mg, 22%)

**TLC** (SiO_2_): R*_f_* = 0.18 (PE:EtOAc = 4:1)

**^1^H NMR (400 MHz, CDCl_3_)** δ 7.47 – 7.41 (m, 2H), 7.37 – 7.32 (m, 2H), 7.32 – 7.23 (m, 5H), 7.23 – 7.18 (m, 1H), 5.87 (ddd, *J* = 17.2, 10.4, 7.9 Hz, 1H), 5.42 (dt, *J* = 8.0, 1.1 Hz, 1H), 5.32 (dt, *J* = 17.2, 1.2 Hz, 1H), 5.31 (dt, *J* = 10.4, 1.1 Hz, 1H), 4.01 (td, *J* = 8.6, 3.2 Hz, 1H), 3.91 (dt, *J* = 10.5, 8.2 Hz, 1H), 2.85 (dt, *J* = 10.5, 8.8 Hz, 1H), 2.30 (dd, *J* = 14.4, 3.8 Hz, 1H), 1.88 (dd, *J* = 14.4, 5.9 Hz, 1H), 1.74 (td, *J* = 8.4, 3.2 Hz, 1H), 1.54 – 1.43 (m, 1H), 0.67 (d, *J* = 6.7 Hz, 3H), 0.24 (d, *J* = 6.5 Hz, 3H).

**^13^C NMR (101 MHz, CDCl_3_)** δ 159.7, 143.9, 142.7, 132.9, 130.5, 130.2, 127.8, 127.7, 127.0, 126.4, 121.1, 63.0, 62.4, 55.8, 48.3, 42.0, 25.0, 24.3, 24.1.

**HRMS (pos. ESI)** m/z: [M+H]^+^ Calcd for C_23_H_28_O_2_N 350.2115. Found 350.2117.

**Melting point:** 165-167 °C

##### isopropyl 2-(4-(2-(4-chlorophenyl)-1-(tetrahydro-2*H*-pyran-4-yl)pent-4-en-2-yl)phenoxy)-2-methylpropanoate (4-41)

([See spectra](#_1H_NMR_Spectrum_41))

The reaction was performed following the ***GP 3***. The crude mixture was first roughly purified though column chromatography on silica gel (PE:EtOAc = 10:1) to get rid of the majority of side products. The residue was then carefully purified by a reverse phase column chromatography on C18 to afford the title compound as a white solid (84.9 mg, 88%).

**TLC** (SiO_2_): R*_f_* = 0.25 (PE:EtOAc = 10:1)

**^1^H NMR (400 MHz, CDCl_3_)** δ 7.23 – 7.18 (m, 2H), 7.11 – 7.06 (m, 2H), 7.02 – 6.96 (m, 2H), 6.77 – 6.71 (m, 2H), 5.35 – 5.23 (m, 1H), 5.07 (hept, *J* = 6.3 Hz, 1H), 4.97 – 4.88 (m, 2H), 3.79 – 3.66 (m, 2H), 3.17 (td, *J* = 11.8, 2.6 Hz, 1H), 3.14 (td, *J* = 11.6, 2.3 Hz, 1H), 2.90 – 2.77 (m, 2H), 1.98 (dd, *J* = 14.1, 4.8 Hz, 1H), 1.94 (dd, *J* = 14.0, 4.9 Hz, 1H)., 1.56 (s, 6H), 1.46 – 1.34 (m, 1H), 1.23 – 1.07 (m, 8H), 1.04 (ddq, *J* = 13.6, 4.5, 2.3 Hz, 1H), 0.94 (ddq, *J* = 13.3, 4.4, 2.3 Hz, 1H).

**^13^C NMR (101 MHz, CDCl_3_)** δ 173.7, 153.7, 147.1, 141.2, 134.6, 131.6, 129.4, 128.6, 127.9, 118.5, 118.0, 79.1, 68.9, 68.10, 68.07, 48.6, 44.6, 43.2, 35.0, 34.9, 31.2, 25.4, 21.61, 21.62.

**HRMS (pos. APCI)** m/z: [M+H]^+^ Calcd for C_29_H_38_O_4_^35^Cl 485.2453. Found 485.2453.

**Melting point:** 93-95 °C

##### isopropyl 2-(4-(4-(1-((*tert*-butoxycarbonyl)(methyl)amino)hex-5-en-3-yl)benzoyl)phenoxy)-2-methylpropanoate (4-42)

([See spectra](#_1H_NMR_Spectrum_42))

The reaction was performed following the ***GP 4***. The crude mixture was first roughly purified though column chromatography on silica gel (PE:EtOAc = 5:1) to get rid of the majority of side products. The residue was then carefully purified by a reverse phase column chromatography on C18 to afford the title compound as a colorless oil (20.3 mg, 19%).

**TLC** (SiO_2_): R*_f_* = 0.21 (PE:EtOAc = 5:1)

**^1^H NMR (500 MHz, CDCl_3_,** **333 K)** δ 7.76 – 7.72 (m, 2H), 7.72 – 7.67 (m, 2H), 7.27 – 7.23 (m, 2H), 6.91 – 6.85 (m, 2H), 5.66 (ddt, *J* = 17.2, 10.2, 7.0 Hz, 1H), 5.07 (hept, *J* = 6.3 Hz, 1H), 5.00 – 4.94 (m, 2H), 3.21 – 3.02 (m, 2H), 2.80 – 2.66 (m, 4H), 2.47 – 2.35 (m, 2H), 2.00 – 1.92 (m, 1H), 1.89 – 1.79 (m, 1H), 1.65 (s, 6H), 1.42 (s, 9H), 1.20 (d, *J* = 6.3 Hz, 6H).

**^13^C NMR (126 MHz, CDCl_3_, 333 K)** δ 195.1, 173.2, 159.7, 155.8, 149.3, 136.6, 136.2, 131.9, 131.4, 130.2, 127.6, 117.8, 116.7, 79.7, 79.4, 69.3, 47.5, 43.8, 41.2, 34.3, 33.6, 28.6, 25.6, 21.6.

**HRMS (pos. APCI)** m/z: [M+H]^+^ Calcd for C_32_H_44_O_6_N 538.3163. Found 538.3156.

##### 2-(4-(6-methyl-4-phenylhept-1-en-4-yl)phenyl)propanoic acid (4-43)

([See spectra](#_1H_NMR_Spectrum_43))

The reaction was performed following a slightly modified ***GP 3*** using (η^3^-cinnamyl)PdCp instead of Pd(OAc)_2_. The crude mixture was first roughly purified though column chromatography on silica gel (PE:EtOAc = 4:1, acidified with 1 mol% HCO_2_H) to get rid of the majority of side products. The residue was then carefully purified by a reverse phase column chromatography on C18 to afford the title compound as a yellowish oil (52.5 mg, 78%). The diasteroselecctivty was determined to be 1:1 based on both ^1^H NMR and ^13^C NMR.

**TLC** (SiO_2_): R*_f_* = 0.29 (PE:EtOAc = 4:1, acidified with 1 mol% HCO_2_H)

**^1^H NMR (400 MHz, CDCl_3_)** δ 7.25 – 7.09 (m, 8H), 7.07 – 7.03 (m, 1H), 5.33 (ddt, *J* = 17.2, 10.1, 7.0 Hz, 1H) (*Set of isomer pick 1*), 5.33 (ddt, *J* = 17.2, 10.1, 7.0 Hz, 1H) (*Set of isomer pick 1*), 4.98 – 4.86 (m, 2H), 3.66 (q, *J* = 7.2 Hz, 1H), 2.90 (dt, *J* = 7.0, 1.3 Hz, 2H), 2.06 – 1.94 (m, 2H), 1.56 – 1.48 (m, 1H), 1.46 (d, *J* = 7.2 Hz, 3H) (*Set of isomer pick 2*), 1.45 (d, *J* = 7.2 Hz, 3H) (*Set of isomer pick 2*), 0.61 (d, *J* = 6.6 Hz, 3H) (*Set of isomer pick 3*), 0.61 (d, *J* = 6.7 Hz, 3H) (*Set of isomer pick 3*), 0.57 (d, *J* = 6.7 Hz, 3H) (*Set of isomer pick 4*), 0.56 (d, *J* = 6.7 Hz, 3H) (*Set of isomer pick 4*).

**^13^C NMR (101 MHz, CDCl_3_)** δ 180.6, 149.13 (*Set of isomer pick 1*), 149.11 (*Set of isomer pick 1*), 148.4, 139.3 (*Set of isomer pick 2*), 139.2 (*Set of isomer pick 2*), 135.1, 128.3, 128.1, 127.89 (*Set of isomer pick 3*), 127.85 (*Set of isomer pick 3*), 127.78, 127.5, 125.8, 124.7 (*Set of isomer pick 4*), 124.6 (*Set of isomer pick 4*), 117.6, 49.8, 46.4, 45.6, 43.19 (*Set of isomer pick 5*), 43.16 (*Set of isomer pick 5*), 24.9, 24.8, 24.2, 18.34 (*Set of isomer pick 6*), 18.26 (*Set of isomer pick 6*).

**HRMS (neg. ESI)** m/z: [M-H]^-^ Calcd for C_23_H_27_O_2_ 335.2017. Found 335.2010.

##### 2-(11-allyl-11-isobutyl-6,11-dihydrodibenzo[*b,e*]oxepin-2-yl)acetic acid (4-44)

([See spectra](#_1H_NMR_Spectrum_44))

The reaction was performed following the ***GP 3***. The crude mixture was first roughly purified though column chromatography on silica gel (PE:EtOAc = 4:1, acidified with 1 mol% HCO_2_H) to get rid of the majority of side products. The residue was then carefully purified by a reverse phase column chromatography on C18 to afford the title compound as a white solid (51.4 mg, 73%).

**TLC** (SiO_2_): R*_f_* = 0.25 (PE:EtOAc = 4:1, acidified with 1 mol% HCO_2_H)

**^1^H NMR (400 MHz, CDCl_3_)** δ 7.57 (dd, *J* = 8.3, 1.1 Hz, 1H), 7.44 (d, *J* = 2.1 Hz, 1H), 7.29 (ddd, *J* = 8.2, 7.2, 1.6 Hz, 1H), 7.14 (td, *J* = 7.4, 1.1 Hz, 1H), 7.03 (dd, *J* = 7.5, 1.6 Hz, 1H), 7.02 (dd, *J* = 8.1, 2.1 Hz, 1H), 6.91 (d, *J* = 8.1 Hz, 1H), 5.39 (ddt, *J* = 17.2, 10.2, 6.9 Hz, 1H), 5.01 (d, *J* = 13.6 Hz, 1H), 4.97 (d, *J* = 13.6 Hz, 1H), 4.76 (ddt, *J* = 10.2, 2.2, 1.1 Hz, 1H), 4.68 (ddt, *J* = 17.1, 2.2, 1.3 Hz, 1H), 3.61 (s, 2H), 2.95 (ddt, *J* = 14.4, 7.2, 1.2 Hz, 1H), 2.82 (ddt, *J* = 14.4, 6.7, 1.3 Hz, 1H), 2.29 (dd, *J* = 14.3, 5.8 Hz, 1H), 2.06 (dd, *J* = 14.4, 5.6 Hz, 1H), 1.43 – 1.29 (m, 1H), 0.51 (d, *J* = 6.7 Hz, 3H), 0.49 (d, *J* = 6.7 Hz, 3H).

**^13^C NMR (101 MHz, CDCl_3_)** δ 178.0, 161.3, 143.8, 138.8, 135.5, 134.4, 132.0, 129.5, 128.1, 127.93, 127.87, 127.7, 125.9, 121.3, 117.5, 77.0, 55.1, 53.6, 51.4, 40.8, 25.5, 24.1, 24.0.

**HRMS (pos. APCI)** m/z: [M+H]^+^ Calcd for C_23_H_27_O_3_ 351.1955. Found 351.1951.

**Melting point:** 105-107 °C.

#### Dienylic products

##### (2,7-dimethyl-6-methyleneoct-7-ene-4,4-diyl)dibenzene (5-1)

([See spectra](#_1H_NMR_Spectrum_45))

The reaction was performed following a slightly modified ***GP 5*** using 1.2 equiv. DHP, 1.0 mol% [Ir(ppy)_2_dtbbpy]PF_6_, 10 mol% Pd(OAc)_2_ and 24 mol% (2-furyl)_3_P. The crude mixture was first roughly purified though column chromatography on silica gel (PE) to get rid of the majority of polar side products. The residue was then carefully purified by a reverse phase column chromatography on C18 to afford the title compound as a colorless oil (30.1 mg, 50%).

**TLC** (SiO_2_): R*_f_* = 0.63 (PE)

**^1^H NMR (500 MHz, CDCl_3_)** δ 7.22 – 7.16 (m, 8H), 7.16 – 7.11 (m, 2H), 4.99 (d, *J* = 1.7 Hz, 1H), 4.77 – 4.73 (m, 1H), 4.62 – 4.59 (m, 1H), 4.44 – 4.40 (m, 1H), 3.14 (d, *J* = 0.9 Hz, 2H), 2.02 (d, *J* = 5.2 Hz, 2H), 1.65 (dd, *J* = 1.3, 0.6 Hz, 3H), 1.58 – 1.50 (m, 1H), 0.51 (d, *J* = 6.7 Hz, 6H).

**^13^C NMR (126 MHz, CDCl_3_)** δ 148.9, 145.8, 144.8, 128.9, 127.5, 125.7, 115.9, 112.2, 50.5, 46.4, 41.2, 24.7, 24.5, 21.6.

**HRMS (pos. APCI)** m/z: [M+H]^+^ Calcd for C_23_H_29_ 305.2264. Found 305.2271.

##### benzyl 4-(5-methyl-4-methylene-2,2-diphenylhex-5-en-1-yl)piperidine-1-carboxylate (5-3)

([See spectra](#_1H_NMR_Spectrum_46))

The reaction was performed following the ***GP 5***. The crude mixture was first roughly purified though column chromatography on silica gel (PE:EtOAc = 10:1) to get rid of the majority of side products. The residue was then carefully purified by a reverse phase column chromatography on C18 to afford the title compound as a colorless oil (54.0 mg, 56%).

**TLC** (SiO_2_): R*_f_* = 0.22 (PE:EtOAc = 10:1)

**^1^H NMR (400 MHz, CDCl_3_)** δ 7.37 – 7.23 (m, 5H), 7.25 – 7.09 (m, 10H), 5.05 (s, 2H), 4.98 (d, *J* = 1.5 Hz, 1H), 4.79 – 4.74 (m, 1H), 4.64 – 4.60 (m, 1H), 4.36 – 4.32 (m, 1H)., 3.19 – 3.10 (m, 2H), 2.61 – 2.43 (m, 2H), 2.01 (br, s, 2H), 1.65 (dd, *J* = 1.4, 0.9 Hz, 3H), 1.45 – 1.30 (m, 1H), 0.88 (br, s, 4H).

**^13^C NMR (101 MHz, CDCl_3_)** δ 155.2, 148.4, 145.5, 144.8, 137.1, 128.7, 128.5, 127.9, 127.8, 127.7, 125.9, 116.1, 112.3, 66.9, 50.4, 44.3, 44.1, 41.4, 33.6, 32.5, 21.6.

**HRMS (pos. ESI)** m/z: [M+H]^+^ Calcd for C_33_H_38_O_2_N 480.2897. Found 480.2896.

##### 5-(2,7-dimethyl-6-methylene-4,4-diphenyloct-7-en-1-yl)benzo[*d*][1,3]dioxole (5-4)

([See spectra](#_1H_NMR_Spectrum_47)) ([See gram scale reaction](#_Dienylation))

The reaction was performed following a slightly modified ***GP 5*** using 4.0 equiv. of diphenylethylene. The crude mixture was first roughly purified though column chromatography on silica gel (PE:EtOAc = 40:1) to get rid of the majority of polar side products. The residue was then carefully purified by a reverse phase column chromatography on C18 to afford the title compound as a colorless oil (49.9 mg, 59%).

**TLC** (SiO_2_): R*_f_* = 0.30 (PE:EtOAc = 40:1)

**^1^H NMR (500 MHz, CDCl_3_)** δ 7.26 – 7.21 (m, 4H), 7.21 – 7.11 (m, 6H), 6.66 – 6.61 (m, 1H), 6.39 – 6.34 (m, 2H), 5.87 (d, *J* = 1.5 Hz, 1H), 5.87 (d, *J* = 1.5 Hz, 1H), 4.98 (d, *J* = 1.6 Hz, 1H), 4.80 – 4.78 (m, 1H), 4.67 – 4.62 (m, 1H), 4.43 – 4.39 (m, 1H), 3.17 (s, 2H), 2.16 (dd, *J* = 14.0, 4.5 Hz, 1H), 2.08 (dd, *J* = 13.2, 4.7 Hz, 1H), 2.05 (dd, *J* = 14.0, 5.5 Hz, 1H), 1.90 (dd, *J* = 13.3, 9.7 Hz, 1H), 1.66 (dd, *J* = 1.3, 0.7 Hz, 3H), 1.63 – 1.54 (m, 1H), 0.44 (d, *J* = 6.6 Hz, 3H).

**^13^C NMR (126 MHz, CDCl_3_)** δ 148.5, 148.5, 147.3, 145.5, 145.4, 144.9, 135.5, 129.0, 129.0, 127.7, 127.6, 125.9, 125.8, 122.1, 116.0, 112.3, 109.6, 107.8, 100.7, 50.7, 44.9, 44.8, 41.6, 31.7, 21.7, 21.0.

**HRMS (pos. APCI)** m/z: [M+H]^+^ Calcd for C_30_H_33_O_2_ 425.2475. Found 425.2469.

##### (2,7,11-trimethyl-3-methylenedodeca-1,10-diene-5,5-diyl)dibenzene (5-5)

([See spectra](#_1H_NMR_Spectrum_48))

The reaction was performed following the ***GP 5***. The crude mixture was first roughly purified though column chromatography on silica gel (PE) to get rid of the majority of polar side products. The residue was then carefully purified by a reverse phase column chromatography on C18 to afford the title compound as a colorless oil (22.5 mg, 30%).

**TLC** (SiO_2_): R*_f_* = 0.41 (PE)

**^1^H NMR (400 MHz, CDCl_3_)** δ 7.23 – 7.17 (m, 8H), 7.17 – 7.10 (m, 2H), 4.99 (d, *J* = 1.6 Hz, 1H), 4.93 – 4.84 (m, 1H), 4.80 – 4.75 (m, 1H), 4.65 – 4.60 (m, 1H), 4.46 – 4.42 (m, 1H), 3.16 (dd, *J* = 14.1, 1.2 Hz, 1H), 3.12 (dd, *J* = 14.0, 1.4 Hz, 1H), 2.12 (dd, *J* = 13.9, 4.2 Hz, 1H), 1.96 (dd, *J* = 13.9, 6.0 Hz, 1H), 1.88 – 1.67 (m, 2H), 1.65 (dd, *J* = 1.3, 0.6 Hz, 3H), 1.64 (q, *J* = 1.3 Hz, 3H), 1.52 (d, *J* = 1.3 Hz, 3H), 1.45 – 1.34 (m, 1H), 0.96 – 0.87 (m, 2H), 0.45 (d, *J* = 6.6 Hz, 3H).

**^13^C NMR (101 MHz, CDCl_3_)** δ 148.83, 148.78, 145.7, 144.9, 130.9, 128.92, 128.90, 127.5, 125.7, 125.0, 115.8, 112.2, 50.6, 45.5, 41.3, 38.9, 28.8, 25.8, 25.5, 21.6, 20.9, 17.7.

**HRMS (pos. APCI)** m/z: [M+H]^+^ Calcd for C_28_H_37_ 373.2890. Found 373.2896.

##### 4,4'-(5-methyl-4-methylene-1-(4-(4-methylpent-3-en-1-yl)cyclohex-3-en-1-yl)hex-5-ene-2,2-diyl)bis(methoxybenzene) (5-6)

([See spectra](#_1H_NMR_Spectrum_49))

The reaction was performed following the ***GP 5***. The crude mixture was first roughly purified though column chromatography on silica gel (PE:EtOAc = 40:1) to get rid of the majority of polar side products. The residue was then carefully purified by a reverse phase column chromatography on C18 to afford the title compound as a colorless oil (37.1 mg, 38%). The NMR spectra showed two set of picks with a ratio of 2.6:1. The formation of atropisomers was proposed to rationalize the observed phenomena according to the precedent example (See 4-6).

**TLC** (SiO_2_): R*_f_* = 0.30 (PE:EtOAc = 40:1)

**^1^H NMR (500 MHz, CDCl_3_)** δ 7.13 – 7.05 (m, 4H), 6.78 – 6.71 (m, 4H), 5.24 (dq, *J* = 3.3, 1.7 Hz, 1H) (*Set of isomer peak 1*), 5.15 (qd, *J* = 2.6, 1.2 Hz, 1H) (*Set of isomer peak 1*), 5.08 – 5.01 (m, 1H), 4.99 (d, *J* = 1.7 Hz, 1H), 4.78 – 4.77 (m, 1H) (*Set of isomer peak 2*), 4.77 – 4.75 (m, 1H) (*Set of isomer peak 2*), 4.66 – 4.61 (m, 1H), 4.42 – 4.39 (m, 1H) (*Set of isomer peak 3*), 4.39 – 4.37 (m, 1H) (*Set of isomer peak 3*), 3.79 (s, 3H) (*Set of isomer peak 4*), 3.78 (s, 3H) (*Set of isomer peak 5*), 3.78 (s, 3H) (*Set of isomer peak 4*), 3.77 (s, 3H) (*Set of isomer peak 5*), 3.12 (d, *J* = 13.4 Hz, 1H) (*Set of isomer peak 6*), 3.12 (dd, *J* = 13.4, 0.6 Hz, 1H) (*Set of isomer peak 6*), 3.05 (d, *J* = 13.3 Hz, 1H) (*Set of isomer peak 7*), 3.04 (dd, *J* = 13.4, 0.8 Hz, 1H) (*Set of isomer peak 7*), 2.05 – 1.80 (m, 6H), 1.76 – 1.70 (m, 2H), 1.69 – 1.63 (m, 6H), 1.59 – 1.56 (m, 3H), 1.52 – 1.27 (m, 3H), 1.10 – 1.02 (m, 1H), 1.02 – 0.86 (m, 1H).

**^13^C NMR (126 MHz, CDCl_3_)** **(Major)** δ 157.53, 157.49, 145.84, 144.81, 141.20, 141.11, 137.35, 129.74, 129.69, 124.59, 120.68, 115.94, 112.81, 112.78, 112.21, 55.27, 49.29, 44.77, 41.76, 37.60, 33.82, 30.94, 30.09, 28.81, 26.52, 25.77, 21.67, 17.74.

**^13^C NMR (126 MHz, CDCl_3_)** **(Minor)** δ 157.61, 157.53, 145.82, 144.85, 141.16, 137.73, 131.28, 131.23, 124.56, 120.26, 115.96, 112.86, 112.79, 112.23, 55.27, 49.35, 44.86, 41.70, 37.92, 36.79, 30.55, 30.45, 28.81, 26.50, 25.56, 21.69, 17.72.

**HRMS (pos. APCI)** m/z: [M+H]^+^ Calcd for C_34_H_45_O_2_ 485.3414. Found 485.3413.

##### *tert*-butyldimethyl((6-methyl-5-methylene-3,3-diphenylhept-6-en-1-yl)oxy)silane (5-7)

([See spectra](#_1H_NMR_Spectrum_50))

The reaction was performed following a slightly modified ***GP 5*** with 100 mg 3 Å MS as additive. The crude mixture was first roughly purified though column chromatography on silica gel (PE) to get rid of the majority of polar side products. The residue was then carefully purified by a reverse phase column chromatography on C18 to afford the title compound as a colorless oil (37.1 mg, 38%).

**TLC** (SiO_2_): R*_f_* = 0.30 (PE)

**^1^H NMR (400 MHz, CDCl_3_)** δ 7.23 – 7.18 (m, 4H), 7.17 – 7.11 (m, 6H), 5.08 – 5.03 (m, 1H), 4.70 (dq, *J* = 1.3, 0.6 Hz, 1H), 4.60 – 4.55 (m, 2H), 3.36 – 3.28 (m, 2H), 3.10 (d, *J* = 0.8 Hz, 2H), 2.44 – 2.36 (m, 2H), 1.65 (dd, *J* = 1.4, 0.6 Hz, 3H), 0.81 (s, 9H), -0.09 (s, 6H).

**^13^C NMR (101 MHz, CDCl_3_)** δ 148.3, 145.4, 144.4, 128.4, 127.7, 125.8, 116.2, 112.3, 60.6, 48.9, 41.1, 39.9, 26.1, 21.6, 18.3, -5.2.

**HRMS (pos. APCI)** m/z: [M+H]^+^ Calcd for C_27_H_39_OSi 407.2765. Found 407.2764.

##### (1,1-dimethoxy-6-methyl-5-methylenehept-6-ene-3,3-diyl)dibenzene (5-8)

([See spectra](#_1H_NMR_Spectrum_51))

The reaction was performed following a slightly modified ***GP 5*** using 1.0 mol% [Ir(ppy)_2_dtbbpy]PF_6_, 10 mol% Pd(OAc)_2_ and 24 mol% (2-furyl)_3_P. The crude mixture was first roughly purified though column chromatography on silica gel (PE:EtOAc = 20:1) to get rid of the majority of polar side products. The residue was then carefully purified by a reverse phase column chromatography on C18 to afford the title compound as a colorless oil (33.8 mg, 50%).

**TLC** (SiO_2_): R*_f_* = 0.35 (PE:EtOAc = 20:1)

**^1^H NMR (400 MHz, CDCl_3_)** δ 7.26 – 7.17 (m, 4H), 7.19 – 7.10 (m, 6H), 5.09 (d, *J* = 1.9 Hz, 1H), 4.81 – 4.76 (m, 1H), 4.63 (dq, *J* = 1.3, 0.6 Hz, 1H), 4.53 – 4.48 (m, 1H), 3.85 (t, *J* = 5.0 Hz, 1H), 3.20 (d, *J* = 0.8 Hz, 2H), 3.10 (s, 6H), 2.50 (d, *J* = 5.1 Hz, 2H), 1.63 (dd, *J* = 1.3, 0.6 Hz, 3H).

**^13^C NMR (101 MHz, CDCl_3_)** δ 148.3, 145.7, 144.3, 128.6, 127.6, 125.9, 116.9, 112.3, 104.1, 53.2, 48.6, 40.9, 40.4, 21.5.

**HRMS (pos. APCI)** m/z: [M+NH_4_]^+^ Calcd for C_23_H_32_O_2_N 354.2428. Found 354.2432.

##### *tert*-butyl methyl(6-methyl-5-methylene-3,3-diphenylhept-6-en-1-yl)carbamate (5-9)

([See spectra](#_1H_NMR_Spectrum_52))

The reaction was performed following a slightly modified ***GP 5*** using 1.0 mol% [Ir(ppy)_2_dtbbpy]PF_6_, 10 mol% Pd(OAc)_2_ and 24 mol% (2-furyl)_3_P. The crude mixture was first roughly purified though column chromatography on silica gel (PE:EtOAc = 20:1) to get rid of the majority of polar side products. The residue was then carefully purified by a reverse phase column chromatography on C18 to afford the title compound as a colorless oil (48.5 mg, 60%).

**TLC** (SiO_2_): R*_f_* = 0.32 (PE:EtOAc = 20:1)

**^1^H NMR (500 MHz, CDCl_3_, 333 K)** δ 7.23 – 7.19 (m, 4H), 7.19 – 7.12 (m, 6H), 5.04 (d, *J* = 1.6 Hz, 1H), 4.72 (s, 1H), 4.65 – 4.46 (m, 2H), 3.09 (d, *J* = 0.8 Hz, 2H), 2.94 – 2.87 (m, 2H), 2.67 (s, 3H), 2.36 – 2.29 (m, 2H), 1.65 (d, *J* = 1.3 Hz, 3H), 1.40 (s, 9H).

**^13^C NMR (126 MHz, CDCl_3_, 333 K)** δ 155.7, 147.9, 145.7, 144.8, 128.5, 127.8, 126.0, 116.2, 112.4, 79.3, 49.3, 45.7, 41.4, 35.3, 34.1, 28.7, 21.5.

**HRMS (pos. ESI)** m/z: [M+Na]^+^ Calcd for C_27_H_35_O_2_NNa 428.2560. Found 428.2565.

##### ethyl allyl(6-methyl-5-methylene-3,3-diphenylhept-6-en-1-yl)carbamate (5-10)

([See spectra](#_1H_NMR_Spectrum_67))

The reaction was performed following a slightly modified ***GP 5*** using 1.0 mol% [Ir(ppy)_2_dtbbpy]PF_6_, 10 mol% Pd(OAc)_2_ and 24 mol% (2-furyl)_3_P. The crude mixture was first roughly purified though column chromatography on silica gel (PE:EtOAc = 10:1) to get rid of the majority of side products. The residue was then carefully purified by a reverse phase column chromatography on C18 to afford the title compound as a colorless oil (49.8 mg, 62%).

**TLC** (SiO_2_): R*_f_* = 0.28 (PE:EtOAc = 10:1)

**^1^H NMR (500 MHz, CDCl_3_, 333 K)** δ 7.26 – 7.21 (m, 4H), 7.21 – 7.14 (m, 6H), 5.65 (ddt, *J* = 17.1, 10.2, 5.9 Hz, 1H), 5.07 (d, *J* = 1.6 Hz, 1H), 5.00 (dq, *J* = 10.2, 1.4 Hz, 1H), 4.91 (dq, *J* = 17.1, 1.5 Hz, 1H), 4.74 – 4.70 (m, 1H), 4.68 – 4.54 (m, 2H), 4.11 (q, *J* = 7.1 Hz, 2H), 3.76 – 3.58 (m, 2H), 3.10 (d, *J* = 0.8 Hz, 2H), 2.98 – 2.92 (m, 2H), 2.41 – 2.34 (m, 2H), 1.66 (dd, *J* = 1.5, 0.7 Hz, 3H), 1.23 (t, *J* = 7.1 Hz, 3H).

**^13^C NMR (126 MHz, CDCl_3_, 333 K)** δ 156.3, 147.9, 145.7, 144.8, 134.2, 128.5, 127.8, 126.0, 116.6, 116.1, 112.4, 61.2, 49.7, 49.4, 43.5, 41.2, 35.7, 21.5, 14.8.

**HRMS (pos. ESI)** m/z: [M+Na]^+^ Calcd for C_27_H_33_O_2_NNa 426.2404. Found 426.2403.

##### 1-(6-methyl-5-methylene-3,3-diphenylhept-6-en-1-yl)-1*H*-indole (5-11)

([See spectra](#_1H_NMR_Spectrum_53))

The reaction was performed following a slightly modified ***GP 5*** using 1.0 mol% [Ir(ppy)_2_dtbbpy]PF_6_, 10 mol% Pd(OAc)_2_ and 24 mol% (2-furyl)_3_P. The crude mixture was first roughly purified though column chromatography on silica gel (PE) to get rid of the majority of polar side products. The residue was then carefully purified by a reverse phase column chromatography on C18 to afford the title compound as a white solid (22.9 mg, 29%).

**TLC** (SiO_2_): R*_f_* = 0.34 (PE)

**^1^H NMR (500 MHz, CDCl_3_)** δ 7.58 (dt, *J* = 7.8, 1.0 Hz, 1H), 7.32 – 7.24 (m, 4H), 7.24 – 7.17 (m, 6H), 7.10 (ddd, *J* = 8.3, 7.0, 1.3 Hz, 1H), 7.05 (ddd, *J* = 8.0, 7.0, 1.1 Hz, 1H), 6.95 (dd, *J* = 8.2, 1.0 Hz, 1H), 6.92 (d, *J* = 3.1 Hz, 1H), 6.44 (dd, *J* = 3.1, 0.9 Hz, 1H), 5.06 (d, *J* = 1.4 Hz, 1H), 4.92 – 4.88 (m, 1H), 4.79 – 4.74 (m, 1H), 4.42 – 4.38 (m, 1H), 3.85 – 3.78 (m, 2H), 3.22 (d, *J* = 0.8 Hz, 2H), 2.61 – 2.54 (m, 2H), 1.75 (d, *J* = 1.1 Hz, 3H).

**^13^C NMR (126 MHz, CDCl_3_)** δ 147.8, 145.1, 144.8, 135.9, 128.6, 128.2, 128.1, 127.4, 126.3, 121.3, 121.0, 119.2, 116.3, 112.6, 109.2, 101.2, 49.3, 43.0, 40.3, 37.6, 21.8.

**HRMS (pos. APCI)** m/z: [M+H]^+^ Calcd for C_29_H_30_N 392.2373. Found 392.2380.

**Melting point:** 107-109 °C

##### 9-(6-methyl-5-methylene-3,3-diphenylhept-6-en-1-yl)-9*H*-carbazole (5-12)

([See spectra](#_1H_NMR_Spectrum_54)) ([See crystal structure](#_Dienylic_product_5-12))

The reaction was performed following a slightly modified ***GP 5*** using 1.0 mol% [Ir(ppy)_2_dtbbpy]PF_6_, 10 mol% Pd(OAc)_2_ and 24 mol% (2-furyl)_3_P. The crude mixture was first roughly purified though column chromatography on silica gel (PE:EtOAc = 50:1) to get rid of the majority of polar side products. The residue was then carefully purified by a reverse phase column chromatography on C18 to afford the title compound as a white solid (33.7 mg, 38%).

**TLC** (SiO_2_): R*_f_* = 0.27 (PE:EtOAc = 50:1)

**^1^H NMR (400 MHz, CDCl_3_)** δ 8.04 (ddd, *J* = 7.8, 1.3, 0.7 Hz, 2H), 7.35 (ddd, *J* = 8.3, 7.2, 1.2 Hz, 2H), 7.32 – 7.26 (m, 4H), 7.26 – 7.20 (m, 6H), 7.17 (ddd, *J* = 8.0, 7.2, 0.9 Hz, 2H), 6.97 (dt, *J* = 8.2, 0.9 Hz, 2H), 5.13 (d, *J* = 1.5 Hz, 1H), 4.95 – 4.92 (m, 1H), 4.78 (p, *J* = 1.2 Hz, 1H), 4.60 – 4.56 (m, 1H), 4.07 – 3.98 (m, 2H), 3.29 (d, *J* = 0.9 Hz, 2H), 2.61 – 2.52 (m, 2H), 1.74 (dd, *J* = 1.4, 0.6 Hz, 3H).

**^13^C NMR (101 MHz, CDCl_3_)** δ 148.0, 145.3, 144.9, 140.2, 128.5, 128.1, 126.3, 125.5, 123.0, 120.3, 118.8, 116.2, 112.8, 108.6, 49.4, 40.3, 39.5, 35.8, 21.7.

**HRMS (pos. APCI)** m/z: [M+H]^+^ Calcd for C_33_H_32_N 442.2529. Found 442.2530.

**Melting point:** 136-138 °C

##### (6-methyl-5-methylenehept-6-ene-1,3,3-triyl)tribenzene (5-13)

([See spectra](#_1H_NMR_Spectrum_55))

The reaction was performed following the ***GP 5***. The crude mixture was first roughly purified though column chromatography on silica gel (PE) to get rid of the majority of polar side products. The residue was then carefully purified by a reverse phase column chromatography on C18 to afford the title compounds as a colorless oil (12.3 mg, 17%). The two regioisomers cannot be separated and the regioselectivity was determined to be 6.7:1 based on ^1^H NMR.

**TLC** (SiO_2_): R*_f_* = 0.18 (PE)

**^1^H NMR (500 MHz, CDCl_3_)** δ 7.27 – 7.00 (m, 18H) (Major + Minor), 6.56 – 6.52 (m, 0.31H) (Minor), 5.04 (d, *J* = 1.6 Hz, 1H), 5.02 (d, *J* = 1.4 Hz, 0.17H) (Minor), 4.91 (s, 0.15H) (Minor), 4.82 – 4.81 (m, 0.16H) (Minor), 4.81 – 4.78 (m, 1H), 4.68 – 4.66 (m, 0.16H) (Minor), 4.66 – 4.63 (m, 1H), 4.48 – 4.44 (m, 1H), 3.44 (s, 0.31H) (Minor), 3.19 (d, *J* = 0.8 Hz, 2H), 2.43 – 2.36 (m, 2H), 2.29 – 2.22 (m, 2H), 2.15 – 2.05 (m, 0.65H), 1.82 (d, *J* = 1.3 Hz, 0.48H), 1.69 (dd, *J* = 1.6, 0.7 Hz, 3H).

**^13^C NMR (126 MHz, CDCl_3_) (Major)** δ 148.6, 145.6, 144.6, 143.0, 128.5, 128.40, 128.37, 127.8, 125.8, 125.7, 115.9, 112.5, 50.4, 40.1, 39.6, 31.2, 21.7.

**^13^C NMR (126 MHz, CDCl_3_)** **(Minor)** δ 148.5, 148.3, 142.5, 137.9, 130.6, 127.8, 127.4, 126.1, 125.9, 112.8, 112.1, 50.9, 43.8, 36.3, 28.8, 21.2.

**HRMS (pos. APCI) (Major)** m/z: [M+H]^+^ Calcd for C_27_H_29_ 353.2264. Found 353.2269.

**HRMS (pos. APCI) (Minor)** m/z: [M+H]^+^ Calcd for C_27_H_29_ 353.2264. Found 353.2261.

##### 6-methyl-5-methylene-1,3,3-triphenylhept-6-en-1-one (5-14)

([See spectra](#_1H_NMR_Spectrum_97))

The reaction was performed following the ***GP 5***. The crude mixture was first roughly purified though column chromatography on silica gel (PE:EtOAc = 40:1) to get rid of the majority of polar side products. The residue was then carefully purified by a reverse phase column chromatography on C18 to afford the title compound as a white solid (20.3 mg, 28%).

**TLC** (SiO_2_): R*_f_* = 0.24 (PE:EtOAc = 40:1)

**^1^H NMR (400 MHz, CDCl_3_)** δ 7.80 – 7.75 (m, 2H), 7.49 – 7.43 (m, 1H), 7.38 – 7.32 (m, 2H), 7.24 – 7.16 (m, 8H), 7.13 – 7.08 (m, 2H), 5.02 (d, *J* = 1.8 Hz, 1H), 4.71 (dq, *J* = 1.3, 0.6 Hz, 1H), 4.52 – 4.48 (m, 1H), 4.48 – 4.43 (m, 1H), 3.93 (s, 2H), 3.53 (d, *J* = 0.7 Hz, 2H), 1.64 (dd, *J* = 1.4, 0.6 Hz, 3H).

**^13^C NMR (101 MHz, CDCl_3_)** δ 198.4, 148.5, 145.8, 144.2, 138.5, 132.5, 128.4, 128.2, 127.74, 127.71, 125.9, 116.8, 112.9, 48.8, 44.2, 40.5, 21.4.

**HRMS (pos. APCI)** m/z: [M+H]^+^ Calcd for C_27_H_27_O 367.2056. Found 367.2055.

**Melting point:** 74-76 °C

##### (4-methyl-3-methylenepent-4-ene-1,1-diyl)dibenzene (5-16)

([See spectra](#_1H_NMR_Spectrum_56))

The reaction was performed following the ***GP 5***. The crude mixture was first roughly purified though column chromatography on silica gel (PE) to get rid of the majority of polar side products. The residue was then carefully purified by a reverse phase column chromatography on C18 to afford the title compound as a colorless oil (9.6 mg, 19%).

**TLC** (SiO_2_): R*_f_* = 0.53 (PE)

**^1^H NMR (400 MHz, CDCl_3_)** δ 7.28 – 7.23 (m, 4H), 7.22 – 7.19 (m, 4H), 7.18 – 7.13 (m, 2H), 5.10 (dq, *J* = 1.3, 0.6 Hz, 1H), 5.02 – 4.99 (m, 1H), 4.99 – 4.96 (m, 1H), 4.69 – 4.65 (m, 1H), 4.22 (t, *J* = 7.6 Hz, 1H), 3.01 (dd, *J* = 7.6, 1.1 Hz, 2H), 1.86 (dd, *J* = 1.4, 0.6 Hz, 3H).

**^13^C NMR (101 MHz, CDCl_3_)** δ 145.5, 145.0, 142.8, 128.4, 128.1, 126.2, 114.6, 112.8, 49.8, 40.1, 21.4.

**HRMS (pos. APCI)** m/z: [M+H]^+^ Calcd for C_19_H_21_ 249.1638. Found 249.1636.

##### 4-(5-methyl-4-methylene-2-phenyl-2-(4-(p-tolylthio)phenyl)hex-5-en-1-yl)tetrahydro-2*H*-pyran (5-17)

([See spectra](#_1H_NMR_Spectrum_57))

The reaction was performed following a slightly modified ***GP 5*** with 100 mg 3 Å MS as additive. The crude mixture was first roughly purified though column chromatography on silica gel (PE:EtOAc = 10:1) to get rid of the majority of polar side products. The residue was then carefully purified by a reverse phase column chromatography on C18 to afford the title compound as a white solid (46.7 mg, 50%). The chemoselectivity between dienylation and allylation was determined to be 7.2:1 based on crude ^1^H NMR.

**TLC** (SiO_2_): R*_f_* = 0.36 (PE:EtOAc = 10:1)

**^1^H NMR (400 MHz, CDCl_3_)** δ 7.23 – 7.12 (m, 9H), 7.11 – 7.06 (m, 4H), 5.01 (d, *J* = 1.5 Hz, 1H), 4.76 – 4.71 (m, 1H), 4.64 – 4.58 (m, 1H), 4.43 – 4.38 (m, 1H), 3.71 (ddd, *J* = 11.7, 4.3, 2.1 Hz, 1H), 3.69 (ddd, *J* = 11.5, 4.4, 2.2 Hz, 1H), 3.20 – 3.05 (m, 4H), 2.32 (t, *J* = 0.7 Hz, 3H), 2.02 (dd, *J* = 14.0, 4.9 Hz, 1H), 1.98 (dd, *J* = 14.0, 5.0 Hz, 1H), 1.65 (dd, *J* = 1.4, 0.6 Hz, 3H), 1.51 – 1.36 (m, 1H), 1.16 – 0.97 (m, 2H), 0.88 (ddq, *J* = 13.3, 4.2, 2.3 Hz, 1H), 0.78 (ddq, *J* = 13.4, 4.6, 2.1 Hz, 1H).

**^13^C NMR (101 MHz, CDCl_3_)** δ 148.1, 147.6, 145.5, 144.6, 137.1, 133.5, 132.5, 131.1, 130.2, 130.0, 129.6, 128.7, 127.7, 126.0, 116.2, 112.5, 68.2, 50.2, 44.6, 41.3, 34.9, 34.8, 31.6, 21.6, 21.1.

**HRMS (pos. ESI)** m/z: [M+H]^+^ Calcd for C_32_H_37_OS 469.2560. Found 469.2564.

**Melting point:** 80-82 °C

##### 4-(5-methyl-4-methylene-2-phenyl-2-(4-(trifluoromethyl)phenyl)hex-5-en-1-yl)tetrahydro-2*H*-pyran (5-18)

([See spectra](#_1H_NMR_Spectrum_58))

The reaction was performed following the ***GP 5***. The crude mixture was first roughly purified though column chromatography on silica gel (PE:EtOAc = 10:1) to get rid of the majority of polar side products. The residue was then carefully purified by a reverse phase column chromatography on C18 to afford the title compound as a colorless oil (43.8 mg, 53%).

**TLC** (SiO_2_): R*_f_* = 0.18 (PE:EtOAc = 10:1)

**^1^H NMR (500 MHz, CDCl_3_)** δ 7.48 – 7.44 (m, 2H), 7.30 – 7.26 (m, 2H), 7.26 – 7.21 (m, 2H), 7.20 – 7.14 (m, 3H), 5.02 (d, *J* = 1.5 Hz, 1H), 4.74 – 4.70 (m, 1H), 4.62 – 4.58 (m, 1H), 4.41 – 4.38 (m, 1H), 3.73 (ddd, *J* = 11.5, 4.5, 2.0 Hz, 1H), 3.68 (ddd, *J* = 11.4, 4.3, 1.8 Hz, 1H), 3.21 (dd, *J* = 13.4, 0.8 Hz, 1H), 3.18 – 3.07 (m, 3H), 2.09 (dd, *J* = 14.0, 4.7 Hz, 1H), 2.03 (dd, *J* = 14.0, 5.5 Hz, 1H), 1.64 (dd, *J* = 1.5, 0.6 Hz, 3H), 1.50 – 1.40 (m, 1H), 1.13 (dtd, *J* = 13.3, 11.7, 4.3 Hz, 1H), 1.04 (dtd, *J* = 13.4, 11.7, 4.4 Hz, 1H), 0.92 (ddq, *J* = 13.3, 4.4, 2.3 Hz, 1H), 0.73 (ddq, *J* = 13.4, 4.5, 2.3 Hz, 1H).

**^19^F NMR (471 MHz, CDCl_3_)** δ -62.3 (s, 3H).

**^13^C NMR (126 MHz, CDCl_3_)** δ 152.9, 147.7, 145.2, 144.5, 129.1, 128.6, 128.2 (q, *J* = 32.4 Hz), 127.9, 126.3, 124.5 (q, *J* = 3.7 Hz), 124.4 (q, *J* = 271.8 Hz), 116.5, 112.5, 68.14, 68.11, 50.5, 44.5, 41.2, 34.8, 34.7, 31.6, 21.6.

**HRMS (pos. APCI)** m/z: [M+H]^+^ Calcd for C_26_H_30_F_3_O 415.2243. Found 415.2251.

##### 2-(4-(2,7-dimethyl-6-methylene-4-phenyloct-7-en-4-yl)phenyl)propanenitrile (5-19)

([See spectra](#_1H_NMR_Spectrum_59))

The reaction was performed following a slightly modified ***GP 5*** with 100 mg 3 Å MS as additive. The crude mixture was first roughly purified though column chromatography on silica gel (PE:EtOAc = 10:1) to get rid of the majority of polar side products. The residue was then carefully purified by a reverse phase column chromatography on C18 to afford the title compounds as a colorless oil (38.4 mg, 54%). The diastereoselectivity was determined to be 1:1 based on both ^1^H NMR and ^13^C NMR.

**TLC** (SiO_2_): R*_f_* = 0.25 (PE:EtOAc = 10:1)

**^1^H NMR (400 MHz, CDCl_3_)** δ 7.25 – 7.09 (m, 9H), 5.02 (d, *J* = 1.6 Hz, 1H), 4.72 – 4.66 (m, 1H), 4.59 – 4.54 (m, 1H), 4.49 – 4.47 (m, 1H) (*Set of isomer peak 1*), 4.47 – 4.43 (m, 1H) (*Set of isomer peak 1*), 3.80 (q, *J* = 7.3 Hz, 1H), 3.16 (dd, *J* = 13.4, 0.8 Hz, 1H) (*Set of isomer peak 2*), 3.15 (dd, *J* = 13.4, 0.8 Hz, 1H) (*Set of isomer peak 2*), 3.10 (dd, *J* = 13.4, 0.8 Hz, 1H) (*Set of isomer peak 3*), 3.10 (dd, *J* = 13.4, 0.8 Hz, 1H) (*Set of isomer peak 3*), 2.09 – 1.95 (m, 2H), 1.63 (dd, *J* = 1.4, 0.6 Hz, 3H) (*Set of isomer peak 4*), 1.62 (dd, *J* = 1.5, 0.7 Hz, 3H) (*Set of isomer peak 4*), 1.58 – 1.48 (m, 4H), 0.55 (d, *J* = 6.6 Hz, 3H) (*Set of isomer peak 5*), 0.53 (d, *J* = 6.6 Hz, 3H) (*Set of isomer peak 6*), 0.49 (d, *J* = 6.6 Hz, 3H) (*Set of isomer peak 5*), 0.47 (d, *J* = 6.6 Hz, 3H) (*Set of isomer peak 6*).

**^13^C NMR (101 MHz, CDCl_3_)** δ 149.99, 148.25, 145.74 (*Set of isomer peak 1*), 145.68 (*Set of isomer peak 1*), 144.56, 136.18 (*Set of isomer peak 2*), 136.16 (*Set of isomer peak 2*), 128.85, 128.81, 128.33 (*Set of isomer peak 3*), 128.29 (*Set of isomer peak 3*), 127.68 (*Set of isomer peak 4*), 127.66 (*Set of isomer peak 4*), 127.55 (*Set of isomer peak 5*), 127.52 (*Set of isomer peak 5*), 125.94, 123.95 (*Set of isomer peak 6*), 123.93 (*Set of isomer peak 6*), 121.75 (*Set of isomer peak 7*), 121.72 (*Set of isomer peak 7*), 116.18 (*Set of isomer peak 8*), 116.14 (*Set of isomer peak 8*), 112.32, 50.66, 46.44 (*Set of isomer peak 9*), 46.41 (*Set of isomer peak 9*), 41.47 (*Set of isomer peak 10*), 41.43 (*Set of isomer peak 10*), 31.45 (*Set of isomer peak 11*), 31.43 (*Set of isomer peak 11*), [24.76, 24.70, 24.61, 24.53] (*Set of isomer peak 12 and 13*), 24.46, 21.60, 21.56.

**HRMS (pos. APCI)** m/z: [M+H]^+^ Calcd for C_26_H_32_N 358.2529. Found 358.2533.

##### 2-(4-(2,7-dimethyl-6-methylene-4-phenyloct-7-en-4-yl)phenyl)-4,4,5,5-tetramethyl-1,3,2-dioxaborolane (5-20)

([See spectra](#_1H_NMR_Spectrum_60))

The reaction was performed following a slightly modified ***GP 5*** with 100 mg 3 Å MS as additive. The crude mixture was first roughly purified though column chromatography on silica gel (PE) to get rid of the majority of polar side products. The residue was then carefully purified by a reverse phase column chromatography on C18 to afford the title compounds as a colorless oil (20.7 mg, 24%).

**TLC** (SiO_2_): R*_f_* = 0.34 (PE)

**^1^H NMR (500 MHz, CDCl_3_)** δ 7.67 – 7.63 (m, 2H), 7.22 – 7.10 (m, 7H), 4.97 (d, *J* = 1.6 Hz, 1H), 4.79 – 4.76 (m, 1H), 4.64 – 4.60 (m, 1H), 4.41 – 4.37 (m, 1H), 3.16 (dd, *J* = 13.8, 0.7 Hz, 1H), 3.15 – 3.11 (m, 1H), 2.04 (dd, *J* = 13.8, 5.4 Hz, 1H), 2.00 (dd, *J* = 13.9, 5.2 Hz, 1H), 1.66 (d, *J* = 1.3 Hz, 3H), 1.56 – 1.48 (m, 1H), 1.34 (s, 12H), 0.51 (d, *J* = 6.6 Hz, 3H), 0.50 (d, *J* = 6.6 Hz, 3H).

**^13^C NMR (126 MHz, CDCl_3_)** δ 152.3, 148.7, 145.6, 144.8, 134.1, 128.9, 128.3, 127.5, 125.7, 116.0, 112.3, 83.7, 50.7, 46.3, 41.1, 25.00, 24.98, 24.8, 24.7, 24.5, 21.7.

**HRMS (pos. APCI)** m/z: [M+H]^+^ Calcd for C_29_H_40_O_2_B 431.3116. Found 431.3109.

##### 4-(2,7-dimethyl-6-methylene-4-phenyloct-7-en-4-yl)phenol (5-21)

([See spectra](#_1H_NMR_Spectrum_61))

The reaction was performed following a slightly modified ***GP 5*** with 100 mg 3 Å MS as additive. The crude mixture was first roughly purified though column chromatography on silica gel (PE:EtOAc = 4:1) to get rid of the majority of side products. The residue was then carefully purified by a reverse phase column chromatography on C18 to afford the title compounds as a colorless oil (20.1 mg, 31%).

**TLC** (SiO_2_): R*_f_* = 0.25 (PE:EtOAc = 4:1)

**^1^H NMR (400 MHz, CDCl_3_)** δ 7.23 – 7.16 (m, 4H), 7.16 – 7.10 (m, 1H), 7.06 – 7.01 (m, 2H), 6.71 – 6.65 (m, 2H), 5.00 (d, *J* = 1.7 Hz, 1H), 4.77 – 4.72 (m, 1H), 4.65 (br, s, 1H), 4.63 – 4.59 (m, 1H), 4.45 – 4.41 (m, 1H), 3.11 (dd, *J* = 13.5, 0.9 Hz, 1H), 3.08 (dd, *J* = 13.6, 0.8 Hz, 1H), 1.97 (d, *J* = 5.2 Hz, 2H), 1.65 (dd, *J* = 1.4, 0.6 Hz, 3H), 1.58 – 1.46 (m, 1H), 0.53 (d, *J* = 6.6 Hz, 3H), 0.50 (d, *J* = 6.6 Hz, 3H).

**^13^C NMR (101 MHz, CDCl_3_)** δ 153.4, 149.0, 145.9, 144.8, 141.3, 130.0, 128.8, 127.5, 125.6, 115.9, 114.3, 112.2, 49.9, 46.6, 41.4, 24.8, 24.7, 24.5, 21.6.

**HRMS (pos. APCI)** m/z: [M+H]^+^ Calcd for C_23_H_29_O 321.2213. Found 321.2209.

##### methyl 4-(2,7-dimethyl-6-methylene-4-phenyloct-7-en-4-yl)benzoate (5-23)

([See spectra](#_1H_NMR_Spectrum_62))

The reaction was performed following a slightly modified ***GP 5*** with 100 mg 3 Å MS as additive. The crude mixture was first roughly purified though column chromatography on silica gel (PE:EtOAc = 40:1) to get rid of the majority of polar side products. The residue was then carefully purified by a reverse phase column chromatography on C18 to afford the title compounds as a colorless oil (18.5 mg, 26%).

**TLC** (SiO_2_): R*_f_* = 0.26 (PE:EtOAc = 40:1)

**^1^H NMR (400 MHz, CDCl_3_)** δ 7.90 – 7.85 (m, 2H), 7.28 – 7.18 (m, 4H), 7.18 – 7.13 (m, 3H), 5.00 (d, *J* = 1.6 Hz, 1H), 4.73 (dq, *J* = 1.2, 0.6 Hz, 1H), 4.62 – 4.57 (m, 1H), 4.47 – 4.42 (m, 1H), 3.89 (s, 3H), 3.18 (dd, *J* = 13.5, 0.9 Hz, 1H), 3.13 (dd, *J* = 13.4, 0.8 Hz, 1H), 2.07 (dd, *J* = 13.8, 4.9 Hz, 1H), 2.02 (dd, *J* = 13.9, 5.3 Hz, 1H), 1.64 (dd, *J* = 1.4, 0.6 Hz, 3H), 1.60 – 1.47 (m, 1H), 0.54 (d, *J* = 6.6 Hz, 3H), 0.49 (d, *J* = 6.6 Hz, 3H).

**^13^C NMR (101 MHz, CDCl_3_)** δ 167.3, 154.6, 148.1, 145.5, 144.6, 129.0, 128.83, 128.78, 127.7, 127.6, 126.0, 116.2, 112.5, 52.0, 50.8, 46.3, 41.1, 24.8, 24.6, 24.5, 21.6.

**HRMS (pos. ESI)** m/z: [M+H]^+^ Calcd for C_25_H_31_O_2_ 363.2319. Found 363.2318.

##### 4,4'-(2,7-dimethyl-6-methyleneoct-7-ene-4,4-diyl)bis(methoxybenzene) (5-24)

([See spectra](#_1H_NMR_Spectrum_63))

The reaction was performed following a slightly modified ***GP 5*** using 1.0 mol% [Ir(ppy)_2_dtbbpy]PF_6_, 10 mol% Pd(OAc)_2_ and 24 mol% (2-furyl)_3_P. The crude mixture was first roughly purified though column chromatography on silica gel (PE:EtOAc = 40:1) to get rid of the majority of polar side products. The residue was then carefully purified by a reverse phase column chromatography on C18 to afford the title compounds as a colorless oil (14.6 mg, 20%).

**TLC** (SiO_2_): R*_f_* = 0.25 (PE:EtOAc = 40:1)

**^1^H NMR (400 MHz, CDCl_3_)** δ 7.12 – 7.04 (m, 4H), 6.79 – 6.71 (m, 4H), 5.00 (d, *J* = 1.7 Hz, 1H), 4.78 – 4.73 (m, 1H), 4.65 – 4.60 (m, 1H), 4.44 – 4.40 (m, 1H), 3.78 (s, 6H), 3.07 (d, *J* = 0.9 Hz, 2H), 1.95 (d, *J* = 5.2 Hz, 2H), 1.66 (dd, *J* = 1.4, 0.6 Hz, 3H), 1.57 – 1.46 (m, 1H), 0.52 (d, *J* = 6.6 Hz, 6H).

**^13^C NMR (101 MHz, CDCl_3_)** δ 157.5, 145.9, 144.9, 141.2, 129.8, 115.8, 112.8, 112.2, 55.3, 49.3, 46.8, 41.6, 24.8, 24.5, 21.7.

**HRMS (pos. ESI)** m/z: [M+H]^+^ Calcd for C_25_H_33_O_2_ 365.2475. Found 365.2477.

##### 1-(1-cyclohexyl-2-(4-fluorophenyl)-5-methyl-4-methylenehex-5-en-2-yl)-4-methoxybenzene (5-25)

([See spectra](#_1H_NMR_Spectrum_64))

The reaction was performed following the ***GP 5***. The crude mixture was first roughly purified though column chromatography on silica gel (PE) to get rid of the majority of polar side products. The residue was then carefully purified by a reverse phase column chromatography on C18 to afford the title compounds as a colorless oil (27.3 mg, 35%).

**TLC** (SiO_2_): R*_f_* = 0.20 (PE)

**^1^H NMR (500 MHz, CDCl_3_)** δ 7.16 – 7.10 (m, 2H), 7.09 – 7.05 (m, 2H), 6.91 – 6.85 (m, 2H), 6.78 – 6.73 (m, 2H), 5.01 (d, *J* = 1.6 Hz, 1H), 4.74 – 4.71 (m, 1H), 4.64 – 4.60 (m, 1H), 4.46 – 4.43 (m, 1H), 3.79 (s, 3H), 3.08 (dd, *J* = 13.5, 0.8 Hz, 1H), 3.05 (dd, *J* = 13.4, 0.9 Hz, 1H), 1.93 (dd, *J* = 13.2, 4.1 Hz, 1H), 1.90 (dd, *J* = 13.2, 4.2 Hz, 1H), 1.65 (dd, *J* = 1.3, 0.5 Hz, 3H), 1.53 – 1.41 (m, 3H), 1.23 – 1.13 (m, 1H), 1.10 – 0.93 (m, 5H), 0.77 – 0.63 (m, 2H).

**^19^F NMR (471 MHz, CDCl_3_)** δ -118.09 – -118.23 (m, 3F).

**^13^C NMR (126 MHz, CDCl_3_)** δ 161.0 (d, *J* = 244.2 Hz), 157.6, 145.7, 144.8 (d, *J* = 3.1 Hz), 144.7, 140.7, 130.2 (d, *J* = 7.6 Hz), 129.7, 116.0, 114.1 (d, *J* = 20.8 Hz), 112.9, 112.3, 55.3, 49.5, 45.6, 41.8, 35.2, 35.1, 34.1, 26.7, 26.5, 21.6.

**HRMS (pos. APCI)** m/z: [M+H]^+^ Calcd for C_27_H_34_FO 393.2588. Found 393.2596.

##### 4-bromo-1-chloro-2-(4-(4-ethoxyphenyl)-2,7-dimethyl-6-methyleneoct-7-en-4-yl)benzene (5-26)

([See spectra](#_1H_NMR_Spectrum_65))

The reaction was performed following a slightly modified ***GP 5*** using 1.0 mol% [Ir(ppy)_2_dtbbpy]PF_6_, 10 mol% Pd(OAc)_2_ and 24 mol% (2-furyl)_3_P. The crude mixture was first roughly purified though column chromatography on silica gel (PE:EtOAc = 100:1) to get rid of the majority of polar side products. The residue was then carefully purified by a reverse phase column chromatography on C18 to afford the title compounds as a colorless oil (24.8 mg, 27%).

**TLC** (SiO_2_): R*_f_* = 0.30 (PE:EtOAc = 100:1)

**^1^H NMR (400 MHz, CDCl_3_)** δ 7.59 (d, *J* = 2.4 Hz, 1H), 7.26 (dd, *J* = 8.4, 2.4 Hz, 1H), 7.04 (d, *J* = 8.4 Hz, 1H), 6.97 – 6.90 (m, 2H), 6.77 – 6.70 (m, 2H), 5.05 (d, *J* = 1.8 Hz, 1H), 4.75 – 4.70 (m, 1H), 4.58 – 4.53 (m, 2H), 4.00 (q, *J* = 7.0 Hz, 2H), 3.44 (d, *J* = 13.0 Hz, 1H), 3.02 (d, *J* = 13.0 Hz, 1H), 2.17 (dd, *J* = 13.7, 5.0 Hz, 1H), 1.88 (dd, *J* = 13.6, 5.0 Hz, 1H), 1.63 (dd, *J* = 1.3, 0.7 Hz, 3H), 1.60 – 1.45 (m, 1H), 1.39 (t, *J* = 7.0 Hz, 3H), 0.58 (d, *J* = 6.6 Hz, 3H), 0.49 (d, *J* = 6.7 Hz, 3H).

**^13^C NMR (101 MHz, CDCl_3_)** δ 157.1, 147.3, 146.4, 144.6, 138.8, 134.6, 133.1, 133.0, 130.4, 128.4, 120.2, 116.6, 113.8, 112.2, 63.4, 49.9, 24.7, 24.5, 24.4, 21.8, 14.9.

**HRMS (pos. APCI)** m/z: [M+H]^+^ Calcd for C_25_H_31_^81^Br^35^ClO 463.1221. Found 463.1209.

##### 9-(cyclohexylmethyl)-10,10-dimethyl-9-(3-methyl-2-methylenebut-3-en-1-yl)-9,10-dihydroanthracene (5-28)

([See spectra](#_1H_NMR_Spectrum_66))

The reaction was performed following a slightly modified ***GP 5*** with 100 mg 3 Å MS as additive. The crude mixture was first roughly purified though column chromatography on silica gel (PE) to get rid of the majority of polar side products. The residue was then carefully purified by a reverse phase column chromatography on C18 to afford the title compounds as a colorless oil (42.3 mg, 55%).

**TLC** (SiO_2_): R*_f_* = 0.36 (PE)

**^1^H NMR (400 MHz, CDCl_3_)** δ 7.42 (dd, *J* = 7.7, 1.6 Hz, 2H), 7.40 (dd, *J* = 7.9, 6.3 Hz, 2H), 7.19 (ddd, *J* = 7.9, 7.1, 1.6 Hz, 2H), 7.14 (ddd, *J* = 7.7, 7.0, 1.6 Hz, 2H), 4.67 – 4.66 (m, 1H), 4.66 (dq, *J* = 1.8, 0.6 Hz, 1H), 4.56 – 4.51 (m, 1H), 4.10 – 4.05 (m, 1H), 3.02 (d, *J* = 1.0 Hz, 2H), 2.08 (d, *J* = 5.2 Hz, 2H), 1.64 (s, 3H), 1.61 (s, 3H), 1.45 (dd, *J* = 1.4, 0.6 Hz, 3H), 1.40 – 1.28 (m, 3H), 1.00 – 0.72 (m, 6H), 0.67 – 0.53 (m, 2H).

**^13^C NMR (101 MHz, CDCl_3_)** δ 144.8, 144.7, 143.8, 137.8, 127.3, 126.7, 125.9, 125.6, 114.9, 111.8, 54.4, 49.0, 45.8, 36.9, 35.9, 34.9, 34.7, 34.4, 26.32, 26.27, 21.3.

**HRMS (pos. APCI)** m/z: [M+NH_4_]^+^ Calcd for C_29_H_40_N 402.3155. Found 402.3159.

##### isopropyl 2-(4-(2-(4-chlorophenyl)-5-methyl-4-methylene-1-(tetrahydro-2*H*-pyran-4-yl)hex-5-en-2-yl)phenoxy)-2-methylpropanoate (5-30)

([See spectra](#_1H_NMR_Spectrum_68))

The reaction was performed following the ***GP 5***. The crude mixture was first roughly purified though column chromatography on silica gel (PE:EtOAc = 10:1) to get rid of the majority of side products. The residue was then carefully purified by a reverse phase column chromatography on C18 to afford the title compounds as a colorless oil (51.5 mg, 49%).

**TLC** (SiO_2_): R*_f_* = 0.20 (PE:EtOAc = 10:1)

**^1^H NMR (400 MHz, CDCl_3_)** δ 7.20 – 7.13 (m, 2H), 7.12 – 7.05 (m, 2H), 7.04 – 6.96 (m, 2H), 6.75 – 6.68 (m, 2H), 5.08 (hept, *J* = 6.3 Hz, 1H), 5.00 (d, *J* = 1.6 Hz, 1H), 4.74 – 4.69 (m, 1H), 4.64 – 4.59 (m, 1H), 4.40 – 4.35 (m, 1H), 3.76 – 3.63 (m, 2H), 3.19 – 2.98 (m, 4H), 1.97 (dd, *J* = 14.1, 4.9 Hz, 1H), 1.93 (dd, *J* = 14.1, 5.0 Hz, 1H), 1.64 (dd, *J* = 1.4, 0.6 Hz, 3H), 1.55 (s, 6H), 1.47 – 1.35 (m, 1H), 1.22 (d, *J* = 6.3 Hz, 6H), 1.15 – 0.96 (m, 2H), 0.88 (ddq, *J* = 13.6, 4.5, 2.3 Hz, 1H), 0.80 (ddq, *J* = 13.4, 4.0, 2.4 Hz, 1H).

**^13^C NMR (101 MHz, CDCl_3_)** δ 173.8, 153.7, 147.3, 145.4, 144.6, 141.6, 131.6, 130.2, 129.2, 127.6, 118.5, 116.3, 112.5, 79.1, 68.9, 68.16, 68.13, 49.5, 44.7, 41.5, 34.84, 34.78, 31.6, 25.3, 21.66, 21.60.

**HRMS (pos. ESI)** m/z: [M+Na]^+^ Calcd for C_32_H_41_O_4_^35^ClNa 547.2586. Found 547.2584.

### Crystal Structure and Crystallographic Data

#### Allylic product 4-11 (CCDC-2408584)


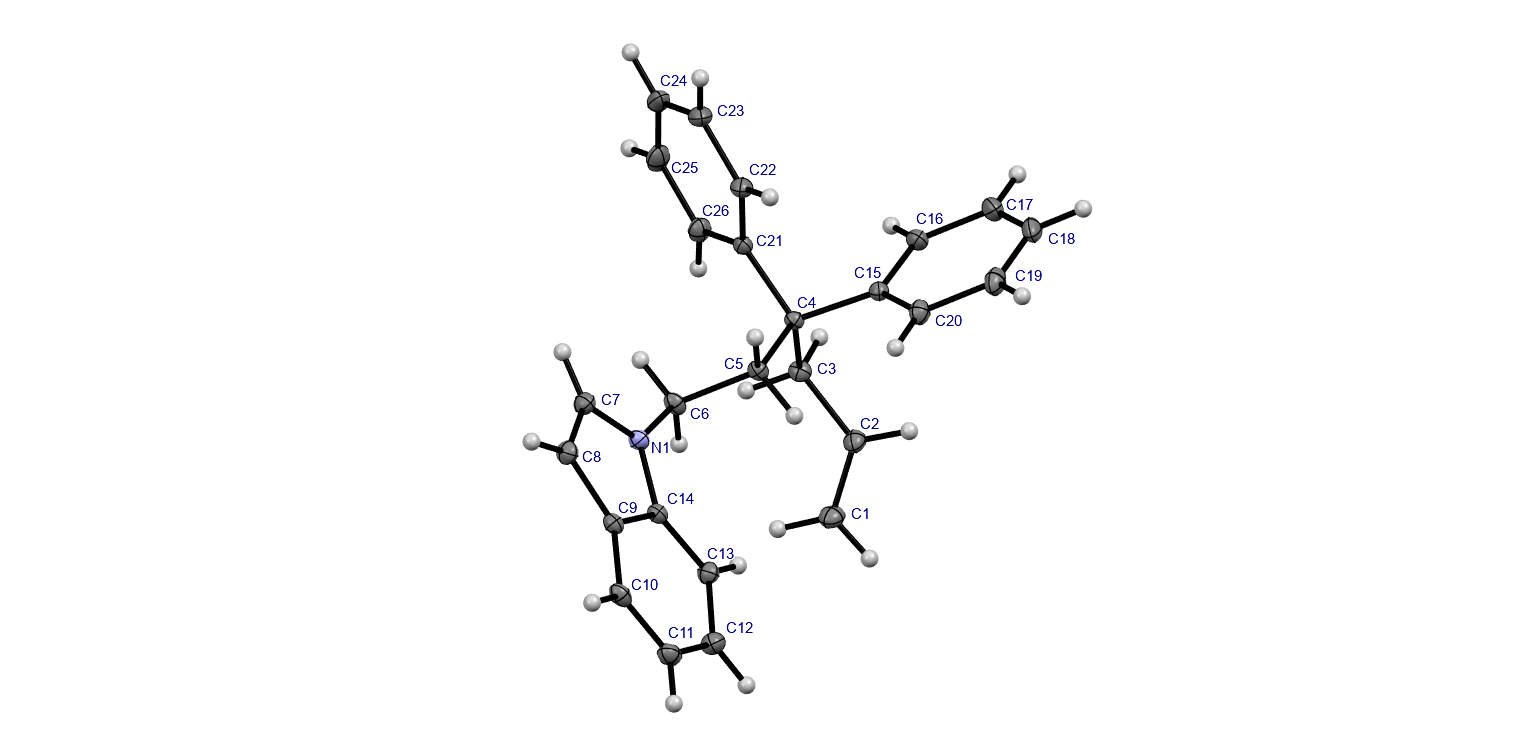


Figure 1 Crystal Structure of 4-11 (ellipsoid contour at 50% probability)

Crystals were obtained at room temperature by slow diffusion of pentane into a solution of the compound dissolved in dichloromethane by the aid of layering. A colorless, block-shaped crystal was mounted on a MiTeGen micromount with perfluoroether oil. Data for **4-11** were collected from a shock-cooled single crystal at 100(2) K on a Bruker D8 VENTURE dual wavelength Mo/Cu three-circle diffractometer with a microfocus sealed X-ray tube using a mirror optics as monochromator and a Bruker PHOTON III detector. The diffractometer was equipped with an Oxford Cryostream 800 low temperature device and used Mo*K_α_* radiation (λ = 0.71073 Å). All data were integrated with SAINT V8.41 and a multi-scan absorption correction using SADABS 2016/2 was applied. The structure was solved by direct methods with SHELXT and refined by full-matrix least-squares methods against *F*^2^ using SHELXL-2019/2. All non-hydrogen atoms were refined with anisotropic displacement parameters. All hydrogen atoms were refined isotropic on calculated positions using a riding model with their *U*_iso_ values constrained to 1.5 times the *U*_eq_ of their pivot atoms for terminal sp^3^ carbon atoms and 1.2 times for all other carbon atoms. Crystallographic data for the structures reported in this paper have been deposited with the Cambridge Crystallographic Data Centre. CCDC 2408584 contain the supplementary crystallographic data for this paper. These data can be obtained free of charge from The Cambridge Crystallographic Data Centre via www.ccdc.cam.ac.uk/‌structures. This report and the CIF file were generated using FinalCif ([See procedure](#_1-(3,3-diphenylhex-5-en-1-yl)-1H-in)).

| CCDC number | 2408584 |
| --- | --- |
| Empirical formula | C_26_H_25_N |
| Formula weight | 351.47 |
| Temperature [K] | 100(2) |
| Crystal system | monoclinic |
| Space group (number) | $P2_{1}/c$ (14) |
| *a* [Å] | 19.257(3) |
| *b* [Å] | 6.1188(9) |
| *c* [Å] | 17.604(4) |
| α [°] | 90 |
| β [°] | 111.078(5) |
| γ [°] | 90 |
| Volume [Å^3^] | 1935.5(6) |
| *Z* | 4 |
| *ρ*_calc_ [gcm^−3^] | 1.206 |
| *μ* [mm^−1^] | 0.069 |
| *F*(000) | 752 |
| Crystal size [mm^3^] | 0.122×0.228×0.422 |
| Crystal color | colorless |
| Crystal shape | block |
| Radiation | Mo*K_α_* (λ=0.71073 Å) |
| 2θ range [°] | 4.53 to 66.55 (0.65 Å) |
| Index ranges | −29 ≤ h ≤ 29 −9 ≤ k ≤ 9 −27 ≤ l ≤ 27 |
| Reflections collected | 130201 |
| Independent reflections | 7406  *R*_int_ = 0.0434 *R*_sigma_ = 0.0172 |
| Completeness to θ = 25.242° | 99.9 % |
| Data / Restraints / Parameters | 7406 / 0 / 244 |
| Absorption correction T_min_/T_max_ (method) | 0.7162 / 0.7465  (multi-scan) |
| Goodness-of-fit on *F*^2^ | 1.039 |
| Final *R* indexes  [*I*≥2σ(*I*)] | *R*_1_ = 0.0402 w*R*_2_ = 0.1079 |
| Final *R* indexes  [all data] | *R*_1_ = 0.0455 w*R*_2_ = 0.1120 |
| Largest peak/hole [eÅ^−3^] | 0.48/−0.22 |

#### Dienylic product 5-12 (CCDC-2401874)


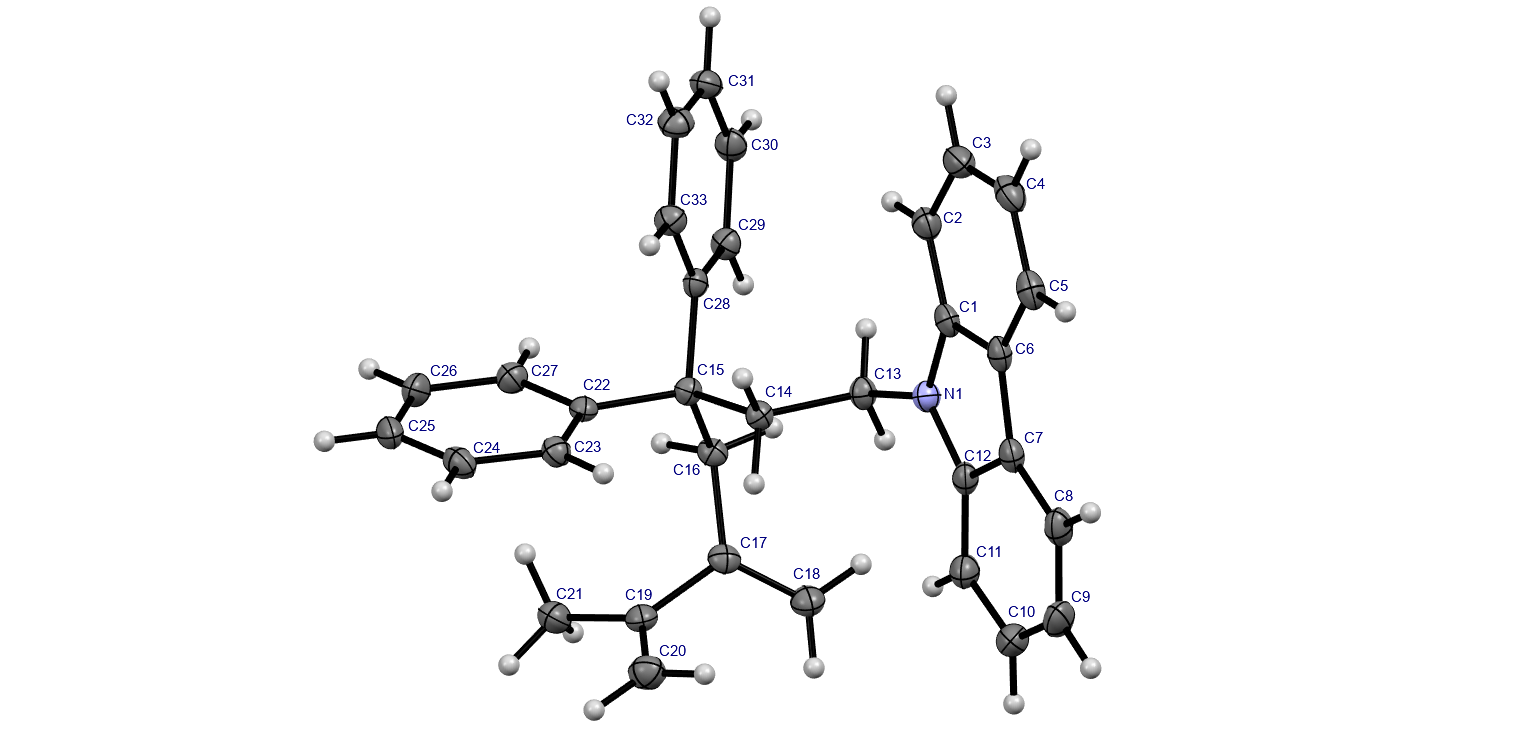


Figure 2 Crystal Structure of 5-12 (ellipsoid contour at 50% probability)

Crystals were obtained at 4 °C by slow diffusion of diethyl ether into a solution of the compound dissolved in dichloromethane by the aid of layering. A colorless, plate-shaped crystal was mounted on a MiTeGen micromount with perfluoroether oil. Data for **5-12** were collected from a shock-cooled single crystal at 100(2) K on a Bruker D8 VENTURE dual wavelength Mo/Cu three-circle diffractometer with a microfocus sealed X-ray tube using a mirror optics as monochromator and a Bruker PHOTON III detector. The diffractometer was equipped with an Oxford Cryostream 800 low temperature device and used Cu*K_α_* radiation (λ = 1.54178 Å). All data were integrated with SAINT V8.41 and a multi-scan absorption correction using SADABS 2016/2 was applied. The structure was solved by direct methods with SHELXT and refined by full-matrix least-squares methods against *F*^2^ using SHELXL-2019/2. All non-hydrogen atoms were refined with anisotropic displacement parameters. All hydrogen atoms were refined isotropic on calculated positions using a riding model with their *U*_iso_ values constrained to 1.5 times the *U*_eq_ of their pivot atoms for terminal sp^3^ carbon atoms and 1.2 times for all other carbon atoms. Crystallographic data for the structures reported in this paper have been deposited with the Cambridge Crystallographic Data Centre. CCDC 2401874 contain the supplementary crystallographic data for this paper. These data can be obtained free of charge from The Cambridge Crystallographic Data Centre via www.ccdc.cam.ac.uk/‌structures. This report and the CIF file were generated using FinalCif. ([See procedure](#_9-(6-methyl-5-methylene-3,3-dipheny)).

| CCDC number | 2401874 |
| --- | --- |
| Empirical formula | C_33_H_31_N |
| Formula weight | 441.59 |
| Temperature [K] | 100(2) |
| Crystal system | orthorhombic |
| Space group (number) | $P2_{1}2_{1}2_{1}$ (19) |
| *a* [Å] | 6.1819(5) |
| *b* [Å] | 19.2993(18) |
| *c* [Å] | 20.5111(19) |
| α [°] | 90 |
| β [°] | 90 |
| γ [°] | 90 |
| Volume [Å^3^] | 2447.1(4) |
| *Z* | 4 |
| *ρ*_calc_ [gcm^−3^] | 1.199 |
| *μ* [mm^−1^] | 0.517 |
| *F*(000) | 944 |
| Crystal size [mm^3^] | 0.019×0.088×0.134 |
| Crystal color | colorless |
| Crystal shape | plate |
| Radiation | Cu*K_α_* (λ=1.54178 Å) |
| 2θ range [°] | 6.29 to 149.10 (0.80 Å) |
| Index ranges | −7 ≤ h ≤ 7 −24 ≤ k ≤ 24 −25 ≤ l ≤ 25 |
| Reflections collected | 132034 |
| Independent reflections | 5015  *R*_int_ = 0.0721 *R*_sigma_ = 0.0171 |
| Completeness to θ = 67.679° | 100.0 % |
| Data / Restraints / Parameters | 5015 / 0 / 308 |
| Absorption correction T_min_/T_max_ (method) | 0.7059 / 0.7501  (multi-scan) |
| Goodness-of-fit on *F*^2^ | 1.044 |
| Final *R* indexes  [*I*≥2σ(*I*)] | *R*_1_ = 0.0290 w*R*_2_ = 0.0699 |
| Final *R* indexes  [all data] | *R*_1_ = 0.0311 w*R*_2_ = 0.0715 |
| Largest peak/hole [eÅ^−3^] | 0.19/−0.18 |
| Flack X parameter | -0.01(19) |

## Extended scope

Unless otherwise noted, the chemoselectivity between allylation and dienylation for both the allylation and dienylation direction was higher than 19:1.

### Allylation direction

#### Scope

Scheme 1 Allylation direction-Scope. Std. Cond. 1: Propa-1,2-diene (0.2 mmol, 1.0 equiv.), 1,1-diphenylethylene (1.8 equiv.), iPr-DHP (1.5 equiv.), [Ir(ppy)_2_dtbbpy]PF_6_ (0.5 mol%), Pd(OAc)_2_ (7.5 mol%), Xantphos (9 mol%), 4-Cl-BzOH (5 mol%) in toluene (2 mL) were irradiated with 16 W blue LEDs for 16 h under argon. Std. Cond. 2: Propa-1,2-diene (0.2 mmol, 1.0 equiv.), 1,1-diphenylethylene (1.8 equiv.), iPr-DHP (1.5 equiv.), [Ir(ppy)_2_dtbbpy]PF_6_ (0.5 mol%), Pd_2_(4,4’-MeO-dba)_3_ (3.75 mol%), (2-furyl)_3_P (18 mol%), PPTS (10 mol%) in toluene (2 mL) were irradiated with 16 W blue LEDs for 16 h under argon. Unless otherwise noted, the reaction was performed following the Std. Cond. 1. a: w/ (η^3^-cinnamyl)PdCp, b: w/ 1.2 equiv. DHP. c: w/ 1.0 mol% [Ir(ppy)_2_dtbbpy]PF_6_, 10 mol% Pd(OAc)_2_ and 12.5 mol% Xantphos., d: w/ 1.5 equiv. diPhethylene. e: w/ Std. Cond. 2.

#### Limitations

Scheme 2 Allylation direction-Limitations. Std. Cond. 1: Propa-1,2-diene (0.2 mmol, 1.0 equiv.), 1,1-diphenylethylene (1.8 equiv.), iPr-DHP (1.5 equiv.), [Ir(ppy)_2_dtbbpy]PF_6_ (0.5 mol%), Pd(OAc)_2_ (7.5 mol%), Xantphos (9 mol%), 4-Cl-BzOH (5 mol%) in toluene (2 mL) were irradiated with 16 W blue LEDs for 16 h under argon. Std. Cond. 2: Propa-1,2-diene (0.2 mmol, 1.0 equiv.), 1,1-diphenylethylene (1.8 equiv.), iPr-DHP (1.5 equiv.), [Ir(ppy)_2_dtbbpy]PF_6_ (0.5 mol%), Pd_2_(4,4’-MeO-dba)_3_ (3.75 mol%), (2-furyl)_3_P (18 mol%), PPTS (10 mol%) in toluene (2 mL) were irradiated with 16 W blue LEDs for 16 h under argon. Unless otherwise noted, the reaction was performed following the Std. Cond. 1. a: w/ (η^3^-cinnamyl)PdCp. b: The reaction was performed following the Std. Cond. 2. c: w/ Pd_2_(dba)_3_. d: Isolation failed. DHP = Dihydropyridine. PPTS = Pyridinium *p*-toluenesulfonate.

#### Comparisons

Scheme 3 Allylation direction-Comparisons. Std. Cond. 1: Propa-1,2-diene (0.2 mmol, 1.0 equiv.), 1,1-diphenylethylene (1.8 equiv.), iPr-DHP (1.5 equiv.), [Ir(ppy)_2_dtbbpy]PF_6_ (0.5 mol%), Pd(OAc)_2_ (7.5 mol%), Xantphos (9 mol%), 4-Cl-BzOH (5 mol%) in toluene (2 mL) were irradiated with 16 W blue LEDs for 16 h under argon. Std. Cond. 2: Propa-1,2-diene (0.2 mmol, 1.0 equiv.), 1,1-diphenylethylene (1.8 equiv.), iPr-DHP (1.5 equiv.), [Ir(ppy)_2_dtbbpy]PF_6_ (0.5 mol%), Pd_2_(4,4’-MeO-dba)_3_ (3.75 mol%), (2-furyl)_3_P (18 mol%), PPTS (10 mol%) in toluene (2 mL) were irradiated with 16 W blue LEDs for 16 h under argon. Unless otherwise noted, the reaction was performed following the Std. Cond. 1. a: w/ 1.2 equiv. DHP. b: w/ 1.0 mol% [Ir(ppy)_2_dtbbpy]PF_6_, 10 mol% Pd(OAc)_2_ and 12.5 mol% Xantphos. c: w/ 1.5 equiv. diPhethylene. d: The reaction was performed following the Std. Cond. 2. DHP = Dihydropyridine. PPTS = Pyridinium *p*-toluenesulfonate.

### Dienylation direction

#### Scope

Scheme 4 Dienylation direction-Scope. Reaction condition: Propa-1,2-diene (0.4 mmol, 2*1.0 equiv.), 1,1-diphenylethylene (3.0 equiv.), iPr-DHP (1.5 equiv.), [Ir(ppy)_2_dtbbpy]PF_6_ (0.5 mol%), Pd(OAc)_2_ (7.5 mol%), (2-furyl)_3_P (18 mol%), 4-OMe-Py·TsOH (10 mol%) in toluene (2 mL) were irradiated with 16 W blue LEDs for 16 h under argon. a: w/ 1.2 equiv. DHP. b: w/ 1.0 mol% [Ir(ppy)_2_dtbbpy]PF_6_, 10 mol% Pd(OAc)_2_ and 24 mol% (2-furyl)_3_P. c: w/ 4.0 equiv. diPhethylene. d: w/ 100 mg 3 Å MS. MS = molecular sieve.

#### Limitations

Scheme 5 Dienylation direction-Limitation. Reaction condition: Propa-1,2-diene (0.4 mmol, 2*1.0 equiv.), 1,1-diphenylethylene (3.0 equiv.), iPr-DHP (1.2 equiv.), [Ir(ppy)_2_dtbbpy]PF_6_ (0.5 mol%), Pd(OAc)_2_ (7.5 mol%), (2-furyl)_3_P (18 mol%), 4-OMe-Py·TsOH (10 mol%) in toluene (2 mL) were irradiated with 16 W blue LEDs for 16 h under argon. a: w/ 100 mg 3 Å MS. b: w/ 1.0 mol% [Ir(ppy)_2_dtbbpy]PF_6_, 10 mol% Pd(OAc)_2_ and 24 mol% (2-furyl)_3_P. MS = molecular sieve.

## Practicability investigation

### Gram scale reactions

#### Allylation-Std. Cond. 1

The reaction was performed following ***GP 3*** on a 5 mmol scale. The reaction time was extended to 48 h to ensure a high degree of starting materials consumption. The reaction mixture was evaporated under reduced pressure and the residue was first roughly purified though column chromatography on silica gel (PE:EtOAc = 100:1) to get rid of the majority of polar side products. The residue was then carefully purified by a reverse phase column chromatography portionwise on C18 to afford the title compound as a yellowish oil (1.35 g, 70%). The characterization data was in accordance with the catalytic example ([See characterization data](#_(See_gram_scale)).

#### Allylation-Std. Cond. 2

The reaction was performed following ***GP 4*** on a 5 mmol scale. The reaction time was extended to 48 h to ensure a high degree of starting materials consumption. The reaction mixture was evaporated under reduced pressure and the residue was first roughly purified though column chromatography on silica gel (PE:EtOAc = 10:1) to get rid of the majority of side products. The residue was then carefully purified by a reverse phase column chromatography portionwise on C18 to afford the title compound as a colorless oil (1.60 g, 87%). The characterization data was in accordance with the catalytic example ([See characterization data](#_tert-butyl_(3,3-diphenylhex-5-en-1-)).

#### Dienylation

The reaction was performed following a slightly modified ***GP 5*** with 4.0 equiv. of DHP added instead of 3. 0 equiv. on a 5 mmol scale. The reaction time was extended to 48 h to ensure a high degree of starting materials consumption. The reaction mixture was evaporated under reduced pressure and the residue was first roughly purified though column chromatography on silica gel (PE:EtOAc = 50:1) to get rid of the majority of polar side products. The residue was then carefully purified by a reverse phase column chromatography portionwise on C18 to afford the title compound as a yellowish oil (1.21 g, 57%). The characterization data was in accordance with the catalytic example ([See characterization data](#_5-(2,7-dimethyl-6-methylene-4,4-dip)).

*Note: Although the scale up experiments under 1 atm. of propadiene have not yet been conducted, based on the current results, we believe that under such condition the scale-up can also be achieved. In this context, the choice of methods more or less up to the user’s choice. For a long term manufacture a gas cylinder might be more beneficially while for a one time use the preparation of a stock solution might be economically more acceptable.*

### Sequential functionalization of pharmaceuticals

#### An overview

#### Synthesis and characterization data

Catalytic scale:

The reaction was performed following a modified literature report. To an oven dried 8 mL Schlenk tube equipped with a magnetic stirring bar was charged with [Pd(allyl)]_2_Cl_2_ (3.7 mg, 0.01 mmol, 5.0 mol%) and rac-BINAP (14.9 mg, 0.024 mmol, 12.0 mol%) in air. The tube was evacuated and refilled with argon for three times before toluene was added. The tube was then sealed with a screw cap and allowed to stir for 30 min before Cs_2_CO_3_ (6.5 mg, 0.02 mmol, 10 mol%), Fenofibric acid (63.7 mg, 0.2 mmol, 1.0 equiv.) and the stock solution of Propa-1,2-diene (5.0 equiv.) was subjected to the reaction mixture under an argon counterflow. The overall concentration of propadiene set to be 0.2 M *via* adjusting the volume of toluene added during pre-string. The resulting mixture was allowed to stir for 16 h at 60 °C. The crude mixture was then cooled to room temperature and evaporated under reduced pressure. The residue was then redissolved in CDCl_3_ and subjected to ^1^H NMR measurement. The chemoselectivity between allylic product and dienylic product was determined to be 12.9:1 according to the NMR measurement. The sample was then taken to dryness and the residue was purified by flash column chromatography on silica gel (PE:EtOAc = 10:1). Both the allylic product and dienylic product can be isolated and separated in this way.

Characterization data for allylic product:

##### allyl 2-(4-(4-chlorobenzoyl)phenoxy)-2-methylpropanoate (6-1)

[(See spectra](#_1H_NMR_Spectrum_90))

The title compound was collected as a colorless oil (48.1 mg, 67%).

**TLC** (SiO_2_): R*_f_* = 0.24 (PE:EtOAc = 10:1)

**^1^H NMR (500 MHz, CDCl_3_)** δ 7.75 – 7.69 (m, 4H), 7.47 – 7.43 (m, 2H), 6.89 – 6.85 (m, 2H), 5.86 (ddt, *J* = 17.2, 10.4, 5.8 Hz, 1H), 5.30 (dq, *J* = 17.2, 1.5 Hz, 1H), 5.23 (dq, *J* = 10.4, 1.2 Hz, 1H), 4.67 (dt, *J* = 5.8, 1.3 Hz, 2H), 1.69 (s, 6H).

**^13^C NMR (126 MHz, CDCl_3_)** δ 194.3, 173.4, 159.6, 138.4, 136.4, 132.0, 131.4, 131.2, 130.5, 128.6, 119.2, 117.5, 79.5, 66.2, 25.5.

**HRMS (pos. ESI)** m/z: [M+H]^+^ Calcd for C_20_H_20_O_4_^35^Cl 359.1045. Found 359.1039.

Characterization data for dienylic product:

##### 3-methyl-2-methylenebut-3-en-1-yl 2-(4-(4-chlorobenzoyl)phenoxy)-2-methylpropanoate (6-2)

[(See spectra](#_1H_NMR_Spectrum_91))

The title compound was collected as a colorless oil (3.4 mg, 4%).

**TLC** (SiO_2_): R*_f_* = 0.26 (PE:EtOAc = 10:1)

**^1^H NMR (500 MHz, CDCl_3_)** δ 7.7 – 7.7 (m, 4H), 7.5 – 7.4 (m, 2H), 6.9 – 6.8 (m, 2H), 5.3 (d, *J* = 0.9 Hz, 1H), 5.2 – 5.2 (m, 1H), 5.0 – 5.0 (m, 2H), 4.9 (d, *J* = 1.1 Hz, 2H), 1.9 (dd, *J* = 1.4, 0.7 Hz, 3H), 1.7 (s, 6H).

**^13^C NMR (126 MHz, CDCl_3_)** δ 194.3, 173.4, 159.7, 141.5, 140.3, 138.5, 136.5, 132.1, 131.3, 130.5, 128.6, 117.5, 116.1, 113.7, 79.6, 66.2, 25.5, 20.9.

**HRMS (pos. APCI)** m/z: [M+H]^+^ Calcd for C_23_H_24_O_4_^35^Cl 399.1358. Found 399.1350.

Gram scale:

The reaction was performed following a modified literature report. To an oven dried 100 mL Schlenk tube equipped with a magnetic stirring bar was charged with [Pd(allyl)]_2_Cl_2_ (91.5 mg, 0.25 mmol, 5.0 mol%) and rac-BINAP (373.6 mg, 0.60 mmol, 12.0 mol%) in air. The tube was evacuated and refilled with argon for three times before toluene was added. The tube was then sealed with a screw cap and allowed to stir for 30 min before Cs_2_CO_3_ (162.9 mg, 0.50 mmol, 10 mol%), Fenofibric acid (1.59 g, 5.0 mmol, 1.0 equiv.) and the stock solution of Propa-1,2-diene (5.0 equiv.) was subjected to the reaction mixture under an argon counterflow. The overall concentration of propadiene set to be 0.2 M *via* adjusting the volume of toluene added during pre-string. The resulting mixture was allowed to stir for 24 h at 60 °C. The crude mixture was evaporated under reduced pressure and the residue was purified by flash column chromatography on silica gel (PE:EtOAc = 10:1) to afford the title compound as a yellowish oil (0.90 g, 50%). The characterization data were in accordance with the catalytic scale reaction.

##### allyl 2-(4-(1-(4-chlorophenyl)vinyl)phenoxy)-2-methylpropanoate (7)

[(See spectra](#_1H_NMR_Spectrum_92))

The reaction was performed following the ***GP 1*** on a 2.5 mmol scale. The residue was purified by flash column chromatography on silica gel (PE:EtOAc = 40:1) to afford the title compound as a colorless oil (0.48 g, 53%).

**TLC** (SiO_2_): R*_f_* = 0.44 (PE:EtOAc = 40:1)

**^1^H NMR (400 MHz, CDCl_3_)** δ 7.31 – 7.27 (m, 2H), 7.27 – 7.23 (m, 2H), 7.20 – 7.16 (m, 2H), 6.82 – 6.77 (m, 2H), 5.88 (ddt, *J* = 17.2, 10.4, 5.8 Hz, 1H), 5.40 (d, *J* = 1.2 Hz, 1H), 5.34 (d, *J* = 1.2 Hz, 1H), 5.30 (dq, *J* = 17.2, 1.5 Hz, 1H), 5.23 (dq, *J* = 10.5, 1.2 Hz, 1H), 4.67 (dt, *J* = 5.8, 1.4 Hz, 2H), 1.63 (s, 6H).

**^13^C NMR (101 MHz, CDCl_3_)** δ 173.9, 155.4, 148.5, 140.2, 134.9, 133.6, 131.7, 129.7, 129.1, 128.4, 118.95, 118.87, 113.8, 79.3, 66.0, 25.6.

**HRMS (pos. APCI)** m/z: [M+H]^+^ Calcd for C_21_H_22_O_3_^35^Cl 357.1252. Found 357.1248.

The reaction was performed following the ***GP 5*** on a 0.2 mmol scale. The crude mixture was first roughly purified though column chromatography on silica gel (PE) to get rid of the majority of side products. Both isomers can be successfully isolated and separated *via* a reverse phase column chromatography on C18.

*Note: The condition was optimized for the dienylation direction. However, in his specific case both products were observed. The reaction indeed favored allylation instead of dienylation with the chemoselectivety between dienylation and allylation determined to be 0.59:1. We rationalized that allylic ester was not stable under Pd catalytic condition, which lead to the formation of π-ally Pd intermediate. The competition* *raised this way caused the observed result.*

Characterization data for dienylic product:

##### allyl 2-(4-(2-(4-chlorophenyl)-5-methyl-4-methylene-1-(tetrahydro-2*H*-pyran-4-yl)hex-5-en-2-yl)phenoxy)-2-methylpropanoate (8-1)

[(See spectra](#_1H_NMR_Spectrum_93))

The title compound was collected as colorless oil (33.1 mg, 32%).

**TLC** (SiO_2_): R*_f_* = 0.22 (PE:EtOAc = 10:1)

**^1^H NMR (400 MHz, CDCl_3_)** δ 7.24 – 7.18 (m, 2H), 7.11 – 7.06 (m, 2H), 7.02 – 6.96 (m, 2H), 6.78 – 6.70 (m, 2H), 5.35 – 5.23 (m, 2H), 5.23 – 5.20 (m, 1H), 5.00 – 4.98 (m, 2H), 4.97 – 4.90 (m, 2H), 4.87 – 4.84 (m, 2H), 3.79 – 3.67 (m, 2H), 3.18 (dd, *J* = 11.6, 2.4 Hz, 1H), 3.13 (dd, *J* = 11.7, 2.3 Hz, 1H), 2.90 – 2.77 (m, 2H), 1.96 (d, *J* = 4.9 Hz, 2H), 1.90 (dd, *J* = 1.0 Hz, 3H), 1.58 (s, 6H), 1.45 – 1.34 (m, 1H), 1.23 – 1.06 (m, 2H), 1.02 (ddq, *J* = 13.3, 4.5, 2.4 Hz, 1H), 0.95 (ddq, *J* = 13.4, 4.5, 2.3 Hz, 1H).

**^13^C NMR (101 MHz, CDCl_3_)** δ 173.9, 153.6, 147.1, 141.7, 141.6, 140.4, 134.6, 131.7, 129.5, 128.7, 128.0, 119.0, 118.0, 115.4, 113.6, 79.3, 68.12, 68.10, 65.8, 48.6, 44.6, 43.2, 35.0, 34.9, 31.2, 25.5, 21.0.

**HRMS (pos. APCI)** m/z: [M+H]^+^ Calcd for C_32_H_40_O_4_^35^Cl 523.2610. Found 523.2604.

Characterization data for allylic product:

##### allyl 2-(4-(1-(4-chlorophenyl)vinyl)phenoxy)-2-methylpropanoate ()

[(See spectra](#_1H_NMR_Spectrum_94))

The title compound was collected as colorless oil (52.2 mg, 54%).

**TLC** (SiO_2_): R*_f_* = 0.44 (PE:EtOAc = 40:1)

**^1^H NMR (400 MHz, CDCl_3_)** δ 7.23 – 7.18 (m, 2H), 7.11 – 7.06 (m, 2H), 7.02 – 6.97 (m, 2H), 6.77 – 6.71 (m, 2H), 5.86 (ddt, *J* = 17.2, 10.5, 5.8 Hz, 1H), 5.35 – 5.24 (m, 2H), 5.21 (dq, *J* = 10.5, 1.3 Hz, 1H), 4.97 – 4.89 (m, 2H), 4.65 (dt, *J* = 5.7, 1.4 Hz, 2H), 3.78 – 3.67 (m, 2H), 3.17 (td, *J* = 11.6, 2.4 Hz, 1H), 3.14 (td, *J* = 11.7, 2.4 Hz, 1H), 2.86 (ddt, *J* = 13.5, 7.0, 1.2 Hz, 1H), 2.81 (ddt, *J* = 13.9, 6.9, 1.3 Hz, 1H), 2.02 – 1.91 (m, 2H), 1.58 (s, 6H), 1.40 (tt, *J* = 11.3, 4.2 Hz, 1H), 1.23 – 1.06 (m, 2H), 1.03 (ddq, *J* = 13.3, 4.4, 2.3 Hz, 1H), 0.94 (ddq, *J* = 13.3, 4.4, 2.3 Hz, 1H).

**^13^C NMR (101 MHz, CDCl_3_)** δ 173.9, 153.6, 147.1, 141.6, 134.6, 131.7, 129.5, 128.7, 128.0, 118.9, 118.8, 118.0, 79.3, 68.11, 68.09, 65.9, 48.6, 44.6, 43.2, 35.0, 34.9, 31.2, 25.5.

**HRMS (pos. APCI)** m/z: [M+H]^+^ Calcd for C_29_H_36_O_4_^35^Cl 483.2297. Found 483.2293.

Please see [here](#_2-(11-methylene-6,11-dihydrodibenzo) and [here](#_2-(11-allyl-11-isobutyl-6,11-dihydr) for the synthesis and characterization data for Compound **2-44** and **4-44**.

##### allyl 2-(11-allyl-11-isobutyl-6,11-dihydrodibenzo[*b,e*]oxepin-2-yl)acetate (9)

[(See spectra](#_1H_NMR_Spectrum_95))

The reaction was performed following a modified literature report. To an oven dried 8 mL Schlenk tube equipped with a magnetic stirring bar was charged with [Pd(allyl)]_2_Cl_2_ (3.7 mg, 0.01 mmol, 5.0 mol%) and *rac*-BINAP (14.9 mg, 0.024 mmol, 12.0 mol%) in air. The tube was evacuated and refilled with argon for three times before toluene was added. The tube was then sealed with a screw cap and allowed to stir for 30 min before Cs_2_CO_3_ (6.5 mg, 0.02 mmol, 10 mol%), Isoxepac derivative (70.1 mg, 0.2 mmol, 1.0 equiv.) and the stock solution of Propa-1,2-diene (5.0 equiv.) was subjected to the reaction mixture under an argon counterflow. The resulting mixture was allowed to stir for 16 h at 40 °C. The crude mixture was then cooled to room temperature and evaporated under reduced pressure. The residue was then redissolved in CDCl_3_ and subjected to ^1^H NMR measurement. The chemoselectivity between allylic product and dienylic product was determined to be 8.3:1 according to the NMR result. The sample was taken to dryness and the residue was purified by flash column chromatography on silica gel (PE:EtOAc = 40:1) to afford the title compound as a colorless oil (68.2 mg, 87%). The dienylic product was not characterized.

**TLC** (SiO_2_): R*_f_* = 0.25 (PE:EtOAc = 40:1)

**^1^H NMR (500 MHz, CDCl_3_)** δ 7.59 (dd, *J* = 8.2, 1.2 Hz, 1H), 7.46 (d, *J* = 2.1 Hz, 1H), 7.31 (ddd, *J* = 8.4, 7.2, 1.6 Hz, 1H), 7.15 (td, *J* = 7.4, 1.1 Hz, 1H), 7.08 – 7.02 (m, 2H), 6.93 (d, *J* = 8.1 Hz, 1H), 5.91 (ddt, *J* = 17.2, 10.4, 5.7 Hz, 1H), 5.42 (ddt, *J* = 17.2, 10.3, 7.0 Hz, 1H), 5.28 (dq, *J* = 17.2, 1.5 Hz, 1H), 5.21 (dq, *J* = 10.4, 1.3 Hz, 1H), 5.02 (d, *J* = 13.6 Hz, 1H), 4.98 (d, *J* = 13.6 Hz, 1H), 4.78 (ddt, *J* = 10.2, 2.1, 1.1 Hz, 1H), 4.70 (ddd, *J* = 17.1, 2.3, 1.2 Hz, 1H), 4.60 (dt, *J* = 5.7, 1.4 Hz, 2H), 3.63 (s, 2H), 2.98 (ddt, *J* = 14.3, 7.1, 1.3 Hz, 1H), 2.84 (ddt, *J* = 14.4, 6.9, 1.3 Hz, 1H), 2.31 (dd, *J* = 14.4, 5.7 Hz, 1H), 2.08 (dd, *J* = 14.4, 5.7 Hz, 1H), 1.44 – 1.31 (m, 1H), 0.54 (d, *J* = 6.7 Hz, 3H), 0.51 (d, *J* = 6.7 Hz, 3H).

**^13^C NMR (126 MHz, CDCl_3_)** δ 171.4, 161.1, 143.8, 138.8, 135.4, 134.4, 132.1, 131.7, 129.5, 128.6, 128.0, 127.8, 127.7, 125.9, 121.2, 118.4, 117.4, 77.0, 65.5, 55.1, 53.5, 51.3, 41.1, 25.4, 24.15, 24.06.

**HRMS (pos. APCI)** m/z: [M+H]^+^ Calcd for C_26_H_30_O_3_ 391.2268. Found 391.2278.

## Mechanistic investigation

### Radical capture experiments

Allylation-Stand. Cond. 1

The reaction was performed following ***GP 3*** with 1.5 equiv. of TEMPO as the additive. Upon completion, the reaction mixture was filtered through a pad a silica, evaporated under reduced pressure and measured by ^1^H NMR with 2-picoline as the internal standard. The formation of product was completely shut down judged by NMR analysis. Instead, the existence of TEMPO-iPr adduct was confirmed GC-HRMS study.

1-isopropoxy-2,2,6,6-tetramethylpiperidine

**HRMS (pos. APCI)** m/z: [M+H]^+^ Calcd for C_12_H_26_NO 200.2009. Found 200.2008.

Allylation-Stand. Cond. 2

The reaction was performed following ***GP 4*** with 1.5 equiv. of TEMPO as the additive. Upon completion, the reaction mixture was filtered through a pad a silica, evaporated under reduced pressure and measured by ^1^H NMR with 2-picoline as the internal standard. The formation of product was completely shut down judged by NMR analysis. Instead, the existence of TEMPO-iPr adduct was confirmed GC-HRMS study.

1-isopropoxy-2,2,6,6-tetramethylpiperidine

**HRMS (pos. APCI)** m/z: [M+H]^+^ Calcd for C_12_H_26_NO 200.2009. Found 200.2006.

Dienylation

The reaction was performed following a slightly modified ***GP 5*** with 1.2 equiv. DHP instead. In addition, 1.2 equiv. and 2.4 equiv. of TEMPO was subjected to the reaction mixture as the radical trapping reagent, respectively. Upon completion, the reaction mixture was filtered through a pad a silica, evaporated under reduced pressure and measured by ^1^H NMR with 2-picoline as the internal standard. When 1.2 equiv. of TEMPO was added, the yield of **5** dropped to 22%. Meanwhile, the TEMPO-diene adduct (**10**) was detected in 37% yield based on ^1^H NMR analysis. The isolation and characterization of **10** was successfully conducted. In the other case, when 2.4 equiv. of TEMPO was added, the formation of product was completely shut down judged by NMR analysis. Instead, the existence of TEMPO-iPr adduct was confirmed GC-HRMS study.

1-isopropoxy-2,2,6,6-tetramethylpiperidine

**HRMS (pos. APCI)** m/z: [M+H]^+^ Calcd for C_12_H_26_NO 200.2009. Found 200.2007.

##### allyl 2-(11-allyl-11-isobutyl-6,11-dihydrodibenzo[*b,e*]oxepin-2-yl)acetate (10)

[(See spectra](#_1H_NMR_Spectrum_96))

The title compound purified via flash column chromatography on silica gel (PE:EtOAc = 10:1) to afford the title compound as colorless oil (3.5 mg, 7%).

**TLC** (SiO_2_): R*_f_* = 0.25 (PE:EtOAc = 10:1)

**^1^H NMR (500 MHz, CDCl_3_, 253 K)** δ 5.45 – 5.41 (m, 1H), 5.27 – 5.23 (m, 1H), 4.98 (s, 1H), 4.96 (q, *J* = 1.4 Hz, 1H), 4.52 (t, *J* = 1.5 Hz, 2H), 1.94 (d, *J* = 1.2 Hz, 3H), 1.65 – 1.53 (m, 1H), 1.52 – 1.46 (m, 4H), 1.40 – 1.33 (m, 2H), 1.19 (s, 6H), 1.15 (s, 6H).

**^13^C NMR (126 MHz, CDCl_3_, 253 K)** δ 143.1, 140.8, 112.2, 111.9, 76.2, 59.9, 39.3, 32.8, 21.1, 20.4, 17.0.

**HRMS (pos. APCI)** m/z: [M+H]^+^ Calcd for C_15_H_28_NO 238.2165. Found 238.2169.

### Deuterium labeling experiments

Allylation-Stand. Cond. 1

The reaction was performed following ***GP 3*** with 1.5 equiv. Deuterated iPr-DHP (**3-1***-d*). The crude mixture was first roughly purified though column chromatography on silica gel (PE) to get rid of the majority of polar side products. The residue was then carefully purified by a reverse phase column chromatography on C18 to afford the title compound as a colorless oil (27.3 mg, 52%). The deuterium incorporation was exclusive on the internal site of the vinyl moiety with a D/H ratio of 56% determined by ^1^H NMR analysis.

###### ^1^H NMR of **4-1***-d* (400 MHz, CDCl_3_)

Allylation-Stand. Cond. 2

The reaction was performed following ***GP 4*** with 1.5 equiv. Deuterated iPr-DHP (**3-1***-d*). The crude mixture was first roughly purified though column chromatography on silica gel (PE) to get rid of the majority of polar side products. The residue was then carefully purified by a reverse phase column chromatography on C18 to afford the title compound as a colorless oil (26.4 mg, 50%). The deuterium incorporation was exclusive on the internal site of the vinyl moiety with a D/H ratio of 64% determined by ^1^H NMR analysis.

###### ^1^H NMR of **4-1***-d* (500 MHz, CDCl_3_)

Dienylation

The reaction was performed following ***GP 5*** with 1.2 equiv. Deuterated iPr-DHP (**3-1***-d*). The crude mixture was first roughly purified though column chromatography on silica gel (PE) to get rid of the majority of polar side products. The residue was then carefully purified by a reverse phase column chromatography on C18 to afford the title compound as a colorless oil (24.7 mg, 41%). The deuterium incorporation was exclusive on the methyl group of the diene moiety with a D/H ratio of 47% determined by ^1^H NMR analysis.

###### ^1^H NMR of **5-1***-d* (500 MHz, CDCl_3_)

### Stochiometric experiment

To an oven dried 8 mL Schlenk tube equipped with a magnetic stirring bar was charged with [PdAllyl]_2_Cl_2_ (18.3 mg, 0.05 mmol, 0.5 equiv.), Xantphos (69.4 mg, 0.12 mmol, 1.2 equiv.) and [Ir(ppy)_2_dtbbpy]PF_6_ (0.5 mg, 0.0005 mmol, 0.5 mol%) in air. The tube was evacuated and refilled with argon for three times before toluene (1 mL) was added. The tube was then sealed with a screw cap and the mixture was allowed to stir for 30 min before 4-CF_3_-BzOH (1.9 mg, 0.01 mmol, 10 mol%), **2-1** (31.8 µL, 0.18 mmol, 1.8 equiv.), **3-1** (44.3 mg, 0.15 mmol, 1.5 equiv.) were subjected to the reaction mixture under an argon counterflow. The resulting mixture was allowed to stir for 24 h under the irradiation of a 16 W Blue LEDs strip. A cooling fan was place above the tubes to keep the temperature at room temperature during the reaction. Upon completion, the crude mixture was filtered through a pad a silica, evaporated under reduced pressure and subjected to ^1^H NMR analysis with 2-picolin as the internal standard. The yield was determined to be 9% based on the proton NMR study.

###### ^1^H NMR of **4-1** (300 MHz, CDCl_3_)

### Post dienylation

| **Entry** | **Variations** | **Dienylic/%** | **Allylic (remain)/%** |
| --- | --- | --- | --- |
| 1 | - | n.d. | Quanti. |
| 2 | w/ 1.0 equiv. Acid | n.d. | Quanti. |
| 3 | w/ 1.2 equiv. iPr-DHP | n.d. | Quanti. |
| 4 | w/ 1.2 equiv. iPr-DHP and 1.0 equiv. Acid | n.d. | Quanti. |
| 5 | w/ 1.2 equiv. HEH | n.d. | Quanti. |
| 6 | w/ 1.2 equiv. HEH and 1.0 equiv. Acid | n.d. | Quanti. |

Reaction condition: Propa-1,2-diene (0.1 mmol, 1.0 equiv.), **4-1** (1.5 equiv.), , [Ir(ppy)_2_dtbbpy]PF_6_ (0.5 mol%), Pd(OAc)_2_ (7.5 mol%), (2-furyl)_3_P (18 mol%), 4-OMe-Py·TsOH (10 mol%) in toluene (1 mL) were irradiated with 16 W blue LEDs for 16 h under argon.

The results illustrated that the diene moiety was constructed around the Pd center as a whole rather than a post functionalization of allylic product.

## DFT calculation

### General Remarks

All DFT calculations have been carried out using Gaussian16.^[4]^ All geometries were optimized in the gas phase using the B3LYP^[5]^ functional in combination with the 6-31G(d,p) basis set^[6]^ for all atoms except palladium for which the SDD^[7]^ pseudopotential was applied. In all cases the ultrafine integral grid was employed (int=grid=ultrafine), moreover, we used the scf=tight keyword for all structures. During the geometry optimization we considered dispersion energies by the use of the Grimme D3 correction together with Becke-Johnson damping (keyword EmpiricalDispersion=GD3BJ).^[8]^ Frequency calculations were performed in order to obtain thermal corrections at 298 K. All optimized structures of intermediated showed no imaginary frequencies during vibrational analysis and all optimized transition states showed only one imaginary frequencies during vibrational analysis. All other transition states were obtained without restricted coordinates and confirmed by IRC calculations. In case the transition state could only be obtained with fixed coordinates (keyword=modredundant), this was indicated in the list of atomic coordinates and in the discussion in brackets. Single-point energy calculations were performed on the optimized geometries using the B3LYP^[5]^ functional and the def2QZVP^[9]^ basis set for all atoms, except palladium, for which the SDD pseudopotential was employed. These calculations included the Grimme D3 dispersion correction with Becke–Johnson damping.^[8]^ Additionally, solvation effects were modeled using the SMD solvent model with toluene as the solvent.^[10]^ All visualizations of calculated structures were prepare using CYLview.^[11]^ In the following discussion HE is used as abbreviation for Hantzsch ester and BzOH as abbreviation for 4-chloro-benzoic acid.

### Mechanistic Discussion

#### Allylation

To investigate the reaction mechanism leading to the formation of the allylic product, we explored three outer-sphere pathways (Figure 3, Figure 4 and Figure 5) as well as an inner-sphere mechanism for Xantphos (Figure 6). Besides this, we explored three outer-sphere mechanisms using the P(2-furyl)_3_ ligand.

The first considered outer-sphere mechanism (Figure 3) starts from [Pd^0^(Xantphos)] by the oxidative addition of BzOH (**TS-1**)to generate a palladium hydride species (**Int-1**). After coordination of propadiene **Int-2** is formed, which undergoes hydrometalation via **TS-2** to afford the π-allyl complex **Int-3**, which then can subsequently undergo single-electron transfer (SET) reduction by the iridium photocatalyst to generate the Pd(I) species **Int-4** which is 11.7 kcal/mol higher in energy. The radical **Rad·**, previously formed by the photocatalyst, can then attack **Int-4** via an outer-sphere mechanism (**TS-3**, fixed coordinates between C77 and C88) to form the product complex **Int-5**.


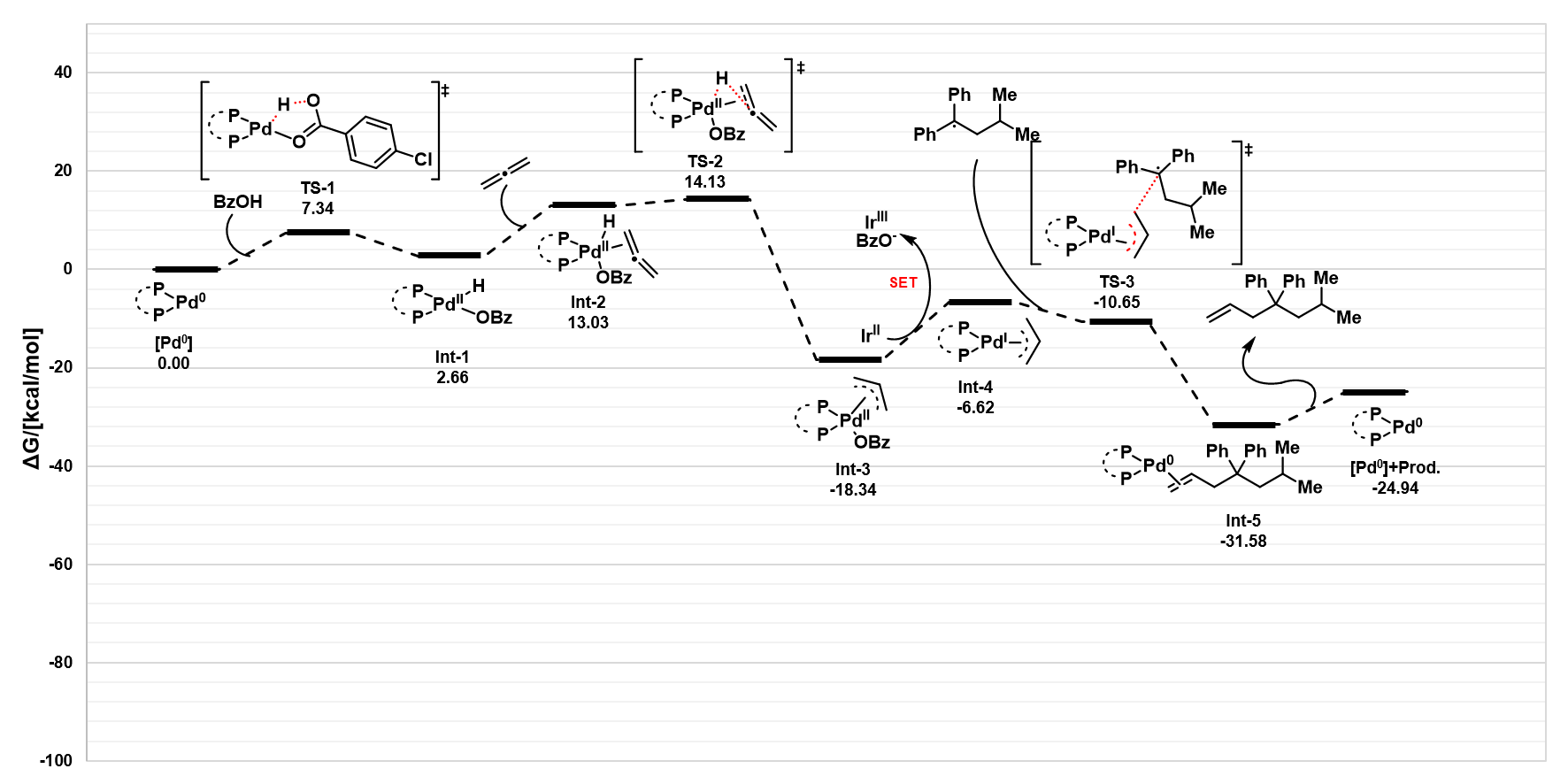


Figure 3 Calculated ΔG profile (B3LYP/def2qzvp/SDD/D3BJ/SMD(toluene)//B3LYP/6-31G(d,p)/SDD/D3BJ) for the formation of the allylic product via an outer-sphere attack (**TS-3**) on a [(π-allyl)Pd^I^(Xantphos)] complex (**Int-4**). This complex was formed before via sequence of oxidative addition (**TS-1**), hydrometalation (**TS-2**) and SET reduction by the photocatalyst.

Figure 4 shows an alternative route to **Int-3**. Starting with the formation of the π-complex (**Int-6**) via propadiene coordination and proceeding with the direct protonation of the coordinated propadiene (**TS-4**), **Int-3** can also be accessed. However, the pathway towards **Int-3** initiated by oxidative addition is energetically more favorable, with a difference of 5.3 kcal/mol.


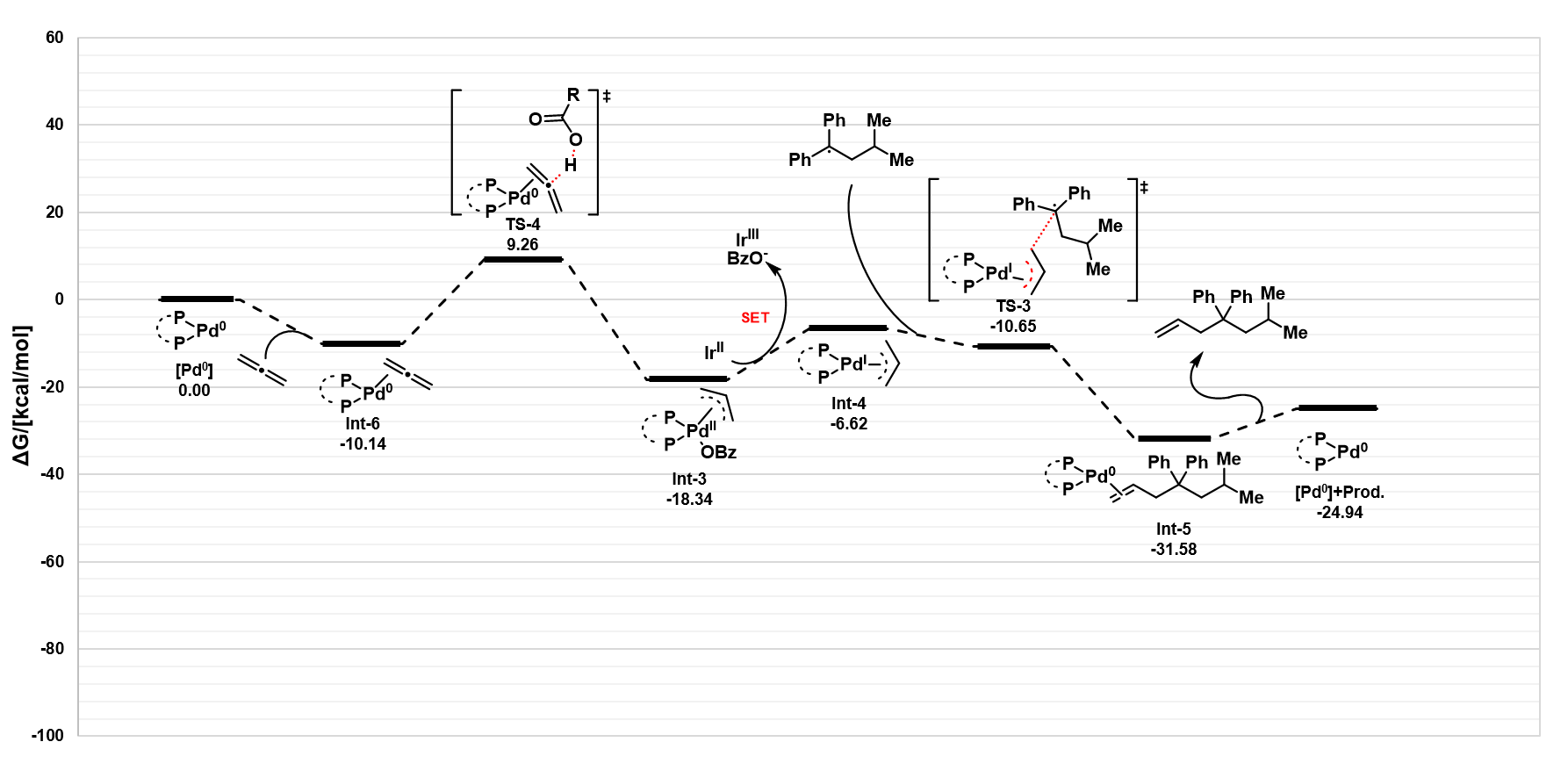


Figure 4 Calculated ΔG profile (B3LYP/def2qzvp/SDD/D3BJ/SMD(toluene)//B3LYP/6‑31G(d,p)/SDD/D3BJ) for the formation of the allylic product via an outer-sphere attack (**TS-3**) on a [(π-allyl)Pd^I^(Xantphos)] complex (**Int-4)**. This complex can also be formed from a [(propadiene)Pd(Xantphos)] complex (**Int-6**) via direct protonation (**TS-4**) followed by SET reduction by the photocatalyst.

As a mechanistic alternative, we considered the direct outer-sphere attack of **Rad·** on **Int-3** via **TS-5** (Figure 5). The activation barrier for this Pd(II) pathway is 3.9 kcal/mol higher in energy compared to the energy barrier for Pd(I) pathway between **Int-3** and **TS-3**. The resulting Pd(I) product complex **Int-7** can then release the product and the formed [(BzO)Pd^I^(Xantphos)] complex (**Int-8**) can then be reduced subsequently by the iridium photocatalyst to regenerate the palladium catalyst.


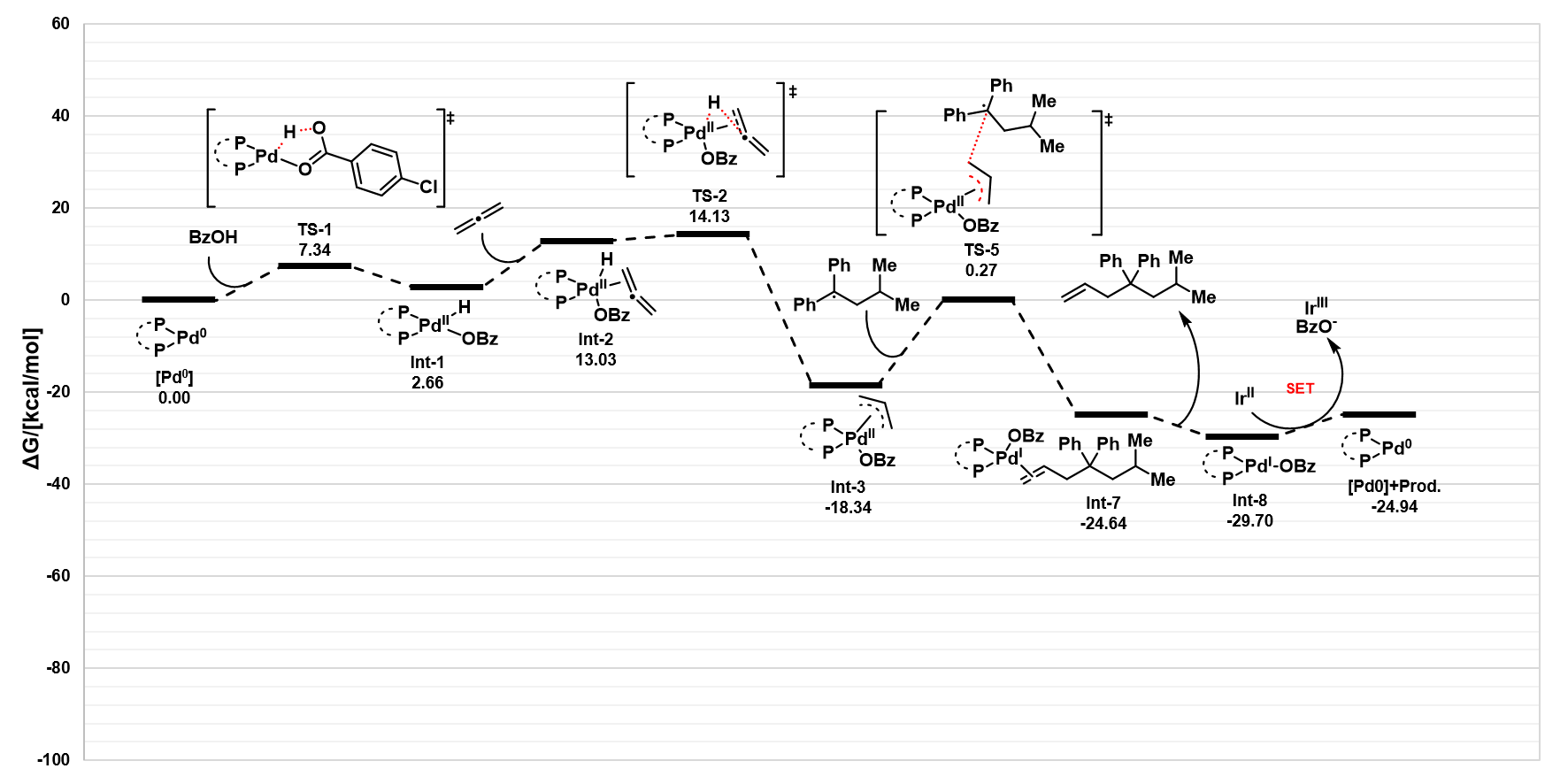


Figure 5 Calculated ΔG profile (B3LYP/def2qzvp/SDD/D3BJ/SMD(toluene)//B3LYP/6-31G(d,p)/SDD/D3BJ) for the formation of the allylic product via an outer-sphere attack (**TS-5**) on a [(BzO)(π-allyl)Pd^II^(Xantphos)] complex (**Int-3**).

Another mechanistic possibility for the Xantphos ligand involves the direct coordination of the radical **Rad·** to **Int-4**, forming the Pd(II) species **Int-9** (Figure 6). From this intermediate, the product can be generated via an inner-sphere reductive elimination through **TS-6**. This pathway is significantly less favorable compared to the Pd(I) as well as the Pd(II) outer-sphere attacks (**TS-3** and **TS-5**).

Additionally, The Pd(III) mediated inner-sphere reductive elimination was also considered. However, we couldn’t find the corresponding intermediates with both benzyl and allyl group attached to Pd(III) center. Computationally, we found that during the geometry optimization, the benzyl radical either directly attacked the Pd(II)-allyl moiety through an outer-sphere pathway or stay at the addition of their Van der Waals radius and couldn’t get closer. The results were collected with or without the counter anion coordinated to Pd(II). We assume that the steric bulkiness might hinder the coordination of the benzyl radical to a more constrained Pd(III) center. Since the allocation of Pd(III)-allyl/benzyl complex was not success, the following reductive eliminations were also considered unlikely.


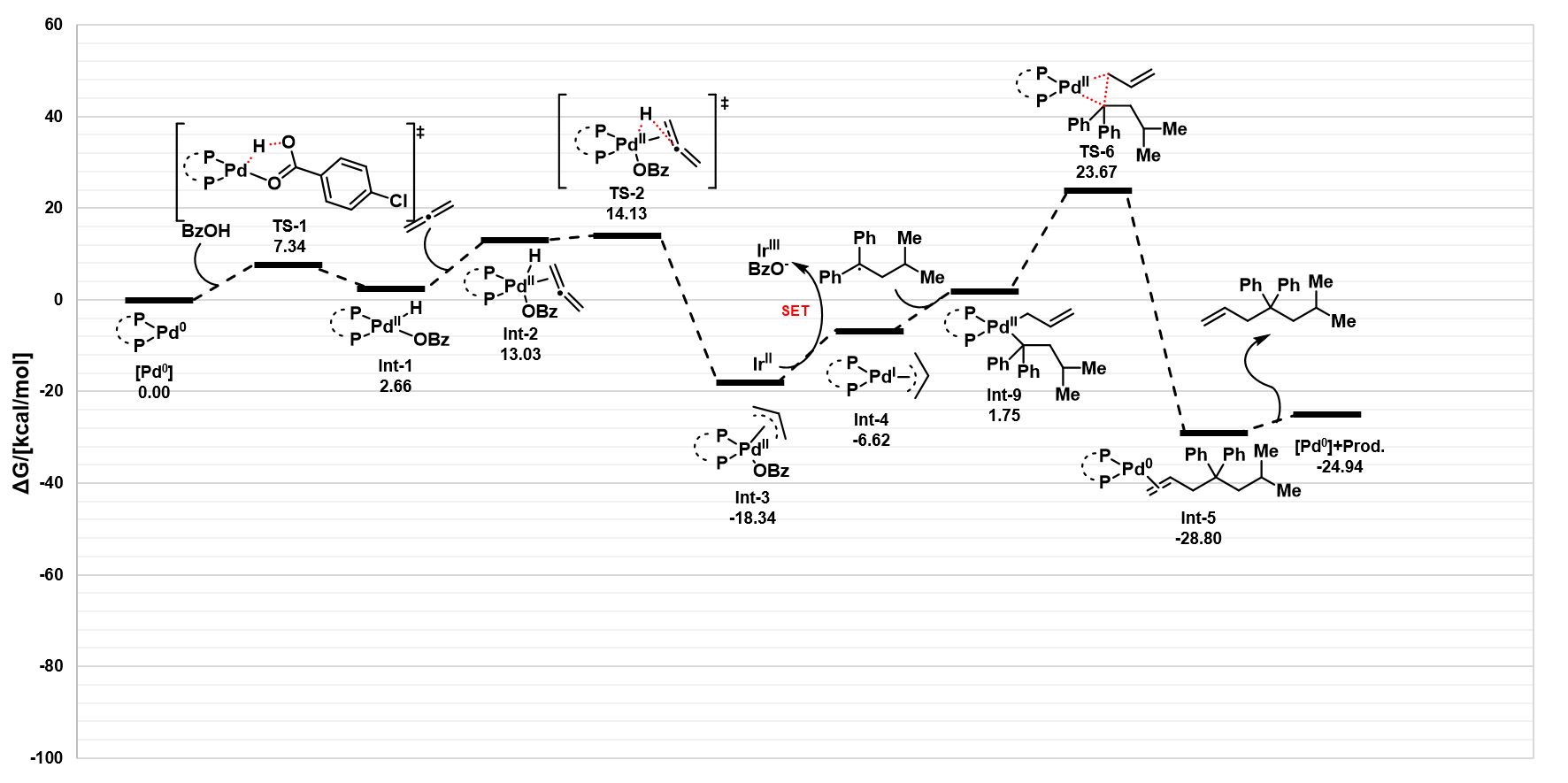


Figure 6 Calculated ΔG profile (B3LYP/def2qzvp/SDD/D3BJ/SMD(toluene)//B3LYP/6-31G(d,p)/SDD/D3BJ) for the formation of the allylic product via reductive elimination (**TS-6**) from a [(R)(σ-allyl)Pd^II^(Xantphos)] complex (**Int-9**). This complex was formed before via coordination of the radical to **Int-4**.

We then repeated our investigations using the P(2-furyl)₃ ligand (Figure 7, Figure 8 and Figure 9). The first considered pathway starts by coordination of a propadiene ligand forming **Int-10**. The propadiene ligand can then be protonated by BzOH (**TS-7**) producing the π-allyl complex **Int-11**. This intermediate can by reduced by the iridium photocatalyst to generate the Pd(I) species **Int-12**. Via an outer-sphere-attack of **Rad·** (**TS-8**) the product complex **Int-13** is formed. Subsequent release of the product regenerates the Pd(0) catalyst.


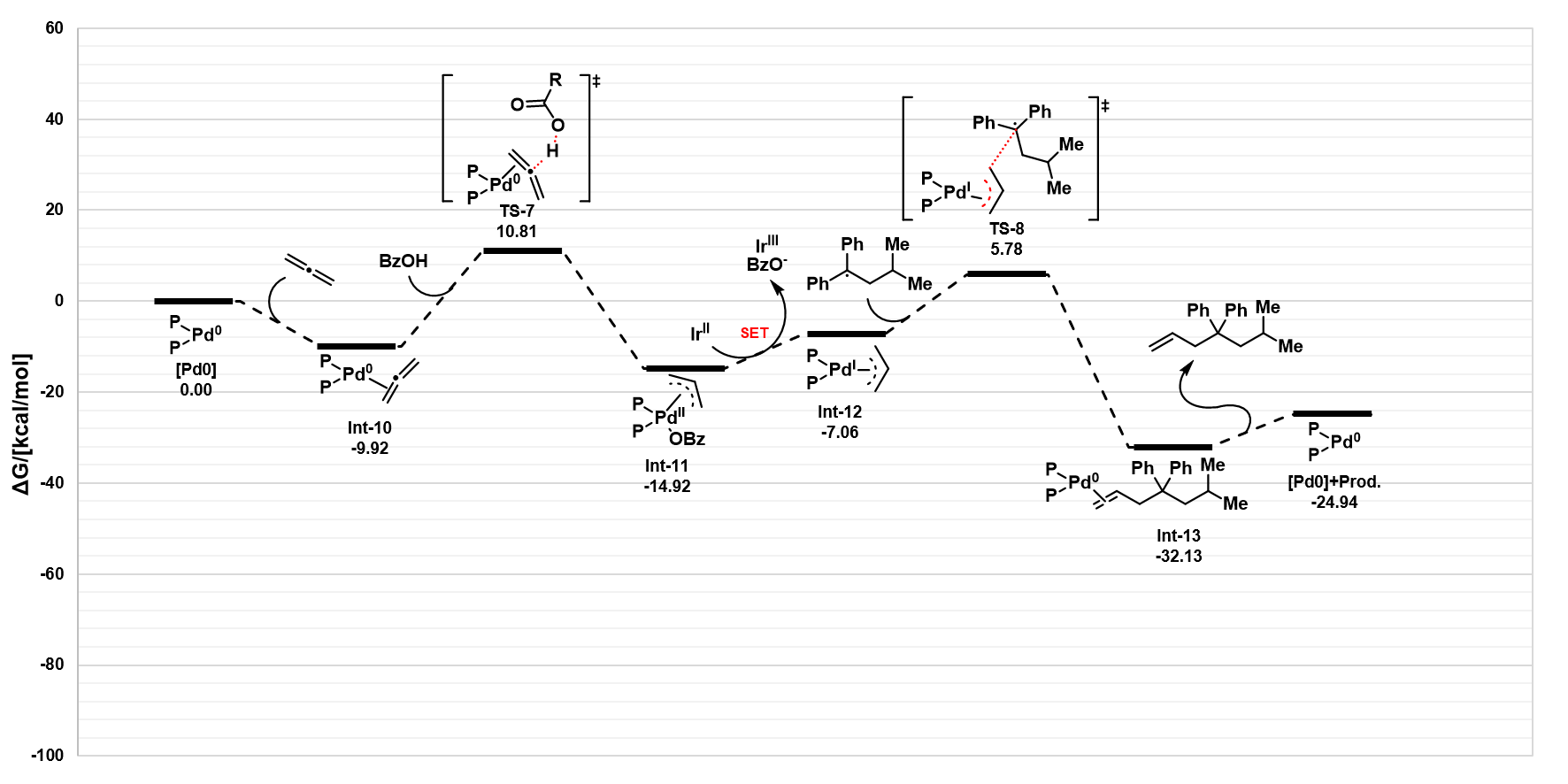


Figure 7 Calculated ΔG profile (B3LYP/def2qzvp/SDD/D3BJ/SMD(toluene)//B3LYP/6-31G(d,p)/SDD/D3BJ) for the formation of the allylic product via an outer-sphere attack (**TS-8**) on a [(π-allyl)Pd^I^(P(2-furyl)_3_)_2_] complex (**Int-12**). This complex was formed from a [(propadiene)Pd(P(2-furyl)_3_)_2_] complex (**Int-10**) via direct protonation (**TS-7**) followed by SET reduction by the photocatalyst.

The π-allyl complex **Int-11** can be accessed via an alternative route starting by oxidative addition of BzOH to **Int-10**. The formed palladium hydride complex can form **Int-11** via hydrometalation (**TS-10**). However, this pathway is 11.9 kcal/mol less favored compared to **TS-7**.


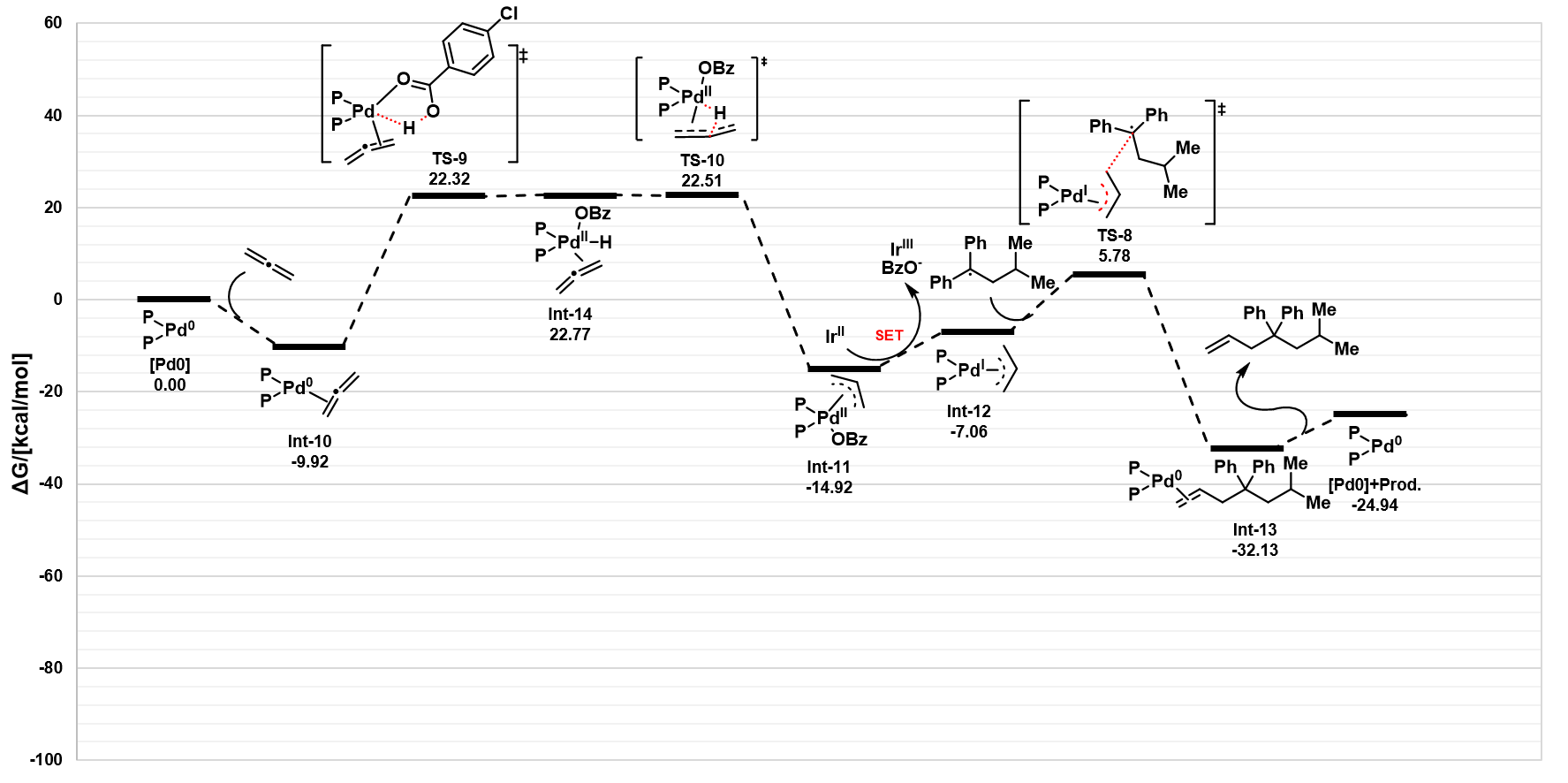


Figure 8 Calculated ΔG profile (B3LYP/def2qzvp/SDD/D3BJ/SMD(toluene)//B3LYP/6-31G(d,p)/SDD/D3BJ) for the formation of the allylic product via an outersphere attack (**TS-8**) on a [(π-allyl)Pd^I^(P(2-furyl)_3_)_2_] complex (**Int-12**). This complex can also be formed from [(propadiene)Pd(P(2-furyl)_3_)_2_] complex (**Int-10**) via a sequence of oxidative addition (**TS-9**) and hydrometallation (**TS-10**) followed by SET reduction by the photocatalyst.

Moreover, there is the possibility of an outer-sphere attack (**TS-11**) to the Pd(II) π-allyl complex **Int-11** yielding the Pd(I) product complex **Int-15**. After release of the allylic product **Int-16** can be reduced by the iridium photocatalyst to regenerate the palladium catalyst. **TS-11** is, however, 1.7 higher in energy compared to **TS-8**.


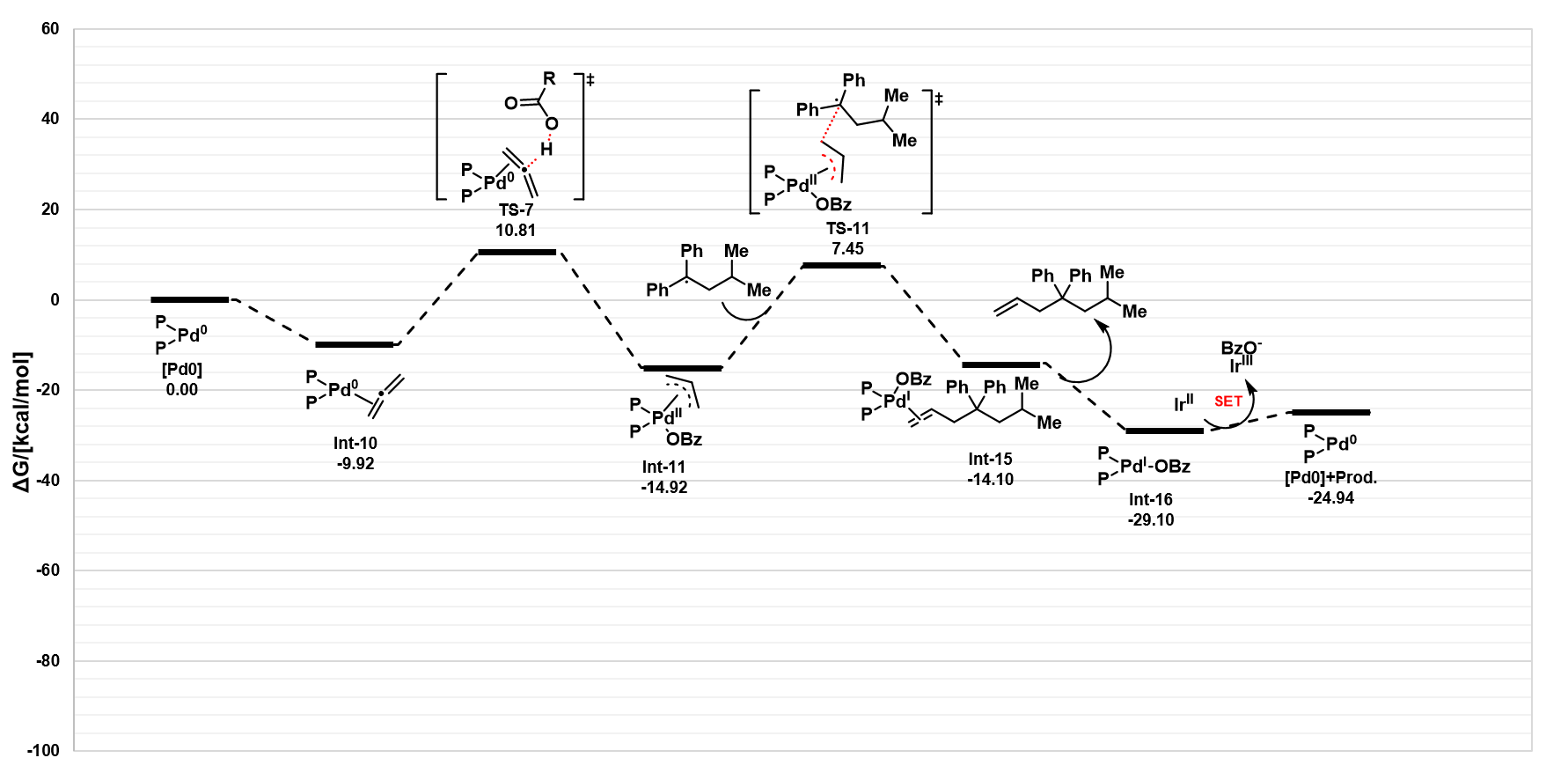


Figure 9 Calculated ΔG profile (B3LYP/def2qzvp/SDD/D3BJ/SMD(toluene)//B3LYP/6‑31G(d,p)/SDD/D3BJ) for the formation of the allylic product via an outer-sphere attack (**TS-11**) on a [(BzO)(π-allyl)Pd^II^(P(2-furyl)_3_)_2_] complex (**Int-11)**.

For both ligands an outer-sphere attack (see Figure 3 and Figure 7) of the radical **Rad·** on a Pd(I) π-allyl complex seems to be the most favored pathway towards the allylic product.

#### Dienylation (allylation conditions)

To obtain further insight on the origin of the experimentally observed chemoselectivity for the allylic product when BzOH is uses as acid additive, further DFT calculations were performed for Xantphos (Figure 10) and P(2-furyl)_3_ (Figure 11). Other mechanistic pathways towards the diene product will be later discussed. Here we discuss the later found lowest energy pathway with an exchanged acid additive (BzOH instead of PyH^+^). Starting with Xantphos by formation of **Int-1** via oxidative addition of BzOH (**TS-1**). After coordination of propadiene, the terminal position of the propadiene ligand can be hydrometallated (**TS-12**) yielding a methylvinyl complex **Int-17**. Ligand exchange of the benzoate with another propadiene leads to **Int-19**. This coordinated propadiene undergoes insertion into the Pd–C bond of the methylvinyl ligand through **TS-13**, generating **Int-20**, a palladium complex in which one face of the dienyl ligand adopts a π-allyl coordination mode. Intermediate **Int-20** can be reduced by the iridium photocatalyst to form the Pd(I) complex **Int-21**. An outer-sphere attack by the radical **Rad·** via **TS-14** (notably, this transition state could only be located by constraining the distance between C19 and C17) then forms the product complex **Int-22**, which releases the dienyl product and regenerates the palladium catalyst. The energy barrier between **Int-17** and **TS-13** as energetic span of this mechanism is with 55.5 kcal/mol very high compared to the before discussed mechanism leading to the allylic product. This is in agreement with high experimentally observed chemoselectivity.


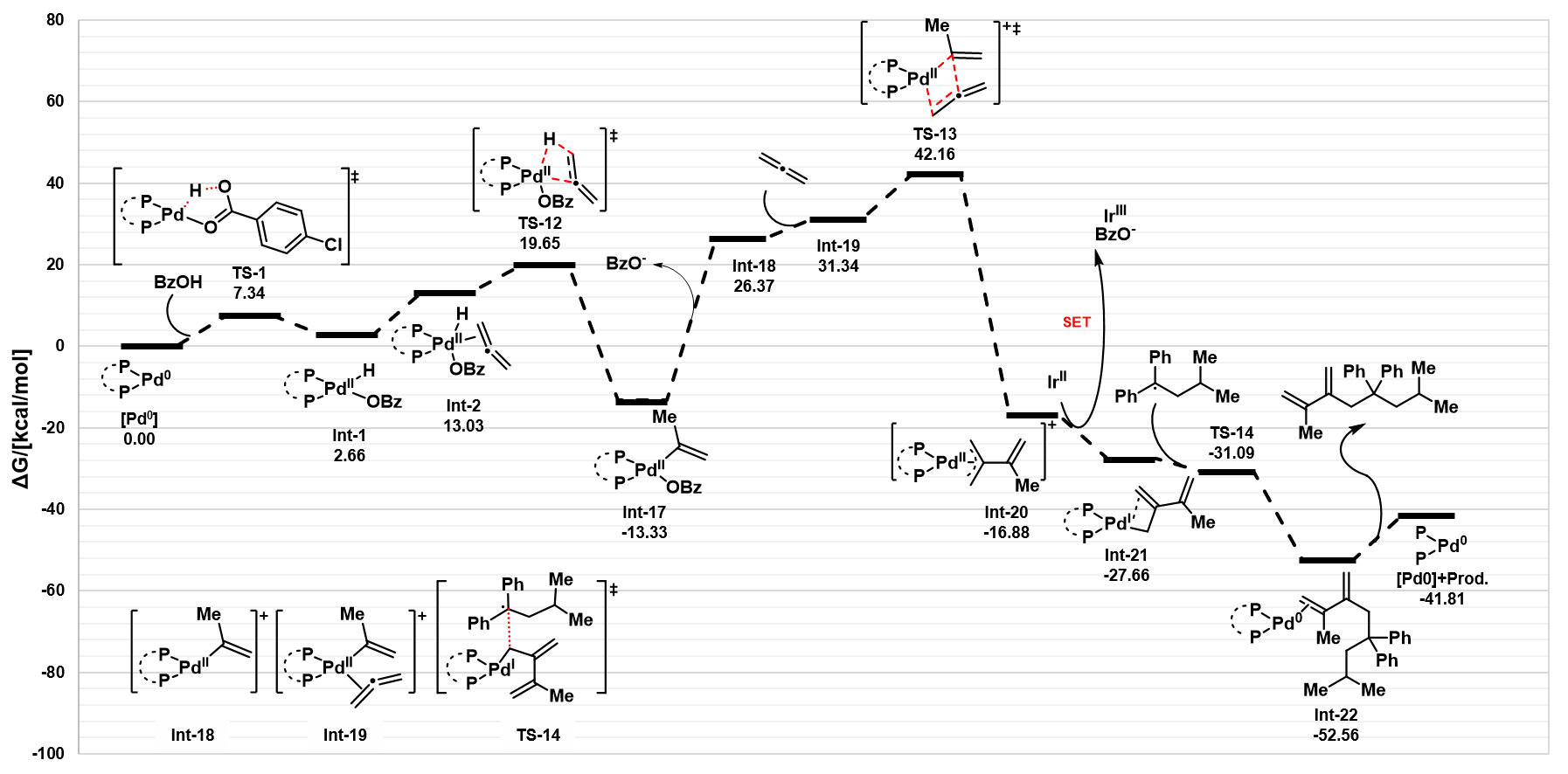


Figure 10 Calculated ΔG profile (B3LYP/def2qzvp/SDD/D3BJ/SMD(toluene)//B3LYP/6-31G(d,p)/SDD/D3BJ) for the formation of the diene product via an outersphere attack (**TS-14**) on [(σ-allyl)Pd^I^(Xantphos)] (**Int-21**) under the allylation conditions (BzOH as acid additive). **Int-20** was formed before via a sequence of oxidative addition (**TS-1**), allene coordination, hydrometalation (**TS-12**) to a methylvinyl complex (**Int-17**), ligand exchange, dienyl formation (**TS-13**) and SET reduction by the photocatalyst.

In case of the P(2-furyl)_3_ ligand the mechanism towards the dienyl product starts by propadiene coordination forming **Int-10**, followed by oxidative addition of BzOH (**TS-9**). The palladium hydride **Int-14** can then hydrometallate the terminal position of its propadiene ligand yielding the methylvinyl complex **Int-23**. After ligand exchange and insertion of the propadiene ligand into the Pd-C bond of the methylvinyl ligand (**TS-16**), **Int-26**, a palladium complex in which one face of the dienyl ligand adopts a π-allyl coordination mode, is formed. Intermediate **Int-26** can be reduced by the iridium photocatalyst to form the Pd(I) complex **Int-27**. An outer-sphere attack by the radical **Rad·** via **TS-17** then forms the product complex **Int-28**, which releases the dienyl product and regenerates the palladium catalyst. The energy barrier between **Int-23** and **TS-16** as energetic span of this mechanism is with 56.2 kcal/mol very high compared to the before discussed mechanism leading to the allylic product for this ligand. This is in agreement with high experimentally observed chemoselectivity.


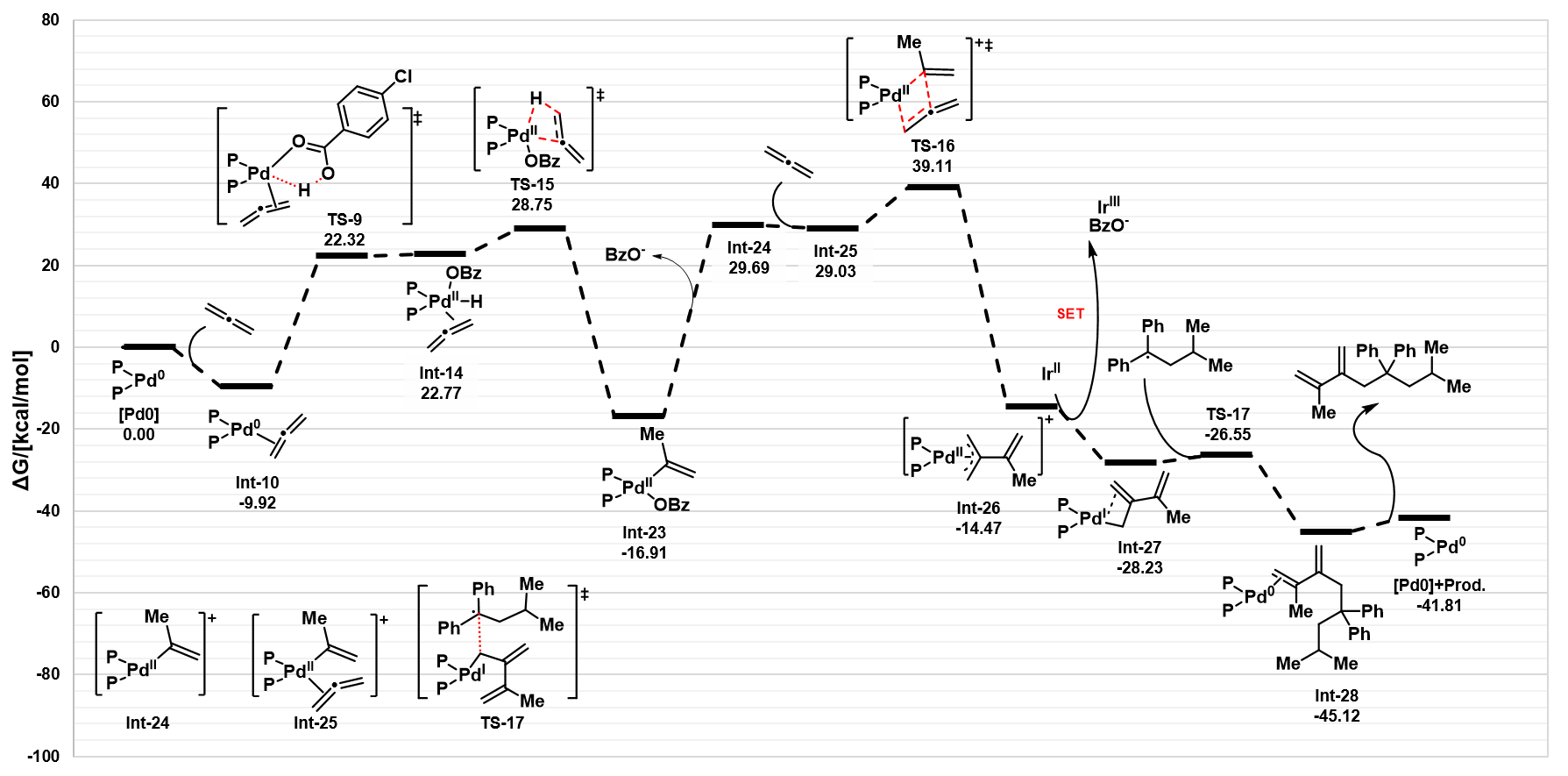


Figure 11 Calculated ΔG profile (B3LYP/def2qzvp/SDD/D3BJ/SMD(toluene)//B3LYP/6-31G(d,p)/SDD/D3BJ) for the formation of the diene product via an outersphere attack (**TS-17**) on [(σ-allyl)Pd^I^(P(2-furyl)_3_)_2_] (**Int-27**) under the allylation conditions (BzOH as acid additive). **Int-27** was formed before via a sequence of oxidative addition (**TS-9**), allene coordination, hydrometalation (**TS-15**) to a methylvinyl complex (**Int-23**), ligand exchange, dienyl formation (**TS-16**) and SET reduction by the photocatalyst.

#### Dienylation

Finally, we considered seven different mechanisms for P(2-furyl)_3_ and for Xantphos respectively. The first mechanism (Figure 12) starts with the P(2-furyl)_3_ ligand by coordination of propadiene to the palladium catalyst generating **Int-10**, followed by protonation of the terminal position of the propadiene ligand with PyH^+^ (**TS-18**) forming the cationic methylvinyl complex **Int-29**. After ligand exchange of pyridine by propadiene, this ligand can insert into the Pd-C bond of the methylvinyl ligand (**TS-16**), generating the π-allyl complex **Int-26**. After reduction with the iridium photocatalyst, an outer-sphere attack (**TS-17**) of the radical forms the product complex **Int-28**. The palladium catalyst is regenerated after release of the diene product.


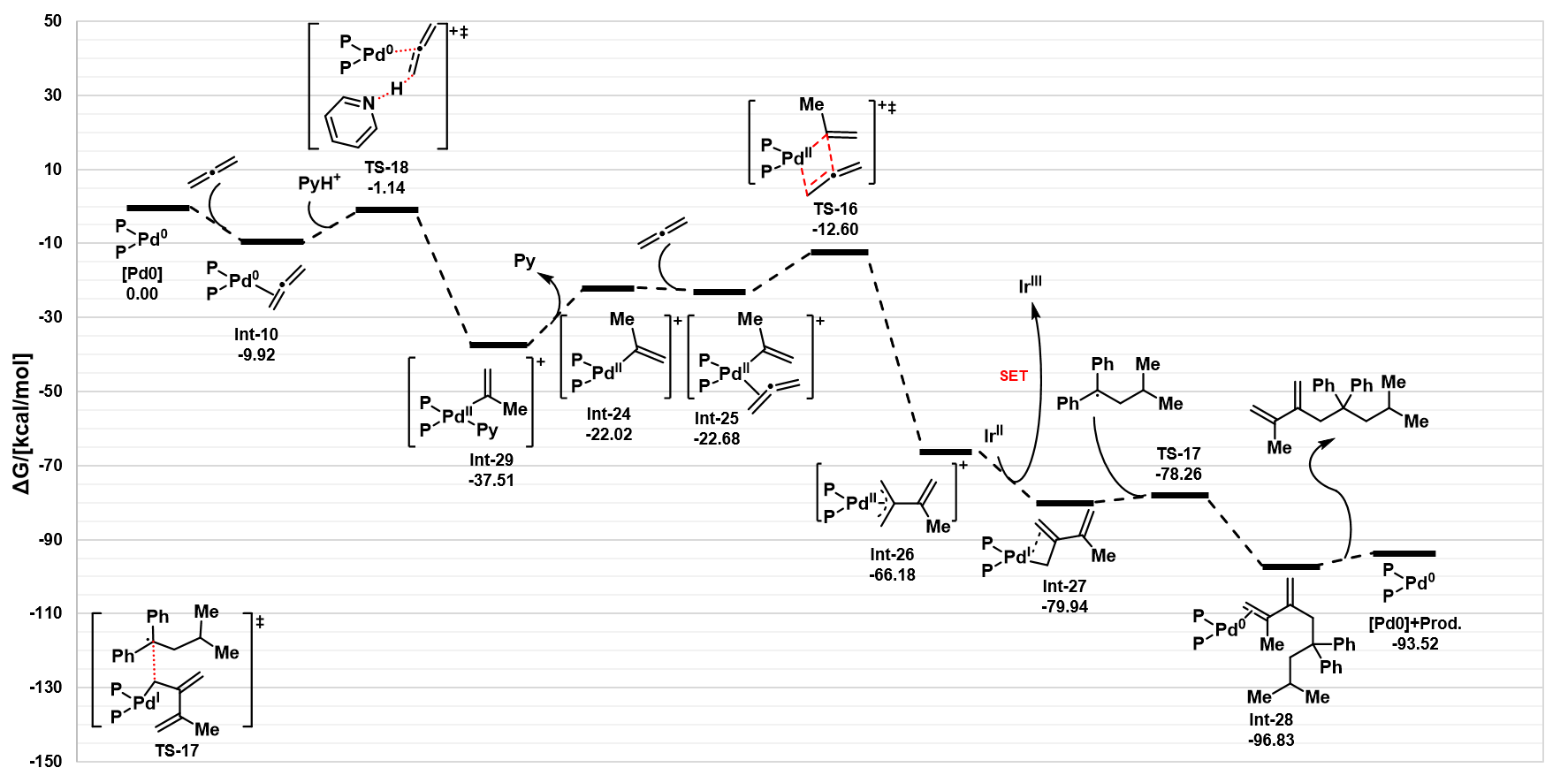


Figure 12 Calculated ΔG profile (B3LYP/def2qzvp/SDD/D3BJ/SMD(toluene)//B3LYP/6-31G(d,p)/SDD/D3BJ) for the formation of the diene product via an outersphere attack (**TS-17**) on [(σ-allyl)Pd^I^(P(2-furyl)_3_)_2_] (**Int-27**) under the dienylation conditions (PyH^+^ as acid additive). **Int-27** was formed before via a sequence of propadiene complex protonation (**TS-18**) to a methylvinyl complex (**Int-29**), ligand exchange, dienyl formation (**TS-16**) and SET reduction by the photocatalyst.

The second mechanism (Figure 13) is similar to the first one until the formation of **Int-29**, which is SET-reduced by the iridium photocatalyst to form **Int-30**. Following the ligand exchange of pyridine with propadiene to form **Int-32**, propadiene can be inserted into the Pd-C bond of the Pd(I) complex (**TS-19**), resulting in **Int-27**. From this intermediate, the mechanism is similar to the one previously discussed (Figure 12). This early reduction lowers the energetic span of this pathway by 3.9 kcal/mol compared to that discussed in Figure 12.


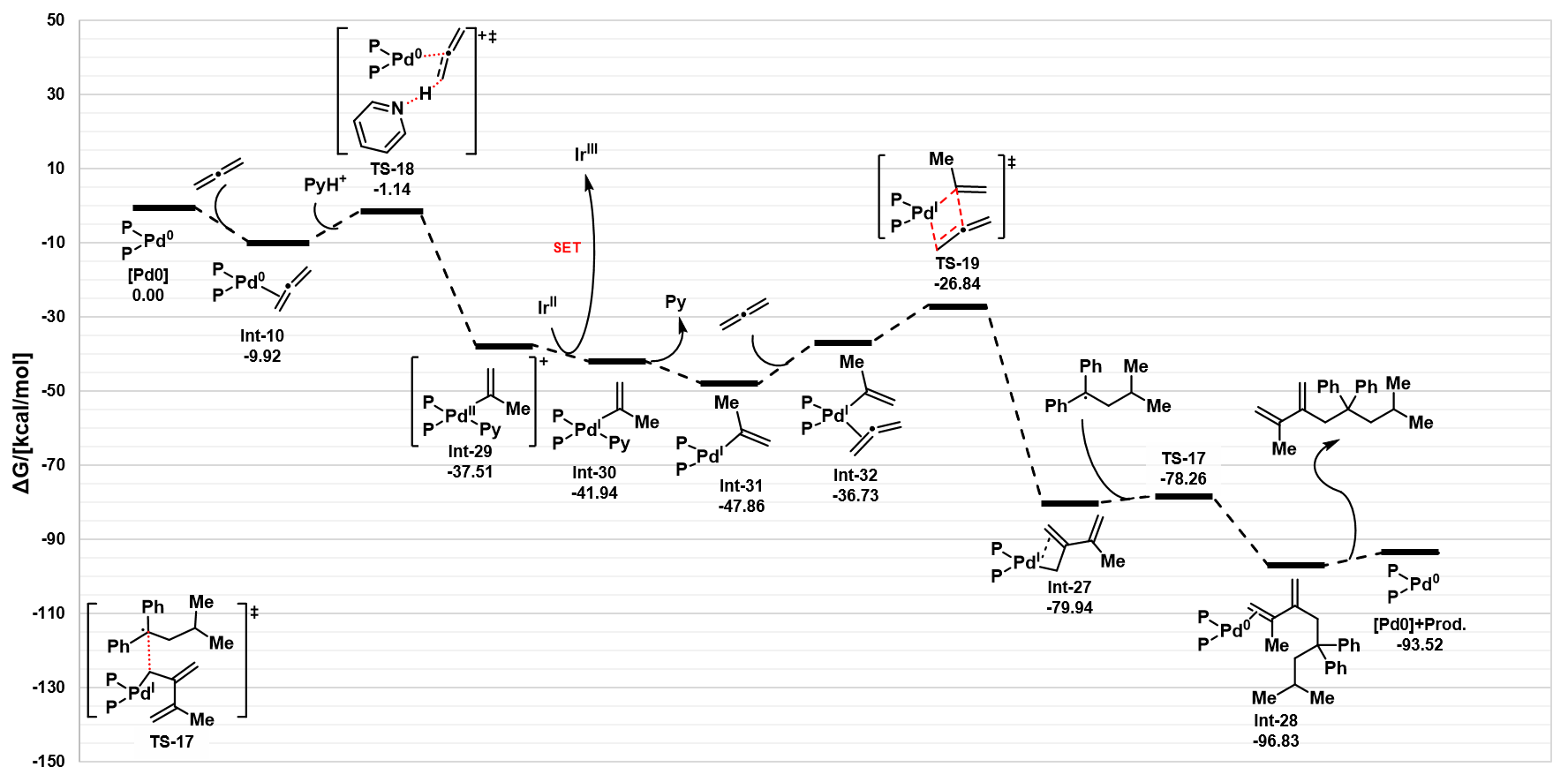


Figure 13 Calculated ΔG profile (B3LYP/def2qzvp/SDD/D3BJ/SMD(toluene)//B3LYP/6-31G(d,p)/SDD/D3BJ) for the formation of the diene product via an outersphere attack (**TS-17**) on [(σ-allyl)Pd^I^(P(2-furyl)_3_)_2_] (**Int-27**) under the dienylation conditions (PyH^+^ as acid additive). **Int-27** was formed before via propadiene complex protonation (**TS-18**) to a methylvinyl complex (**Int-29**), followed by SET reduction using the photocatalyst, ligand exchange and dienyl formation (**TS-19**).

The third pathway (Figure 14) begins with the oxidative addition of PyH⁺ to the palladium catalyst (**TS-20**), yielding **Int-33**. Following the coordination of a propadiene ligand, hydrometallation of the internal position of this ligand (**TS-21**) results in the formation of the π-allyl complex **Int-35**. This cationic intermediate can lose its pyridine ligand and the π-allyl ligand of the obtained **Int-36** can rearrange (**TS-22**) into the methylvinyl complex **Int-24**. This transition state is the energetic span of this pathway and requires 55.5 kcal/mol in energy. From **Int-24** the mechanism is similar to the one shown in Figure 12.


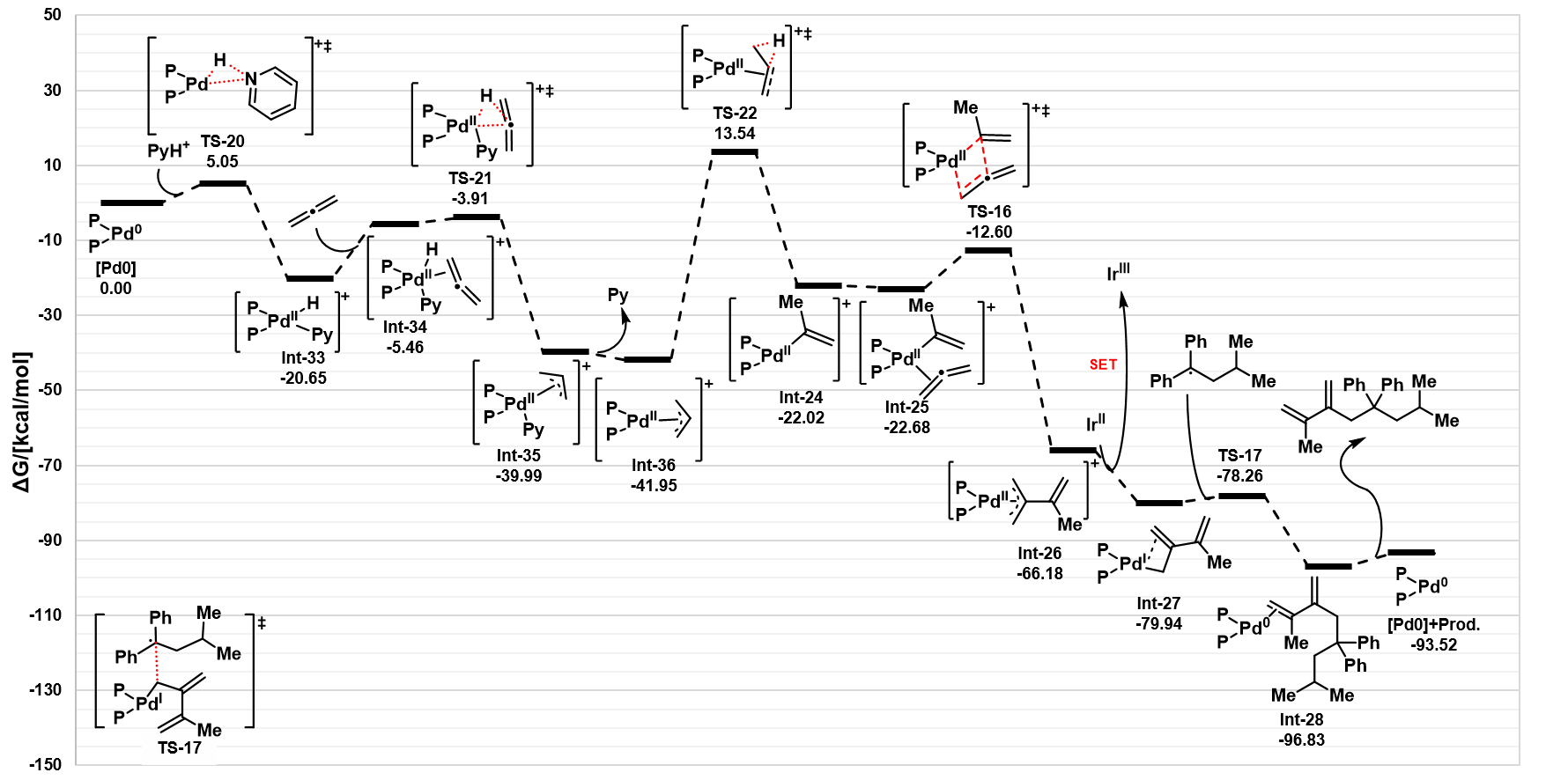


Figure 14 Calculated ΔG profile (B3LYP/def2qzvp/SDD/D3BJ/SMD(toluene)//B3LYP/6-31G(d,p)/SDD/D3BJ) for the formation of the diene product via an outersphere attack (**TS-17**) on [(σ-allyl)Pd^I^(P(2-furyl)_3_)_2_] (**Int-27**) under the dienylation conditions (PyH^+^ as acid additive). **Int-27** was formed before via a sequence of oxidative addition (**TS-20**), propadiene coordination, hydrometalation to the π-allyl complex (**TS-21**), rearrangement the methylvinyl complex (**TS-22**), propadiene coordination, dienyl formation (**TS-16**) and SET-reduction by the photocatalyst.

Figure 15 shows the fourth pathway considered. Similar to the previously discussed pathway, **Int-33** is first formed. However, starting from this intermediate, a ligand exchange of pyridine with propadiene takes place, forming **Int-37**. The terminal position of this propadiene ligand can be hydrometaled in this cationic palladium hydride complex (**TS-23**) yielding again **Int-24**. Afterwards this mechanism is similar to the one presented in Figure 12. This alternative access to **Int-24** makes this pathway the lowest in energy for the formation the diene product using the P(2-furyl)_3_ ligand with an energetic span of ΔG = 16.0 kcal/mol.


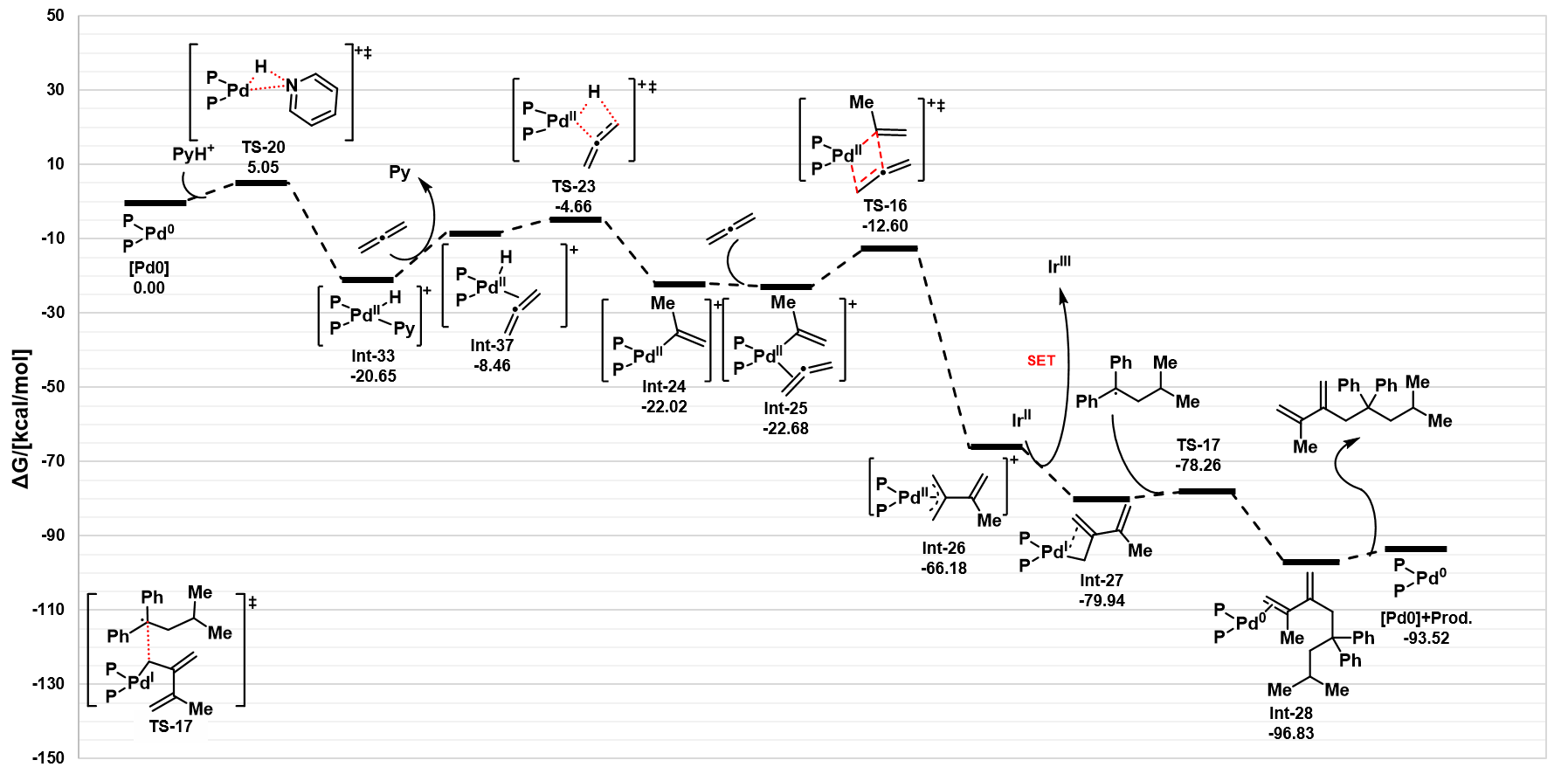


Figure 15 Calculated ΔG profile (B3LYP/def2qzvp/SDD/D3BJ/SMD(toluene)//B3LYP/6-31G(d,p)/SDD/D3BJ) for the formation of the diene product via an outersphere attack (**TS-17**) on [(σ-allyl)Pd^I^(P(2-furyl)_3_)_2_] (**Int-27**) under the dienylation conditions (PyH^+^ as acid additive). **Int-27** was formed before via a sequence of oxidative addition (**TS-20**), hydrometalation to the methylvinyl complex (**TS-23**), propadiene coordination, dienyl formation (**TS-16**) and SET reduction by the photocatalyst.

The fifth pathway begins with the coordination of propadiene to form **Int-10**. This complex can undergo oxidative cyclisation with another propadiene ligand (**TS-24**), resulting in a five-membered palladacycle (**Int-38**). **Int-26** can be formed via protodemetalation with PyH^+^. However, the energy required for **TS-24** is significantly higher than that required for most of the previously discussed mechanisms.


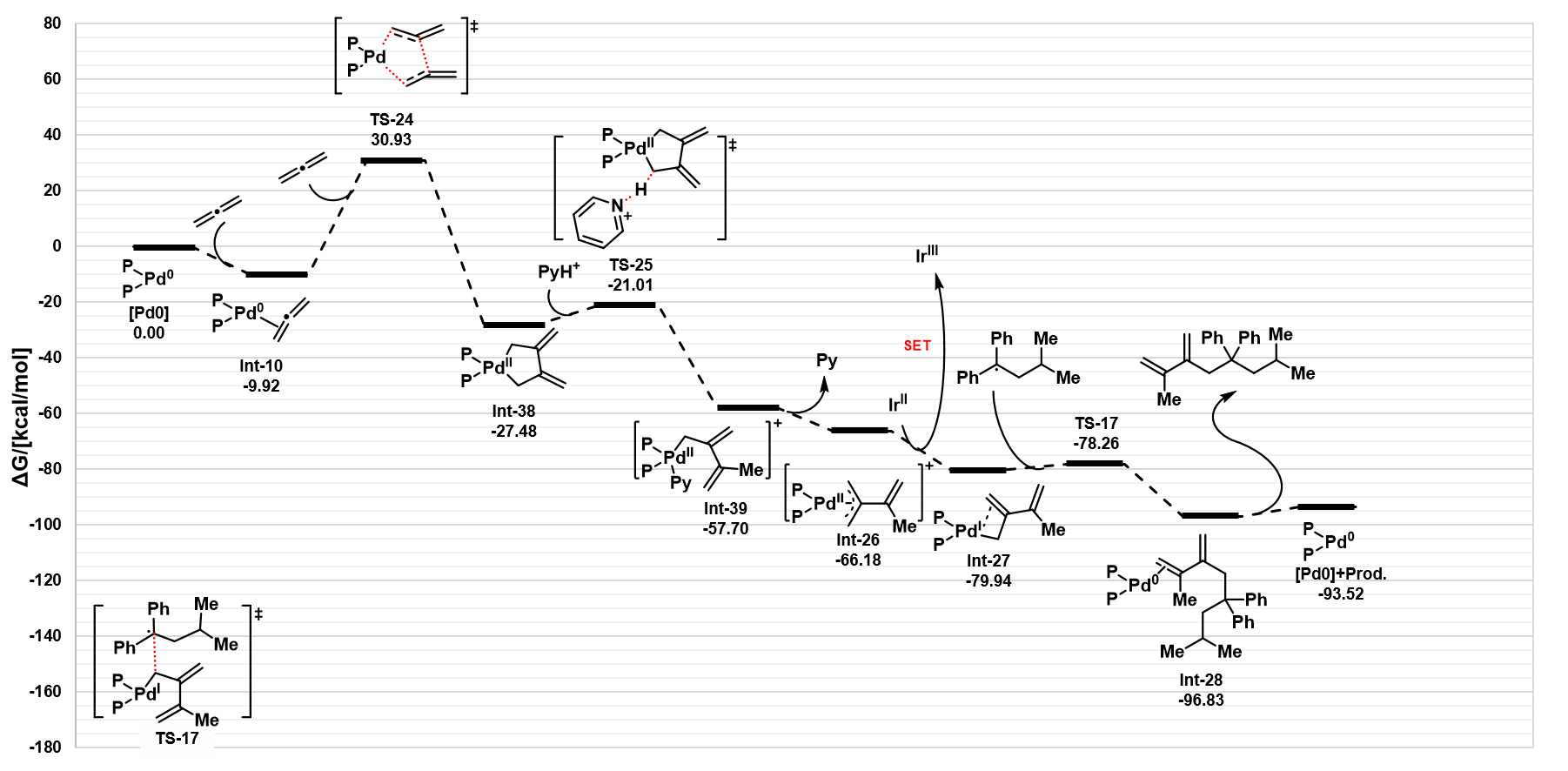


Figure 16 Calculated ΔG profile (B3LYP/def2qzvp/SDD/D3BJ/SMD(toluene)//B3LYP/6-31G(d,p)/SDD/D3BJ) for the formation of the diene product via an outersphere attack (**TS-17**) on [(σ-allyl)Pd^I^(P(2-furyl)_3_)_2_] (**Int-27**) under the dienylation conditions (PyH^+^ as acid additive). **Int-27** was formed before via a sequence of propadiene coordination, oxidative cyclization (**TS-24**), protodemetalation (**TS-25**) and SET reduction by the photocatalyst.

The sixth considered mechanism with this ligand is shown Figure 17. In difference to the before discussed mechanisms, here an outer-sphere attack (**TS-26**) of the radical on the Pd(II) π-allyl complex **Int-26** takes place. This pathway is 1.2 kcal/mol less favored to the one shown in Figure 15. The obtained Pd(I) product complex **Int-40** can then either be reduced by the photocatalyst to the Pd(0) product complex **Int-28** or as an alternative first release the product and then be reduced by the photocatalyst.


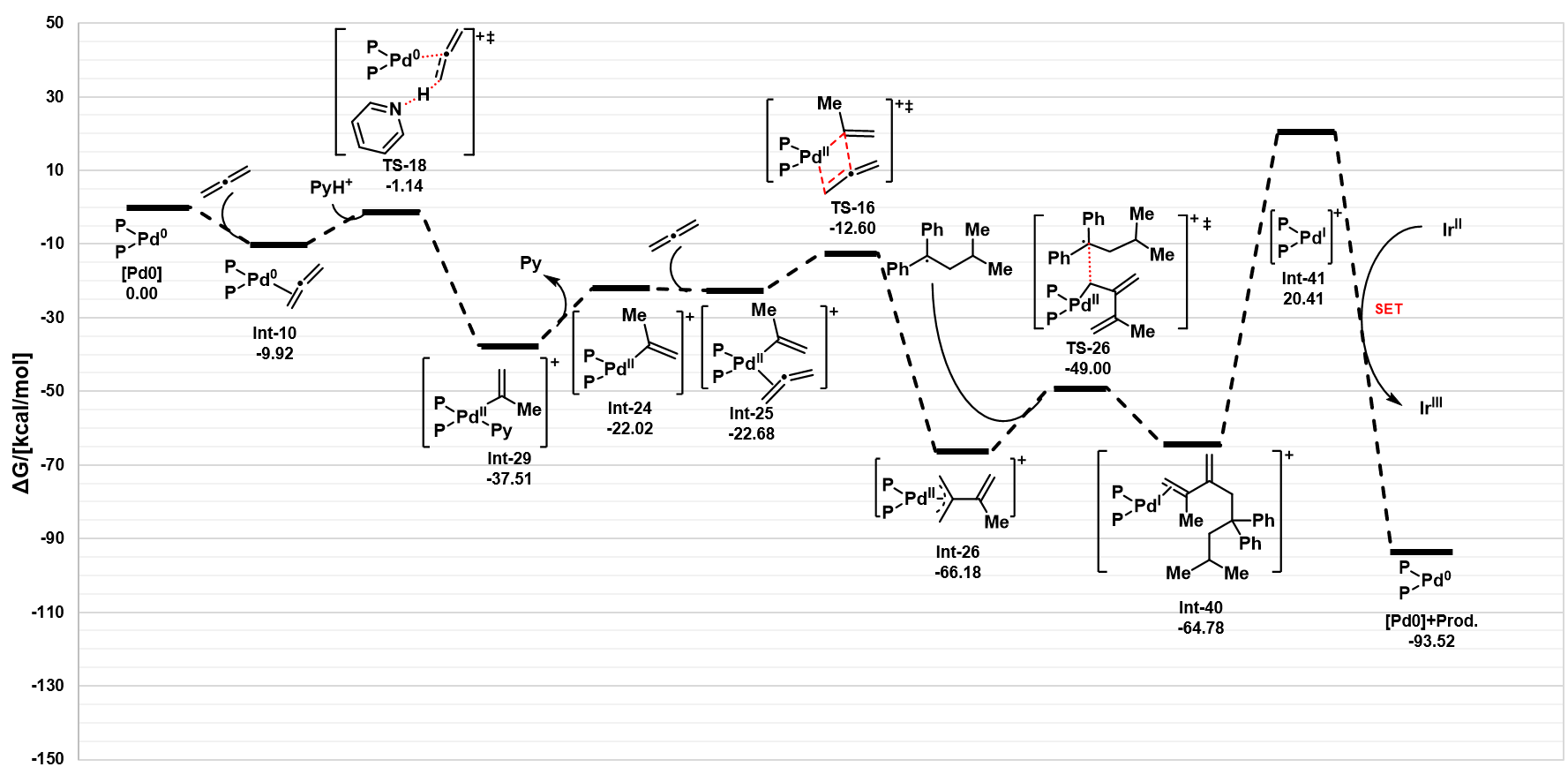


Figure 17 Calculated ΔG profile (B3LYP/def2qzvp/SDD/D3BJ/SMD(toluene)//B3LYP/6-31G(d,p)/SDD/D3BJ) for the formation of the diene product via an outersphere attack (**TS-26**) on [(π-allyl)Pd^II^(P(2-furyl)_3_)_2_]+ (**Int-26**) under the dienylation conditions (PyH^+^ as acid additive). **Int-26** was formed before via a sequence of propadiene complex protonation (**TS-18**) to a methylvinyl complex (**Int-29**), ligand exchange and dienyl formation (**TS-16**).

The last considered pathway is the inner-sphere mechanism shown in Figure 19. Here the radical can coordinate the Pd(I) π-allyl complex **Int-27** to form **Int-42**. The diene product can then be formed via reductive elimination (**TS-27**). The energetic span between **Int-27** and **TS-27** is with ΔG = 31.0 kcal/mol significantly higher than for the most favored pathway discussed before (Figure 15).

Additionally, The Pd(III) mediated inner-sphere reductive elimination was also considered. However, we couldn’t find the corresponding intermediates with both benzyl and dienyl group attached to Pd(III) center. Computationally, we found that during the geometry optimization, the benzyl radical either directly attacked the Pd(II)-allyl moiety through an outer-sphere pathway or stay at the addition of their Van der Waals radius and couldn’t get closer. The results were collected with or without the counter anion coordinated to Pd(II). We assume that the steric bulkiness might hinder the coordination of the benzyl radical to a more constrained Pd(III) center. Since the allocation of Pd(III)-allyl/benzyl complex was not success, the following reductive eliminations were also considered unlikely.


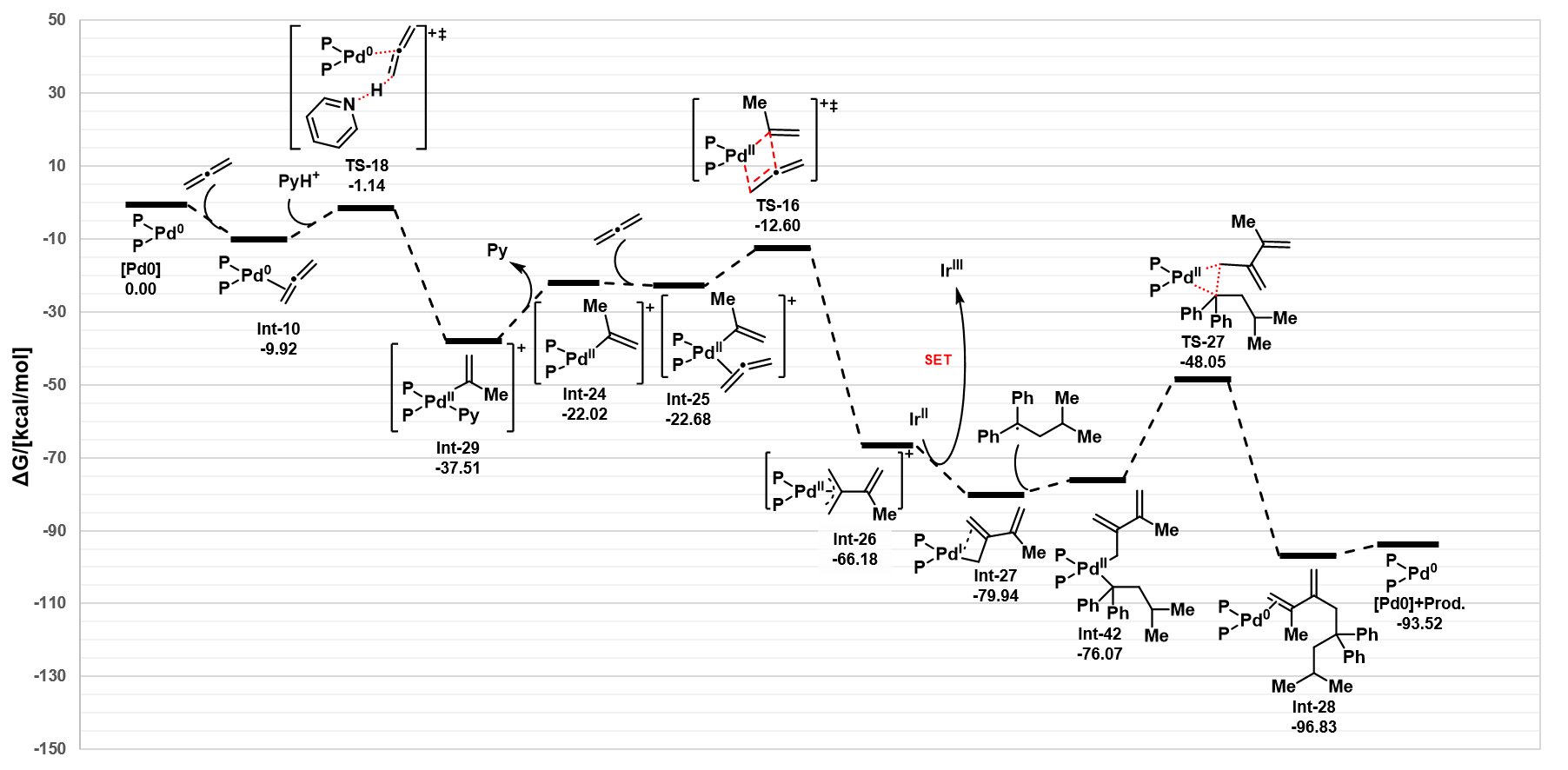


Figure 18 Calculated ΔG profile (B3LYP/def2qzvp/SDD/D3BJ/SMD(toluene)//B3LYP/6-31G(d,p)/SDD/D3BJ) for the formation of the diene product via reductive elimination (**TS-27**) from a [(R)(σ-allyl)Pd^II^(P(2-furyl)_3_)_2_] complex (**Int-42**). This complex was formed before via coordination of the radical to **Int-27**.

Similar to the P(2-furyl)_3_ ligand we considered seven different mechanistic pathways to the diene product using the Xantphos ligand. The first mechanism (Figure 19) starts by coordination of propadiene to the palladium catalyst generating **Int-6**, followed by protonation of the terminal position of the propadiene ligand with PyH^+^ (**TS-28**) forming the cationic methylvinyl complex **Int-43**. After ligand exchange of pyridine by propadiene, this ligand can insert into the Pd-C bond of the methylvinyl ligand (**TS-13**), generating the π-allyl complex **Int-20**. After SET reduction with the iridium photocatalyst, an outer-sphere attack (**TS-14**) of the radical takes place forming product complex **Int-28**. Finally, the palladium catalyst is regenerated by release of the diene product. This pathway is lowest in energy for all considered mechanisms towards the diene product using Xantphos with an energetic span between **Int-18** and **TS-13** of ΔG = 15.8 kcal/mol.


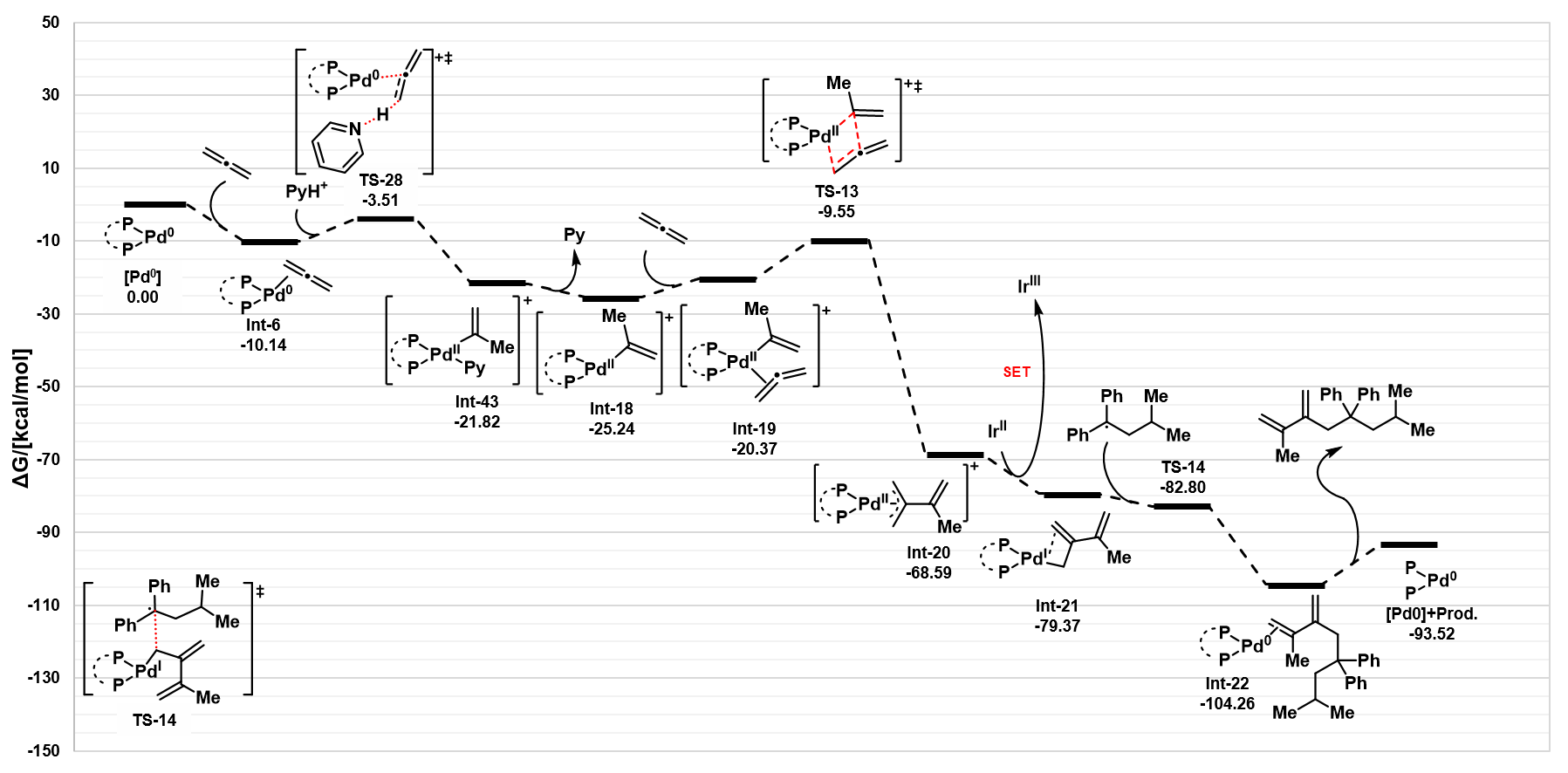


Figure 19 Calculated ΔG profile (B3LYP/def2qzvp/SDD/D3BJ/SMD(toluene)//B3LYP/6-31G(d,p)/SDD/D3BJ) for the formation of the diene product via an outersphere attack (**TS-14**) on [(σ-allyl)Pd^I^(Xantphos)] (**Int-21**) under the dienylation conditions (PyH^+^ as acid additive). **Int-21** was formed before via a sequence of propadiene complex protonation (**TS-18**) to a methylvinyl complex (**Int-43**), ligand exchange, dienyl formation (**TS-13**) and SET reduction by the photocatalyst.

The second mechanism (Figure 20) is similar to the first one (Figure 19) until the formation of **Int-43**, which is SET-reduced by the iridium photocatalyst to form **Int-44**. Following the ligand exchange of pyridine with propadiene to form **Int-46**, propadiene can then be inserted into the Pd-C bond of the Pd(I) complex (**TS-29**), resulting in **Int-21**. From this intermediate, the mechanism is similar to the one previously discussed.


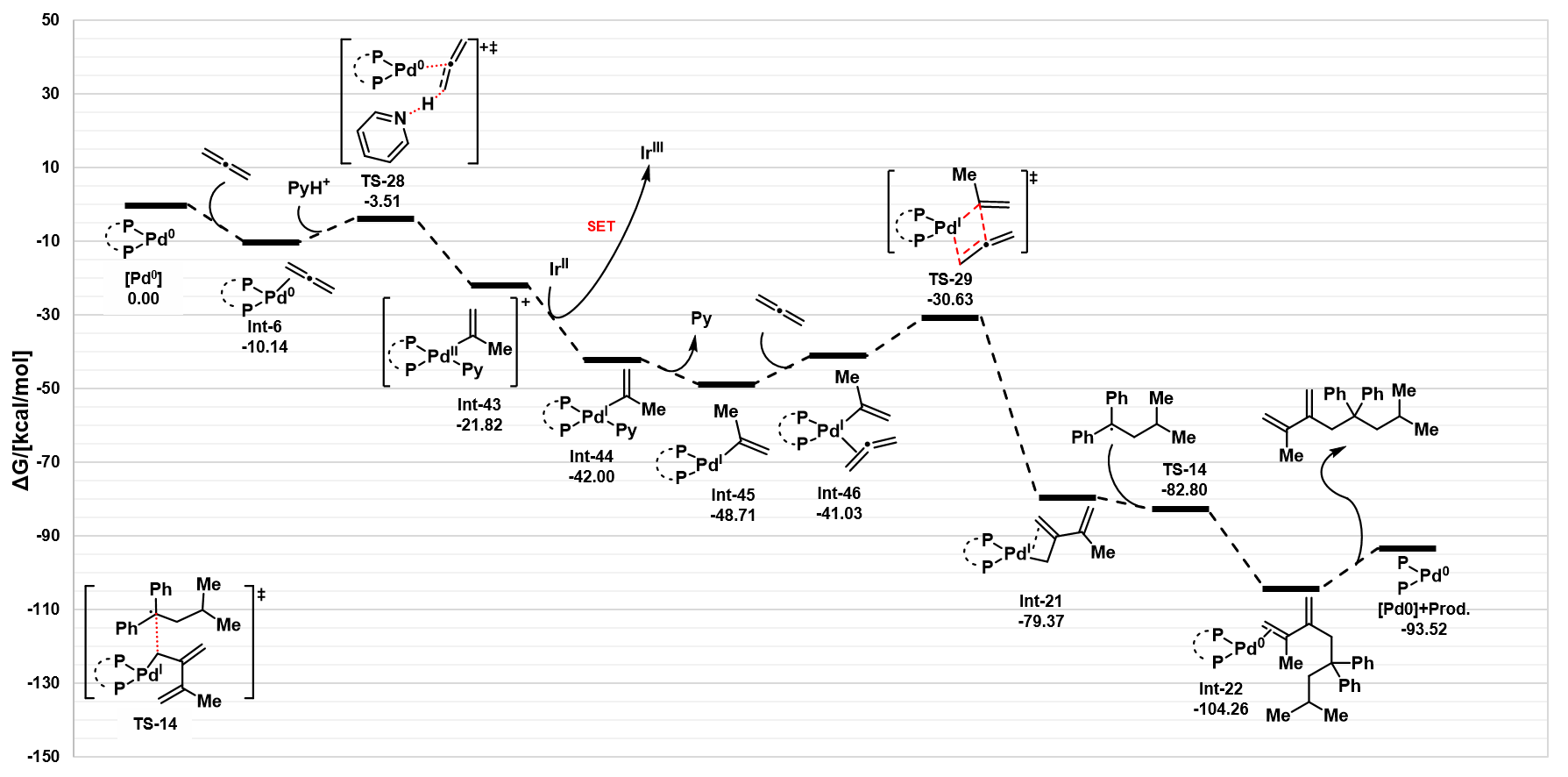


Figure 20 Calculated ΔG profile (B3LYP/def2qzvp/SDD/D3BJ/SMD(toluene)//B3LYP/6-31G(d,p)/SDD/D3BJ) for the formation of the diene product via an outersphere attack (**TS-14**) on [(σ-allyl)Pd^I^(P(Xantphos)] (**Int-21**) under the dienylation conditions (PyH^+^ as acid additive). **Int-21** was formed before via propadiene complex protonation (**TS-28**) to a methylvinyl complex (**Int-43**), followed by SET reduction using the photocatalyst, ligand exchange and dienyl formation (**TS-29**).

The third pathway (Figure 21) begins with the oxidative addition of PyH⁺ to the palladium catalyst (**TS-30**), yielding **Int-33**. Following the coordination of a propadiene ligand, hydrometallation of the internal position of this ligand (**TS-31**) results in the formation of the π-allyl complex **Int-49**. This cationic intermediate can lose its pyridine ligand and the π-allyl ligand of the obtained **Int-50** can rearrange (**TS-32**) into the methylvinyl complex **Int-18**. This transition state is the energetic span of this pathway and requires 57.0 kcal/mol in energy. From **Int-18** the mechanism is similar to the one shown in Figure 19.


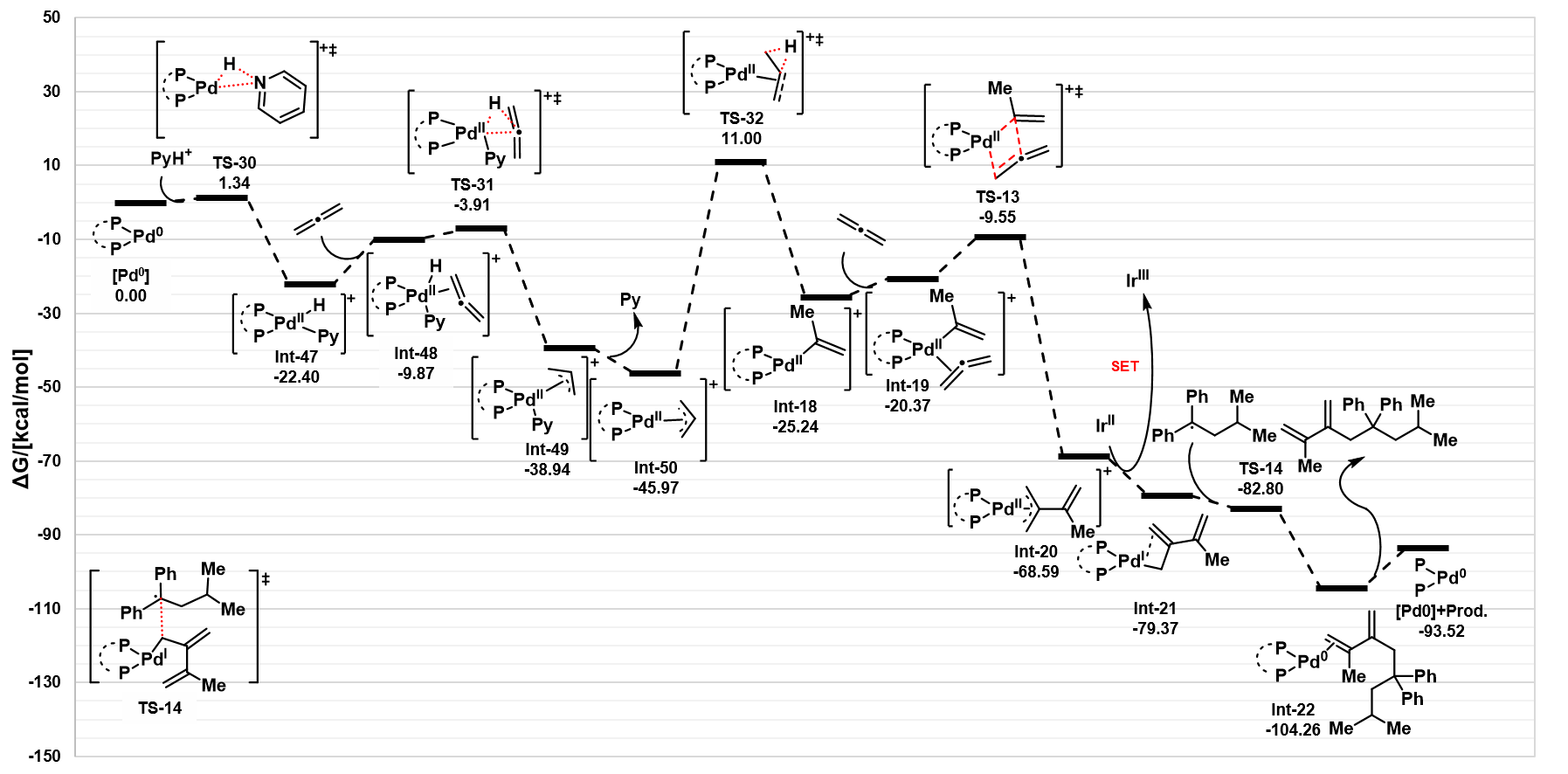


Figure 21 Calculated ΔG profile (B3LYP/def2qzvp/SDD/D3BJ/SMD(toluene)//B3LYP/6-31G(d,p)/SDD/D3BJ) for the formation of the diene product via an outersphere attack (**TS-14**) on [(σ-allyl)Pd^I^(Xantphos)] (**Int-21**) under the dienylation conditions (PyH^+^ as acid additive). **Int-21** was formed before via a sequence of oxidative addition (**TS-30**), propadiene coordination, hydrometalation to the π-allyl complex (**TS-31**), rearrangement the methylvinyl complex (**TS-32**), propadiene coordination, dienyl formation (**TS-13**) and SET-reduction by the photocatalyst.

Figure 22 shows the fourth pathway considered for the Xantphos ligand. Similar to the previously discussed pathway (Figure 21), **Int-47** is first formed. However, starting from this intermediate, a ligand exchange of pyridine with propadiene takes place, forming **Int-51**. The terminal position of this propadiene ligand can be hydrometaled in this cationic palladium hydride complex (**TS-33**) yielding again **Int-18**. Afterwards this mechanism is similar to the one presented in Figure S17. Unlike the corresponding mechanism of the P(2-furyl)_3_ ligand (see Figure 12 and Figure 15), this mechanism is 2.3 kcal/mol higher in energy compared to the mechanism shown in Figure 19.


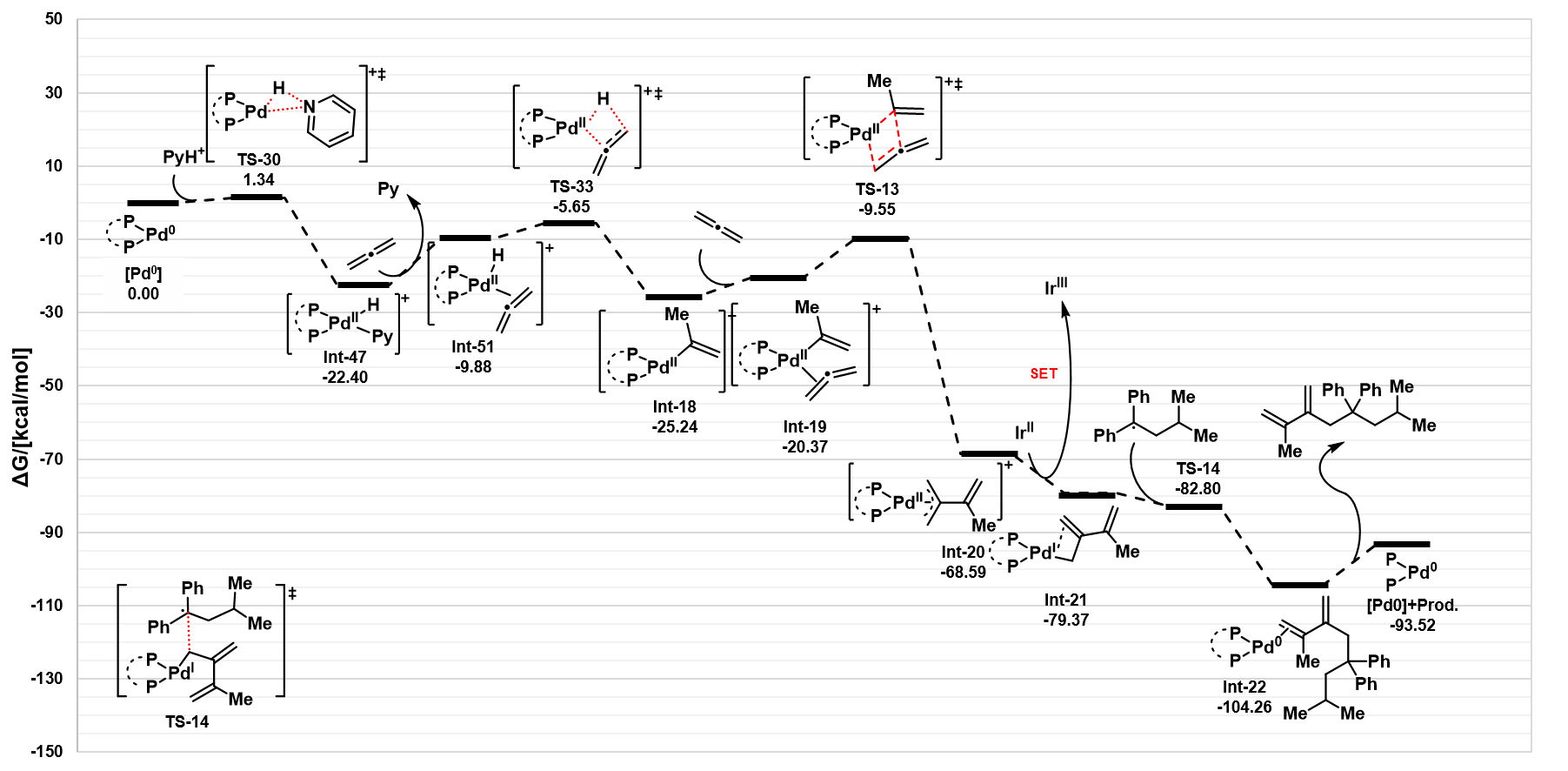


Figure 22 Calculated ΔG profile (B3LYP/def2qzvp/SDD/D3BJ/SMD(toluene)//B3LYP/6‑31G(d,p)/SDD/D3BJ) for the formation of the diene product via an outersphere attack (**TS-14**) on [(σ-allyl)Pd^I^(P(Xantphos)] (**Int-21)** under the dienylation conditions (PyH^+^ as acid additive). **Int-27** was formed before via a sequence of oxidative addition (**TS-30**), hydrometalation to the methylvinyl complex (**TS-33**), propadiene coordination, dienyl formation (**TS-13**) and SET reduction by the photocatalyst.

The fifth pathway (Figure 23) begins with the coordination of propadiene to form **Int-6**. This complex can undergo oxidative cyclisation with another propadiene ligand (**TS-34**), resulting in a five-membered palladacycle (**Int-52**). **Int-20** can then be formed via protodemetalation (**TS-35**) with PyH^+^. However, the energy required for the oxidative cyclization (**TS-24**) is significantly higher than that required for most of the previously discussed mechanisms.


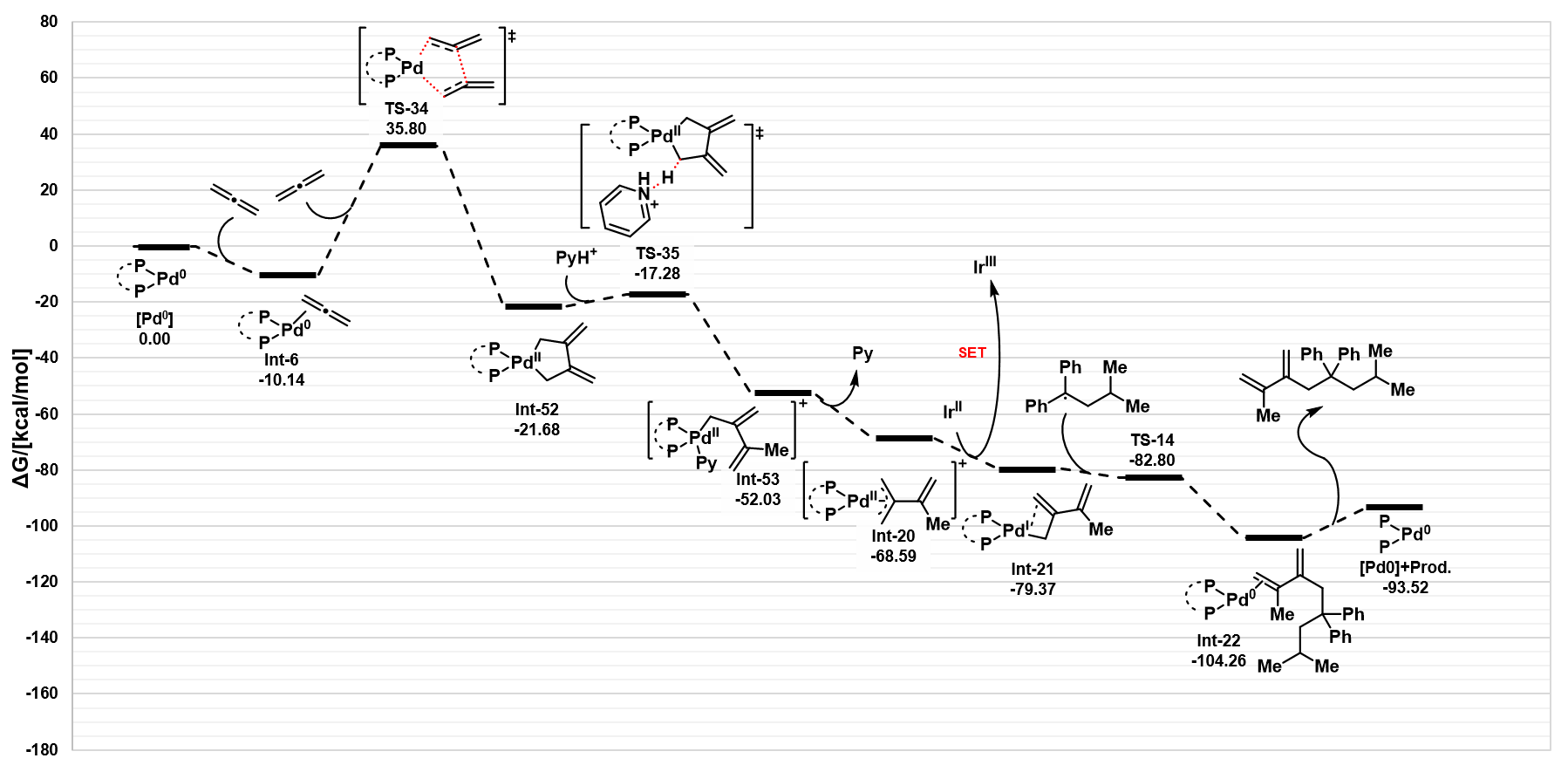


Figure 23 Calculated ΔG profile (B3LYP/def2qzvp/SDD/D3BJ/SMD(toluene)//B3LYP/6-31G(d,p)/SDD/D3BJ) for the formation of the diene product via an outersphere attack (**TS-14**) on [(σ-allyl)Pd^I^(Xantphos)] (**Int-21**) under the dienylation conditions (PyH^+^ as acid additive). **Int-21** was formed before via a sequence of propadiene coordination, oxidative cyclization (**TS-34**), protodemetalation (**TS-35**) and SET reduction by the photocatalyst.

The sixth considered mechanism with this ligand is shown Figure 24. In difference to the before discussed mechanisms, here an outer-sphere attack (**TS-36**) of the radical on the Pd(II) π-allyl complex **Int-20** takes place. This pathway is 1.0 kcal/mol less favored to the one shown in Figure 19. The obtained Pd(I) product complex **Int-55** can then either be reduced by the photocatalyst to the Pd(0) product complex **Int-22** or as an alternative first release the product and then be reduced by the photocatalyst.


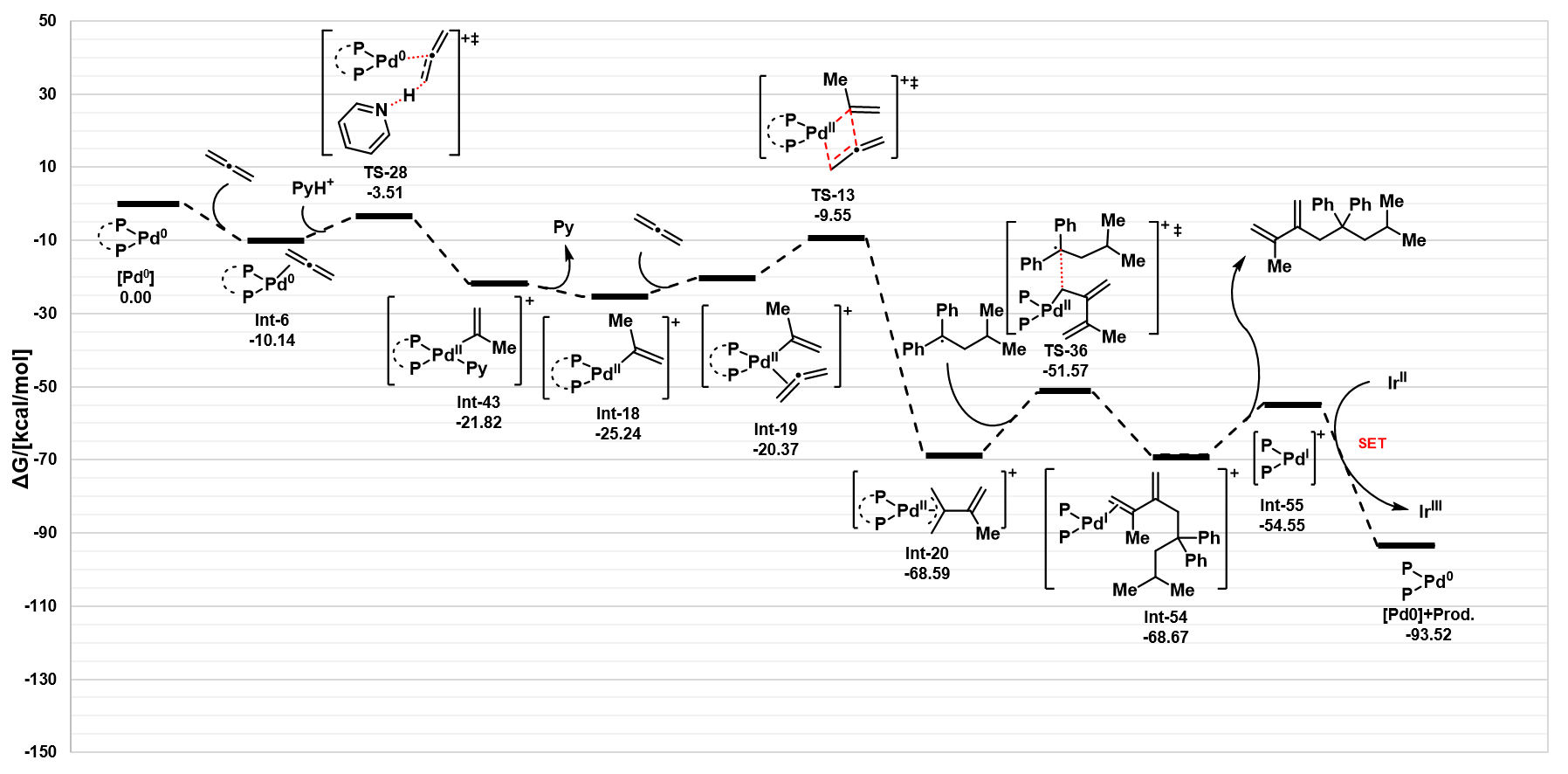


Figure 24 Calculated ΔG profile (B3LYP/def2qzvp/SDD/D3BJ/SMD(toluene)//B3LYP/6-31G(d,p)/SDD/D3BJ) for the formation of the diene product via an outersphere attack (**TS-36**) on [(π-allyl)Pd^II^(Xantphos)]+ (**Int-20**) under the dienylation conditions (PyH^+^ as acid additive). **Int-20** was formed before via a sequence of propadiene complex protonation (**TS-28**) to a methylvinyl complex (**Int-43**), ligand exchange and dienyl formation (**TS-13**).

The last considered pathway is the inner-sphere mechanism shown in Figure 25. Here the radical can coordinate the Pd(I) π-allyl complex **Int-21** to form **Int-56**. The diene product can then be formed via reductive elimination (**TS-37**). The energetic span between **Int-27** and **TS-27** is with ΔG = 34.5 kcal/mol significantly higher than for the most favored pathway discussed before (Figure 19).


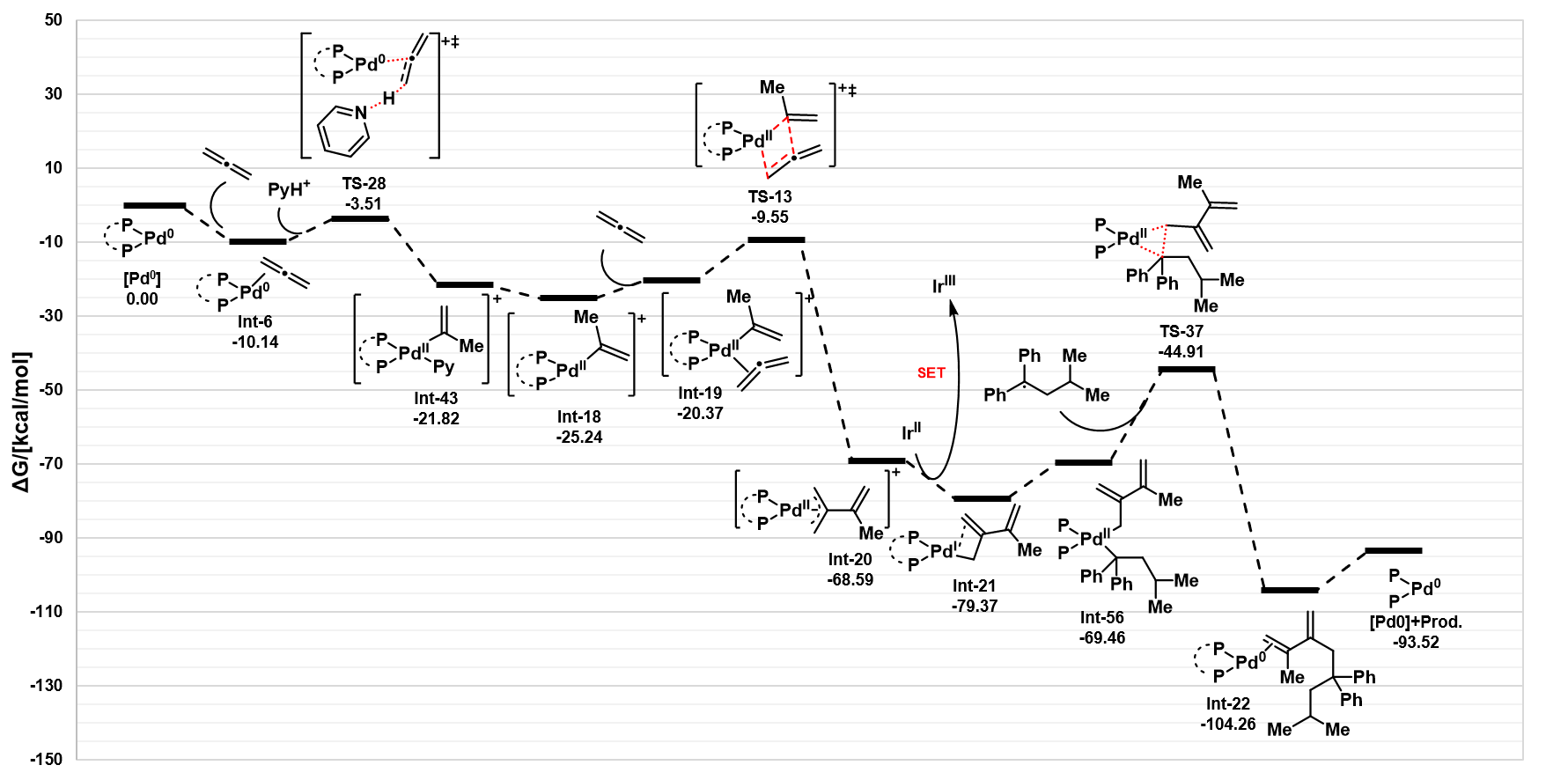


Figure 25 Calculated ΔG profile (B3LYP/def2qzvp/SDD/D3BJ/SMD(toluene)//B3LYP/6-31G(d,p)/SDD/D3BJ) for the formation of the diene product via reductive elimination (**TS-37**) from a [(R)(σ-allyl)Pd^II^(Xantphos)] complex (**Int-56**). This complex was formed before via coordination of the radical to **Int-21**.

For the formation of the diene product we can conclude that an outer-sphere attack on a Pd(I) π-allyl complex is the most favorable pathway (see Figure 15).

#### Mechanistic comparison

Allylation-Std. Cond. 1

Scheme 6 Overview of mechanistic comparison of allylation under std. cond. 1.

Scheme 6 shows the representative mechanistic considerations in terms of allylation under the std. cond. 1. As depicted in Scheme 6, regarding the formation of π-ally palladium complex, the oxidative addition of benzoic acid to Pd (**TS-1**) followed by propadiene hydrometallation (**TS-2**) outcompete the direct protonation of the coordinated propadiene (**TS-4**). Upon the formation of π-ally palladium complex (**Int-3**), three competing pathways were considered. The first alternative (green line) started from the SET reduction of Pd^II^ complex (**Int-3**) to Pd^I^ complex (**Int-4**) by reduced photocatalyst Ir^II^. An outer-sphere reductive elimination of **Int-4** with the benzyl radical can afford the product complex, which, upon dissociation of Pd^0^ species, will deliver the product and close the Pd catalytic cycle. A direct reductive elimination of Pd^II^ complex (**Int-3**) with the radical species was then tested (gray line). It was found that the energetic span of this route was 18.6 kcal/mol, which is 3.9 kcal/mol higher than the Pd^I^ route. Finally, we considered the feasibility of an inner-sphere reductive elimination mechanism (red line). In this scenario, Pd^II^ complex (**Int-3**) was first reduced to Pd^I^ complex (**Int-4**), followed by the coordination of benzyl radical to deliver the Pd^II^ species (**Int-9**). Afterwards an inner-sphere reductive elimination mechanism would proceed to give the product complex. However, this route was significantly less favorable compared to the outer-sphere alternatives due to the significantly higher energetic barrier.

Dienylation

Scheme 7 Overview of mechanistic comparison of dienylation under std. cond.

Scheme 7 shows the representative mechanistic considerations in terms of dienylation under the std. cond. Three possible mechanisms regarding the formation of diene unit were evaluated. The first route started from the oxidative addition of PPTS to Pd complex, leading to the Pd-H species (**Int-33**) (green line). The insertion of one equivalent of propadiene would give a methylvinyl-Pd intermediate (**Int-24**). Another round of propadiene insertion would deliver the π-allyl complex **Int-26**. A mechanistic alternative initiated from the protonation of Pd-coordinated propadiene, giving rise to **Int-29**, upon pyridine ligand dissociation **Int-24** would be formed (orange line). The rest of the process is similar to the first alternative described before. However, when comparing with the first route, the overall energic span for this pathway is higher. The third pathway include the formation of a key five numbered palladacycle through the cyclization of Pd species with two equivalents of propadiene (red line). Afterwards a protodepalladation would proceed to generate the η-allyl Pd complex (**Int-39**), which can easily tautomerized to the π-allyl complex **Int-26**. However, the high activation energy of **TS-24** indicates that this route is quite unlikely. Starting from the **Int-26**, we investigated three viable pathways leading to the final product. We first considered two outer-sphere reductive elimination pathways based on Pd^I^ intermediate **Int-27** (green line) and Pd^II^ intermediate **Int-26** (gray line). The results favored the reductive elimination at the lower valent palladium complex. Alternatively, an inner-sphere reductive elimination from Pd^II^ complex **Int-42** was also tested (light green line). However, it was also proved to be a more energetically demanding pathway.

### Atomic Coordinates

#### Rad· = 1,1-Diphenyl-3-methylbutan-2-yl

| 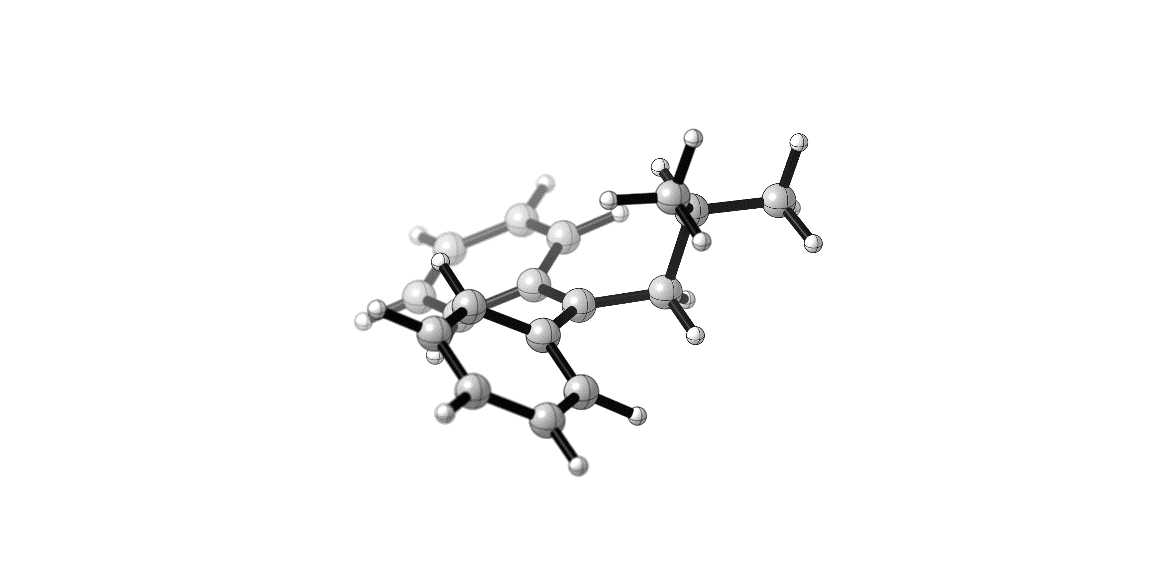 | E/hartree | -659.320959 |
| --- | --- | --- |
|  | E+zvp/hartree | -659.009783 |
|  | G/hartree | -659.053833 |
|  | E(_SP)_/hartree | -659.607780 |

C -0.0158352 1.3654479 -0.8617773

H 0.8633468 1.5120619 -1.4987823

C -0.1573782 -0.0894661 -0.4954483

C 1.0504098 -0.8878801 -0.3054163

C 2.1970398 -0.6993001 -1.1106163

C 1.1441228 -1.8450701 0.7338197

C 3.3584998 -1.4350551 -0.9016843

H 2.1648778 0.0121659 -1.9284733

C 2.3102748 -2.5714801 0.9465937

H 0.2975018 -1.9841671 1.3966887

C 3.4260448 -2.3759961 0.1284967

H 4.2160508 -1.2760471 -1.5491183

H 2.3532548 -3.2863601 1.7632787

H 4.3357788 -2.9445791 0.2944487

C -1.4743092 -0.6393811 -0.2764693

C -1.7202602 -2.0390311 -0.3166873

C -2.6032732 0.1991619 -0.0724613

C -2.9948352 -2.5574411 -0.1356713

H -0.8981292 -2.7124221 -0.5275543

C -3.8749382 -0.3274391 0.1133057

H -2.4740052 1.2743229 -0.0398463

C -4.0843312 -1.7093481 0.0893047

H -3.1448952 -3.6322431 -0.1839973

H -4.7113732 0.3451679 0.2807157

H -5.0797582 -2.1177821 0.2328167

C 0.1337468 2.3257069 0.3515747

H -0.7234192 2.1599279 1.0175967

C 1.4105878 2.0516229 1.1532997

H 1.4996868 2.7556429 1.9875077

H 1.4255878 1.0383459 1.5606547

H 2.2993588 2.1659059 0.5215057

C 0.0917718 3.7793159 -0.1332603

H -0.8355842 3.9960759 -0.6746883

H 0.1638128 4.4781439 0.7066107

H 0.9292748 3.9862079 -0.8105273

H -0.8787062 1.6852639 -1.4557393

#### Propadiene

| 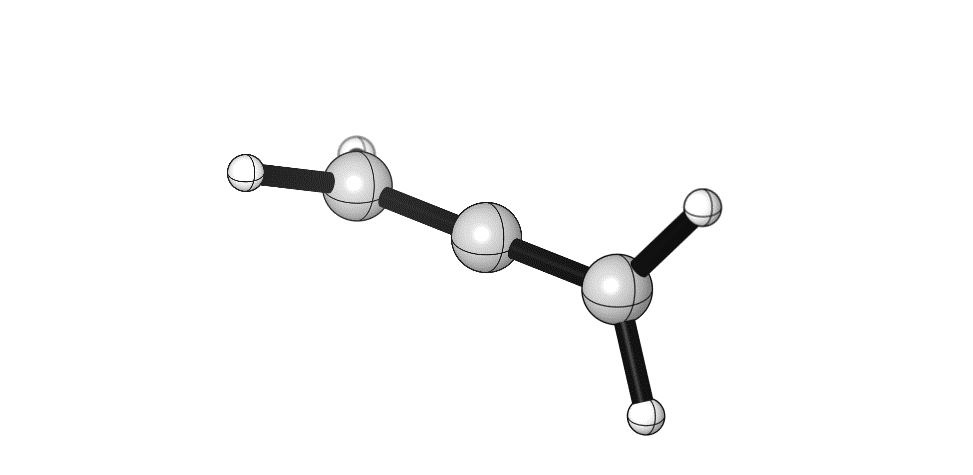 | E/hartree | -116.667180 |
| --- | --- | --- |
|  | E+zvp/hartree | -116.611872 |
|  | G/hartree | -116.635967 |
|  | E(_SP)_/hartree | -116.725729 |

C 0.0001400 -0.0003991 -0.0000984

C 0.8146010 0.5089809 -0.8855054

H 1.6835370 1.0909209 -0.5875004

H 0.6506200 0.3712459 -1.9516474

C -0.8141420 -0.5096431 0.8855586

H -0.6579060 -1.5060411 1.2921116

H -1.6768500 0.0449359 1.2470816

#### (6-methylhept-1-ene-4,4-diyl)dibenzene

| 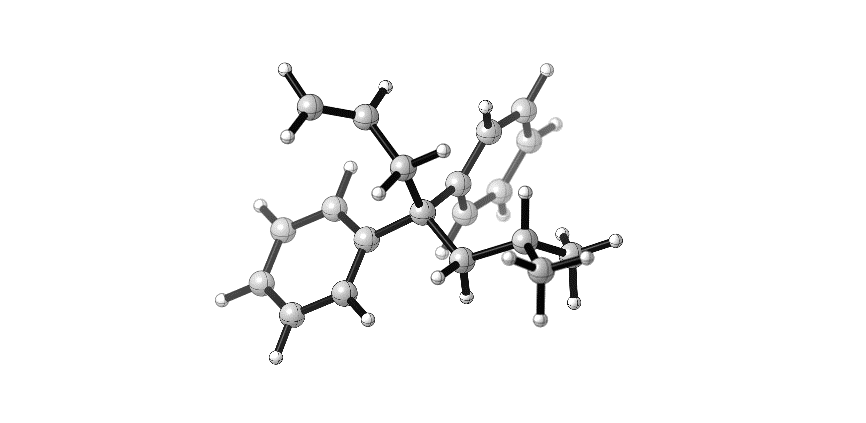 | E/hartree | -776.675892 |
| --- | --- | --- |
|  | E+zvp/hartree | -776.290205 |
|  | G/hartree | -776.337391 |
|  | E(_SP)_/hartree | -777.011249 |

C -0.2224149 -0.2574733 0.1826968

C 0.6656911 0.9797667 0.0077138

C 0.6551911 1.6638097 -1.2182812

C 1.5804541 1.3991187 0.9804568

C 1.5246861 2.7233277 -1.4632892

H -0.0452329 1.3569227 -1.9878642

C 2.4551731 2.4616417 0.7401648

H 1.6306921 0.8924997 1.9370658

C 2.4327891 3.1286877 -0.4824462

H 1.4949891 3.2320417 -2.4224432

H 3.1549331 2.7636397 1.5139388

H 3.1118961 3.9547487 -0.6702882

C -1.6207629 -0.0061993 -0.4058122

C -2.2649979 1.2190917 -0.1722672

C -2.3256089 -0.9865443 -1.1145842

C -3.5562519 1.4587057 -0.6343692

H -1.7416819 1.9978167 0.3702248

C -3.6218339 -0.7513993 -1.5799572

H -1.8718259 -1.9491923 -1.3167102

C -4.2438489 0.4720857 -1.3432972

H -4.0259149 2.4185347 -0.4397012

H -4.1408039 -1.5300603 -2.1315832

H -5.2502439 0.6568877 -1.7069692

C -0.4245269 -0.6671683 1.6800508

H -1.0407279 -1.5722003 1.6800588

H 0.5477991 -0.9576563 2.0975998

C -1.0622729 0.3565997 2.5744928

C -2.2289969 0.1870997 3.1954938

H -0.5220749 1.2913127 2.7124378

H -2.6491989 0.9521627 3.8414528

H -2.8110699 -0.7229643 3.0726878

C 0.4769171 -1.4553843 -0.5334592

H -0.1045369 -2.3548473 -0.3033362

H 0.3979031 -1.3065283 -1.6164042

C 1.9582001 -1.7259703 -0.1699062

H 2.1701741 -1.2906673 0.8141718

C 2.2105111 -3.2367893 -0.0684022

H 3.2560681 -3.4490283 0.1789148

H 1.5801181 -3.6956363 0.7010878

H 1.9877581 -3.7330713 -1.0207752

C 2.9321951 -1.0934313 -1.1741632

H 3.9698391 -1.2914653 -0.8844482

H 2.7791741 -1.5201483 -2.1730542

H 2.8016741 -0.0126763 -1.2468992

#### (2,7-dimethyl-6-methyleneoct-7-ene-4,4-diyl)dibenzene

| **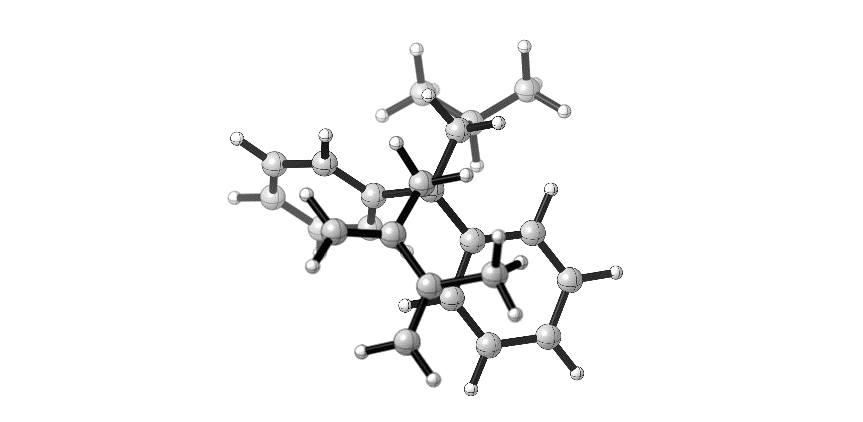** | E/hartree | -893.409917 |
| --- | --- | --- |
|  | E+zvp/hartree | -892.961965 |
|  | G/hartree | -893.012689 |
|  | E(_SP)_/hartree | -893.791370 |

C 0.2249122 0.0224953 -0.4628561

C 1.1639672 -0.9046847 0.3241109

C 1.5058452 -0.6498747 1.6596179

C 1.8358262 -1.9511817 -0.3244001

C 2.4614202 -1.4190727 2.3245729

H 1.0294302 0.1668963 2.1875059

C 2.7893772 -2.7251087 0.3343639

H 1.6360432 -2.1571827 -1.3692261

C 3.1062472 -2.4656607 1.6678759

H 2.7041372 -1.1913787 3.3584669

H 3.2889102 -3.5285157 -0.1992061

H 3.8488872 -3.0664307 2.1840249

C -0.7410358 0.7889763 0.4508069

C -1.4074188 0.1225303 1.4901349

C -1.0706078 2.1334533 0.2289729

C -2.3588228 0.7691363 2.2752179

H -1.1843498 -0.9200007 1.6788629

C -2.0221528 2.7876933 1.0136699

H -0.5901058 2.6883753 -0.5661921

C -2.6725588 2.1080143 2.0419269

H -2.8634108 0.2184683 3.0633649

H -2.2517548 3.8307523 0.8163669

H -3.4139108 2.6140213 2.6530209

C -0.6366648 -0.7585437 -1.5231651

H -1.1924528 0.0033643 -2.0780371

H 0.0481782 -1.2073277 -2.2468601

C -1.5995388 -1.8333017 -1.0485191

C -1.2504818 -3.1273527 -1.1240461

H -1.9502198 -3.9174877 -0.8739501

H -0.2621358 -3.4339007 -1.4470231

C 1.1268192 0.9811303 -1.3024441

H 1.6941342 0.3603193 -2.0052341

H 0.4711792 1.5982063 -1.9271511

C 2.1141322 1.8865473 -0.5255921

H 1.7276842 2.0274943 0.4912779

C 3.5170772 1.2687723 -0.4234591

H 4.1846712 1.9210253 0.1500369

H 3.5050592 0.2906873 0.0584269

H 3.9517492 1.1472953 -1.4233571

C 2.2088922 3.2689833 -1.1870251

H 2.9148052 3.9143143 -0.6537321

H 2.5583452 3.1799133 -2.2227931

H 1.2397322 3.7787983 -1.2066591

C -2.9736518 -1.4577847 -0.6047441

C -3.6198328 -2.1588157 0.3381029

H -4.6377628 -1.9119747 0.6235409

H -3.1445258 -2.9771817 0.8686769

C -3.6270438 -0.2637727 -1.2582861

H -3.1248028 0.6653353 -0.9697621

H -3.5880478 -0.3321057 -2.3519541

H -4.6741708 -0.1843597 -0.9572711

#### BzOH

| 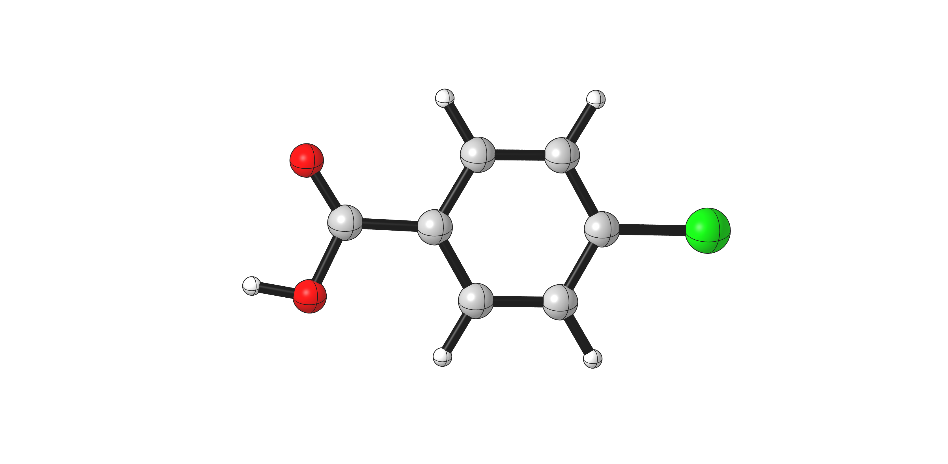 | E/hartree | -880.448066 |
| --- | --- | --- |
|  | E+zvp/hartree | -880.341687 |
|  | G/hartree | -880.375858 |
|  | E(_SP)_/hartree | -880.704249 |

C 2.1231745 0.0441069 0.0000266

C 1.4264015 -1.1649271 -0.0000564

C 0.0347495 -1.1432251 -0.0001834

C -0.6510755 0.0778379 -0.0002514

C 0.0666905 1.2802939 -0.0001664

C 1.4567955 1.2709579 -0.0000294

H 1.9688995 -2.1030231 -0.0000174

H -0.5228565 -2.0721501 -0.0002624

H -0.4842195 2.2141679 -0.0002314

H 2.0220425 2.1955099 0.0000266

Cl 3.8753515 0.0216579 0.0002016

C -2.1330955 0.1539909 -0.0004664

O -2.7743765 1.1859149 0.0004016

O -2.7262445 -1.0659431 0.0003386

H -3.6822375 -0.8951701 0.0006696

#### BzO^-^

| 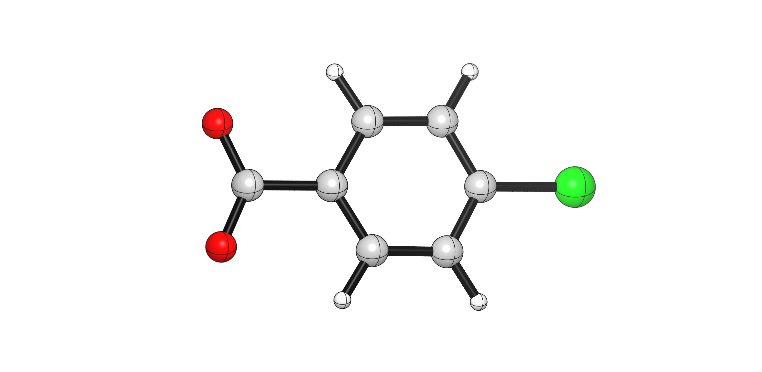 | E/hartree | -879.882064 |
| --- | --- | --- |
|  | E+zvp/hartree | -879.789466 |
|  | G/hartree | -879.822908 |
|  | E(_SP)_/hartree | -880.197118 |

C 0.0000000 0.0000000 1.8670541

C 0.0000000 1.2176550 1.1890261

C 0.0000000 1.2020820 -0.2064619

C 0.0000000 0.0000000 -0.9205079

C 0.0000000 -1.2020820 -0.2064619

C 0.0000000 -1.2176550 1.1890261

H 0.0000000 2.1491490 1.7469371

H 0.0000000 2.1170940 -0.7913279

H 0.0000000 -2.1170940 -0.7913279

H 0.0000000 -2.1491490 1.7469371

Cl 0.0000000 0.0000000 3.6482521

C 0.0000000 0.0000000 -2.4731529

O 0.0000000 -1.1404190 -2.9989959

O 0.0000000 1.1404190 -2.9989959

#### PyH^+^

| 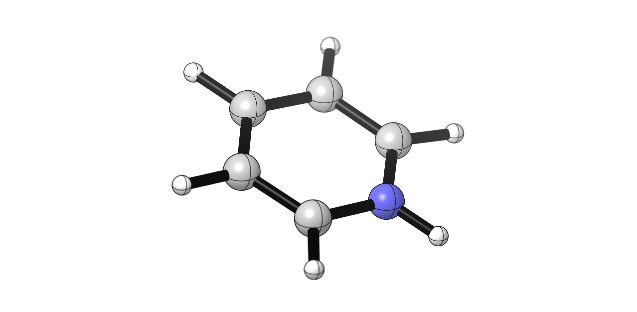 | E/hartree | -248.681091 |
| --- | --- | --- |
|  | E+zvp/hartree | -248.577907 |
|  | G/hartree | -248.605407 |
|  | E(_SP)_/hartree | -248.846340 |

C 1.1901137 -0.6900567 0.0000000

C 1.2115827 0.6934573 0.0000000

C -0.0000153 1.3921103 0.0000000

C -1.2115863 0.6934593 0.0000000

C -1.1900893 -0.6900777 0.0000000

H 0.0000157 -2.3488407 0.0000000

H 2.0792098 -1.3085067 0.0000000

H 2.1632997 1.2109823 0.0000000

H 0.0000067 2.4771143 0.0000000

H -2.1633343 1.2109263 0.0000000

H -2.0792043 -1.3085027 0.0000000

N 0.0000007 -1.3320657 0.0000000

#### Pyridine

| 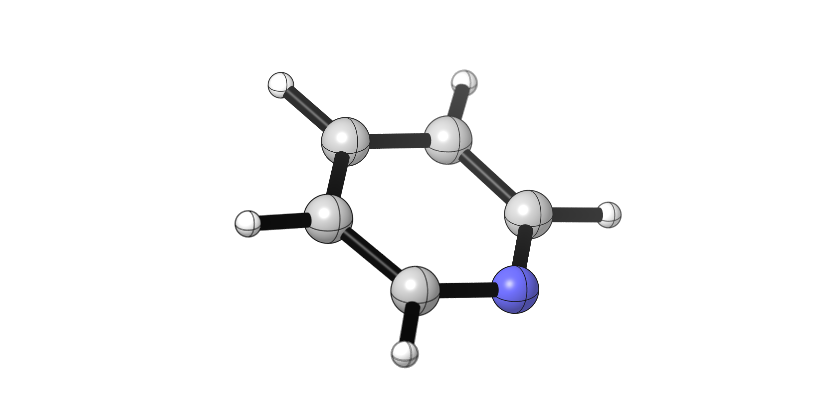 | E/hartree | -248.304204 |
| --- | --- | --- |
|  | E+zvp/hartree | -248.215228 |
|  | G/hartree | -248.242628 |
|  | E(_SP)_/hartree | -248.420556 |

C 1.1425535 -0.9113964 -0.0000973

C 1.1982915 0.4831196 0.0000107

C -0.0001705 1.1954866 -0.0000273

C -1.1984115 0.4828396 -0.0000373

C -1.1422885 -0.9117154 0.0000437

H 2.0603505 -1.4968694 0.0000997

H 2.1565345 0.9925996 0.0001107

H -0.0002635 2.2814876 -0.0000043

H -2.1568245 0.9919996 -0.0000643

H -2.0599715 -1.4973754 -0.0000183

N 0.0002005 -1.6101764 -0.0000163

#### PF_6_^-^

| 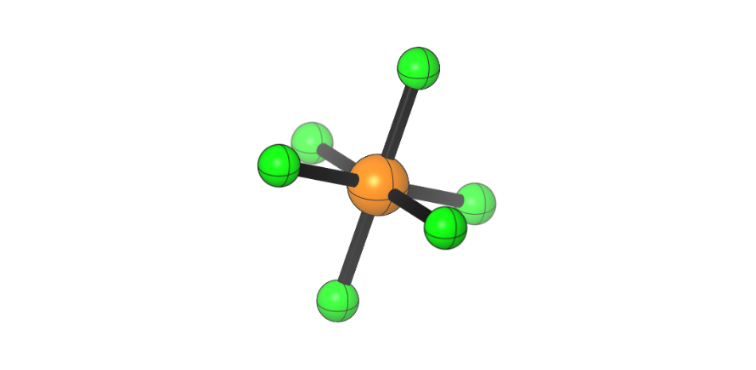 | E/hartree | -940.643079 |
| --- | --- | --- |
|  | E+zvp/hartree | -940.623854 |
|  | G/hartree | -940.654081 |
|  | E(_SP)_/hartree | -941.127824 |

P 0.0001017 0.0000580 0.0000087

F -1.6259203 0.1384240 0.1412937

F -0.1719973 -1.5666160 -0.4460073

F -0.0975943 0.4575190 -1.5697993

F 1.6258187 -0.1383020 -0.1412563

F 0.1721437 1.5665430 0.4459697

F 0.0974477 -0.4576260 1.5697907

#### [Ir(II)]

| 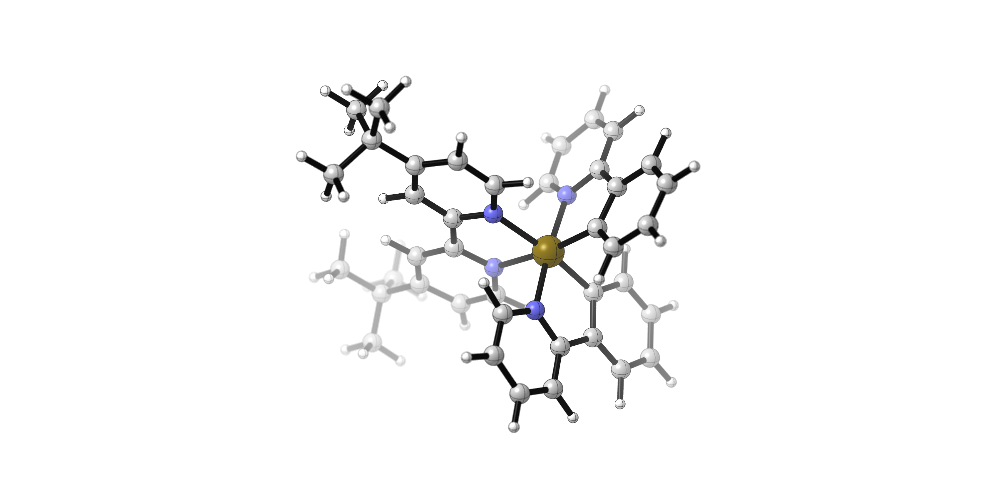 | E/hartree | -1872.135425 |
| --- | --- | --- |
|  | E+zvp/hartree | -1871.430180 |
|  | G/hartree | -1871.504875 |
|  | E(_SP)_/hartree | -1872.896451 |

Ir 1.7209035 0.0078793 0.0035105

C 3.1669425 1.4142073 0.1737865

C 3.4602355 2.1074503 -1.0331305

C 3.8797035 1.7952323 1.3215665

C 4.4141575 3.1369843 -1.0713645

C 4.8274825 2.8179713 1.2814755

C 5.0971635 3.4941703 0.0851095

H 4.6262615 3.6619733 -1.9988455

H 5.3644475 3.0923543 2.1861995

H 5.8352125 4.2898163 0.0588895

C 2.6824885 -1.6648087 2.2058295

C 2.7873825 -2.1807377 3.5048835

C 2.0037785 -1.6645497 4.5271225

H 3.4857205 -2.9850227 3.7017745

C 1.0388615 -0.1493687 2.9467465

C 1.1090695 -0.6282917 4.2460145

H 2.0862695 -2.0645297 5.5328765

H 0.3682155 0.6500013 2.6579075

H 0.4753425 -0.1986447 5.0130425

C 3.1532785 -1.4113057 -0.1677655

C 3.8623775 -1.7976107 -1.3160425

C 3.4404255 -2.1081687 1.0384795

C 4.8008365 -2.8289027 -1.2769605

C 4.3850395 -3.1462907 1.0756215

C 5.0646155 -3.5085887 -0.0812435

H 5.3350825 -3.1073427 -2.1820495

H 4.5924955 -3.6739737 2.0026155

H 5.7954375 -4.3109017 -0.0558685

C 2.6984945 1.6696853 -2.2002955

C 1.0413595 0.1679503 -2.9397985

C 2.8090165 2.1824053 -3.5001405

C 1.1170185 0.6440033 -4.2398605

C 2.0213805 1.6715423 -4.5220245

H 3.5150695 2.9796713 -3.6980775

H 0.4801795 0.2186753 -5.0067165

H 2.1084075 2.0688813 -5.5284505

C -1.2042125 0.7180683 0.1559465

C 0.1013465 2.6386653 0.5677615

C -2.3706575 1.5128033 0.3266815

C -1.0122835 3.4420053 0.7344265

H 1.1043035 3.0449733 0.6552305

C -2.3063715 2.8612593 0.6139235

H -3.3371915 1.0344633 0.2236445

H -0.8731535 4.4919563 0.9506295

C 0.0753925 -2.6079727 -0.5634875

C -1.2100205 -0.6739627 -0.1495115

C -1.0455535 -3.3944227 -0.7286825

H 1.0741375 -3.0241817 -0.6516575

C -2.3905535 -1.4542817 -0.3185155

C -2.3396495 -2.7993177 -0.6058235

H -0.9247405 -4.4483977 -0.9460655

H -3.3464625 -0.9603547 -0.2127055

N 0.0401915 1.3249693 0.3000815

N 0.0278985 -1.2909237 -0.2943415

H 3.6788515 -1.2820557 -2.2546775

H 0.3635645 -0.6248667 -2.6494375

N 1.8033235 -0.6462947 1.9575785

N 1.8096285 0.6599223 -1.9510945

H 3.6917055 1.2820313 2.2606495

C -3.5880935 -3.6631267 -0.7918815

C -4.8880185 -2.8603027 -0.6265865

C -3.5722425 -4.2768637 -2.2093375

C -3.5793405 -4.7968017 0.2573135

H -4.9658595 -2.4210657 0.3730315

H -4.9596225 -2.0533077 -1.3627705

H -5.7505245 -3.5188577 -0.7691155

H -2.6803135 -4.8870967 -2.3751625

H -4.4500815 -4.9151567 -2.3589795

H -3.5865325 -3.4905587 -2.9708825

H -4.4580005 -5.4392927 0.1323555

H -2.6882785 -5.4238167 0.1667635

H -3.5967645 -4.3846427 1.2711885

C -3.5966605 3.6737153 0.7886665

C -4.4254345 3.6041703 -0.5122625

C -4.4196105 3.0824213 1.9536315

C -3.3115495 5.1522023 1.1007025

H -3.8587965 4.0128633 -1.3548355

H -4.6982775 2.5749193 -0.7612265

H -5.3505435 4.1823913 -0.4082265

H -3.8498325 3.1176563 2.8876765

H -5.3456055 3.6509993 2.0958265

H -4.6904025 2.0395893 1.7665455

H -4.2556205 5.6935463 1.2179985

H -2.7442645 5.2667943 2.0296255

H -2.7493795 5.6337193 0.2945745

#### [Ir(III)]

| 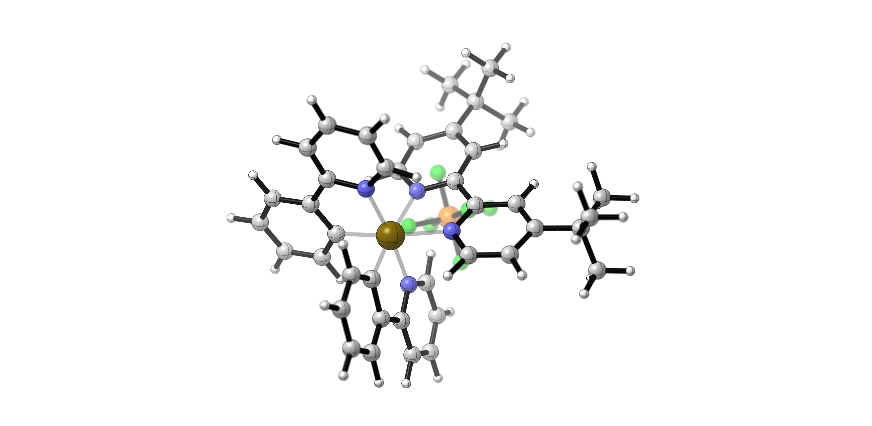 | E/hartree | -2812.777734 |
| --- | --- | --- |
|  | E+zvp/hartree | -2812.047858 |
|  | G/hartree | -2812.130223 |
|  | E(_SP)_/hartree | -2813.948481 |

Ir 1.6958162 -0.7062122 -0.1487781

C 2.2072452 -2.6552902 -0.1485131

C 2.6345142 -3.1564982 1.1110399

C 2.1404902 -3.5577692 -1.2191761

C 2.9784932 -4.5084132 1.2770409

C 2.4802222 -4.8997252 -1.0487021

C 2.9023232 -5.3792182 0.1977659

H 3.2997472 -4.8838832 2.2445519

H 2.4130942 -5.5814252 -1.8921031

H 3.1636402 -6.4252032 0.3220409

C 2.4152942 -0.2757212 -2.9573771

C 2.2721072 -0.1913122 -4.3445511

C 1.0376812 -0.4455692 -4.9327871

H 3.1305392 0.0640898 -4.9540671

C 0.1362612 -0.8452952 -2.7522621

C -0.0498038 -0.7790052 -4.1277651

H 0.9276562 -0.3883522 -6.0113621

H -0.6774718 -1.1127812 -2.0945351

H -1.0329398 -1.0004842 -4.5197641

C 3.5511712 -0.2386922 -0.8027671

C 4.7092262 -0.0450262 -0.0360921

C 3.6522882 -0.0496392 -2.2062441

C 5.9155502 0.3318348 -0.6301621

C 4.8648462 0.3303428 -2.7999441

C 5.9961962 0.5227658 -2.0131861

H 6.7995452 0.4737908 -0.0137601

H 4.9320312 0.4760358 -3.8742911

H 6.9351042 0.8152518 -2.4727851

C 2.6624212 -2.1782342 2.1924399

C 2.2253882 0.0700548 2.7398839

C 3.0611162 -2.4166812 3.5159009

C 2.6105302 -0.1177342 4.0577559

C 3.0371552 -1.3907642 4.4489869

H 3.3894222 -3.4088712 3.8000239

H 2.5764902 0.7113888 4.7546659

H 3.3475262 -1.5771032 5.4721759

C -1.0525898 0.1882238 0.7376289

C -0.9180788 -2.1256852 0.8371139

C -2.3255898 0.1547288 1.2917129

C -2.1930638 -2.2172582 1.3800429

H -0.3272368 -3.0078162 0.6250309

C -2.9444318 -1.0625202 1.6001999

H -2.8626438 1.0804578 1.4498319

H -2.6040578 -3.1987622 1.5629989

C 1.5876272 2.4229148 -0.4298241

C -0.3811058 1.4380278 0.3256189

C 0.9923482 3.6712708 -0.4931481

H 2.6232472 2.2630388 -0.7093711

C -1.0341198 2.6736418 0.2660249

C -0.3610428 3.8230848 -0.1473951

H 1.5836672 4.5153678 -0.8279581

H -2.0830578 2.7197948 0.5181719

N -0.3534658 -0.9549742 0.5217719

N 0.9215502 1.3264468 -0.0286221

H 4.6735912 -0.1959252 1.0389539

H 1.8903262 1.0309898 2.3690859

N 1.3338222 -0.6049102 -2.1858781

N 2.2509822 -0.9235612 1.8335619

H 1.8094362 -3.2130452 -2.1931241

C -1.0380168 5.1912128 -0.2495491

C -2.5144808 5.1421258 0.1750669

C -0.2935558 6.1883118 0.6655209

C -0.9623988 5.6742718 -1.7151191

H -3.0988228 4.4704268 -0.4612961

H -2.6278448 4.8211618 1.2156159

H -2.9519248 6.1406778 0.0889079

H 0.7568962 6.2965628 0.3814139

H -0.7607328 7.1759018 0.5994609

H -0.3296138 5.8633028 1.7100849

H -1.4406138 6.6542548 -1.8102351

H 0.0712922 5.7716438 -2.0583971

H -1.4760858 4.9764048 -2.3830561

C -4.3934138 -1.0781332 2.0835569

C -4.5005998 -0.2794612 3.4001239

C -5.2720638 -0.4208592 0.9924299

C -4.9016568 -2.5085462 2.3228729

H -3.8878958 -0.7313442 4.1874629

H -4.1797968 0.7599018 3.2771279

H -5.5400158 -0.2661992 3.7433059

H -5.1566738 -0.9340502 0.0375609

H -6.3232108 -0.4622872 1.2969189

H -5.0112178 0.6323118 0.8466429

H -5.9390788 -2.4710942 2.6682219

H -4.8712028 -3.0898192 1.3994269

H -4.3166228 -3.0245412 3.0922719

P -3.3878838 -2.2803972 -2.1544731

F -2.8480288 -0.9127132 -1.3905191

F -1.9046608 -2.9517412 -1.8895531

F -3.8939788 -2.8785242 -0.7085891

F -3.8824378 -3.6247942 -2.9021531

F -4.8351688 -1.5690522 -2.3625071

F -2.8555178 -1.6590952 -3.5742711

#### [Pd^0^(Xantphos]

| 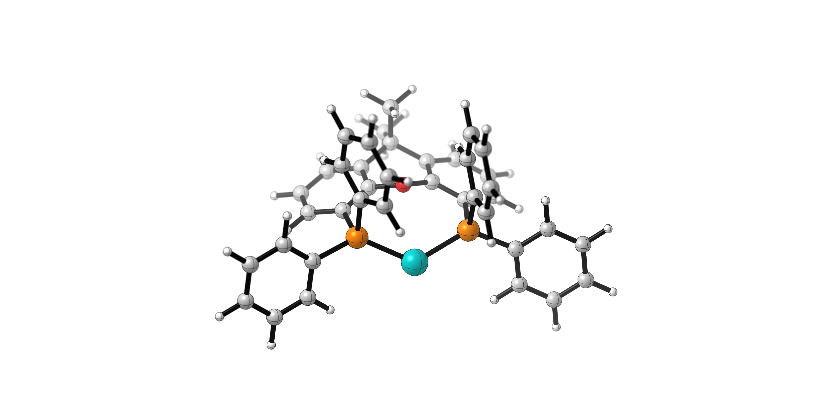 | E/hartree | -2391.593260 |
| --- | --- | --- |
|  | E+zvp/hartree | -2390.995062 |
|  | G/hartree | -2391.066697 |
|  | E(_SP)_/hartree | -2392.366599 |

C -3.3700094 1.7215036 0.9136490

C -2.1970404 1.0086996 0.6250340

C -1.0697534 1.7584546 0.2520070

C -1.0686654 3.1555476 0.1722060

C -2.2529094 3.8257176 0.4870340

C -3.3958494 3.1130686 0.8500010

C 1.3921226 3.0562546 0.2823640

C 1.2738236 1.6655496 0.3439720

C 2.3049966 0.8230476 0.7854380

C 3.5040996 1.4290076 1.1821150

C 3.6441686 2.8163996 1.1515730

C 2.5972116 3.6238966 0.7062440

H -4.2619144 1.1814876 1.2072460

H -2.2904754 4.9080876 0.4441820

H -4.3120104 3.6455606 1.0857350

H 4.3246606 0.8092976 1.5249010

H 4.5770586 3.2705346 1.4709750

H 2.7270006 4.6997636 0.6823730

C 0.2156366 3.8318926 -0.3139460

C 0.2590976 5.3200526 0.0514910

H -0.5742264 5.8550896 -0.4108720

H 0.2120566 5.4711346 1.1337520

H 1.1759546 5.7811996 -0.3241750

C 0.2817016 3.6900106 -1.8592840

H 1.2051256 4.1379206 -2.2399930

H 0.2638116 2.6384316 -2.1562090

H -0.5720294 4.1950916 -2.3222610

O 0.0898246 1.0725546 -0.0515000

P -2.0635234 -0.8225314 0.8466680

P 1.9958306 -0.9945874 0.8674030

Pd -0.0646904 -1.4012764 1.7584300

C -3.7523874 -1.2514214 1.4457450

C -4.8650864 -1.3561654 0.5978130

C -3.9179124 -1.4725544 2.8191270

C -6.1193224 -1.6721134 1.1182810

H -4.7448424 -1.1919784 -0.4681990

C -5.1748324 -1.7802824 3.3411060

H -3.0507624 -1.4072404 3.4712050

C -6.2765724 -1.8822854 2.4906250

H -6.9748184 -1.7539664 0.4539860

H -5.2915494 -1.9497154 4.4074490

H -7.2540664 -2.1304704 2.8935880

C -2.1153694 -1.4682214 -0.8780960

C -1.9248964 -0.6626584 -2.0056180

C -2.3022624 -2.8491984 -1.0535800

C -1.9234944 -1.2252704 -3.2833940

H -1.7805174 0.4051596 -1.8913690

C -2.3221474 -3.4052484 -2.3294970

H -2.4389814 -3.4855674 -0.1836870

C -2.1289824 -2.5933874 -3.4507170

H -1.7645354 -0.5886204 -4.1486950

H -2.4804954 -4.4732104 -2.4497380

H -2.1326644 -3.0276094 -4.4460570

C 3.6410876 -1.6207454 1.4126640

C 4.7068386 -1.8746384 0.5382800

C 3.8180716 -1.8428034 2.7859260

C 5.9277326 -2.3350014 1.0319780

H 4.5795066 -1.7153424 -0.5272100

C 5.0428216 -2.2903764 3.2801560

H 2.9847806 -1.6655414 3.4611540

C 6.0996956 -2.5393194 2.4024910

H 6.7459886 -2.5336444 0.3457270

H 5.1685426 -2.4554214 4.3462120

H 7.0511666 -2.8980814 2.7838750

C 1.9522286 -1.4270274 -0.9226170

C 2.5570936 -0.6327154 -1.9062980

C 1.2634986 -2.5844624 -1.3092310

C 2.4799526 -0.9954624 -3.2515690

H 3.0808546 0.2738216 -1.6203300

C 1.1971226 -2.9516894 -2.6515320

H 0.7548096 -3.1740914 -0.5528670

C 1.8024806 -2.1566324 -3.6257790

H 2.9473546 -0.3693494 -4.0063430

H 0.6467306 -3.8412104 -2.9379710

H 1.7370586 -2.4351014 -4.6735440

#### TS-1 = TS-oxidative addition

| 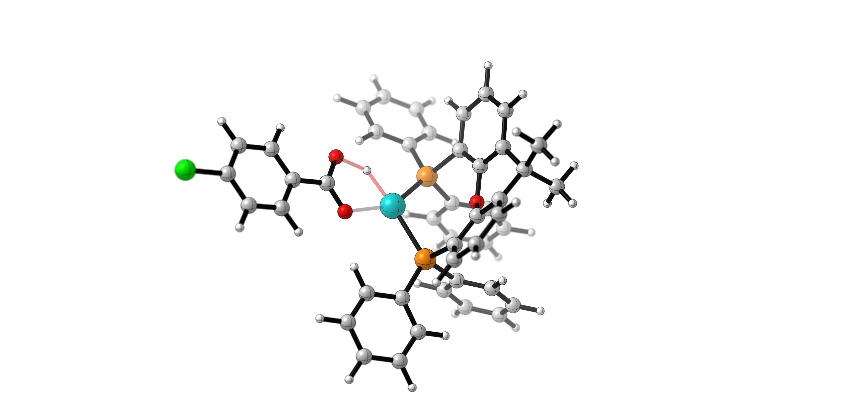 | E/hartree | -3272.059732 |
| --- | --- | --- |
|  | E+zvp/hartree | -3271.359819 |
|  | G/hartree | -3271.444850 |
|  | E(_SP)_/hartree | -3273.075260 |
|  | Imaginary frequency/cm^-1^ | -601.34 |

C 1.2884085 3.2733348 1.9086632

C 1.2343615 2.0757948 1.1826642

C 1.5164735 0.8940088 1.8777152

C 1.8078485 0.8444978 3.2430002

C 1.8371405 2.0585528 3.9344622

C 1.5901175 3.2610828 3.2700242

C 1.1797955 -1.5414202 3.1733442

C 0.9396865 -1.3726232 1.8092012

C 0.1725025 -2.2496432 1.0358452

C -0.3780625 -3.3628332 1.6822492

C -0.1695525 -3.5564382 3.0484892

C 0.5990025 -2.6548532 3.7882512

H 1.0687085 4.2108298 1.4126142

H 2.0530625 2.0719468 4.9966262

H 1.6208815 4.1958268 3.8209102

H -0.9791875 -4.0653732 1.1163272

H -0.6083155 -4.4180292 3.5418922

H 0.7475115 -2.8272632 4.8481552

C 2.1079415 -0.5292232 3.8522622

C 1.9445975 -0.5303302 5.3764872

H 2.6369145 0.1775328 5.8393142

H 0.9256935 -0.2645612 5.6719162

H 2.1798605 -1.5153852 5.7873782

C 3.5714565 -0.9081262 3.4980182

H 3.8108815 -1.9003882 3.8932712

H 3.7189865 -0.9263502 2.4151212

H 4.2661145 -0.1801792 3.9288722

O 1.4745005 -0.2805582 1.1541912

P 0.6053195 1.9619188 -0.5485388

P -0.1327295 -1.7898132 -0.7191048

Pd -1.0880695 0.4937448 -0.6777308

C 0.3925335 3.7375448 -0.9957128

C 1.5136695 4.5634688 -1.1850408

C -0.8878485 4.2857918 -1.1303828

C 1.3537195 5.9115488 -1.4949178

H 2.5110395 4.1461208 -1.0914278

C -1.0454835 5.6382888 -1.4430348

H -1.7615735 3.6599368 -0.9947368

C 0.0712815 6.4521248 -1.6251718

H 2.2278865 6.5398658 -1.6383698

H -2.0446195 6.0503548 -1.5477278

H -0.0532745 7.5025338 -1.8714188

C 2.0845725 1.5656008 -1.5701598

C 3.2891655 1.0918538 -1.0419328

C 1.9776645 1.7650648 -2.9557288

C 4.3724135 0.8338228 -1.8836308

H 3.3899335 0.9300228 0.0240362

C 3.0646335 1.5236468 -3.7906548

H 1.0440095 2.1293148 -3.3749908

C 4.2674285 1.0574458 -3.2550578

H 5.2997745 0.4576938 -1.4626558

H 2.9726635 1.6955008 -4.8591628

H 5.1143275 0.8611078 -3.9056158

C -1.1128745 -3.2245332 -1.3219798

C -0.5211275 -4.4483722 -1.6691568

C -2.5053575 -3.0812682 -1.4134838

C -1.3110545 -5.5150102 -2.0950678

H 0.5562255 -4.5629022 -1.6085608

C -3.2913795 -4.1548432 -1.8363918

H -2.9638345 -2.1323922 -1.1551298

C -2.6981035 -5.3704822 -2.1778748

H -0.8445675 -6.4583212 -2.3640998

H -4.3689415 -4.0368232 -1.9072198

H -3.3117575 -6.2018382 -2.5125078

C 1.5008735 -2.0963862 -1.4999478

C 2.5562125 -2.7497882 -0.8518128

C 1.6681305 -1.6794392 -2.8274838

C 3.7545595 -2.9916852 -1.5250078

H 2.4389505 -3.0766352 0.1759042

C 2.8582775 -1.9364632 -3.5026698

H 0.8652925 -1.1439112 -3.3253338

C 3.9050365 -2.5929292 -2.8528668

H 4.5672375 -3.4984132 -1.0123198

H 2.9769885 -1.6043502 -4.5287948

H 4.8368815 -2.7832162 -3.3770728

H -2.0647295 1.8004068 -0.3599008

O -3.2389785 -0.0846542 -0.5437228

C -3.9129755 0.9554788 -0.2837858

O -3.3930165 2.1133798 -0.1417488

C -5.3912975 0.8299468 -0.1314588

C -6.0024995 -0.4251762 -0.2367298

C -6.1759855 1.9618098 0.1188932

C -7.3807535 -0.5542062 -0.0955408

H -5.3832995 -1.2941052 -0.4264228

C -7.5560535 1.8478108 0.2602822

H -5.6896915 2.9268348 0.2009152

C -8.1438275 0.5875438 0.1510902

H -7.8626585 -1.5219312 -0.1739638

H -8.1716165 2.7187868 0.4533132

Cl -9.8840505 0.4337418 0.3292632

#### Int-1 = [(H)(BzO)Pd^II^(Xantphos]

| 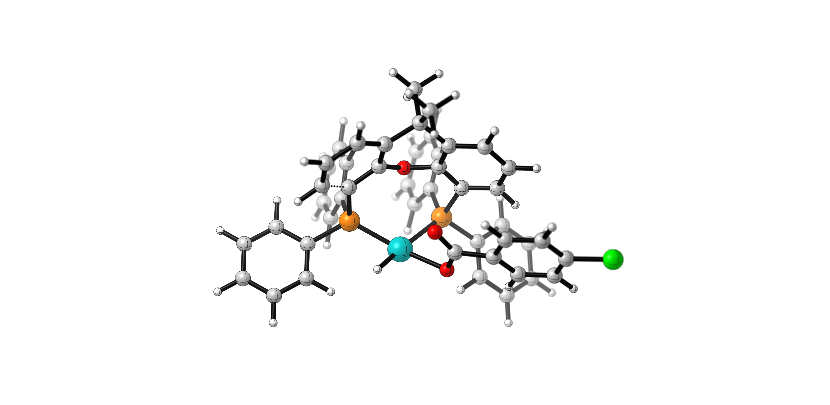 | E/hartree | -3272.070642 |
| --- | --- | --- |
|  | E+zvp/hartree | -3271.368333 |
|  | G/hartree | -3271.451420 |
|  | E(_SP)_/hartree | -3273.087068 |

C -2.5816383 -3.2612368 -0.1019559

C -2.0022253 -1.9965418 0.0606711

C -1.0009903 -1.8655968 1.0309641

C -0.5155653 -2.9284058 1.7935041

C -1.1086473 -4.1772238 1.5914421

C -2.1421183 -4.3376528 0.6669881

C 1.5049707 -1.5562798 2.2058201

C 0.8998607 -0.5583318 1.4485921

C 1.5893987 0.5031892 0.8576311

C 2.9720627 0.5694512 1.0533221

C 3.6136977 -0.4135178 1.8071561

C 2.8906557 -1.4658208 2.3721071

H -3.3492033 -3.4097828 -0.8517389

H -0.7634623 -5.0368278 2.1540961

H -2.5927803 -5.3151918 0.5286481

H 3.5473537 1.3563942 0.5806321

H 4.6909247 -0.3757918 1.9291271

H 3.4185157 -2.2259288 2.9364101

C 0.5980187 -2.6348078 2.8073391

C 1.3852887 -3.9001098 3.1687801

H 0.7310627 -4.6474288 3.6248601

H 1.8574927 -4.3387918 2.2856791

H 2.1613317 -3.6723038 3.9038701

C -0.0563063 -2.0544778 4.0895721

H 0.7128307 -1.8036428 4.8268801

H -0.6215273 -1.1465668 3.8621901

H -0.7418663 -2.7850778 4.5306451

O -0.4614993 -0.6075048 1.2057551

P -2.2777913 -0.6122438 -1.1231849

P 0.6272417 1.5318292 -0.3119609

Pd -0.2069183 -0.1449408 -1.8689949

C -3.5458723 -1.2648398 -2.2758989

C -4.8648303 -1.4559518 -1.8295799

C -3.2170523 -1.5879468 -3.5965469

C -5.8297033 -1.9744088 -2.6890429

H -5.1327943 -1.1965918 -0.8105119

C -4.1883643 -2.1022128 -4.4579799

H -2.1997613 -1.4350138 -3.9404919

C -5.4925933 -2.2977838 -4.0064589

H -6.8448203 -2.1229998 -2.3334059

H -3.9223973 -2.3464358 -5.4818309

H -6.2472773 -2.6971508 -4.6773979

C -3.2510063 0.6600052 -0.2309629

C -3.3364923 0.7117272 1.1640541

C -3.9422433 1.6106212 -0.9983239

C -4.1173533 1.6895232 1.7809141

H -2.7993003 -0.0076448 1.7687761

C -4.7298773 2.5769322 -0.3793139

H -3.8807473 1.5769772 -2.0820999

C -4.8211833 2.6159882 1.0135781

H -4.1708493 1.7268182 2.8642551

H -5.2712643 3.2992272 -0.9830139

H -5.4311633 3.3721942 1.4981951

C 1.8364937 2.7442832 -0.9631599

C 2.4048777 3.7161802 -0.1256379

C 2.1900387 2.7093002 -2.3184899

C 3.3301077 4.6263852 -0.6326869

H 2.1180367 3.7608912 0.9204171

C 3.1117827 3.6280742 -2.8236259

H 1.7665867 1.9460882 -2.9597669

C 3.6854897 4.5823982 -1.9830859

H 3.7698587 5.3717802 0.0232211

H 3.3827967 3.5935372 -3.8745899

H 4.4049667 5.2935202 -2.3781109

C -0.3986153 2.5947062 0.7747421

C -0.3021943 2.5717612 2.1707231

C -1.2745293 3.5016282 0.1625451

C -1.0599563 3.4543802 2.9420241

H 0.3731117 1.8736312 2.6531921

C -2.0217193 4.3872592 0.9333821

H -1.3691963 3.5114502 -0.9193279

C -1.9144183 4.3674352 2.3256761

H -0.9751563 3.4315352 4.0246421

H -2.6989803 5.0832822 0.4490001

H -2.5009963 5.0563882 2.9260771

H -0.5310783 -1.3353208 -2.8150939

O 1.8440937 -0.2968018 -2.3618009

C 2.3828927 -1.2875088 -1.7137409

O 1.7753807 -2.1900708 -1.1257759

C 3.8888477 -1.2375718 -1.6436829

C 4.6082847 -0.1303818 -2.1044319

C 4.5691277 -2.2816658 -1.0093869

C 5.9889427 -0.0570348 -1.9274139

H 4.0743217 0.6820362 -2.5815869

C 5.9487127 -2.2295428 -0.8318459

H 3.9868957 -3.1185958 -0.6411699

C 6.6435887 -1.1098998 -1.2902039

H 6.5514477 0.8034182 -2.2721529

H 6.4823867 -3.0350908 -0.3398829

Cl 8.3865527 -1.0217308 -1.0507509

#### Int-2 = [(H)(BzO)(allene)Pd^II^(Xantphos]

| 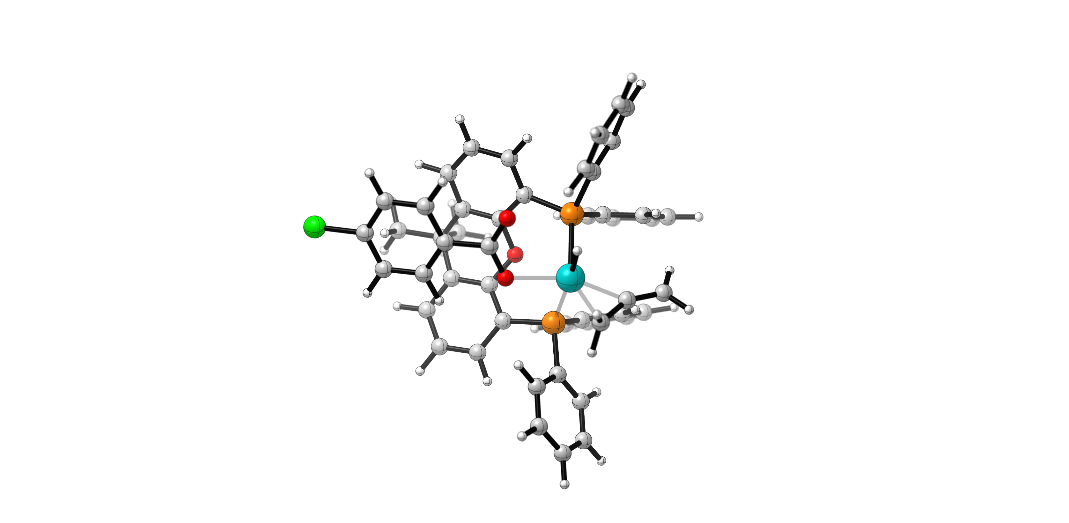 | E/hartree | -3388.764026 |
| --- | --- | --- |
|  | E+zvp/hartree | -3388.002006 |
|  | G/hartree | -3388.087043 |
|  | E(_SP)_/hartree | -3389.822814 |

C 1.8323707 3.1280732 0.4147836

C 0.9525357 2.0396012 0.4856966

C 1.4234677 0.8761312 1.0932276

C 2.7029257 0.7392952 1.6368006

C 3.5552907 1.8392912 1.5356456

C 3.1206907 3.0224892 0.9334576

C 2.3702247 -1.7009328 1.5393756

C 1.1187367 -1.4361248 0.9830016

C 0.3771867 -2.3644348 0.2520646

C 0.9163567 -3.6471918 0.1016926

C 2.1663607 -3.9487858 0.6456826

C 2.8886427 -2.9846858 1.3517926

H 1.5169377 4.0405722 -0.0750894

H 4.5673097 1.7762822 1.9175916

H 3.7989467 3.8663552 0.8584676

H 0.3703277 -4.4006928 -0.4525764

H 2.5808797 -4.9435888 0.5167896

H 3.8625647 -3.2372168 1.7548696

C 3.0347197 -0.5795458 2.3414376

C 2.3808337 -0.5498398 3.7500026

H 2.7915077 0.2757962 4.3393996

H 1.2980057 -0.4170768 3.6784356

H 2.5761227 -1.4894368 4.2765736

C 4.5450827 -0.7891338 2.4956336

H 4.7517327 -1.7269508 3.0179396

H 5.0488047 -0.8077068 1.5276456

H 4.9814857 0.0093372 3.1011216

O 0.5479587 -0.1928308 1.1356776

P -0.6860473 2.0104132 -0.3444594

P -1.2023833 -1.7829008 -0.4808004

Pd -1.0442743 0.2126502 -1.9624874

C -1.9308593 2.0299282 1.0086466

C -3.2306913 2.4594002 0.6933306

C -1.6619733 1.5947492 2.3112646

C -4.2329583 2.4632672 1.6594496

H -3.4450473 2.8257432 -0.3052034

C -2.6716033 1.5866522 3.2743646

H -0.6679823 1.2646882 2.5810836

C -3.9562923 2.0219932 2.9550596

H -5.2283873 2.8123832 1.4008046

H -2.4489313 1.2368292 4.2775886

H -4.7377553 2.0164462 3.7087666

C -0.8034403 3.7246862 -1.0002814

C -1.2449813 4.7860592 -0.1948514

C -0.3683923 3.9905552 -2.3060514

C -1.2810653 6.0855382 -0.6985844

H -1.5637173 4.5983592 0.8241566

C -0.4028583 5.2936692 -2.8032804

H 0.0430767 3.1847822 -2.9029114

C -0.8659793 6.3413002 -2.0066884

H -1.6311913 6.8971072 -0.0675154

H -0.0616063 5.4873612 -3.8157204

H -0.8964013 7.3535502 -2.3991844

C -2.3452643 -1.7386918 0.9466826

C -2.0347953 -2.3221648 2.1814896

C -3.5831223 -1.1027798 0.7754366

C -2.9544413 -2.2749218 3.2291496

H -1.0776593 -2.8126408 2.3218106

C -4.5043603 -1.0721378 1.8197516

H -3.8182143 -0.6345698 -0.1736424

C -4.1915313 -1.6555548 3.0484866

H -2.7050143 -2.7264858 4.1848806

H -5.4573453 -0.5725788 1.6797206

H -4.9065483 -1.6203308 3.8650936

C -1.7256003 -3.2569798 -1.4346834

C -0.9786783 -3.5758278 -2.5828074

C -2.8331663 -4.0400178 -1.0950914

C -1.3309573 -4.6725918 -3.3658204

H -0.1269193 -2.9571858 -2.8535834

C -3.1901753 -5.1311028 -1.8912604

H -3.4140793 -3.7999658 -0.2113544

C -2.4407183 -5.4506648 -3.0228684

H -0.7460813 -4.9145058 -4.2480844

H -4.0532863 -5.7326118 -1.6220564

H -2.7197593 -6.3003888 -3.6385404

H -0.8259833 1.3621242 -2.9370154

C -2.2020963 -0.6017898 -3.6058884

H -1.8409583 -0.1846288 -4.5417794

H -2.2427043 -1.6846818 -3.5523604

C -2.9712723 0.1745702 -2.7440934

C -4.1415473 0.6842142 -2.4023984

H -4.2701283 1.2982032 -1.5195014

H -5.0245583 0.4948262 -3.0107064

O 1.0746267 -0.3769998 -2.0507924

C 1.9327867 0.5632742 -2.2396464

O 1.6957817 1.7163462 -2.6367704

C 3.3506607 0.1932572 -1.8719754

C 3.6833587 -1.1145348 -1.5062024

C 4.3340997 1.1862192 -1.8415744

C 4.9811247 -1.4329678 -1.1130094

H 2.9104297 -1.8730498 -1.5164924

C 5.6333957 0.8896372 -1.4372514

H 4.0504217 2.1936742 -2.1224854

C 5.9403587 -0.4215578 -1.0752074

H 5.2432557 -2.4440258 -0.8229444

H 6.3981527 1.6571742 -1.3968564

Cl 7.5722497 -0.8068428 -0.5313964

#### TS-2 = TS-hydrometalation

| 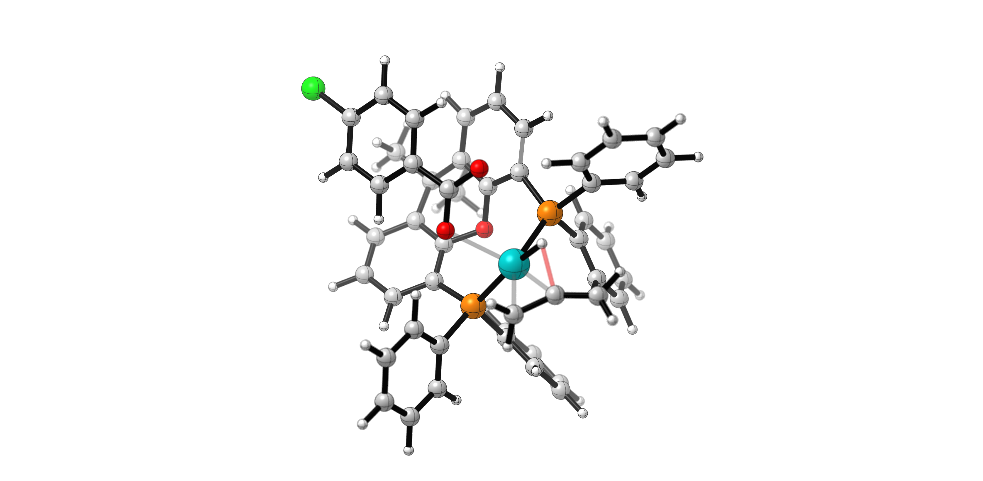 | E/hartree | -3388.755649 |
| --- | --- | --- |
|  | E+zvp/hartree | -3387.995986 |
|  | G/hartree | -3388.082256 |
|  | E(_SP)_/hartree | -3389.817468 |
|  | Imaginary frequency/cm^-1^ | -653.45 |

C 1.6693315 3.2748781 0.1355592

C 0.8674935 2.1498581 0.3543432

C 1.4279875 1.0804241 1.0490582

C 2.7330935 1.0633511 1.5393312

C 3.5141165 2.1933881 1.2890132

C 2.9846145 3.2872891 0.5979262

C 2.5274445 -1.3863159 1.6722932

C 1.2446965 -1.2372849 1.1381172

C 0.5554085 -2.2512559 0.4729732

C 1.1759345 -3.5059919 0.3936452

C 2.4460855 -3.6969209 0.9361612

C 3.1212525 -2.6441649 1.5586472

H 1.2722255 4.1176861 -0.4175458

H 4.5442055 2.2237041 1.6242172

H 3.6100455 4.1541131 0.4103272

H 0.6767735 -4.3213589 -0.1153788

H 2.9200075 -4.6705009 0.8614622

H 4.1192285 -2.8068379 1.9489222

C 3.1551695 -0.1646969 2.3510412

C 2.5404555 -0.0324399 3.7713382

H 2.9405275 0.8543991 4.2727342

H 1.4525565 0.0625371 3.7220642

H 2.7837485 -0.9147359 4.3718052

C 4.6778795 -0.2839469 2.4714822

H 4.9495885 -1.1511879 3.0791572

H 5.1524545 -0.3772439 1.4932652

H 5.0897085 0.5942281 2.9754682

O 0.6096185 -0.0191329 1.2334142

P -0.8306625 1.9145111 -0.2761988

P -1.0390695 -1.8629359 -0.3575968

Pd -1.3058535 0.1182691 -1.7521408

C -1.8496155 1.8227251 1.2435332

C -3.1433075 1.2946701 1.1375242

C -1.3881685 2.2668231 2.4891322

C -3.9672965 1.2239061 2.2586072

H -3.4900215 0.9264671 0.1770872

C -2.2097115 2.1795021 3.6129722

H -0.3877615 2.6766951 2.5792862

C -3.5001145 1.6599711 3.4991832

H -4.9621885 0.8021761 2.1675502

H -1.8415255 2.5190841 4.5766532

H -4.1375255 1.5896461 4.3755682

C -1.2662175 3.5435841 -0.9962518

C -2.1037665 4.4492861 -0.3327808

C -0.7617035 3.8625291 -2.2688318

C -2.4425525 5.6608251 -0.9383818

H -2.4942065 4.2094851 0.6499422

C -1.0967275 5.0801531 -2.8593258

H -0.1004435 3.1579371 -2.7672628

C -1.9404025 5.9781801 -2.2004948

H -3.0979405 6.3552931 -0.4209078

H -0.7003015 5.3245891 -3.8403858

H -2.2053185 6.9212601 -2.6696098

C -2.3062615 -2.0326249 0.9513072

C -1.9846655 -2.0728869 2.3118842

C -3.6575635 -2.0358209 0.5671912

C -2.9962455 -2.1264399 3.2720092

H -0.9458115 -2.0629249 2.6220022

C -4.6638005 -2.1169239 1.5256272

H -3.9185545 -1.9797619 -0.4855808

C -4.3344925 -2.1593079 2.8830772

H -2.7347055 -2.1481099 4.3255392

H -5.7043535 -2.1351129 1.2151002

H -5.1188905 -2.2101639 3.6321282

C -1.2426055 -3.3537409 -1.4074228

C -0.5014495 -3.3848419 -2.6022508

C -2.0596625 -4.4378919 -1.0695448

C -0.5874095 -4.4924599 -3.4421788

H 0.1364145 -2.5406119 -2.8521428

C -2.1486375 -5.5402819 -1.9237128

H -2.6249235 -4.4253319 -0.1441358

C -1.4144165 -5.5694509 -3.1087808

H -0.0111855 -4.5133099 -4.3624738

H -2.7888685 -6.3761819 -1.6574808

H -1.4843165 -6.4275239 -3.7707208

H -1.6613245 1.2651571 -2.7655458

C -2.5582015 -0.8565259 -3.2279268

H -1.9637535 -0.9947589 -4.1271278

H -3.0379935 -1.7462199 -2.8331188

C -3.0481935 0.4103771 -2.8928088

C -4.0363335 1.2964651 -2.9168868

H -3.9686845 2.2370631 -2.3811918

H -4.9307115 1.1069241 -3.5050728

O 0.9093885 -0.5260849 -2.2622288

C 1.6758165 0.4887931 -2.4061828

O 1.3390995 1.6310131 -2.7785128

C 3.1234075 0.2644171 -2.0193398

C 3.5562685 -0.9808759 -1.5539608

C 4.0295645 1.3274521 -2.0729178

C 4.8736455 -1.1676819 -1.1415278

H 2.8424995 -1.7941909 -1.5030408

C 5.3492225 1.1626391 -1.6592248

H 3.6681295 2.2857071 -2.4269958

C 5.7539005 -0.0877229 -1.1938758

H 5.2107975 -2.1288959 -0.7707958

H 6.0543175 1.9859601 -1.6871878

Cl 7.4122965 -0.3037799 -0.6316848

#### Int-3 = [(π-allyl)(BzO)Pd^II^(Xantphos]

| 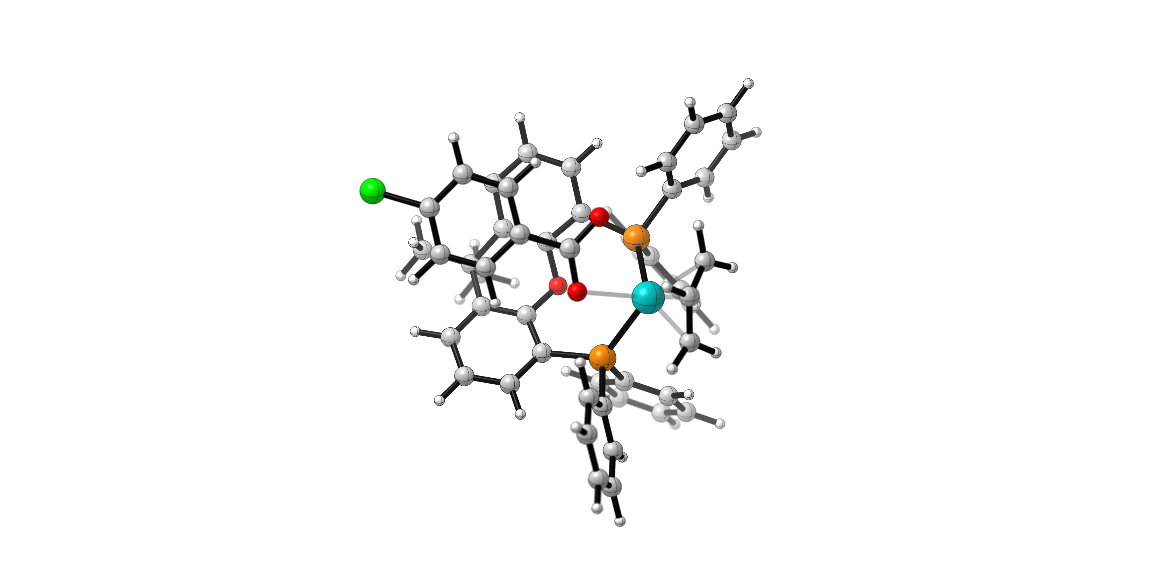 | E/hartree | -3388.818197 |
| --- | --- | --- |
|  | E+zvp/hartree | -3388.051815 |
|  | G/hartree | -3388.137458 |
|  | E(_SP)_/hartree | -3389.876556 |

C 2.1903670 2.9022884 0.1586174

C 1.1929630 1.9329484 0.3200514

C 1.5261300 0.7879824 1.0388304

C 2.7865420 0.5377044 1.5808704

C 3.7673760 1.5081374 1.3717704

C 3.4660380 2.6816014 0.6758604

C 2.1358370 -1.8305696 1.6976164

C 0.9180240 -1.4510846 1.1270924

C 0.0718740 -2.3202626 0.4420974

C 0.4576660 -3.6667896 0.3690504

C 1.6602290 -4.0838266 0.9375714

C 2.4974890 -3.1733726 1.5878114

H 1.9754970 3.8133724 -0.3848416

H 4.7715830 1.3535304 1.7486684

H 4.2391770 3.4284784 0.5268824

H -0.1688610 -4.3794426 -0.1520306

H 1.9529200 -5.1264446 0.8652024

H 3.4380610 -3.5153606 2.0041654

C 2.9449720 -0.7416056 2.4072794

C 2.3020040 -0.4922306 3.7995624

H 2.8364360 0.3077544 4.3213394

H 1.2537070 -0.1981456 3.7033724

H 2.3527160 -1.4016936 4.4066024

C 4.4130980 -1.1345296 2.5977184

H 4.4927740 -2.0347536 3.2130974

H 4.9106690 -1.3150086 1.6438184

H 4.9533920 -0.3440666 3.1250114

O 0.5143710 -0.1373916 1.2085844

P -0.5272960 2.0363864 -0.3119626

P -1.4375040 -1.6524836 -0.3719196

Pd -1.4386840 0.3351354 -1.6749326

C -1.4981280 2.2086704 1.2362754

C -2.8498080 1.8442514 1.2206634

C -0.9341570 2.7024474 2.4202284

C -3.6297930 1.9758424 2.3684894

H -3.2765420 1.4377004 0.3091634

C -1.7124440 2.8222704 3.5707484

H 0.1124050 2.9875514 2.4410834

C -3.0608470 2.4599114 3.5466014

H -4.6714990 1.6755734 2.3470074

H -1.2657540 3.1989574 4.4862124

H -3.6642710 2.5503054 4.4449744

C -0.5697670 3.7227154 -1.0347226

C -1.3779460 4.7340094 -0.5021326

C 0.2145270 3.9858424 -2.1727226

C -1.4043360 5.9971434 -1.0977936

H -1.9829910 4.5413984 0.3766354

C 0.1906480 5.2536364 -2.7513476

H 0.8239250 3.1884404 -2.5922746

C -0.6190020 6.2608484 -2.2191536

H -2.0363470 6.7746004 -0.6786316

H 0.8022150 5.4520704 -3.6266846

H -0.6380490 7.2448564 -2.6783396

C -2.6798450 -1.5745806 0.9740474

C -2.3425850 -1.7035366 2.3249254

C -4.0132250 -1.3023606 0.6258784

C -3.3215180 -1.5660546 3.3107974

H -1.3174660 -1.9118306 2.6090984

C -4.9929080 -1.1932636 1.6086904

H -4.2794670 -1.1824756 -0.4203206

C -4.6470200 -1.3200066 2.9570444

H -3.0452220 -1.6579516 4.3567624

H -6.0230570 -0.9985076 1.3250434

H -5.4075660 -1.2220046 3.7257394

C -1.9296630 -3.1177616 -1.3656836

C -1.1914670 -3.3716126 -2.5363386

C -2.9585460 -3.9887456 -0.9924626

C -1.4971720 -4.4829436 -3.3189146

H -0.3872550 -2.6938896 -2.8132036

C -3.2640620 -5.0950846 -1.7898986

H -3.5202100 -3.8116856 -0.0822996

C -2.5364860 -5.3436736 -2.9526916

H -0.9235470 -4.6752186 -4.2208576

H -4.0677840 -5.7634146 -1.4950916

H -2.7745100 -6.2047746 -3.5701346

H -1.1583210 0.1920384 -4.3968406

C -2.8236000 -0.5121986 -3.2270166

H -2.6936890 -1.5473506 -3.5142886

H -3.7816080 -0.2478036 -2.7846466

C -2.0077590 0.4829594 -3.7854716

C -2.0250660 1.7779874 -3.2297816

H -1.2665580 2.4893334 -3.5239816

H -2.9442470 2.1891224 -2.8181766

O 0.6889180 -0.9139086 -2.5906716

C 1.5242060 0.0420464 -2.6182666

O 1.3074850 1.2173694 -3.0062066

C 2.9129910 -0.2712306 -2.0867196

C 3.2034710 -1.5305116 -1.5531526

C 3.9123650 0.7054764 -2.1107106

C 4.4680670 -1.8145576 -1.0424206

H 2.4189070 -2.2776986 -1.5367626

C 5.1834070 0.4422024 -1.6056196

H 3.6634400 1.6763814 -2.5226616

C 5.4432990 -0.8188056 -1.0726756

H 4.6939930 -2.7874306 -0.6209236

H 5.9598470 1.1991064 -1.6137166

Cl 7.0394230 -1.1564496 -0.3965566

#### Int-4 = [(π-allyl)Pd^I^(Xantphos]

| 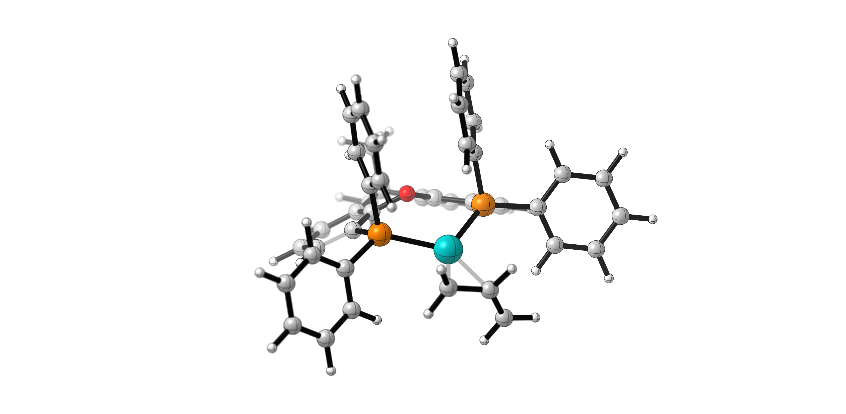 | E/hartree | -2508.908954 |
| --- | --- | --- |
|  | E+zvp/hartree | -2508.242042 |
|  | G/hartree | -2508.319838 |
|  | E(_SP)_/hartree | -2509.732048 |

C -3.3719530 1.7584984 -1.0148637

C -2.1712060 1.1976834 -0.5530907

C -1.0699230 2.0542614 -0.4503017

C -1.0910810 3.4018754 -0.8216427

C -2.2999780 3.9153614 -1.2971417

C -3.4322040 3.1019274 -1.3810387

C 1.3656410 3.2759484 -0.9789327

C 1.2567040 1.9423884 -0.5822487

C 2.2592880 0.9867484 -0.7690697

C 3.4457460 1.4122614 -1.3790737

C 3.5857160 2.7360174 -1.7988627

C 2.5557130 3.6590494 -1.6049117

H -4.2534050 1.1353744 -1.1053887

H -2.3658410 4.9537504 -1.6013877

H -4.3675660 3.5182824 -1.7420187

H 4.2509950 0.7035714 -1.5348877

H 4.5066520 3.0525824 -2.2786457

H 2.6878680 4.6821814 -1.9382107

C 0.1992460 4.2057444 -0.6321797

C 0.3187450 4.5867504 0.8684493

H -0.5255970 5.2160174 1.1673143

H 0.3242640 3.6939754 1.4988113

H 1.2482440 5.1372424 1.0451473

C 0.2104670 5.4849174 -1.4767507

H 1.1335370 6.0463814 -1.3114787

H 0.1249290 5.2630444 -2.5443147

H -0.6142650 6.1429154 -1.1912577

O 0.1001460 1.5068784 0.0332763

P -1.9324460 -0.6089116 -0.2402337

P 1.8831320 -0.7421746 -0.2538097

Pd -0.0798050 -1.8409426 -0.9766517

C -2.0316680 -0.7488266 1.5882123

C -2.1694220 -2.0350716 2.1368333

C -1.9148170 0.3473674 2.4487583

C -2.2204850 -2.2132636 3.5165173

H -2.2419730 -2.8920346 1.4738493

C -1.9406190 0.1631954 3.8321813

H -1.8066040 1.3461614 2.0419023

C -2.1024480 -1.1127566 4.3697383

H -2.3425500 -3.2115936 3.9269593

H -1.8388220 1.0218484 4.4891053

H -2.1301670 -1.2518836 5.4463563

C -3.5718740 -1.2436666 -0.7840447

C -4.6778220 -1.3723916 0.0659783

C -3.7128300 -1.5772866 -2.1393987

C -5.8986420 -1.8331486 -0.4301037

H -4.5851550 -1.1109746 1.1144503

C -4.9353110 -2.0246666 -2.6369257

H -2.8541840 -1.4852146 -2.7982917

C -6.0306670 -2.1585886 -1.7807527

H -6.7482050 -1.9327986 0.2393093

H -5.0306350 -2.2784146 -3.6885277

H -6.9811960 -2.5173036 -2.1642657

C 1.8818760 -0.5859266 1.5787693

C 2.4749870 0.4891964 2.2535873

C 1.2554800 -1.5957806 2.3212703

C 2.4402880 0.5518424 3.6469183

H 2.9616190 1.2779974 1.6893223

C 1.2332290 -1.5388126 3.7132333

H 0.7662550 -2.4108076 1.7967213

C 1.8215830 -0.4627186 4.3788433

H 2.8978170 1.3929744 4.1599773

H 0.7323470 -2.3201276 4.2743053

H 1.7917430 -0.4111486 5.4632913

C 3.4950920 -1.5603006 -0.5969457

C 3.7189070 -2.0289216 -1.9008507

C 4.4943880 -1.7329986 0.3697933

C 4.9231810 -2.6459176 -2.2345717

H 2.9355510 -1.9206106 -2.6442487

C 5.6937450 -2.3650806 0.0372063

H 4.3348590 -1.3741306 1.3807453

C 5.9128310 -2.8192116 -1.2639967

H 5.0848500 -3.0015436 -3.2480887

H 6.4590860 -2.4990816 0.7962863

H 6.8473380 -3.3096876 -1.5199337

C 0.2865390 -3.9534646 -1.8960657

C -1.0559090 -3.7327706 -1.4746867

C 0.7890840 -3.6355506 -3.1614557

H 0.9637860 -4.4505256 -1.2024807

H -1.4095830 -4.2359736 -0.5783777

H 1.8254190 -3.8225426 -3.4140277

H 0.1515290 -3.1964216 -3.9225497

H -1.8235850 -3.5602416 -2.2244327

#### TS-3 = TS-reductive elim.

| 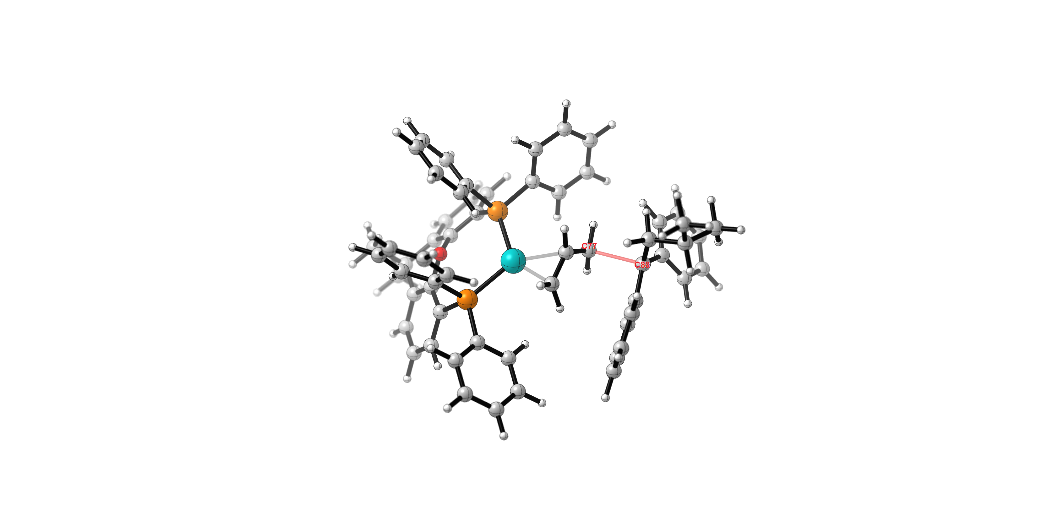  TS with fixed distances between C77-C83 | E/hartree | -3168.277842 |
| --- | --- | --- |
|  | E+zvp/hartree | -3167.295332 |
|  | G/hartree | -3167.392143 |
|  | E(_SP)_/hartree | -3169.375718 |
|  | Imaginary frequency/cm^-1^ | -164.90 |

P 2.1604615 1.8787445 0.0498905

P 1.2347495 -1.8660765 0.4263315

C 3.4928865 1.7022075 -1.2142155

C 4.0430585 0.4374665 -1.4515005

C 3.9286495 2.7532525 -2.0341545

C 4.9716615 0.1720885 -2.4627345

C 4.8533095 2.5226925 -3.0511685

H 3.5281435 3.7486325 -1.8837585

C 3.4510985 -1.8119725 -1.2605755

C 5.3662665 1.2424015 -3.2688365

H 5.1749555 3.3456015 -3.6818565

C 2.3610695 -2.5856105 -0.8480095

C 4.3471155 -2.2017465 -2.2589355

H 6.0797755 1.0833635 -4.0692535

C 2.1980025 -3.8372075 -1.4567745

C 4.1356225 -3.4452595 -2.8605275

C 3.0755015 -4.2584345 -2.4560715

H 1.3730865 -4.4732825 -1.1582395

H 4.8021185 -3.7889535 -3.6433315

H 2.9315235 -5.2271965 -2.9243405

O 3.6244945 -0.5932025 -0.6341715

C -0.0689855 -3.1594135 0.5276645

C -0.3159825 -3.9056575 1.6864685

C -0.9288705 -3.3138655 -0.5735225

C -1.3999195 -4.7853925 1.7423185

H 0.3367925 -3.7986235 2.5459795

C -1.9976395 -4.2056805 -0.5240205

H -0.7650495 -2.7201375 -1.4682475

C -2.2403805 -4.9408195 0.6399275

H -1.5828945 -5.3531825 2.6500065

H -2.6542385 -4.3053815 -1.3827095

H -3.0811305 -5.6264935 0.6861915

C 2.2318655 -2.1265345 1.9497575

C 2.1478015 -1.1743025 2.9731535

C 3.0825785 -3.2298845 2.1082085

C 2.8998495 -1.3212785 4.1386445

H 1.5087005 -0.3081685 2.8356915

C 3.8331645 -3.3765695 3.2737425

H 3.1610485 -3.9680885 1.3162155

C 3.7434695 -2.4217535 4.2899645

H 2.8400735 -0.5641175 4.9130065

H 4.4915605 -4.2329665 3.3876705

H 4.3355385 -2.5339125 5.1936545

C 3.0843615 1.9719755 1.6364335

C 2.3703235 2.3594265 2.7832345

C 4.4226985 1.5883015 1.7687445

C 2.9886875 2.3752505 4.0304385

H 1.3275085 2.6478425 2.6898345

C 5.0356865 1.5868355 3.0233325

H 4.9901905 1.2908095 0.8944275

C 4.3246705 1.9825315 4.1550785

H 2.4271645 2.6866915 4.9065635

H 6.0731805 1.2786175 3.1125165

H 4.8052065 1.9844625 5.1288615

C 1.6453695 3.6216275 -0.2291845

C 2.2782715 4.7186355 0.3715185

C 0.5686695 3.8475165 -1.0990525

C 1.8366985 6.0162425 0.1095585

H 3.1137695 4.5564205 1.0444185

C 0.1348285 5.1443125 -1.3678325

H 0.0633705 2.9998755 -1.5511435

C 0.7660205 6.2314725 -0.7604805

H 2.3303465 6.8593245 0.5842345

H -0.7069395 5.2998985 -2.0345395

H 0.4217585 7.2420885 -0.9593905

C 5.5127755 -1.2569965 -2.5633415

C 6.5898695 -1.4467685 -1.4610235

H 6.9756455 -2.4708415 -1.4826965

H 7.4215005 -0.7535315 -1.6222955

H 6.1713625 -1.2597265 -0.4690555

C 6.1427855 -1.5400935 -3.9319355

H 6.9855365 -0.8684805 -4.1143255

H 6.5378755 -2.5583795 -3.9702325

H 5.4184295 -1.4174165 -4.7421865

Pd 0.4298985 0.3485495 0.3396465

C -1.7415045 0.3641695 0.9684615

C -2.4118905 -0.3429365 -0.0941845

C -1.2588755 1.6802565 0.8402775

H -2.5356665 -1.4108405 0.0274265

H -1.0354755 2.2629965 1.7295875

H -1.4906775 2.2637285 -0.0429955

H -2.2163465 -0.0249745 -1.1130045

C -4.5588715 0.2723555 -0.1503485

C -4.3039155 1.6695725 -0.5634235

C -3.8385835 1.9745135 -1.8652395

C -4.3514435 2.7389895 0.3533095

C -3.4599005 3.2617585 -2.2251685

H -3.7493395 1.1766395 -2.5940705

C -3.9760245 4.0336635 -0.0095315

H -4.6811455 2.5652595 1.3694385

C -3.5292895 4.3088905 -1.2988725

H -3.1034685 3.4498675 -3.2345095

H -4.0292285 4.8283565 0.7292905

H -3.2404535 5.3168845 -1.5800505

C -5.1232455 -0.6929765 -1.1375145

C -4.9689145 -2.0813945 -0.9475145

C -5.8863955 -0.2805045 -2.2459195

C -5.5298265 -3.0037855 -1.8247765

H -4.3982965 -2.4449645 -0.1003185

C -6.4486125 -1.2042695 -3.1271375

H -6.0610935 0.7775455 -2.4039475

C -6.2719515 -2.5727915 -2.9271445

H -5.3883575 -4.0663625 -1.6451675

H -7.0370835 -0.8484295 -3.9683425

H -6.7089685 -3.2919035 -3.6132865

C -4.9699995 0.0377305 1.2944375

H -4.8463685 -1.0236005 1.5383715

H -4.2703805 0.5742235 1.9447235

C -6.4171495 0.4463655 1.6723695

H -6.6165455 1.4376835 1.2456765

C -7.4705425 -0.5220685 1.1188115

H -7.2898125 -1.5404245 1.4838425

H -8.4741545 -0.2247615 1.4430835

H -7.4633495 -0.5573295 0.0282715

C -6.5373145 0.5524335 3.1981025

H -6.3162475 -0.4122655 3.6715425

H -5.8360055 1.2892015 3.6058685

H -7.5487465 0.8456625 3.4992545

H -1.8069625 -0.0739225 1.9626655

#### Int-5 = [(product)Pd(Xantphos)]

| 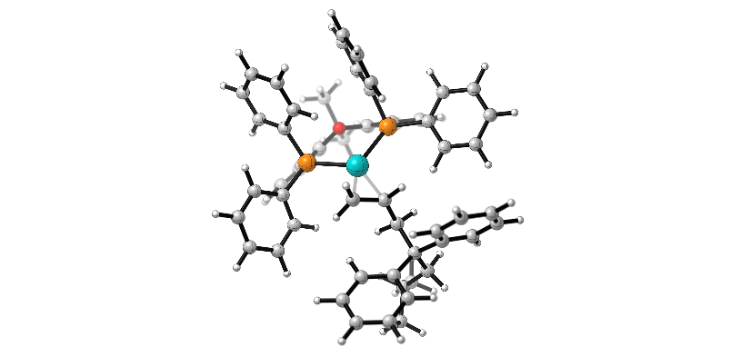 | E/hartree | -3168.316914 |
| --- | --- | --- |
|  | E+zvp/hartree | -3167.330727 |
|  | G/hartree | -3167.427583 |
|  | E(_SP)_/hartree | -3169.412695 |

C -2.6561567 -3.4541014 -1.2893252

C -2.6166707 -2.1846014 -0.6958092

C -3.2639857 -1.1439694 -1.3713532

C -3.9037577 -1.2979444 -2.6044242

C -3.9003017 -2.5738294 -3.1742022

C -3.2893417 -3.6437674 -2.5174562

C -3.7227857 1.1455936 -2.8742112

C -3.0939347 1.1724706 -1.6265692

C -2.2937107 2.2304176 -1.1786172

C -2.1430947 3.3248436 -2.0422502

C -2.7569577 3.3305866 -3.2941872

C -3.5356107 2.2491586 -3.7105232

H -2.1733557 -4.2889214 -0.7948222

H -4.3804127 -2.7410394 -4.1316622

H -3.3051737 -4.6314374 -2.9678292

H -1.5290387 4.1640026 -1.7377892

H -2.6249527 4.1832086 -3.9531292

H -3.9988077 2.2716796 -4.6903312

C -4.6009757 -0.0679984 -3.1930762

C -5.9603667 0.1117596 -2.4646382

H -6.6035637 -0.7552074 -2.6457872

H -5.8155067 0.2106646 -1.3860112

H -6.4685447 1.0100976 -2.8292052

C -4.8559497 -0.2146574 -4.6976832

H -5.3735607 0.6652736 -5.0880102

H -3.9228897 -0.3442574 -5.2534512

H -5.5014427 -1.0738274 -4.8969122

O -3.2455757 0.0980146 -0.7717422

P -1.7215597 -1.7852574 0.8696318

P -1.3619617 2.0275436 0.4015678

Pd -0.2523717 0.0390766 0.9149028

C -3.0919907 -1.7863814 2.0911048

C -2.9657617 -0.9594614 3.2144618

C -4.2509357 -2.5598344 1.9345718

C -3.9771367 -0.9130504 4.1731808

H -2.0833347 -0.3362444 3.3146208

C -5.2635557 -2.5088064 2.8917668

H -4.3608657 -3.1949974 1.0612078

C -5.1271067 -1.6865084 4.0128248

H -3.8741257 -0.2554344 5.0296878

H -6.1601737 -3.1081554 2.7619238

H -5.9197777 -1.6436194 4.7541768

C -0.8507327 -3.3780624 1.1765328

C -1.1701487 -4.2533584 2.2197678

C 0.2578053 -3.6707324 0.3618198

C -0.3952917 -5.3950244 2.4462428

H -2.0230227 -4.0439564 2.8565798

C 1.0181293 -4.8167514 0.5763238

H 0.5223413 -2.9860544 -0.4399972

C 0.6959663 -5.6815734 1.6273958

H -0.6510447 -6.0626704 3.2639818

H 1.8662153 -5.0301854 -0.0682312

H 1.2946153 -6.5697144 1.8058658

C -2.6420377 2.2707206 1.7056088

C -4.0191097 2.2032366 1.4720608

C -2.1853947 2.4698566 3.0195938

C -4.9220307 2.3253456 2.5303728

H -4.3929687 2.0589476 0.4655548

C -3.0871487 2.6076146 4.0711528

H -1.1172227 2.5209466 3.2111518

C -4.4616467 2.5303396 3.8297538

H -5.9884547 2.2633836 2.3343128

H -2.7181217 2.7704086 5.0798468

H -5.1665857 2.6286496 4.6498808

C -0.4687207 3.6375636 0.5090058

C 0.9133613 3.6522246 0.2847978

C -1.1205627 4.8413276 0.8201208

C 1.6360073 4.8436386 0.3660658

H 1.4234223 2.7226216 0.0645528

C -0.4006847 6.0320376 0.9013628

H -2.1903467 4.8406276 1.0041258

C 0.9788323 6.0343566 0.6755648

H 2.7089083 4.8285006 0.2059958

H -0.9140427 6.9576876 1.1456658

H 1.5387333 6.9623426 0.7482968

C 1.9762453 0.2911856 1.1383228

C 1.5472433 -0.8002474 1.8898418

C 2.5505763 0.1611896 -0.2572932

H 2.1672573 1.2300026 1.6482108

H 1.3840683 -0.6887844 2.9597538

H 2.1428893 -0.7363794 -0.7307112

H 2.1972033 0.9969466 -0.8705882

H 1.6706953 -1.8211504 1.5521358

C 4.1126963 0.1653926 -0.4105922

C 4.7959033 -1.0583724 0.2095598

C 6.1702663 -1.0164184 0.4918678

C 4.1338043 -2.2780164 0.3920908

C 6.8522773 -2.1432594 0.9463178

H 6.7110653 -0.0862744 0.3515868

C 4.8095743 -3.4095604 0.8512738

H 3.0799643 -2.3638624 0.1691148

C 6.1731163 -3.3491484 1.1318938

H 7.9164043 -2.0789734 1.1547308

H 4.2596803 -4.3357104 0.9922978

H 6.7011123 -4.2276794 1.4904908

C 4.6165393 1.4641686 0.2381298

C 4.8395583 2.6352946 -0.4969102

C 4.7854603 1.5290436 1.6318158

C 5.2232153 3.8236006 0.1317278

H 4.7219603 2.6363346 -1.5735652

C 5.1575733 2.7131166 2.2635578

H 4.6264083 0.6344316 2.2229998

C 5.3802943 3.8707096 1.5152748

H 5.4018653 4.7113996 -0.4687182

H 5.2772003 2.7299606 3.3429588

H 5.6762263 4.7937816 2.0046068

C 4.4192923 0.1558906 -1.9415792

H 5.4762453 0.4078306 -2.0881232

H 3.8418483 0.9692366 -2.3940122

C 4.1006803 -1.1503294 -2.7112482

H 3.3673073 -1.7309884 -2.1399112

C 5.3437943 -2.0311194 -2.8989992

H 5.0880213 -2.9587624 -3.4227422

H 6.0949433 -1.5057214 -3.5016822

H 5.8033313 -2.2961094 -1.9452112

C 3.4659583 -0.8258874 -4.0706162

H 4.1430403 -0.2167214 -4.6819292

H 3.2432713 -1.7391314 -4.6329282

H 2.5319843 -0.2662804 -3.9489512

#### Int-6 = [(allene)Pd^0^(Xantphos)]

| 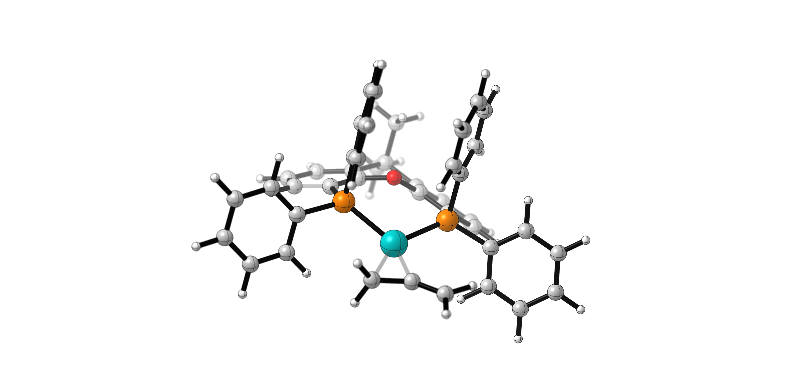 | E/hartree | -2508.308850 |
| --- | --- | --- |
|  | E+zvp/hartree | -2507.652497 |
|  | G/hartree | -2507.728233 |
|  | E(_SP)_/hartree | -2509.131324 |

C -3.4998491 1.6755863 -1.0628212

C -2.2830391 1.1470103 -0.6085782

C -1.2254681 2.0442793 -0.4349722

C -1.3017531 3.4089753 -0.7245302

C -2.5259821 3.8939023 -1.1912282

C -3.6165561 3.0355143 -1.3480422

C 1.1584269 3.3949303 -0.8543352

C 1.1032439 2.0351233 -0.5418172

C 2.1450079 1.1346713 -0.7790022

C 3.3163109 1.6482363 -1.3511142

C 3.4041049 3.0002443 -1.6850732

C 2.3352239 3.8658033 -1.4435612

H -4.3474411 1.0174343 -1.2118182

H -2.6359391 4.9447743 -1.4338112

H -4.5631031 3.4297243 -1.7045982

H 4.1516719 0.9859633 -1.5454092

H 4.3140759 3.3835803 -2.1361132

H 2.4261769 4.9123223 -1.7116392

C -0.0498851 4.2529313 -0.4664832

C 0.0359299 4.5442763 1.0562568

H -0.8379321 5.1174963 1.3817048

H 0.0726859 3.6150403 1.6303838

H 0.9388359 5.1213663 1.2796388

C -0.0837501 5.5814463 -1.2303332

H 0.8112219 6.1711733 -1.0162202

H -0.1455631 5.4230093 -2.3107472

H -0.9406571 6.1837143 -0.9178272

O -0.0391861 1.5167783 0.0334808

P -1.9402471 -0.6557147 -0.4006552

P 1.8568999 -0.6436487 -0.3776602

Pd -0.0187011 -1.8336247 -1.1552542

C -1.9808621 -0.9069897 1.4211798

C -2.0774691 -2.2280087 1.8896628

C -1.8517441 0.1335213 2.3464398

C -2.0709851 -2.4961207 3.2555588

H -2.1628861 -3.0430667 1.1772608

C -1.8239671 -0.1400677 3.7150038

H -1.7755901 1.1587023 2.0039408

C -1.9406591 -1.4510527 4.1736008

H -2.1581321 -3.5213527 3.6036268

H -1.7133351 0.6764323 4.4222688

H -1.9237151 -1.6598027 5.2391078

C -3.5547451 -1.3656167 -0.9227992

C -4.6698191 -1.4337017 -0.0755982

C -3.6719351 -1.8214467 -2.2425902

C -5.8780641 -1.9518657 -0.5418532

H -4.5896471 -1.0829497 0.9479918

C -4.8831091 -2.3298767 -2.7114682

H -2.8022991 -1.7851787 -2.8911572

C -5.9873601 -2.3990087 -1.8603672

H -6.7347811 -2.0049207 0.1236788

H -4.9614351 -2.6811097 -3.7360772

H -6.9283481 -2.8038417 -2.2209732

C 1.8850149 -0.6324087 1.4592958

C 2.3725649 0.4428373 2.2128528

C 1.3826929 -1.7618307 2.1213038

C 2.3578949 0.3884893 3.6071378

H 2.7616999 1.3223073 1.7104158

C 1.3814449 -1.8185457 3.5130728

H 0.9777769 -2.5820877 1.5366248

C 1.8650559 -0.7424357 4.2589538

H 2.7330069 1.2297483 4.1830098

H 0.9789779 -2.6926087 4.0139158

H 1.8506259 -0.7821807 5.3442078

C 3.4980169 -1.3412667 -0.8229602

C 3.6857709 -1.7616157 -2.1477412

C 4.5591839 -1.4457187 0.0849728

C 4.9194679 -2.2607807 -2.5621292

H 2.8511849 -1.7164087 -2.8411302

C 5.7887929 -1.9615347 -0.3274062

H 4.4231589 -1.1248697 1.1122798

C 5.9727689 -2.3648207 -1.6509572

H 5.0522819 -2.5856567 -3.5897162

H 6.6036789 -2.0460707 0.3857128

H 6.9304399 -2.7657997 -1.9695182

C -0.7741461 -3.7039457 -1.8981862

H -1.4734381 -4.1589627 -1.1982542

H -1.1536141 -3.5864087 -2.9120662

C 0.5996999 -3.7481557 -1.6673852

C 1.7144749 -4.4630697 -1.6128222

H 2.6611499 -4.0348647 -1.3024972

H 1.7204989 -5.5163327 -1.8952442

#### TS-4 = TS allene protonation

| 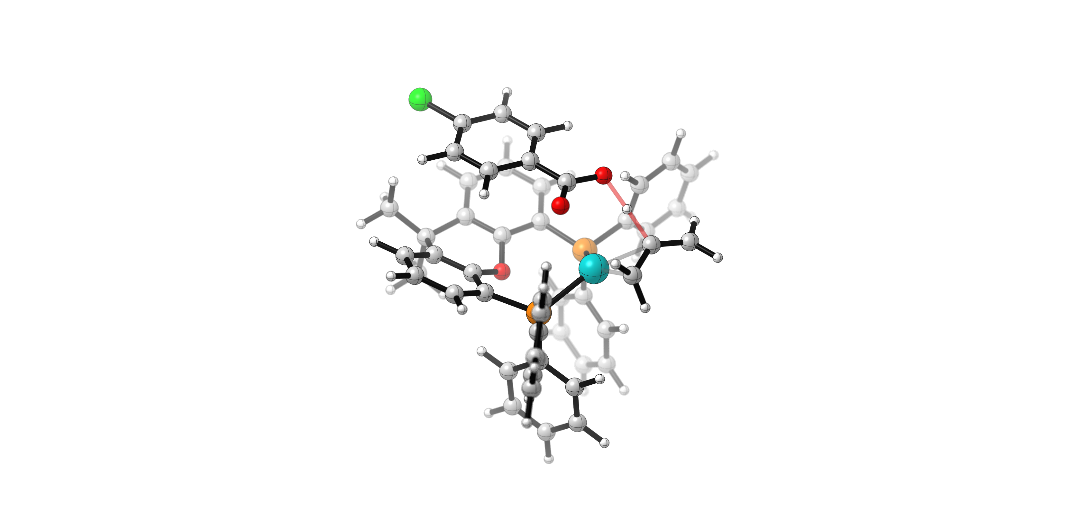 | E/hartree | -3388.768919 |
| --- | --- | --- |
|  | E+zvp/hartree | -3388.009367 |
|  | G/hartree | -3388.094591 |
|  | E(_SP)_/hartree | -3389.826162 |
|  | Imaginary frequency/cm^-1^ | -1413.19 |

Pd -1.1336345 0.6460384 -1.5755427

H -0.1309405 1.0841714 -3.5839077

C -2.3235085 0.3843604 -3.3181297

H -2.0954905 -0.5635666 -3.7903397

H -3.3742495 0.5572354 -3.0839997

C -1.4373695 1.4822204 -3.5376437

C -1.6371475 2.7683814 -3.8924867

H -0.8586125 3.5122994 -3.7434127

H -2.5399905 3.1035664 -4.4054127

O 0.2704715 -1.4328326 -3.4594687

C 1.1601575 -0.5917656 -3.2748947

O 1.1069515 0.6570704 -3.6434277

C 2.4085125 -0.9900276 -2.5315907

C 2.4792855 -2.2608556 -1.9511607

C 3.4903975 -0.1136446 -2.4012267

C 3.6188085 -2.6628726 -1.2589677

H 1.6264095 -2.9225446 -2.0488147

C 4.6405095 -0.5027196 -1.7208307

H 3.4213865 0.8736004 -2.8419957

C 4.6910435 -1.7774626 -1.1615777

H 3.6756865 -3.6449546 -0.8046827

H 5.4829835 0.1703384 -1.6156487

Cl 6.1471555 -2.2716066 -0.3000547

P -1.8891105 -1.1726026 -0.3466867

C -0.6417005 -2.3281166 0.3728843

C -2.9466195 -2.3566446 -1.2694757

C -2.9344895 -0.6529786 1.0658683

C -0.6705575 -3.7158716 0.1674873

C 0.4364635 -1.8174206 1.0991613

C -4.1649705 -2.8421286 -0.7789547

C -2.4591755 -2.8271196 -2.5009077

C -2.5767645 -0.8532266 2.4024393

C -4.1209115 0.0396144 0.7711403

C 0.3556685 -4.5235756 0.6548683

H -1.4844685 -4.1588746 -0.3918237

C 1.4714925 -2.5996496 1.6205653

O 0.4647245 -0.4539056 1.2951043

C -4.8998605 -3.7701376 -1.5195807

H -4.5411265 -2.5020036 0.1790263

C -3.1926965 -3.7630326 -3.2277887

H -1.5048125 -2.4687306 -2.8761057

C -3.3929805 -0.3720536 3.4277163

H -1.6654825 -1.3861246 2.6467923

C -4.9475915 0.4914814 1.7954053

H -4.3956885 0.2139734 -0.2652887

C 1.4190875 -3.9721376 1.3737603

H 0.3248715 -5.5935936 0.4757503

C 2.5390265 -1.8852656 2.4562343

C 1.7193345 0.1215184 1.2291823

C -4.4170945 -4.2309766 -2.7442237

H -5.8467875 -4.1358836 -1.1333657

H -2.8072715 -4.1218946 -4.1774227

C -4.5814985 0.2917654 3.1289873

H -3.0995025 -0.5242036 4.4619533

H -5.8703805 1.0109364 1.5541133

H 2.2059385 -4.6189806 1.7447923

C 2.8164395 -0.5442306 1.7724803

C 3.8056335 -2.7285416 2.6275733

C 1.9349045 -1.5924276 3.8574093

C 1.7908055 1.3607374 0.5945743

H -4.9899615 -4.9534206 -3.3181317

H -5.2176745 0.6574704 3.9292343

C 4.0531485 0.1006424 1.6876553

H 3.5769465 -3.6612496 3.1501873

H 4.2695235 -2.9659236 1.6689123

H 4.5391035 -2.1958106 3.2381803

H 2.6615685 -1.0542246 4.4739543

H 1.0332905 -0.9798476 3.7778803

H 1.6742285 -2.5295856 4.3592123

C 3.0398315 1.9904674 0.5452313

P 0.2331975 1.9895244 -0.1422897

C 4.1586215 1.3596984 1.0907843

H 4.9425725 -0.3804326 2.0770283

H 3.1345775 2.9572124 0.0641023

C 0.7950515 3.5645884 -0.9011737

C -0.7110895 2.5295484 1.3340613

H 5.1275145 1.8462264 1.0389703

C 0.6324095 4.8060574 -0.2741107

C 1.4167345 3.5006654 -2.1591587

C -0.1125175 2.7371974 2.5832843

C -2.0887245 2.7409554 1.1896023

C 1.0798875 5.9715054 -0.8993477

H 0.1567995 4.8634314 0.6988603

C 1.8724635 4.6672974 -2.7719597

H 1.5288395 2.5422764 -2.6585217

C -0.8824755 3.1498264 3.6708783

H 0.9533485 2.5749054 2.7043533

C -2.8530235 3.1665524 2.2737353

H -2.5562495 2.5531364 0.2276183

C 1.7013965 5.9050094 -2.1466007

H 0.9438815 6.9307914 -0.4085287

H 2.3520615 4.6085024 -3.7446297

C -2.2524395 3.3670844 3.5173343

H -0.4112255 3.3026514 4.6374303

H -3.9204205 3.3166404 2.1529763

H 2.0485075 6.8130064 -2.6308257

H -2.8513675 3.6854984 4.3653843

#### TS-5

| 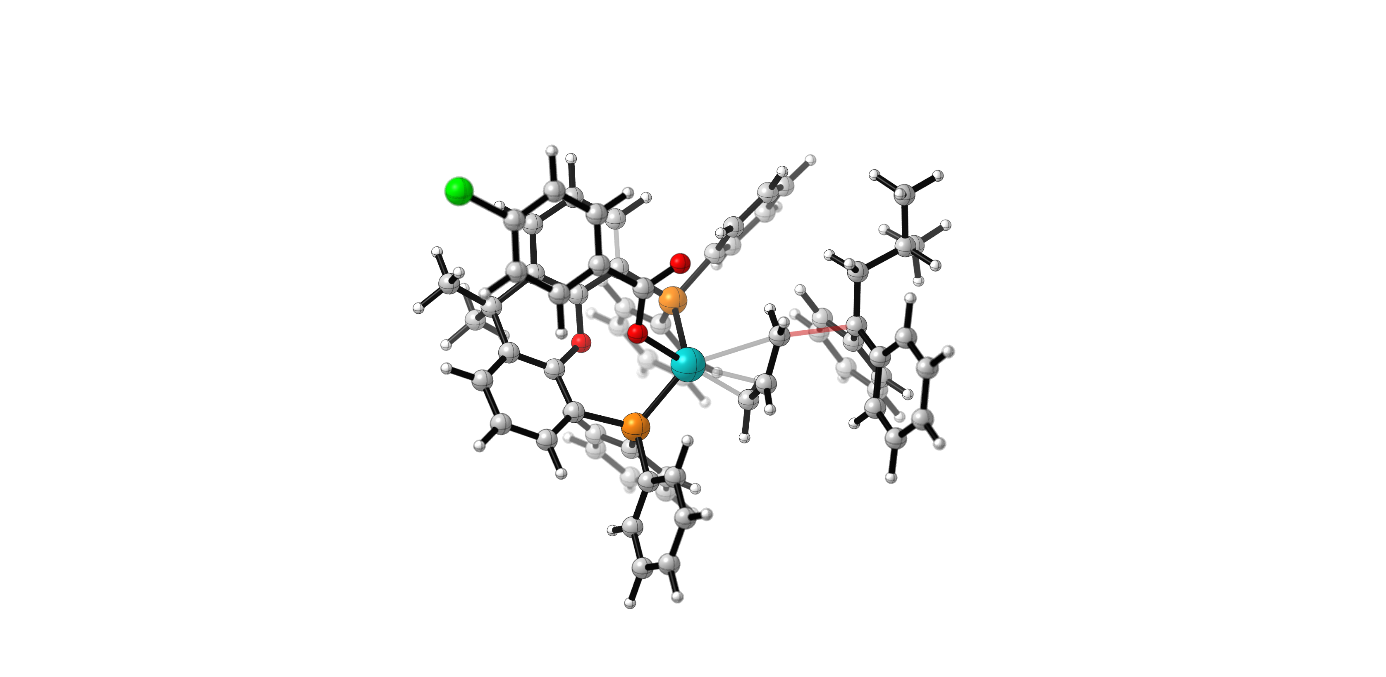 | E/hartree | -4048.148121 |
| --- | --- | --- |
|  | E+zvp/hartree | -4047.069112 |
|  | G/hartree | -4047.175694 |
|  | E(_SP)_/hartree | -4049.479243 |
|  | Imaginary frequency/cm^-1^ | -491.82 |

C -2.3238806 -2.8414084 1.8060272

C -2.1259096 -1.4856924 1.5148132

C -3.2485806 -0.7414004 1.1385712

C -4.5368196 -1.2744424 1.0520812

C -4.6896986 -2.6350374 1.3231432

C -3.5911076 -3.4101984 1.6979972

C -5.1504706 0.6930306 -0.2958498

C -3.8291056 1.1270956 -0.1556768

C -3.2436266 2.0889626 -0.9880758

C -4.0302936 2.6187806 -2.0194558

C -5.3450886 2.1904936 -2.1960428

C -5.9001046 1.2365546 -1.3411698

H -1.4778116 -3.4538744 2.0930602

H -5.6656146 -3.0978594 1.2385292

H -3.7254466 -4.4668244 1.9064362

H -3.6055536 3.3517326 -2.6948648

H -5.9413066 2.6034596 -3.0035468

H -6.9236616 0.9144936 -1.4941088

C -5.6765856 -0.3037784 0.7390302

C -6.0013886 0.4830226 2.0389112

H -6.3532526 -0.2038294 2.8150652

H -5.1158176 1.0003546 2.4171502

H -6.7812066 1.2268466 1.8468402

C -6.9434296 -1.0258334 0.2679812

H -7.7453776 -0.3090204 0.0726872

H -6.7609616 -1.6080854 -0.6373028

H -7.3082736 -1.7007934 1.0463002

O -3.0443566 0.5962576 0.8462132

P -0.4886486 -0.6551924 1.4736852

P -1.4582076 2.4665506 -0.8068318

Pd -0.1608396 0.5507336 -0.7616268

C -0.5860506 0.4773596 2.9193032

C 0.3719664 1.4966506 3.0237432

C -1.5519696 0.3497136 3.9259882

C 0.3803694 2.3522156 4.1228232

H 1.1081104 1.6236156 2.2398952

C -1.5553036 1.2203336 5.0164542

H -2.2984306 -0.4345224 3.8577352

C -0.5852766 2.2177636 5.1214852

H 1.1252424 3.1388506 4.1847832

H -2.3118816 1.1126066 5.7885152

H -0.5872596 2.8928746 5.9721862

C 0.6468864 -1.9685124 2.0863942

C 1.2610534 -1.9204644 3.3460612

C 0.9762484 -3.0131504 1.2023702

C 2.2037784 -2.8841534 3.7094332

H 1.0090034 -1.1266114 4.0402782

C 1.9000104 -3.9863844 1.5838642

H 0.4994074 -3.0491794 0.2264512

C 2.5274354 -3.9187174 2.8306402

H 2.6820294 -2.8272214 4.6829342

H 2.1401264 -4.7924824 0.8966762

H 3.2604834 -4.6675974 3.1151512

C -1.3364946 3.5690656 0.6517722

C -2.2861336 3.5496986 1.6821102

C -0.2148886 4.4053336 0.7790272

C -2.1219776 4.3562026 2.8067932

H -3.1517756 2.9047336 1.6115452

C -0.0579836 5.2150006 1.9015032

H 0.5250514 4.4419436 -0.0133458

C -1.0125236 5.1932196 2.9197072

H -2.8622096 4.3227156 3.5998642

H 0.8091974 5.8643486 1.9784882

H -0.8887836 5.8203126 3.7973932

C -1.1146736 3.5824586 -2.2186858

C -0.5450036 3.0149386 -3.3673828

C -1.4292056 4.9493066 -2.2096888

C -0.2931886 3.8023976 -4.4910028

H -0.3057336 1.9550516 -3.3648178

C -1.1703526 5.7352196 -3.3320038

H -1.8711046 5.3952976 -1.3248328

C -0.6022576 5.1633926 -4.4731048

H 0.1478024 3.3541986 -5.3762338

H -1.4133956 6.7935486 -3.3169628

H -0.4002716 5.7785816 -5.3448688

H 1.9935604 1.1454456 -2.5808848

C 1.6929374 1.5489456 -0.4650358

H 1.6722874 2.6216066 -0.6321438

H 2.0905464 1.2563906 0.4996652

C 1.9594924 0.7106806 -1.5865618

C 2.2041604 -0.6672924 -1.4490908

H 2.1443204 -1.3033744 -2.3242528

H 2.0045154 -1.1494844 -0.5030148

O -1.7737366 -0.5213004 -1.6788008

C -1.7333696 -1.8072724 -1.6446948

O -0.7535076 -2.5057894 -1.3318128

C -3.0468306 -2.4677084 -1.9794438

C -4.1116756 -1.7228584 -2.4971738

C -3.2240106 -3.8278684 -1.7144478

C -5.3432156 -2.3243654 -2.7432358

H -3.9662686 -0.6671764 -2.6895838

C -4.4547456 -4.4418474 -1.9363418

H -2.3853806 -4.3850374 -1.3131778

C -5.5036216 -3.6775564 -2.4450368

H -6.1715726 -1.7537964 -3.1480788

H -4.6060866 -5.4924024 -1.7150648

Cl -7.0748636 -4.4323304 -2.7041958

C 4.3942074 -1.0052884 -1.0772318

C 4.9176454 -0.5185224 -2.3673708

C 4.8933874 0.8561146 -2.7018468

C 5.3798604 -1.4065344 -3.3622238

C 5.3284884 1.3125466 -3.9399928

H 4.4969614 1.5658796 -1.9865508

C 5.8196664 -0.9471444 -4.6018878

H 5.4045244 -2.4713094 -3.1686418

C 5.8031424 0.4148056 -4.9005968

H 5.2884144 2.3754816 -4.1608988

H 6.1769884 -1.6617424 -5.3376828

H 6.1451514 0.7716816 -5.8671378

C 4.5827374 -0.2019494 0.1551342

C 5.5352844 0.8316886 0.2510082

C 3.8244254 -0.4822864 1.3136282

C 5.6916744 1.5697066 1.4239142

H 6.1824554 1.0398866 -0.5917728

C 3.9744134 0.2577506 2.4801182

H 3.0945614 -1.2800124 1.3051882

C 4.9043454 1.2990286 2.5425662

H 6.4415174 2.3547476 1.4618672

H 3.3573954 0.0172436 3.3394592

H 5.0223414 1.8783026 3.4533052

C 4.2876504 -2.5072904 -0.8821578

H 3.8628334 -2.9533214 -1.7872498

H 3.5738774 -2.7263934 -0.0850848

C 5.6056134 -3.2513784 -0.5353048

H 6.3773704 -2.9493654 -1.2545808

C 6.1232454 -2.9315984 0.8729782

H 5.3578594 -3.1499784 1.6265952

H 7.0046904 -3.5413374 1.1001102

H 6.4023294 -1.8822594 0.9814572

C 5.3734454 -4.7607594 -0.6863448

H 4.5967094 -5.1012744 0.0089602

H 5.0484104 -5.0203594 -1.6997678

H 6.2857944 -5.3254234 -0.4679638

#### Int-7

| 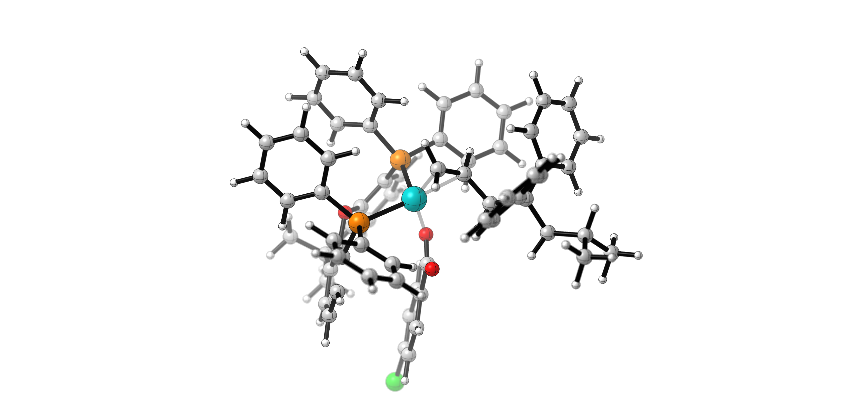 | E/hartree | -4048.18850 |
| --- | --- | --- |
|  | E+zvp/hartree | -4047.10677 |
|  | G/hartree | -4047.21698 |
|  | E(_SP)_/hartree | -4049.518032 |

C -3.5860887 -1.5097318 2.4881918

C -3.1567387 -0.4188058 1.7248848

C -3.8409947 -0.1498118 0.5368628

C -4.9249967 -0.8964788 0.0786898

C -5.3122407 -1.9948678 0.8514128

C -4.6492697 -2.2957698 2.0436878

C -4.5236057 0.0920832 -2.1426332

C -3.4355537 0.7643572 -1.5771312

C -2.3712057 1.2768062 -2.3268212

C -2.4388827 1.1451472 -3.7207292

C -3.5167467 0.4928892 -4.3158982

C -4.5420617 -0.0421438 -3.5325702

H -3.0654457 -1.7551238 3.4073248

H -6.1282787 -2.6277418 0.5231798

H -4.9623587 -3.1563348 2.6258798

H -1.6292897 1.5241132 -4.3331282

H -3.5533147 0.3894082 -5.3956912

H -5.3612767 -0.5649618 -4.0128832

C -5.6193227 -0.4039268 -1.1932442

C -6.5101067 0.8100642 -0.8115922

H -7.2831257 0.5005732 -0.1012472

H -5.9163407 1.6024552 -0.3487912

H -6.9954177 1.2181262 -1.7039462

C -6.4986697 -1.4861468 -1.8278992

H -6.9991587 -1.1037838 -2.7213312

H -5.9150767 -2.3683758 -2.0983102

H -7.2845427 -1.7929308 -1.1330052

O -3.3762377 0.9198282 -0.2103942

P -1.6573267 0.5771192 2.0354218

P -0.8577857 1.8711582 -1.4774222

Pd -0.0707767 0.4372462 0.2260838

C -2.3124657 2.2360692 2.4476808

C -1.4098507 3.3085832 2.4492518

C -3.6572337 2.4720802 2.7581648

C -1.8408067 4.5914182 2.7773028

H -0.3742317 3.1302022 2.1782228

C -4.0910657 3.7619402 3.0671288

H -4.3624607 1.6474342 2.7581448

C -3.1836807 4.8217842 3.0821178

H -1.1343997 5.4144102 2.7697548

H -5.1371947 3.9367432 3.3014378

H -3.5221667 5.8249252 3.3240758

C -1.0100037 -0.0370998 3.6402368

C -1.2656327 0.6119412 4.8558938

C -0.1653107 -1.1609788 3.6227028

C -0.6809177 0.1495892 6.0359538

H -1.9145367 1.4808012 4.8793678

C 0.4082203 -1.6221828 4.8073028

H 0.0187343 -1.6701718 2.6821548

C 0.1582143 -0.9653118 6.0145788

H -0.8821077 0.6622242 6.9721488

H 1.0552483 -2.4946318 4.7843578

H 0.6140903 -1.3208838 6.9340418

C -1.1612117 3.6072752 -1.0204612

C -2.4469687 4.1615492 -1.0185272

C -0.0797497 4.3810412 -0.5653072

C -2.6442597 5.4734332 -0.5885612

H -3.2902987 3.5684502 -1.3538022

C -0.2776477 5.6981632 -0.1604332

H 0.9172093 3.9514702 -0.5419502

C -1.5621777 6.2475492 -0.1705272

H -3.6462627 5.8911482 -0.5841102

H 0.5680493 6.2927122 0.1723948

H -1.7186477 7.2712022 0.1557528

C 0.3862973 1.9026232 -2.8203942

C 0.9103473 0.6541552 -3.2070632

C 0.9063213 3.0717732 -3.3857822

C 1.9603413 0.5887082 -4.1169122

H 0.5060953 -0.2495268 -2.7596092

C 1.9615873 2.9978462 -4.2993552

H 0.5020283 4.0383812 -3.1068552

C 2.4978763 1.7617632 -4.6551482

H 2.3842173 -0.3741428 -4.3824412

H 2.3678923 3.9112122 -4.7238142

H 3.3379633 1.7078382 -5.3395222

H 2.2869143 1.1705852 -1.0656152

C 1.8494983 1.2119732 1.0109168

H 1.8377513 2.2979362 1.0428908

H 1.9648613 0.7217712 1.9715058

C 2.1191453 0.5568672 -0.1881862

C 2.5344503 -0.8886378 -0.3228542

H 2.2420113 -1.2340828 -1.3191002

H 1.9732343 -1.5120158 0.3762218

O -1.0389657 -1.1633188 -1.1225212

C -1.0891947 -2.1531528 -0.3162282

O -0.3896647 -2.2610268 0.7209788

C -2.1171827 -3.2064388 -0.6274822

C -2.8572617 -3.1424888 -1.8130792

C -2.3814537 -4.2220148 0.2950218

C -3.8491917 -4.0828588 -2.0792242

H -2.6527087 -2.3400608 -2.5120542

C -3.3826067 -5.1599178 0.0528278

H -1.8003317 -4.2496618 1.2096108

C -4.1091607 -5.0749888 -1.1336022

H -4.4185387 -4.0501108 -3.0014792

H -3.6041917 -5.9419408 0.7703038

Cl -5.3932847 -6.2395688 -1.4434982

C 4.0668873 -1.1915758 -0.1759502

C 4.7683923 -0.3873708 -1.2787442

C 4.9823143 0.9933022 -1.1226392

C 5.0897363 -0.9508178 -2.5207302

C 5.4920173 1.7726512 -2.1587092

H 4.7432003 1.4605172 -0.1746732

C 5.6076113 -0.1756648 -3.5607542

H 4.9357963 -2.0082018 -2.6934752

C 5.8100233 1.1915132 -3.3871152

H 5.6403693 2.8375872 -2.0043742

H 5.8529223 -0.6485988 -4.5075852

H 6.2129393 1.7961972 -4.1942102

C 4.5673573 -0.8523248 1.2341278

C 5.8642033 -0.3782668 1.4697728

C 3.7655023 -1.1328168 2.3521838

C 6.3362903 -0.1742988 2.7677828

H 6.5179723 -0.1714398 0.6305238

C 4.2276613 -0.9232508 3.6497018

H 2.7640253 -1.5251198 2.2185968

C 5.5190763 -0.4395948 3.8656358

H 7.3481183 0.1917422 2.9169258

H 3.5708263 -1.1349538 4.4886128

H 5.8832803 -0.2749208 4.8752948

C 4.2095263 -2.7302228 -0.3704092

H 3.7654803 -2.9898758 -1.3378022

H 3.5676963 -3.2106828 0.3765408

C 5.6358503 -3.3230008 -0.2690032

H 6.3572263 -2.5315188 -0.5088722

C 5.9493403 -3.8456008 1.1412378

H 5.2713293 -4.6683528 1.3993888

H 6.9734733 -4.2315218 1.1927428

H 5.8413393 -3.0711258 1.9016288

C 5.8232323 -4.4528878 -1.2913292

H 5.0963563 -5.2556318 -1.1184052

H 5.6866373 -4.0980958 -2.3186392

H 6.8246523 -4.8905708 -1.2191802

#### Int-8

| 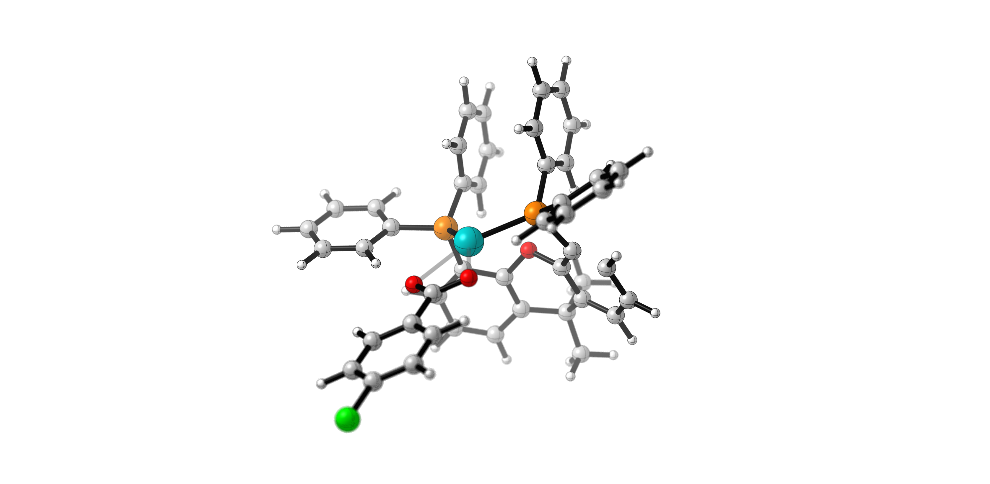 | E/hartree | -3271.475184 |
| --- | --- | --- |
|  | E+zvp/hartree | -3270.781121 |
|  | G/hartree | -3270.867943 |
|  | E(_SP)_/hartree | -3272.489063 |

C 0.4694557 3.3565022 1.8560070

C 0.7230297 2.1648892 1.1625350

C 1.1869077 1.0786822 1.9105780

C 1.3711887 1.1008382 3.2943890

C 1.0951837 2.3030242 3.9513620

C 0.6600777 3.4209902 3.2359420

C 1.2424997 -1.3640968 3.2338990

C 1.0769827 -1.2530588 1.8541360

C 0.5335497 -2.2546768 1.0461200

C 0.1601657 -3.4529678 1.6657900

C 0.3135397 -3.6015648 3.0450940

C 0.8413767 -2.5676648 3.8230060

H 0.0997447 4.2213582 1.3186680

H 1.2163547 2.3736002 5.0262640

H 0.4541707 4.3476762 3.7621930

H -0.2661463 -4.2529028 1.0711210

H 0.0136867 -4.5298518 3.5209670

H 0.9402367 -2.7060518 4.8936680

C 1.8902337 -0.1797108 3.9598420

C 1.6012617 -0.2017088 5.4651140

H 2.0942027 0.6360622 5.9648440

H 0.5282807 -0.1462648 5.6690540

H 1.9946397 -1.1150628 5.9185490

C 3.4242797 -0.2572708 3.7345900

H 3.8243327 -1.1773808 4.1720880

H 3.6633767 -0.2531618 2.6679910

H 3.9196177 0.5993682 4.2022910

O 1.4420117 -0.0853438 1.2139540

P 0.2707137 1.8893422 -0.6027630

P 0.2267547 -1.8372838 -0.7146980

Pd -1.2073263 0.0460712 -0.7598020

C -0.3652453 3.5433182 -1.0974740

C 0.5109647 4.6239912 -1.2974490

C -1.7448543 3.7381342 -1.2436570

C 0.0132257 5.8790522 -1.6383500

H 1.5807417 4.4795012 -1.1886890

C -2.2363993 5.0001312 -1.5883120

H -2.4276453 2.9118492 -1.0824210

C -1.3637323 6.0687272 -1.7866300

H 0.6982127 6.7080502 -1.7905330

H -3.3068033 5.1422102 -1.7040750

H -1.7507523 7.0469082 -2.0569540

C 1.8642527 1.8609482 -1.5150300

C 3.0987057 1.6142692 -0.9058660

C 1.8216537 2.1082662 -2.8969080

C 4.2710847 1.6314682 -1.6623150

H 3.1508657 1.4155132 0.1572010

C 2.9945087 2.1403712 -3.6455410

H 0.8682717 2.3014142 -3.3799070

C 4.2247987 1.9038802 -3.0285250

H 5.2225097 1.4329572 -1.1785890

H 2.9482907 2.3489422 -4.7103920

H 5.1404387 1.9238672 -3.6117810

C -0.3792453 -3.4339938 -1.3963550

C 0.5090927 -4.4462938 -1.7924170

C -1.7619783 -3.6533178 -1.4861130

C 0.0208497 -5.6617078 -2.2684870

H 1.5795137 -4.2833468 -1.7300890

C -2.2421103 -4.8755678 -1.9606080

H -2.4533143 -2.8733258 -1.1876860

C -1.3562533 -5.8790148 -2.3530340

H 0.7157957 -6.4382898 -2.5745540

H -3.3137953 -5.0377598 -2.0290970

H -1.7349653 -6.8259398 -2.7267000

C 1.9097407 -1.7415038 -1.4317200

C 3.0562407 -2.1613668 -0.7468650

C 2.0265527 -1.2892148 -2.7526500

C 4.2997257 -2.1398758 -1.3793870

H 2.9757677 -2.5161438 0.2749970

C 3.2660367 -1.2820158 -3.3862920

H 1.1435397 -0.9378138 -3.2780170

C 4.4053587 -1.7077538 -2.7010560

H 5.1838707 -2.4673948 -0.8401370

H 3.3462797 -0.9241068 -4.4072410

H 5.3730227 -1.6923058 -3.1934840

O -3.2505323 -1.0747678 -0.5908420

C -3.8650983 0.0286022 -0.4636880

O -3.2556773 1.1418862 -0.4841950

C -5.3499533 0.0167972 -0.2809780

C -6.0479453 -1.1955898 -0.2592890

C -6.0501773 1.2186302 -0.1296020

C -7.4297783 -1.2144548 -0.0902610

H -5.4906773 -2.1180778 -0.3758330

C -7.4319753 1.2167482 0.0406330

H -5.4942033 2.1490992 -0.1461090

C -8.1067993 -0.0039148 0.0578400

H -7.9792313 -2.1487598 -0.0722940

H -7.9830603 2.1427182 0.1591080

Cl -9.8520953 -0.0170098 0.2719090

#### Int-9

| 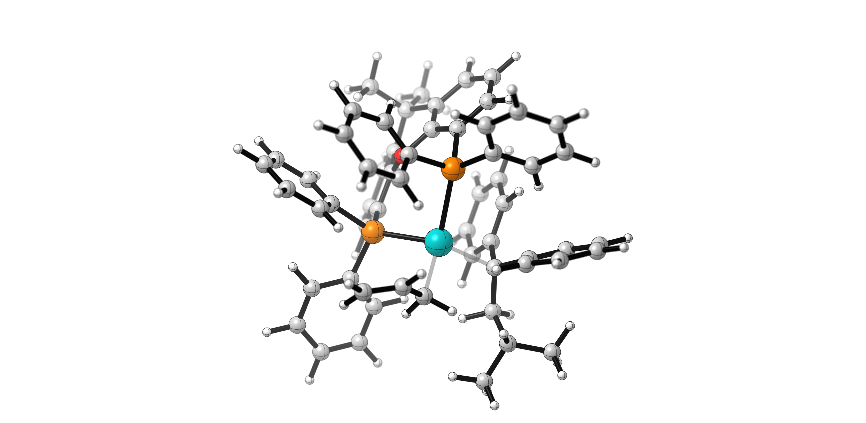 | E/hartree | -3168.273646 |
| --- | --- | --- |
|  | E+zvp/hartree | -3167.290393 |
|  | G/hartree | -3167.383070 |
|  | E(_SP)_/hartree | -3169.360824 |

C -3.1932862 1.4860397 2.1388224

C -2.5416182 0.6690337 1.2091634

C -2.4752102 -0.6966863 1.4918684

C -3.0455592 -1.2908123 2.6182234

C -3.6715932 -0.4410073 3.5340944

C -3.7351972 0.9342287 3.3005714

C -1.7088282 -3.3065193 2.0938504

C -1.1836952 -2.5944663 1.0141944

C 0.0072148 -2.9221213 0.3601214

C 0.6810558 -4.0678763 0.8044944

C 0.1808888 -4.8123913 1.8732214

C -0.9979882 -4.4322693 2.5182634

H -3.2751462 2.5503477 1.9540354

H -4.1214112 -0.8490043 4.4320904

H -4.2309812 1.5786167 4.0199454

H 1.6082038 -4.3632483 0.3289784

H 0.7163848 -5.6950913 2.2087534

H -1.3594402 -5.0183523 3.3554064

C -3.0293852 -2.8201633 2.6961854

C -4.1884092 -3.3469613 1.8067174

H -5.1510212 -2.9964903 2.1926864

H -4.0819832 -2.9908543 0.7784504

H -4.1900942 -4.4415713 1.7944844

C -3.2244062 -3.3251253 4.1306824

H -3.2488232 -4.4173553 4.1565994

H -2.4220062 -2.9802373 4.7890304

H -4.1788852 -2.9798033 4.5353724

O -1.8324362 -1.4715393 0.5519524

P -1.8130432 1.2159587 -0.3942366

P 0.7030518 -1.7032253 -0.8447206

Pd 0.6018568 0.9171977 -0.2973866

C -3.0647882 0.5378557 -1.5697466

C -2.7760522 0.5934767 -2.9385206

C -4.2979892 0.0145217 -1.1552816

C -3.7105872 0.1562237 -3.8754546

H -1.8194822 0.9835197 -3.2656276

C -5.2212722 -0.4443173 -2.0942946

H -4.5441882 -0.0285263 -0.1007306

C -4.9334412 -0.3685143 -3.4577116

H -3.4713712 0.2085957 -4.9326716

H -6.1711502 -0.8508823 -1.7587756

H -5.6563212 -0.7191983 -4.1885836

C -2.2305082 3.0074797 -0.3802776

C -3.1840902 3.5821427 -1.2279176

C -1.5114862 3.8384627 0.4945054

C -3.4077142 4.9610887 -1.2058466

H -3.7489462 2.9557867 -1.9086686

C -1.7474542 5.2103097 0.5283334

H -0.7582252 3.3983537 1.1399894

C -2.6937732 5.7774457 -0.3294066

H -4.1450632 5.3947807 -1.8750236

H -1.1827722 5.8377787 1.2115964

H -2.8691842 6.8489147 -0.3156826

C -0.3553772 -1.9123553 -2.3354836

C -1.4586502 -2.7694423 -2.4016136

C 0.0363728 -1.2173663 -3.4898176

C -2.1450792 -2.9425993 -3.6044176

H -1.7721662 -3.3172463 -1.5204316

C -0.6336752 -1.4092543 -4.6949006

H 0.8780358 -0.5345993 -3.4416076

C -1.7259332 -2.2773093 -4.7558046

H -3.0048212 -3.6047473 -3.6409916

H -0.3095482 -0.8742263 -5.5828176

H -2.2549862 -2.4239023 -5.6926546

C 2.1901038 -2.6639763 -1.3932136

C 3.3869398 -2.5372133 -0.6752146

C 2.1360378 -3.5844613 -2.4527766

C 4.4996168 -3.3132383 -1.0016866

H 3.4635528 -1.8262083 0.1358594

C 3.2521438 -4.3503913 -2.7856926

H 1.2178348 -3.7092403 -3.0142866

C 4.4375098 -4.2191323 -2.0593936

H 5.4154598 -3.1886633 -0.4339076

H 3.1922418 -5.0556503 -3.6097036

H 5.3059088 -4.8177213 -2.3187296

C 0.7723228 2.3168287 -2.8344386

C -0.2472092 2.7602127 -3.5927136

C 1.0138708 2.6529707 -1.4276746

H 1.5023368 1.6504937 -3.2978236

H -0.3549442 2.4666427 -4.6329066

H 2.0576658 2.8847347 -1.2543326

H 0.3823888 3.4650967 -1.0688396

H -0.9973372 3.4349307 -3.1889696

C 2.2960738 1.3799627 0.9964154

C 3.5846568 0.7059117 0.6059264

C 4.4502348 0.1725317 1.5769024

C 4.0204968 0.6922097 -0.7282536

C 5.6969188 -0.3502823 1.2285944

H 4.1433438 0.1782387 2.6177704

C 5.2739828 0.1957067 -1.0781926

H 3.3460988 1.0556647 -1.4949176

C 6.1214308 -0.3284673 -0.1007996

H 6.3425478 -0.7594223 2.0008634

H 5.5799948 0.1959647 -2.1201956

H 7.0944558 -0.7260953 -0.3735186

C 1.5110878 0.7266297 2.0875534

C 0.6227458 1.4638477 2.9104674

C 1.5527728 -0.6704353 2.3082914

C -0.1683902 0.8450797 3.8720864

H 0.5489798 2.5370457 2.7909194

C 0.7732818 -1.2879153 3.2813154

H 2.2174988 -1.2787313 1.7110554

C -0.0993562 -0.5357863 4.0671144

H -0.8519422 1.4435857 4.4657464

H 0.8387958 -2.3633973 3.4078624

H -0.7208952 -1.0155573 4.8160474

C 2.5124038 2.8992697 1.2676524

H 2.7392248 3.0137307 2.3388344

H 1.5630318 3.4158017 1.1090514

C 3.6151968 3.6727507 0.5024914

H 3.7576818 3.2310767 -0.4886216

C 4.9716018 3.6210587 1.2196284

H 5.7281618 4.1800147 0.6580314

H 4.8942628 4.0740597 2.2160624

H 5.3304008 2.5980497 1.3422774

C 3.1784588 5.1317487 0.3096184

H 2.9939248 5.6114697 1.2790624

H 3.9512008 5.7129667 -0.2048706

H 2.2566758 5.1951947 -0.2773996

#### TS-6

| 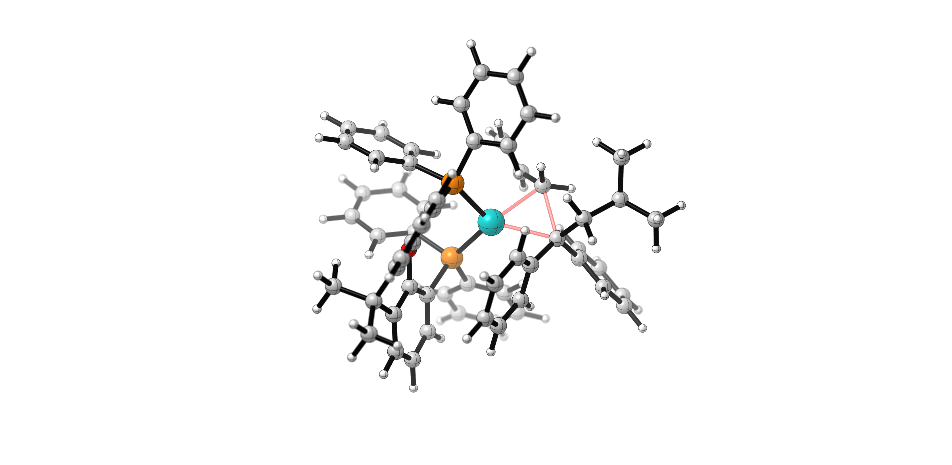 | E/hartree | -3168.236341 |
| --- | --- | --- |
|  | E+zvp/hartree | -3167.253951 |
|  | G/hartree | -3167.346782 |
|  | E(_SP)_/hartree | -3169.324886 |
|  | Imaginary frequency/cm^-1^ | -230.54 |

C -2.1538516 2.9664822 1.9703929

C -2.0283126 1.8426452 1.1467499

C -2.5395126 0.6323322 1.6289259

C -3.1481806 0.4860762 2.8748629

C -3.2372746 1.6258362 3.6799059

C -2.7451736 2.8537182 3.2302159

C -2.7781706 -1.9476348 2.6344299

C -2.1788416 -1.6754768 1.4010459

C -1.3299146 -2.5659588 0.7315919

C -1.1001736 -3.8038468 1.3480149

C -1.6812286 -4.1083358 2.5781739

C -2.5089346 -3.1864398 3.2202389

H -1.7810306 3.9254402 1.6283859

H -3.6960106 1.5636602 4.6601169

H -2.8333946 3.7315532 3.8630739

H -0.4449386 -4.5233198 0.8728619

H -1.4851086 -5.0704728 3.0414539

H -2.9457546 -3.4399688 4.1793469

C -3.7227276 -0.8907398 3.2168009

C -5.0943446 -1.0253348 2.5013769

H -5.7905606 -0.2660038 2.8716589

H -4.9842936 -0.8912728 1.4221979

H -5.5218776 -2.0162288 2.6849079

C -3.9248666 -1.0684178 4.7261179

H -4.3638866 -2.0446768 4.9460629

H -2.9804126 -0.9810588 5.2709549

H -4.6182656 -0.3172608 5.1125499

O -2.4137516 -0.4591098 0.7951759

P -1.1716326 1.7670722 -0.4781201

P -0.4205316 -2.0005018 -0.7793331

Pd 0.5769714 0.1173602 -0.5184721

C -2.6194466 1.5613602 -1.6009001

C -2.3948606 1.0839742 -2.8973431

C -3.9217806 1.9161772 -1.2224541

C -3.4464726 0.9876242 -3.8072471

H -1.3939916 0.7866422 -3.1845101

C -4.9774226 1.7953992 -2.1257601

H -4.1096316 2.2917122 -0.2223211

C -4.7408686 1.3376692 -3.4229981

H -3.2561846 0.6134702 -4.8071541

H -5.9831336 2.0679192 -1.8182271

H -5.5621776 1.2491762 -4.1282881

C -0.7285226 3.5291552 -0.7989001

C -1.2714666 4.2726062 -1.8568801

C 0.3064854 4.1057202 -0.0436381

C -0.7875536 5.5493322 -2.1500081

H -2.0701996 3.8512642 -2.4562021

C 0.7799434 5.3848382 -0.3254861

H 0.7436384 3.5407402 0.7710659

C 0.2373354 6.1107532 -1.3883221

H -1.2179986 6.1071632 -2.9767661

H 1.5803964 5.8074152 0.2747159

H 0.6127684 7.1025352 -1.6212401

C -1.7010996 -2.1016178 -2.1013301

C -3.0688496 -2.2578398 -1.8576761

C -1.2525986 -2.0307978 -3.4304171

C -3.9692286 -2.3543478 -2.9205271

H -3.4343716 -2.3162188 -0.8392971

C -2.1477066 -2.1492758 -4.4893251

H -0.1930916 -1.8952228 -3.6292751

C -3.5125666 -2.3126558 -4.2365711

H -5.0296156 -2.4685428 -2.7158061

H -1.7830056 -2.1085418 -5.5118871

H -4.2141276 -2.3975198 -5.0611291

C 0.5224374 -3.5392038 -1.1904131

C 1.8927394 -3.5654268 -0.9153141

C -0.0780516 -4.6794208 -1.7479531

C 2.6563944 -4.7031348 -1.1796621

H 2.3634754 -2.6804708 -0.5098261

C 0.6823484 -5.8166418 -2.0157191

H -1.1398566 -4.6735748 -1.9715591

C 2.0511724 -5.8323348 -1.7306291

H 3.7201004 -4.6938658 -0.9612401

H 0.2075184 -6.6926108 -2.4485661

H 2.6401614 -6.7198208 -1.9433861

C 1.7283714 0.7909232 -2.7143531

C 0.9373724 1.5015292 -3.5441191

C 2.3043124 1.3175572 -1.4545961

H 2.0772644 -0.1837648 -3.0513771

H 0.6314414 1.1037862 -4.5066041

H 3.3838984 1.2582402 -1.5165351

H 1.9924974 2.3355532 -1.2384921

H 0.5723754 2.4878162 -3.2737901

C 2.8112484 0.5851632 0.5411689

C 3.8961984 -0.4459318 0.2946309

C 4.7477874 -0.7696208 1.3677699

C 4.1196174 -1.0911778 -0.9240141

C 5.7796954 -1.6926918 1.2228679

H 4.5797854 -0.3049878 2.3338419

C 5.1575514 -2.0152018 -1.0783131

H 3.4580704 -0.8997578 -1.7562541

C 5.9939384 -2.3194038 -0.0074621

H 6.4144394 -1.9263148 2.0726759

H 5.2969714 -2.5026388 -2.0386411

H 6.7993764 -3.0381098 -0.1247451

C 1.8313504 0.1436272 1.6074459

C 1.0880094 1.0811932 2.3627289

C 1.7235984 -1.2134378 2.0039569

C 0.2647594 0.6874652 3.4148389

H 1.1471914 2.1333392 2.1234129

C 0.9111794 -1.6007608 3.0621659

H 2.2899574 -1.9668388 1.4743679

C 0.1694374 -0.6550098 3.7737929

H -0.3126796 1.4387122 3.9428469

H 0.8421504 -2.6519298 3.3203629

H -0.4733576 -0.9641648 4.5912179

C 3.4291414 1.9581852 0.9225819

H 3.6338744 1.9051952 1.9998389

H 2.6629724 2.7275002 0.8180779

C 4.7222694 2.4352082 0.2028459

H 4.9946954 1.7096102 -0.5717121

C 5.9038964 2.5032142 1.1809329

H 6.8135784 2.8439522 0.6749509

H 5.6924574 3.2055242 1.9965789

H 6.1102594 1.5244242 1.6214179

C 4.5194104 3.8008052 -0.4689391

H 4.2651834 4.5620482 0.2791229

H 5.4321514 4.1295552 -0.9771241

H 3.7112504 3.7779332 -1.2046631

#### Int-10

| 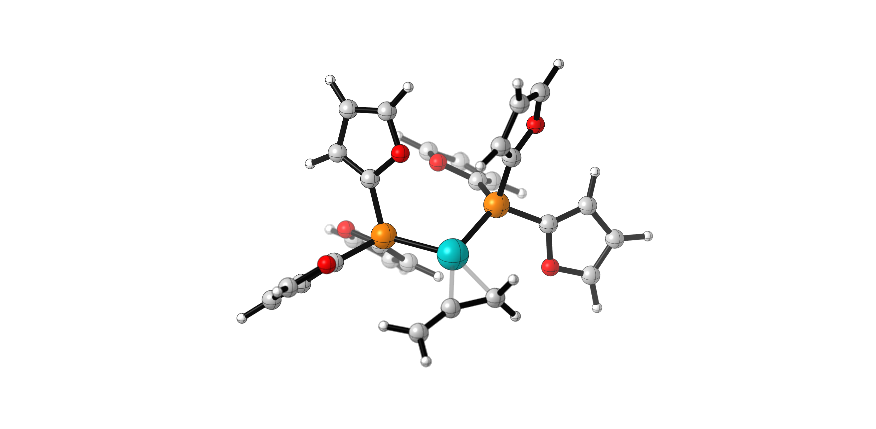 | E/hartree | -2304.097357 |
| --- | --- | --- |
|  | E+zvp/hartree | -2303.671058 |
|  | G/hartree | -2303.745155 |
|  | E(_SP)_/hartree | -2304.871907 |

P -1.8282996 -0.2291023 0.0555161

P 1.8024814 -0.3606813 0.0627761

Pd -0.0770866 -1.7486633 0.1005741

C 3.2377674 -0.9478553 -0.8780409

C 4.5928564 -0.7827073 -0.7759509

O 2.9576974 -1.7549903 -1.9573919

C 5.1814294 -1.5290483 -1.8467689

H 5.0944574 -0.1931093 -0.0238449

C 4.1442174 -2.0925943 -2.5294139

H 6.2326944 -1.6336083 -2.0727189

H 4.0822534 -2.7330233 -3.3953589

C 2.5057784 -0.0740793 1.7011361

C 2.0883394 -0.5340893 2.9199411

O 3.5868154 0.7646097 1.8734601

C 2.9589364 0.0427857 3.8997921

H 1.2435484 -1.1878623 3.0779851

C 3.8438304 0.8171987 3.2088931

H 2.9297004 -0.1012523 4.9700551

H 4.6750724 1.4392467 3.5034131

C 1.6885064 1.3224887 -0.6170019

C 2.3072774 1.9816647 -1.6447509

O 0.6756034 2.1033717 -0.1209139

C 1.6353564 3.2404667 -1.7869469

H 3.1420024 1.6103547 -2.2198709

C 0.6573314 3.2592517 -0.8385419

H 1.8543714 4.0211137 -2.5008569

H -0.1004196 3.9708547 -0.5521089

C -1.7451096 1.0598917 1.3303781

C -2.4106326 2.2301467 1.5793341

O -0.7155316 0.9248637 2.2298461

C -1.7467016 2.8541607 2.6830731

H -3.2604386 2.5970357 1.0236201

C -0.7252586 2.0178457 3.0318431

H -1.9970646 3.7945017 3.1527941

H 0.0433704 2.0476157 3.7882301

C -3.5792666 -0.7028793 0.1156031

C -4.6640206 -0.4794953 -0.6885719

O -3.9408846 -1.5263083 1.1541341

C -5.7576226 -1.2042353 -0.1115959

H -4.6767636 0.1367967 -1.5740879

C -5.2640916 -1.8143213 1.0018891

H -6.7720556 -1.2593793 -0.4790289

H -5.6935006 -2.4557403 1.7553031

C -1.8108376 0.7250487 -1.4794399

C -1.0345696 0.5679177 -2.5947809

O -2.6232446 1.8279537 -1.6398249

C -1.3815256 1.6265907 -3.4939459

H -0.2965016 -0.2080233 -2.7337339

C -2.3446016 2.3568837 -2.8648979

H -0.9621406 1.8199647 -4.4702229

H -2.9068436 3.2374487 -3.1350759

C 0.6034714 -3.8026183 0.0737071

C -0.7814796 -3.7092963 0.1165631

H 1.1685894 -4.0362993 0.9745511

H 1.1220104 -3.9867623 -0.8651859

C -1.9794726 -4.2717623 0.1550931

H -2.8877676 -3.6819033 0.2096891

H -2.0920326 -5.3563813 0.1416831

#### TS-7

| 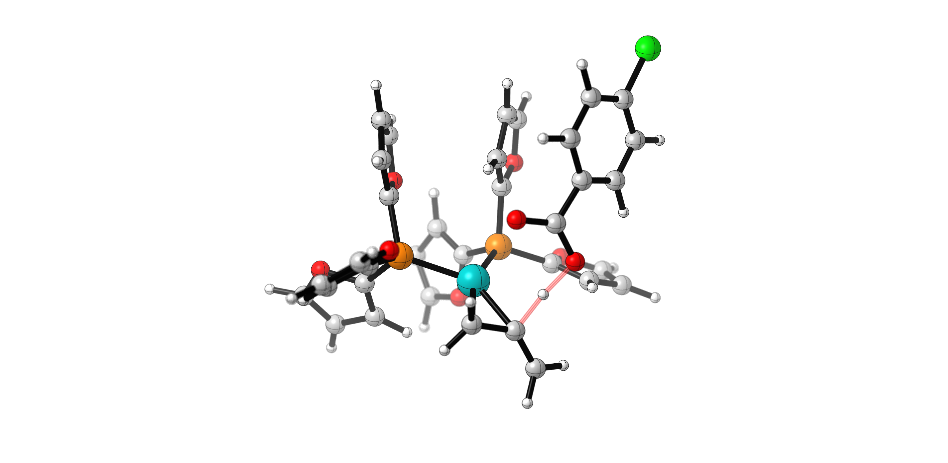 | E/hartree | -3184.544986 |
| --- | --- | --- |
|  | E+zvp/hartree | -3184.015841 |
|  | G/hartree | -3184.102183 |
|  | E(_SP)_/hartree | -3185.561514 |
|  | Imaginary frequency/cm^-1^ | -1244.60 |

P 0.4270866 1.4929111 -0.1466010

P -2.2545694 -0.8926829 0.1249240

Pd -0.7650934 -0.0980119 -1.4090370

H 0.7681936 -0.4947269 -3.1017420

C -1.4634474 -1.2020899 -3.0719140

H -1.1779904 -2.2375759 -2.9091320

H -2.5206434 -1.0251869 -3.2665850

C -0.4764524 -0.2804539 -3.5407940

C -0.5114474 0.6632901 -4.5052060

H 0.2463876 1.4418871 -4.5469190

H -1.2364184 0.6482031 -5.3197110

O 1.1794866 -2.2765799 -1.4253680

C 2.1135516 -1.5619179 -1.8302460

O 2.0489656 -0.7347489 -2.8251800

C 3.4215506 -1.5852659 -1.0825940

C 3.7079416 -2.6683269 -0.2450750

C 4.3052016 -0.5033819 -1.1318410

C 4.8545796 -2.6751459 0.5434950

H 3.0025146 -3.4912599 -0.2079910

C 5.4438076 -0.4812489 -0.3287580

H 4.0778136 0.3350041 -1.7786250

C 5.7041236 -1.5693899 0.5019670

H 5.0857386 -3.5133409 1.1914110

H 6.1189216 0.3669001 -0.3404330

Cl 7.1315856 -1.5445479 1.5321390

C 1.5713886 2.5678851 -1.0407740

C 2.2395136 2.3880301 -2.2236060

O 1.9357686 3.7707841 -0.4809080

C 3.0640946 3.5443311 -2.4020770

H 2.1778356 1.5083641 -2.8475250

C 2.8354346 4.3453721 -1.3217090

H 3.7317056 3.7497631 -3.2261310

H 3.2076626 5.3123771 -1.0202680

C -0.5881374 2.6895631 0.7529320

C -0.8559064 2.8993041 2.0756940

O -1.3499854 3.5381271 -0.0142900

C -1.8383004 3.9410421 2.1325270

H -0.3978854 2.3767781 2.8998410

C -2.0974324 4.2921501 0.8426780

H -2.2838184 4.3707101 3.0177770

H -2.7430154 5.0231911 0.3821960

C 1.4139706 0.7528901 1.1576970

C 1.6835776 -0.5668899 1.4000650

O 2.1418796 1.5443711 2.0230100

C 2.6253206 -0.5978189 2.4757610

H 1.2760366 -1.4015249 0.8486300

C 2.8661096 0.7021991 2.8099100

H 3.0758206 -1.4724379 2.9192410

H 3.4986296 1.1756171 3.5450370

C -1.6301544 -1.3589949 1.7603750

C -1.2784424 -2.5655479 2.3008740

O -1.2926884 -0.3477009 2.6224610

C -0.6945284 -2.2812239 3.5779270

H -1.4151524 -3.5300409 1.8358630

C -0.7279354 -0.9268399 3.7196720

H -0.3051304 -2.9933879 4.2905010

H -0.4083404 -0.2493249 4.4951150

C -3.5735134 0.2714211 0.4865630

C -3.8332684 1.4982001 -0.0609690

O -4.5040874 -0.0060109 1.4638410

C -4.9876024 2.0097781 0.6115190

H -3.2459464 1.9730261 -0.8317700

C -5.3478264 1.0597771 1.5211390

H -5.4753014 2.9586621 0.4447690

H -6.1383414 0.9926911 2.2526650

C -3.1660534 -2.3882889 -0.3246680

C -4.4958114 -2.7039959 -0.3820290

O -2.4188774 -3.4517029 -0.7660450

C -4.5738974 -4.0421739 -0.8884210

H -5.3106844 -2.0624489 -0.0839710

C -3.2893844 -4.4455199 -1.0984780

H -5.4678044 -4.6209419 -1.0691140

H -2.8408834 -5.3558999 -1.4637130

#### Int-11

| 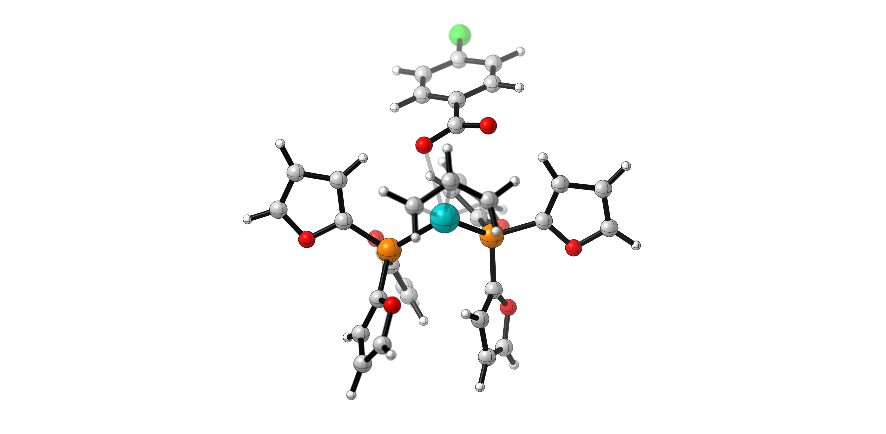 | E/hartree | -3184.596615 |
| --- | --- | --- |
|  | E+zvp/hartree | -3184.059906 |
|  | G/hartree | -3184.143307 |
|  | E(_SP)_/hartree | -3185.613011 |

P -0.1331895 1.7416580 -0.0685356

P -1.7914855 -1.3338090 -0.0202826

Pd -0.7965295 0.0950410 -1.5535086

H 0.4034285 -0.4916250 -3.9612766

C -1.6208435 -0.9236190 -3.3379196

H -1.4908535 -1.9982160 -3.4020316

H -2.6457885 -0.5650150 -3.2996496

C -0.5707145 -0.0704590 -3.7350556

C -0.6222225 1.2960880 -3.4118886

H 0.2755275 1.8912870 -3.5108056

H -1.5729935 1.8231950 -3.3691186

O 1.4445145 -1.3175730 -1.7091156

C 2.4002965 -0.5112930 -1.9558076

O 2.3535005 0.5009540 -2.6950526

C 3.7052605 -0.7888840 -1.2260996

C 3.8818185 -1.9705720 -0.4984886

C 4.7242025 0.1677860 -1.2235226

C 5.0443735 -2.1932310 0.2364124

H 3.0822885 -2.7033720 -0.5054086

C 5.8908765 -0.0302340 -0.4877976

H 4.5764745 1.0729850 -1.8015866

C 6.0347225 -1.2110820 0.2390684

H 5.1859755 -3.1082150 0.8012664

H 6.6776275 0.7158290 -0.4711436

Cl 7.4995045 -1.4691790 1.1853704

C 0.6775075 3.2233780 -0.7004496

C 1.6999885 3.3541970 -1.6025056

O 0.3279205 4.4534340 -0.1953106

C 1.9985275 4.7534990 -1.6587286

H 2.1681575 2.5297220 -2.1298486

C 1.1396275 5.3673460 -0.7947936

H 2.7504315 5.2349610 -2.2670166

H 0.9755745 6.3951550 -0.5104006

C 0.9895845 1.1323860 1.1907114

C 1.6909385 -0.0424680 1.2291554

O 1.4210475 1.9695620 2.1968954

C 2.6048165 0.0682110 2.3234934

H 1.5797755 -0.8554060 0.5279354

C 2.3950845 1.3001780 2.8705654

H 3.3350075 -0.6647840 2.6326764

H 2.8353155 1.8288750 3.7020064

C -1.5923295 2.3583710 0.7939544

C -2.9193345 2.2280580 0.4740214

O -1.4715695 3.0197790 1.9899514

C -3.6575705 2.8421470 1.5347754

H -3.3084325 1.7298540 -0.4010176

C -2.7279445 3.3002730 2.4240434

H -4.7308355 2.9246220 1.6229034

H -2.7932225 3.8245610 3.3648804

C -1.4516875 -3.0646780 -0.3416416

C -0.4276035 -3.6393550 -1.0471136

O -2.2297675 -4.0374590 0.2466134

C -0.5866335 -5.0535200 -0.8917846

H 0.3539735 -3.0875410 -1.5565776

C -1.6881585 -5.2335620 -0.1067396

H 0.0369805 -5.8299250 -1.3104096

H -2.2004815 -6.1074920 0.2655634

C -1.4560135 -1.1793400 1.7540244

C -1.8652805 -0.2983100 2.7204564

O -0.4484115 -1.9679230 2.2469154

C -1.0628935 -0.5638110 3.8757574

H -2.6369155 0.4486150 2.6171924

C -0.2214715 -1.5786730 3.5310404

H -1.1090495 -0.0605990 4.8301894

H 0.5558145 -2.1141160 4.0518604

C -3.5889425 -1.1452120 -0.0040446

C -4.5559965 -1.3645450 0.9388704

O -4.1702445 -0.6270770 -1.1400026

C -5.7997945 -0.9633360 0.3569524

H -4.3862905 -1.7596340 1.9285954

C -5.5064675 -0.5284310 -0.9015226

H -6.7778985 -0.9979300 0.8140754

H -6.1006055 -0.1405040 -1.7141866

#### Int-12

| 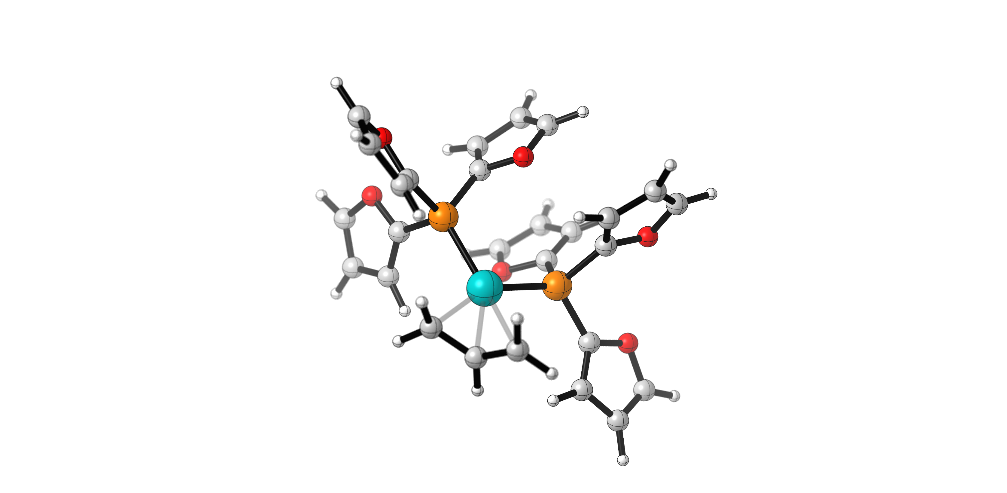 | E/hartree | -2304.699278 |
| --- | --- | --- |
|  | E+zvp/hartree | -2304.262252 |
|  | G/hartree | -2304.337501 |
|  | E(_SP)_/hartree | -2305.474762 |

P -1.8324374 0.2453186 -0.0077783

P 1.7860666 0.1667216 0.0683237

Pd -0.0018344 1.6653946 0.1766177

C -3.2694934 0.8129466 -0.9477493

C -3.4521944 1.9686066 -1.6603913

O -4.3921804 0.0248656 -1.0633123

C -4.7576164 1.8950116 -2.2418263

H -2.7294274 2.7648886 -1.7504823

C -5.2769694 0.6980786 -1.8445403

H -5.2406434 2.6308296 -2.8679823

H -6.2182004 0.2014456 -2.0240963

C -2.5030484 -0.1768064 1.6146447

C -2.0776864 0.2150966 2.8548897

O -3.5729744 -1.0355484 1.7488887

C -2.9246824 -0.4355144 3.8080707

H -1.2434184 0.8747526 3.0411737

C -3.8090154 -1.1774144 3.0811727

H -2.8842944 -0.3557654 4.8846687

H -4.6333324 -1.8217164 3.3458837

C -1.5437214 -1.4087244 -0.6980683

C -1.7977334 -2.6963994 -0.3118433

O -0.8063434 -1.4195594 -1.8596703

C -1.1780694 -3.5461014 -1.2845243

H -2.3579234 -2.9927694 0.5603187

C -0.5926864 -2.7200334 -2.1967673

H -1.1654004 -4.6263134 -1.2934253

H -0.0065184 -2.8789324 -3.0881883

C 1.4336126 -1.4344264 0.8451067

C 1.7368226 -2.7438014 0.5900227

O 0.6234426 -1.3642964 1.9564277

C 1.0732046 -3.5223454 1.5916037

H 2.3557306 -3.0962704 -0.2195383

C 0.4139826 -2.6351114 2.3897967

H 1.0799546 -4.5978324 1.6938877

H -0.2246304 -2.7329114 3.2536337

C 2.2538506 -0.3333964 -1.6025383

C 1.7738566 0.1098926 -2.8041673

O 3.2052496 -1.3069554 -1.8217263

C 2.4561446 -0.6304204 -3.8219073

H 1.0055256 0.8584856 -2.9258943

C 3.3074106 -1.4725124 -3.1685643

H 2.3327206 -0.5430704 -4.8915573

H 4.0256936 -2.2072894 -3.4984433

C 3.3672826 0.6366986 0.8134887

C 3.7598696 1.8298816 1.3599857

O 4.4061876 -0.2629894 0.8893827

C 5.1132446 1.6611886 1.7936317

H 3.1434756 2.7125316 1.4338317

C 5.4485506 0.3761946 1.4830527

H 5.7462796 2.3940376 2.2722617

H 6.3451996 -0.2095964 1.6160137

C -0.8523094 3.6567276 0.5835887

H -1.4908784 3.9394496 -0.2498053

H -1.3293934 3.7009136 1.5580307

C 0.5477136 3.8815276 0.4863847

C 1.2496346 4.1309106 -0.7034863

H 1.1137066 3.9153756 1.4171907

H 2.3281236 4.2353126 -0.7073343

H 0.7325196 4.1817366 -1.6563673

#### TS-8

| 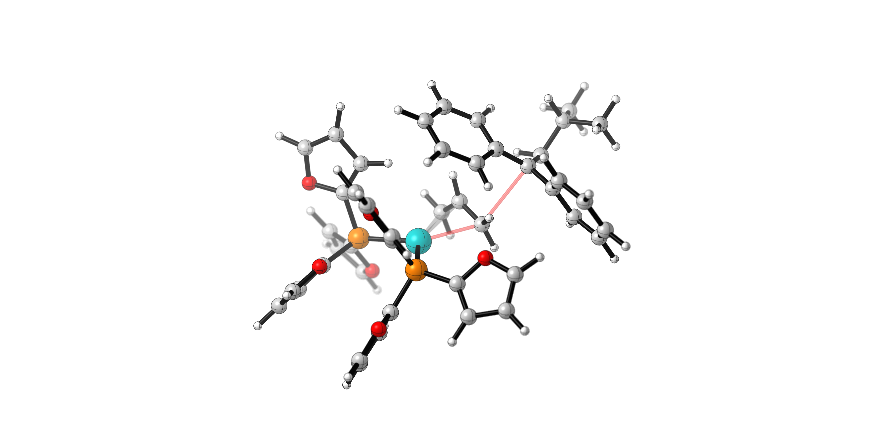 | E/hartree | -2964.039292 |
| --- | --- | --- |
|  | E+zvp/hartree | -2963.288294 |
|  | G/hartree | -2963.381084 |
|  | E(_SP)_/hartree | -2965.091376 |
|  | Imaginary frequency/cm^-1^ | -132.55 |

P -1.3528273 -2.0263593 -0.1407023

P -3.3989683 1.0168007 -0.4041133

Pd -1.2979953 0.1685947 -0.9267313

C -4.1379313 2.1177397 -1.6323953

C -4.8792113 3.2665727 -1.5742013

O -3.9102253 1.7858867 -2.9497163

C -5.1233953 3.6672167 -2.9269973

H -5.2064113 3.7530637 -0.6679773

C -4.5173493 2.7338167 -3.7146323

H -5.6727533 4.5341117 -3.2640033

H -4.4242603 2.6060037 -4.7817463

C -3.2158653 2.0692237 1.0443567

C -2.1136613 2.3181627 1.8173407

O -4.3098643 2.7254137 1.5720177

C -2.5436373 3.1808917 2.8752727

H -1.1236663 1.9103357 1.6754867

C -3.8760853 3.3937367 2.6763147

H -1.9348693 3.5827857 3.6718537

H -4.6251353 3.9657337 3.2020877

C -4.8280653 -0.0253073 -0.0149823

C -6.0870093 -0.1326073 -0.5441973

O -4.6347383 -1.0191323 0.9113087

C -6.7047243 -1.2543843 0.0974957

H -6.5159563 0.5199937 -1.2895603

C -5.7808993 -1.7511953 0.9674847

H -7.7020693 -1.6346463 -0.0683973

H -5.7737893 -2.5763303 1.6616657

C -1.4829473 -2.0089593 1.6600267

C -1.1949893 -2.9030333 2.6562187

O -1.9437933 -0.8414073 2.2151227

C -1.5052373 -2.2506463 3.8918647

H -0.8061533 -3.9001753 2.5124547

C -1.9547593 -1.0038213 3.5604947

H -1.3981593 -2.6523203 4.8889367

H -2.2897973 -0.1509403 4.1293527

C -0.0247463 -3.2186343 -0.4129783

C 0.0187197 -4.5012073 -0.8943973

O 1.2547997 -2.7718403 -0.1922693

C 1.4034347 -4.8595173 -0.9696113

H -0.8354013 -5.1089773 -1.1513833

C 2.1091117 -3.7770773 -0.5328293

H 1.8164417 -5.7986313 -1.3076343

H 3.1593197 -3.5555943 -0.4190473

C -2.7784433 -2.9633903 -0.7243043

C -3.6561803 -2.6862833 -1.7372763

O -3.1360123 -4.1467623 -0.1182203

C -4.6075163 -3.7546473 -1.7621583

H -3.6268823 -1.8112843 -2.3693283

C -4.2446923 -4.6072023 -0.7620143

H -5.4517543 -3.8636283 -2.4265903

H -4.6499563 -5.5365713 -0.3926053

C -0.4408563 2.0814427 -1.7884043

C 0.5324917 1.4240427 -1.0220723

H -0.8562963 3.0220687 -1.4434653

H -0.5791593 1.8503107 -2.8424613

C 1.0917487 0.1849947 -1.3757603

H 1.1032827 -0.1407773 -2.4133893

H 1.5948317 -0.4300143 -0.6567173

C 3.5891447 0.7899457 -0.1257163

C 2.8298787 1.0012517 1.0772797

C 2.5214837 2.3093747 1.5642397

C 2.1781257 -0.0692513 1.7677537

C 1.6482687 2.5177547 2.6276097

H 2.9789307 3.1734227 1.0977587

C 1.3040537 0.1490637 2.8209167

H 2.3296967 -1.0863003 1.4295057

C 1.0228257 1.4461087 3.2732837

H 1.4588637 3.5362737 2.9596967

H 0.8238937 -0.7040753 3.2912287

H 0.3483917 1.6109777 4.1083087

C 4.2301797 -0.4887833 -0.4205903

C 4.7196417 -1.3451593 0.5998607

C 4.4554997 -0.9245793 -1.7522993

C 5.3845327 -2.5334793 0.3101597

H 4.6088567 -1.0397023 1.6342337

C 5.1158617 -2.1122023 -2.0402503

H 4.0802627 -0.3201763 -2.5720433

C 5.5913407 -2.9362993 -1.0131663

H 5.7593757 -3.1438453 1.1282257

H 5.2545217 -2.4056033 -3.0778273

H 6.1126727 -3.8614303 -1.2387713

C 3.9576507 1.9854127 -0.9746303

H 3.1139227 2.6880057 -1.0192603

H 4.1352917 1.6754047 -2.0110493

C 5.2159357 2.7701127 -0.5154763

H 5.0774727 3.0450197 0.5385127

C 6.4863817 1.9184347 -0.6101313

H 6.4150087 1.0226837 0.0104957

H 6.6581577 1.5897037 -1.6424093

H 7.3650017 2.4898757 -0.2894683

C 5.3592187 4.0595657 -1.3323363

H 4.4701327 4.6938407 -1.2387673

H 6.2272257 4.6452857 -1.0098553

H 5.4912367 3.8298327 -2.3972233

H 0.7519727 1.8078967 -0.0296823

#### Int-13

| 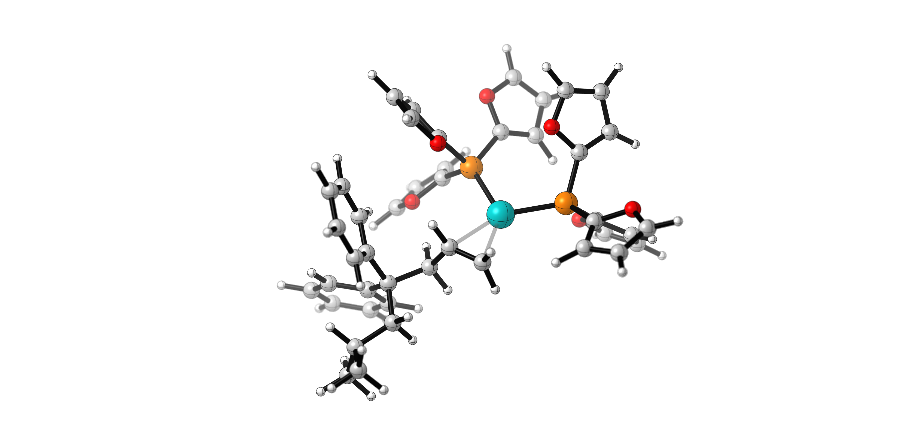 | E/hartree | -2964.111096 |
| --- | --- | --- |
|  | E+zvp/hartree | -2963.354334 |
|  | G/hartree | -2963.447810 |
|  | E(_SP)_/hartree | -2965.156869 |

P 1.3137679 1.7586972 0.0668874

P 3.7111489 -1.1841068 -0.0914196

Pd 1.5091869 -0.5645438 0.1285594

C 4.4180999 -1.3204958 -1.7622996

C 5.3235269 -2.1536208 -2.3622626

O 3.9415829 -0.4253118 -2.6947416

C 5.4140969 -1.7496478 -3.7343016

H 5.8611399 -2.9508068 -1.8728066

C 4.5606579 -0.6980098 -3.8776486

H 6.0305939 -2.1872118 -4.5061156

H 4.2831039 -0.0678288 -4.7079726

C 4.2674949 -2.7686448 0.5930754

C 3.5513569 -3.8319998 1.0717064

O 5.6153539 -3.0519228 0.6845604

C 4.4997769 -4.8254728 1.4762144

H 2.4754659 -3.8840498 1.1237954

C 5.7300299 -4.2981358 1.2202904

H 4.2918199 -5.7948848 1.9050724

H 6.7375679 -4.6576758 1.3629914

C 4.8918879 -0.0205108 0.6558874

C 6.1725399 0.3677992 0.3692244

O 4.4085599 0.7398942 1.6936894

C 6.4956709 1.4262212 1.2766734

H 6.7957319 -0.0543588 -0.4049276

C 5.3896839 1.6086712 2.0535064

H 7.4214519 1.9798572 1.3363154

H 5.1470719 2.2812362 2.8610604

C 0.7320859 2.4184842 1.6559424

C 0.3412779 3.6554832 2.0947824

O 0.6512969 1.5233402 2.6982714

C 0.0097829 3.5185202 3.4789764

H 0.3125289 4.5503452 1.4917474

C 0.2138989 2.2041512 3.7867224

H -0.3399001 4.2894922 4.1504274

H 0.0831809 1.6203342 4.6839554

C 0.2132039 2.6575842 -1.0837636

C 0.4412229 3.5809622 -2.0688526

O -1.1175741 2.3148242 -1.0916926

C -0.8183541 3.8173382 -2.7143206

H 1.3937959 4.0325762 -2.3011576

C -1.7290411 3.0250252 -2.0842926

H -1.0132501 4.4894302 -3.5374616

H -2.7842781 2.8369312 -2.2085346

C 2.7969219 2.7628322 -0.2448746

C 3.8562759 2.5440442 -1.0822416

O 2.9167259 4.0113132 0.3258264

C 4.6802989 3.7134742 -1.0197456

H 4.0203909 1.6545162 -1.6689606

C 4.0657419 4.5644722 -0.1503066

H 5.6116309 3.8848972 -1.5394086

H 4.3101749 5.5429552 0.2335434

C 0.2347199 -2.3231638 0.6829064

C -0.5643991 -1.1849858 0.6625944

H 0.6375489 -2.6909858 1.6223364

H 0.2132749 -3.0335238 -0.1407716

C -1.5148401 -0.8463688 -0.4623186

H -1.3063621 -1.5100528 -1.3078806

H -1.3309481 0.1676912 -0.8070146

C -3.0439981 -0.9315218 -0.1026176

C -3.2180791 -0.2935978 1.2827864

C -3.5752681 -1.0183978 2.4237514

C -2.9108751 1.0687442 1.4441154

C -3.6344161 -0.4074508 3.6805254

H -3.7996301 -2.0744288 2.3517404

C -2.9644171 1.6801932 2.6922424

H -2.6042941 1.6506742 0.5840604

C -3.3296641 0.9434532 3.8220014

H -3.9170641 -0.9979798 4.5474934

H -2.7009281 2.7282552 2.7842234

H -3.3715281 1.4183112 4.7980094

C -3.8379591 -0.1475458 -1.1598916

C -4.9834171 0.5881532 -0.8251846

C -3.4602801 -0.1850878 -2.5105236

C -5.7179041 1.2675652 -1.7977216

H -5.2997611 0.6369662 0.2100764

C -4.1898791 0.4929192 -3.4867976

H -2.5853851 -0.7491508 -2.8117596

C -5.3233221 1.2274982 -3.1354326

H -6.6005071 1.8297552 -1.5062326

H -3.8681531 0.4482562 -4.5233056

H -5.8920961 1.7578592 -3.8933946

C -3.4627781 -2.4259668 -0.1419766

H -2.8636341 -2.9511808 0.6103944

H -3.1355161 -2.8284908 -1.1080786

C -4.9639851 -2.7579988 0.0420184

H -5.4289851 -1.9630588 0.6379874

C -5.7093751 -2.8389398 -1.2988986

H -5.6329621 -1.9123518 -1.8693706

H -5.2975451 -3.6483748 -1.9143676

H -6.7721671 -3.0520968 -1.1392206

C -5.1307811 -4.0799078 0.8044794

H -4.6727871 -4.0378658 1.7988894

H -6.1882931 -4.3348778 0.9323364

H -4.6541351 -4.9027438 0.2579554

H -0.7336281 -0.6646628 1.6002394

#### TS-9

| 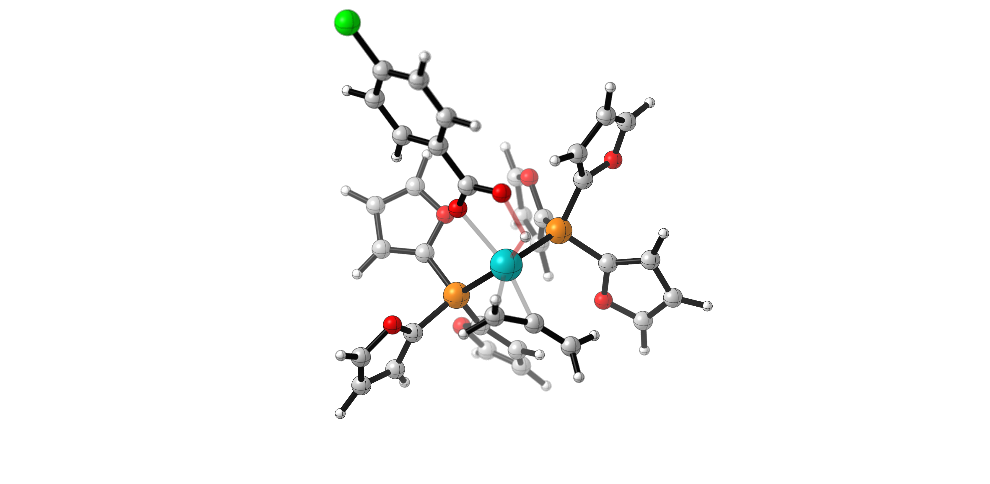 | E/hartree | -3184.528439 |
| --- | --- | --- |
|  | E+zvp/hartree | -3183.998881 |
|  | G/hartree | -3184.084409 |
|  | E(_SP)_/hartree | -3185.544386 |
|  | Imaginary frequency/cm^-1^ | -205.24 |

P 0.7538483 -1.8410335 -0.0164382

P 0.9475483 1.8544045 -0.0272422

Pd 0.0823093 -0.0148095 -1.4001802

H -0.8772887 -0.9894305 -2.1607642

C 0.2579083 0.9421865 -3.3608612

H -0.5711237 0.6229215 -3.9863522

H 0.3601823 2.0127275 -3.2121972

C 1.3074963 0.0781295 -3.1033442

C 2.4385603 -0.5209255 -3.4304432

H 2.9170343 -1.2501985 -2.7888352

H 2.9180063 -0.3056415 -4.3843482

O -2.5673527 -1.0530115 -2.1565542

C -2.8186167 -0.2643755 -1.1958662

O -1.9520937 0.3916135 -0.5404142

C -4.2483197 -0.1012405 -0.7678922

C -5.2690487 -0.7788315 -1.4427872

C -4.5663227 0.7234485 0.3181168

C -6.5963077 -0.6391875 -1.0444712

H -5.0041077 -1.4130495 -2.2810682

C -5.8884957 0.8715785 0.7284038

H -3.7635527 1.2399515 0.8323448

C -6.8900487 0.1866965 0.0400598

H -7.3940507 -1.1594665 -1.5622762

H -6.1455757 1.5069735 1.5683968

Cl -8.5629687 0.3683905 0.5534218

C 2.6532153 1.7662665 0.5496228

C 3.7300313 1.1489585 -0.0272562

O 3.0400113 2.3725105 1.7225528

C 4.8448803 1.3794105 0.8403118

H 3.7065103 0.5842415 -0.9465502

C 4.3667843 2.1177975 1.8841948

H 5.8609153 1.0367455 0.7088198

H 4.8165233 2.5197425 2.7789138

C 0.9210293 3.5188405 -0.7387042

C 1.7692893 4.5908835 -0.6701702

O -0.1472687 3.8309555 -1.5456622

C 1.1901033 5.6242915 -1.4753642

H 2.6875973 4.6317065 -0.1037682

C 0.0317383 5.1096805 -1.9769342

H 1.5852363 6.6134655 -1.6547942

H -0.7385207 5.4970495 -2.6252032

C -0.0598687 2.1185495 1.4452608

C -0.6249317 3.2313745 2.0088288

O -0.5272597 0.9972525 2.0751468

C -1.4878187 2.7634605 3.0507388

H -0.4471417 4.2522885 1.7060308

C -1.3857257 1.4022875 3.0433828

H -2.0993687 3.3602985 3.7115498

H -1.8292647 0.6156915 3.6322548

C 1.1264183 -1.5544335 1.7432098

C 2.2086103 -1.0271035 2.3947708

O 0.1140453 -1.7862705 2.6355718

C 1.8405213 -0.9339045 3.7760078

H 3.1469153 -0.7447245 1.9447258

C 0.5656063 -1.4049485 3.8641868

H 2.4499823 -0.5673455 4.5890038

H -0.1244977 -1.5464655 4.6809688

C 2.2890123 -2.6453635 -0.5624142

C 2.5396573 -3.8209555 -1.2120972

O 3.4570833 -1.9371765 -0.4242412

C 3.9471773 -3.8439935 -1.4870302

H 1.8091863 -4.5770635 -1.4556712

C 4.4547773 -2.6848145 -0.9856602

H 4.5012803 -4.6261755 -1.9850372

H 5.4440183 -2.2583855 -0.9354672

C -0.3409927 -3.2643265 0.0628888

C -1.5486727 -3.5250325 -0.5277602

O 0.0313673 -4.3336175 0.8527278

C -1.9442857 -4.8253725 -0.0794352

H -2.0823927 -2.8600305 -1.1932022

C -0.9543197 -5.2615425 0.7509468

H -2.8476177 -5.3568595 -0.3404302

H -0.8071967 -6.1656605 1.3218258

#### Int-14

| 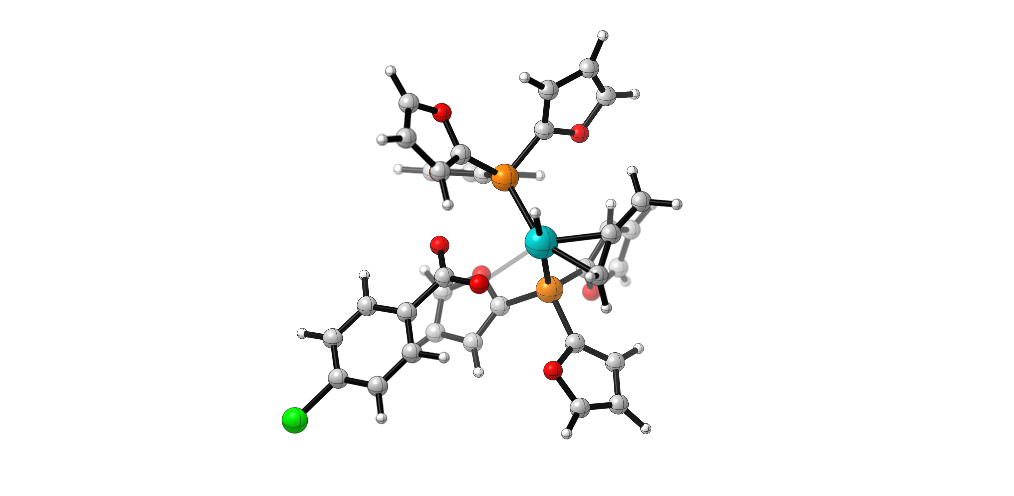 | E/hartree | -3184.530279 |
| --- | --- | --- |
|  | E+zvp/hartree | -3183.998998 |
|  | G/hartree | -3184.083675 |
|  | E(_SP)_/hartree | -3185.546243 |

P -1.3875345 1.5882049 -0.2182289

P -0.0623165 -1.8573291 0.1072701

Pd -0.6232895 -0.2131261 -1.5930669

H -0.9211295 0.8250389 -2.6930069

C -0.9076555 -1.6978301 -3.1522709

H -0.5655425 -1.2850061 -4.0973079

H -0.4643025 -2.6442271 -2.8579009

C -2.1141025 -1.2801861 -2.6047329

C -3.4316445 -1.2800971 -2.5457019

H -3.9787395 -0.6428621 -1.8625569

H -4.0094825 -1.9265481 -3.2044629

O 1.5538685 0.2833949 -1.6272549

C 2.0177895 0.9186949 -0.6109729

O 1.3470605 1.4643179 0.2848591

C 3.5224375 0.9743949 -0.5140249

C 4.3301905 0.2490809 -1.3947589

C 4.1169905 1.7248649 0.5050491

C 5.7175915 0.2634519 -1.2608609

H 3.8521595 -0.3303651 -2.1763629

C 5.5014155 1.7558899 0.6500501

H 3.4678535 2.2664949 1.1833441

C 6.2869415 1.0184869 -0.2363199

H 6.3523425 -0.2995281 -1.9360929

H 5.9711155 2.3359189 1.4366891

Cl 8.0389745 1.0392269 -0.0541869

C -1.4388355 -2.4965601 1.0660991

C -2.7711655 -2.1862101 1.0135511

O -1.2189225 -3.4236711 2.0608181

C -3.4108665 -2.9620181 2.0317241

H -3.2233325 -1.4787051 0.3369551

C -2.4231075 -3.6884211 2.6309021

H -4.4612085 -2.9746341 2.2831531

H -2.4125615 -4.4040471 3.4385961

C 0.6167865 -3.3642261 -0.6269509

C 0.1666375 -4.6536621 -0.7010699

O 1.7519315 -3.2262691 -1.3862799

C 1.0835645 -5.3560541 -1.5501919

H -0.7036935 -5.0497021 -0.2006159

C 2.0227325 -4.4459491 -1.9318789

H 1.0459515 -6.3983991 -1.8305849

H 2.9050015 -4.4955191 -2.5502389

C 1.1944135 -1.4444891 1.3304281

C 2.5502415 -1.6267551 1.3666521

O 0.8029075 -0.6320831 2.3572151

C 3.0224225 -0.8745881 2.4893531

H 3.1355155 -2.1957141 0.6614781

C 1.9240185 -0.2897381 3.0460091

H 4.0462585 -0.7706611 2.8156701

H 1.7634175 0.3609309 3.8901791

C -1.2721025 1.6951359 1.5905721

C -1.8916745 0.9740119 2.5761361

O -0.4159085 2.6012069 2.1468171

C -1.3757055 1.4637189 3.8175471

H -2.6094075 0.1824179 2.4315501

C -0.4860205 2.4468009 3.4953611

H -1.6355195 1.1274649 4.8107541

H 0.1336785 3.1134819 4.0744401

C -3.1915535 1.7892899 -0.4215289

C -3.9814525 2.6886379 -1.0791499

O -3.9800685 0.7955809 0.1138591

C -5.3343615 2.2311229 -0.9398149

H -3.6381825 3.5771659 -1.5855929

C -5.2789595 1.0874349 -0.2058279

H -6.2254355 2.7020679 -1.3287549

H -6.0199745 0.3983679 0.1673591

C -0.8307075 3.1815119 -0.8290099

C 0.0932435 3.4904639 -1.7882329

O -1.3643635 4.3364769 -0.3059489

C 0.1330235 4.9179409 -1.8677839

H 0.6837325 2.7715249 -2.3363309

C -0.7645135 5.3752519 -0.9478429

H 0.7536145 5.5194109 -2.5156269

H -1.0804835 6.3602979 -0.6411129

#### TS-10

| 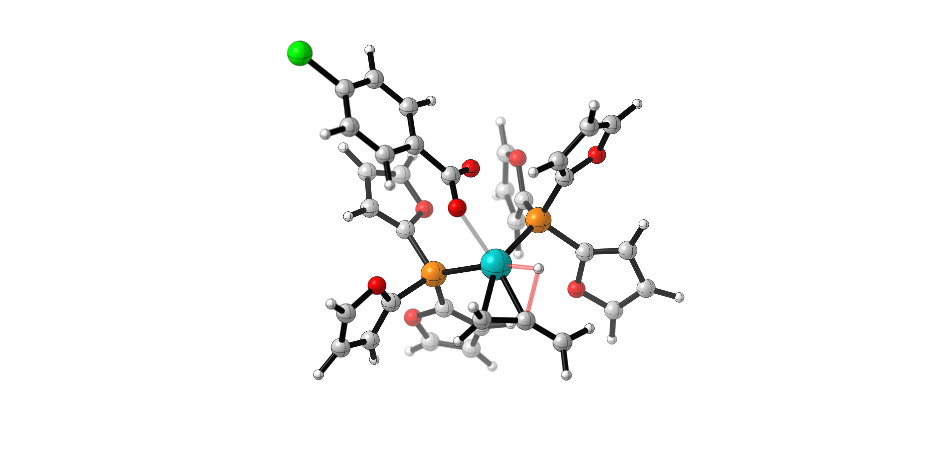 | E/hartree | -3184.519220 |
| --- | --- | --- |
|  | E+zvp/hartree | -3183.989896 |
|  | G/hartree | -3184.074901 |
|  | E(_SP)_/hartree | -3185.544386 |
|  | Imaginary frequency/cm^-1^ | -688.38 |

P 1.4743078 -1.5085293 -0.0675355

P -0.0829272 1.8283927 0.1354065

Pd 0.7873918 0.2651977 -1.4501525

H 1.5495878 -0.5208153 -2.5980695

C 0.9705908 1.7307637 -3.0222535

H 0.2281358 1.4881847 -3.7783285

H 0.9476938 2.7434377 -2.6299825

C 2.1448418 0.9775067 -2.9096705

C 3.4334298 0.8230957 -3.1829285

H 4.0252078 0.0346167 -2.7324485

H 3.9224058 1.4885817 -3.8903725

O -1.4386732 -0.4604313 -1.7183195

C -1.8981742 -1.0442953 -0.6788765

O -1.2275062 -1.5226623 0.2635875

C -3.4037652 -1.1346023 -0.5782435

C -4.2230052 -0.4336963 -1.4677225

C -3.9871922 -1.8829183 0.4484625

C -5.6101662 -0.4670163 -1.3339085

H -3.7509742 0.1446027 -2.2539895

C -5.3713532 -1.9368483 0.5917775

H -3.3296942 -2.4020863 1.1361935

C -6.1678992 -1.2208873 -0.3021245

H -6.2536792 0.0795427 -2.0144695

H -5.8324292 -2.5165803 1.3838435

Cl -7.9205302 -1.2671973 -0.1200635

C 1.2164988 2.6238427 1.0877745

C 2.5700188 2.4172287 1.0561035

O 0.9156848 3.5745087 2.0376725

C 3.1381138 3.2879457 2.0402275

H 3.0775168 1.7075007 0.4198365

C 2.0915298 3.9605147 2.6003735

H 4.1817538 3.3945607 2.2974605

H 2.0176218 4.7084157 3.3748785

C -0.8882262 3.2361057 -0.6663665

C -0.6625612 4.5855787 -0.6498715

O -1.8843592 2.9457067 -1.5643125

C -1.5774882 5.1624567 -1.5904365

H 0.0557038 5.0981627 -0.0286505

C -2.2932352 4.1254917 -2.1091335

H -1.6863042 6.2079677 -1.8388325

H -3.0899802 4.0544707 -2.8326625

C -1.3256322 1.3540437 1.3488935

C -2.6893682 1.4674197 1.3633915

O -0.9083062 0.5775427 2.3934045

C -3.1397122 0.7078837 2.4897785

H -3.2937892 1.9949797 0.6420705

C -2.0212892 0.1886627 3.0710765

H -4.1617982 0.5556907 2.8022255

H -1.8408672 -0.4378463 3.9293865

C 1.3175748 -1.5987053 1.7344505

C 1.9028388 -0.8414623 2.7140325

O 0.4732988 -2.5126093 2.2935005

C 1.3768538 -1.3172783 3.9564195

H 2.6028058 -0.0345463 2.5621225

C 0.5171388 -2.3287693 3.6399115

H 1.6100238 -0.9540133 4.9466875

H -0.0954042 -2.9988863 4.2226155

C 3.2943508 -1.5867683 -0.2415875

C 4.1641768 -2.4755033 -0.8091905

O 3.9842308 -0.4551973 0.1338365

C 5.4617718 -1.8629313 -0.7803905

H 3.9073288 -3.4521793 -1.1883955

C 5.2961648 -0.6447423 -0.1962505

H 6.3900758 -2.2826803 -1.1399295

H 5.9658748 0.1618457 0.0572055

C 1.0387248 -3.1230553 -0.7140525

C 0.1988288 -3.4633083 -1.7372985

O 1.6177098 -4.2536183 -0.1856475

C 0.2601548 -4.8881353 -1.8520475

H -0.4056402 -2.7656183 -2.2988735

C 1.1277338 -5.3120693 -0.8885965

H -0.2795772 -5.5111823 -2.5500575

H 1.4858108 -6.2827803 -0.5825635

#### TS-11

| 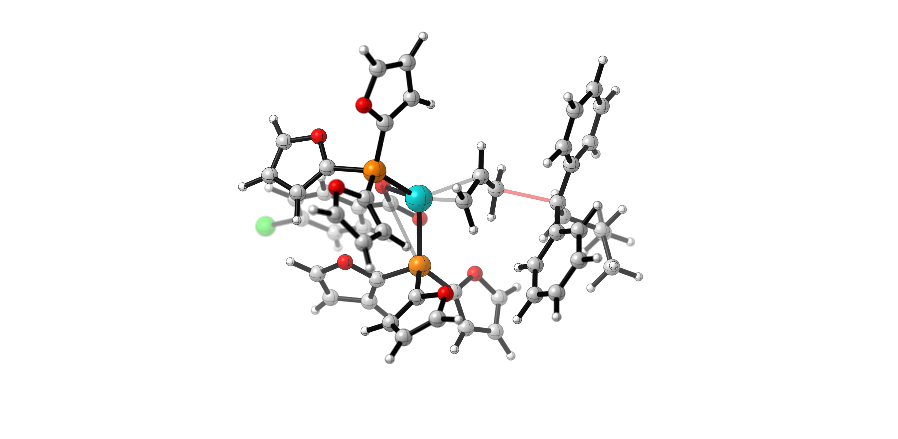 | E/hartree | -3843.914431 |
| --- | --- | --- |
|  | E+zvp/hartree | -3843.066192 |
|  | G/hartree | -3843.173744 |
|  | E(_SP)_/hartree | -3845.205403 |
|  | Imaginary frequency/cm^-1^ | -457.58 |

P -1.0787592 -1.0024143 1.3284514

P -2.1904142 2.7744457 -0.1125366

Pd -0.9934892 0.8221047 -0.3288666

H 1.3085518 2.0315307 -1.7532776

C 0.8692068 1.6216327 0.3287234

H 0.8380398 2.6845597 0.5550664

H 1.2131548 1.0105807 1.1564864

C 1.2462688 1.2509087 -1.0005136

C 1.5319228 -0.0576543 -1.3918836

H 1.5684298 -0.2926023 -2.4491846

H 1.3048258 -0.8849453 -0.7354336

O -2.6363272 0.0300477 -1.4359446

C -2.6257772 -1.2524173 -1.5770176

O -1.6384852 -1.9915533 -1.4235716

C -3.9756972 -1.8605753 -1.8552796

C -5.1203152 -1.0603823 -1.9224756

C -4.1016622 -3.2497803 -1.9360326

C -6.3810142 -1.6356563 -2.0613436

H -5.0090112 0.0125827 -1.8399326

C -5.3544762 -3.8427083 -2.0744626

H -3.2016662 -3.8501343 -1.8651886

C -6.4825852 -3.0245313 -2.1303396

H -7.2735162 -1.0211673 -2.1052866

H -5.4627572 -4.9201203 -2.1310136

Cl -8.0752982 -3.7643193 -2.2817486

C 3.7587258 -0.4706083 -1.0941956

C 4.2835028 0.5075327 -2.0602116

C 4.2316838 1.8969797 -1.7967856

C 4.7753628 0.1161717 -3.3240096

C 4.6677368 2.8308257 -2.7282816

H 3.8129458 2.2395297 -0.8583386

C 5.2158088 1.0539487 -4.2555576

H 4.8236048 -0.9335123 -3.5853566

C 5.1708098 2.4173107 -3.9655986

H 4.6060918 3.8893557 -2.4919136

H 5.5965738 0.7135417 -5.2141366

H 5.5133648 3.1470707 -4.6926516

C 3.9109308 -0.2338993 0.3597474

C 4.8833538 0.6423037 0.8838264

C 3.1275168 -0.9503503 1.2895224

C 5.0495618 0.8023757 2.2584264

H 5.5412898 1.1735517 0.2072064

C 3.2922538 -0.7914413 2.6596724

H 2.3754058 -1.6422203 0.9389384

C 4.2529868 0.0918507 3.1570104

H 5.8171608 1.4772967 2.6261994

H 2.6579648 -1.3559373 3.3342144

H 4.3870608 0.2151117 4.2276654

C 3.6782478 -1.9229743 -1.5273276

H 3.2807778 -1.9699083 -2.5460786

H 2.9529638 -2.4528813 -0.9043436

C 5.0076608 -2.7240373 -1.4774026

H 5.7876108 -2.1314743 -1.9721186

C 5.4757028 -3.0196903 -0.0469136

H 4.6973228 -3.5501583 0.5144324

H 6.3697328 -3.6525733 -0.0626906

H 5.7156568 -2.1095743 0.5048684

C 4.8299508 -4.0305123 -2.2621146

H 4.0423368 -4.6481273 -1.8123626

H 4.5466178 -3.8413913 -3.3031566

H 5.7521808 -4.6205863 -2.2632306

C -0.0737602 -2.5146363 1.4346644

C 0.3734908 -3.2494243 2.5042804

O 0.4632558 -3.0280483 0.2806044

C 1.2200318 -4.2753293 1.9779284

H 0.1189598 -3.0684503 3.5380504

C 1.2333628 -4.0932393 0.6251644

H 1.7428058 -5.0428463 2.5296834

H 1.7109708 -4.6093303 -0.1927486

C -0.8412572 -0.4350133 3.0438114

C -1.6280322 -0.4164773 4.1630174

O 0.3483598 0.2018877 3.3166904

C -0.8805342 0.2608437 5.1833624

H -2.6180042 -0.8396953 4.2417384

C 0.3100978 0.6078747 4.6193384

H -1.1907012 0.4547957 6.1999094

H 1.1940238 1.1124497 4.9762504

C -2.7569652 -1.6596163 1.4890454

C -3.2876482 -2.9133283 1.6172684

O -3.7725812 -0.7412533 1.3722044

C -4.7114112 -2.7611233 1.5752654

H -2.7230652 -3.8287043 1.7093094

C -4.9470882 -1.4272523 1.4245464

H -5.4545662 -3.5430833 1.6223094

H -5.8392772 -0.8328793 1.3137714

C -3.9756652 2.6342057 -0.3625926

C -4.9935202 2.2852677 0.4830514

O -4.4563982 2.7759247 -1.6356566

C -6.1777902 2.2158637 -0.3188386

H -4.8992962 2.0993697 1.5420784

C -5.7947092 2.5223717 -1.5918466

H -7.1790202 1.9783457 0.0105224

H -6.3192312 2.6098737 -2.5300936

C -2.1231612 3.4576377 1.5527584

C -1.4317102 3.0261417 2.6535764

O -2.8755752 4.5618297 1.8876714

C -1.7714842 3.9096697 3.7250294

H -0.7681672 2.1786997 2.6811214

C -2.6466142 4.8167077 3.2018844

H -1.4152892 3.8649907 4.7434104

H -3.1738222 5.6636207 3.6137534

C -1.7101362 4.1206397 -1.2089046

C -0.9233202 4.0845007 -2.3297296

O -2.1777042 5.3993487 -1.0162866

C -0.8996862 5.4138517 -2.8575346

H -0.4447082 3.2012507 -2.7252956

C -1.6737122 6.1632987 -2.0207106

H -0.3789902 5.7613827 -3.7376576

H -1.9483632 7.2067527 -1.9995216

#### Int-15

| 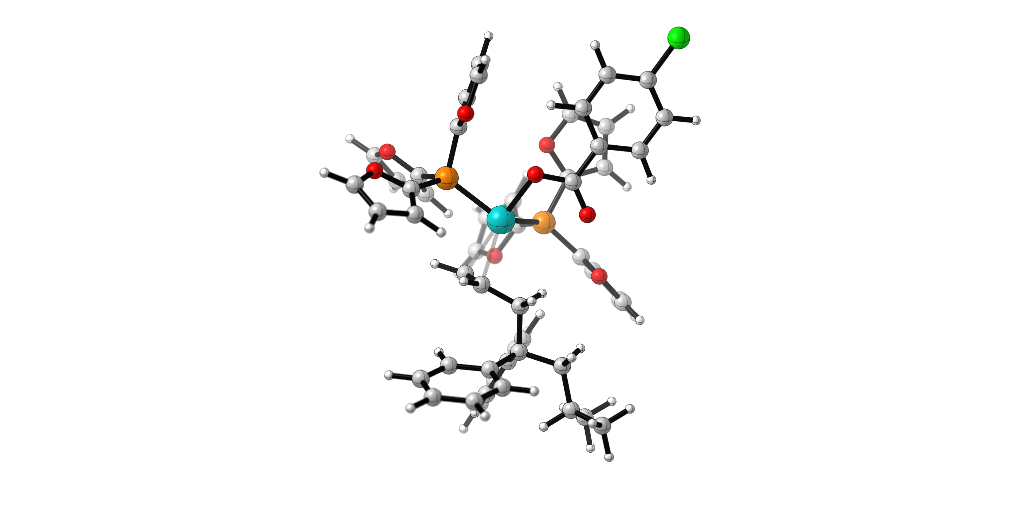 | E/hartree | -3843.952592 |
| --- | --- | --- |
|  | E+zvp/hartree | -3843.101303 |
|  | G/hartree | -3843.209553 |
|  | E(_SP)_/hartree | -3845.242097 |

P -1.1795054 -1.1217308 1.3146783

P -2.0823464 2.8900472 -0.0074127

Pd -1.0088654 0.8029592 -0.2252407

H 1.2490626 2.0045572 -1.4563167

C 1.0346626 1.5144662 0.5851073

H 0.8853726 2.5515412 0.8687283

H 1.2676896 0.8319742 1.3909113

C 1.2777026 1.1962642 -0.7312427

C 1.7563636 -0.1394898 -1.2379527

H 1.4993316 -0.2207278 -2.2977497

H 1.2161216 -0.9481068 -0.7437027

O -2.7330514 0.1848052 -1.3564907

C -2.7140214 -1.0797488 -1.6105517

O -1.7064894 -1.8078488 -1.5531057

C -4.0605074 -1.6859008 -1.9023597

C -5.2177084 -0.9008508 -1.8838767

C -4.1673924 -3.0658498 -2.0944947

C -6.4718704 -1.4836528 -2.0499307

H -5.1218464 0.1642242 -1.7153017

C -5.4136104 -3.6659028 -2.2576307

H -3.2572944 -3.6551648 -2.0915707

C -6.5543994 -2.8638078 -2.2288517

H -7.3743924 -0.8826338 -2.0310197

H -5.5071074 -4.7367958 -2.3995947

Cl -8.1382124 -3.6142598 -2.4135557

C 3.3010486 -0.4190628 -1.1195057

C 4.0164046 0.6816172 -1.9140337

C 4.1404996 1.9727482 -1.3708937

C 4.4700786 0.4869302 -3.2241917

C 4.6941116 3.0196842 -2.1041987

H 3.8043496 2.1552442 -0.3571077

C 5.0296506 1.5324322 -3.9626917

H 4.3891086 -0.4869698 -3.6893337

C 5.1443886 2.8048132 -3.4081087

H 4.7751516 4.0047732 -1.6536247

H 5.3752876 1.3447982 -4.9751407

H 5.5799636 3.6179692 -3.9809467

C 3.7577826 -0.4802868 0.3451183

C 5.0251986 -0.0385438 0.7482063

C 2.9580956 -1.1169418 1.3050643

C 5.4673286 -0.2118238 2.0609523

H 5.6800366 0.4389412 0.0292163

C 3.3901036 -1.2879928 2.6179613

H 1.9861016 -1.5011538 1.0317863

C 4.6509816 -0.8333678 3.0056283

H 6.4565176 0.1385612 2.3414013

H 2.7336886 -1.7803008 3.3284053

H 4.9954086 -0.9667428 4.0269523

C 3.5006666 -1.8365588 -1.7338527

H 3.1241526 -1.8132308 -2.7622077

H 2.8282986 -2.5136778 -1.1974047

C 4.9319696 -2.4249488 -1.7093057

H 5.6494206 -1.5953308 -1.6769007

C 5.1742816 -3.3152468 -0.4807027

H 4.4854286 -4.1693498 -0.4895447

H 6.1948356 -3.7134648 -0.4866567

H 5.0257126 -2.7783498 0.4566013

C 5.2007556 -3.2294178 -2.9889837

H 4.4827436 -4.0528128 -3.0865127

H 5.1134616 -2.6076968 -3.8864227

H 6.2055986 -3.6647728 -2.9797297

C -0.2306104 -2.6693158 1.3251193

C 0.1918726 -3.4846588 2.3454923

O 0.3145806 -3.1070628 0.1437883

C 1.0327426 -4.4805538 1.7580093

H -0.0718254 -3.3722878 3.3867333

C 1.0687136 -4.2008588 0.4224743

H 1.5416316 -5.2900908 2.2602803

H 1.5570026 -4.6601118 -0.4224757

C -0.9281494 -0.6565358 3.0576463

C -1.7359224 -0.6201848 4.1613023

O 0.2976566 -0.1138518 3.3756043

C -0.9658464 -0.0314018 5.2188753

H -2.7554224 -0.9721888 4.2054003

C 0.2578286 0.2507192 4.6906493

H -1.2835344 0.1484832 6.2356603

H 1.1663036 0.6786182 5.0841993

C -2.8831884 -1.7183158 1.4264313

C -3.4636554 -2.9564198 1.4385573

O -3.8609964 -0.7524038 1.4064773

C -4.8799744 -2.7442398 1.4243763

H -2.9362784 -3.8982348 1.4391243

C -5.0617904 -1.3936908 1.4038233

H -5.6538274 -3.4968948 1.4021413

H -5.9303204 -0.7567958 1.3624193

C -3.8684734 2.8651292 -0.2847237

C -4.9180324 2.5649482 0.5411533

O -4.3123954 3.0004152 -1.5714077

C -6.0836594 2.5204102 -0.2897657

H -4.8571964 2.3942962 1.6053413

C -5.6589174 2.7914962 -1.5575057

H -7.1003114 2.3229682 0.0181713

H -6.1573334 2.8827212 -2.5095447

C -2.0035354 3.4752992 1.6956933

C -1.4353344 2.8885962 2.7964803

O -2.6226714 4.6470682 2.0713713

C -1.7143074 3.7421642 3.9089633

H -0.8933034 1.9577232 2.7998243

C -2.4331434 4.7887042 3.4086783

H -1.4206054 3.5888442 4.9366753

H -2.8634914 5.6736652 3.8519823

C -1.4943104 4.2563572 -1.0220867

C -0.6806434 4.2213772 -2.1243637

O -1.8823494 5.5532972 -0.7799817

C -0.5553374 5.5691342 -2.5863877

H -0.2494184 3.3297652 -2.5545437

C -1.3017564 6.3280322 -1.7332207

H 0.0115756 5.9217362 -3.4353207

H -1.5083414 7.3852952 -1.6683607

#### Int-16

| 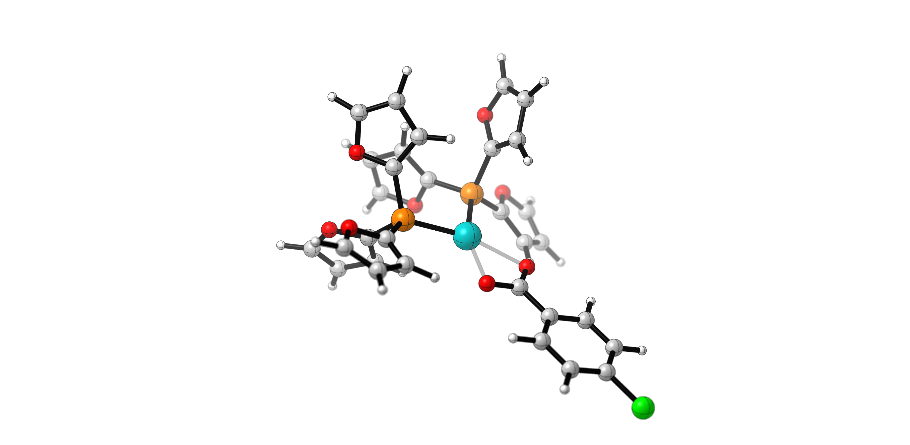 | E/hartree | -3067.262808 |
| --- | --- | --- |
|  | E+zvp/hartree | -3066.798406 |
|  | G/hartree | -3066.881834 |
|  | E(_SP)_/hartree | -3068.231184 |

P -0.9451433 -1.7551929 -0.0181740

P -0.7614423 1.7602411 0.0020860

Pd 0.6821277 -0.0759809 -0.0022820

O 2.7303227 0.9682761 -0.0533210

C 3.3304537 -0.1547789 -0.0065950

O 2.6913737 -1.2502529 0.0523780

C 4.8246487 -0.1781179 -0.0197520

C 5.5479937 1.0197181 -0.0431170

C 5.5098367 -1.3982159 -0.0091000

C 6.9400127 1.0057351 -0.0549090

H 5.0029787 1.9567791 -0.0494290

C 6.9015387 -1.4286009 -0.0223090

H 4.9357127 -2.3175109 0.0081460

C 7.6017517 -0.2222749 -0.0447650

H 7.5090537 1.9282221 -0.0715420

H 7.4410817 -2.3687519 -0.0154990

Cl 9.3590057 -0.2501869 -0.0604290

C -0.1182323 3.2055381 -0.8486400

C 1.1410277 3.4359111 -1.3377970

O -0.9120853 4.3110141 -1.0568690

C 1.1234917 4.7586621 -1.8830910

H 1.9655297 2.7388191 -1.2779700

C -0.1400993 5.2349221 -1.6865840

H 1.9404707 5.2815151 -2.3584600

H -0.6282013 6.1664641 -1.9292070

C -1.1158853 2.2767651 1.6942970

C -0.3482783 2.1116111 2.8177100

O -2.2689253 2.9481221 2.0204250

C -1.0689043 2.7115121 3.8982700

H 0.6104187 1.6135971 2.8505180

C -2.2244353 3.1951771 3.3573740

H -0.7687253 2.7704091 4.9341640

H -3.0772953 3.7143571 3.7664770

C -2.4108873 1.5580131 -0.7043210

C -3.6093423 1.1234371 -0.2074140

O -2.4900803 1.6722551 -2.0707380

C -4.4798523 0.9636641 -1.3335170

H -3.8401353 0.9404221 0.8300570

C -3.7525653 1.3085851 -2.4320220

H -5.5094413 0.6378871 -1.3186390

H -3.9676453 1.3503151 -3.4874940

C -1.7781363 -1.8347909 -1.6118980

C -1.4744453 -1.1832529 -2.7783220

O -2.8251673 -2.6996509 -1.8282650

C -2.3894643 -1.6645449 -3.7667020

H -0.6975693 -0.4432859 -2.8983620

C -3.1811423 -2.5770849 -3.1328320

H -2.4471403 -1.3710639 -4.8045860

H -4.0040533 -3.1967169 -3.4544320

C -0.3511553 -3.4242489 0.2889630

C 0.9323137 -3.8825299 0.4340560

O -1.2391303 -4.4705059 0.4077320

C 0.8315547 -5.2934569 0.6527550

H 1.8202167 -3.2675199 0.3839620

C -0.4998863 -5.5896389 0.6277090

H 1.6417607 -5.9905319 0.8095950

H -1.0594853 -6.5047839 0.7468360

C -2.2723793 -1.5165489 1.1847950

C -2.2526323 -0.8736489 2.3950360

O -3.5356053 -1.9974019 0.9431330

C -3.5784193 -0.9578079 2.9261070

H -1.4017593 -0.3768279 2.8327950

C -4.3114013 -1.6443639 2.0013720

H -3.9336983 -0.5563519 3.8637770

H -5.3444063 -1.9515249 1.9488620

#### TS-12

| 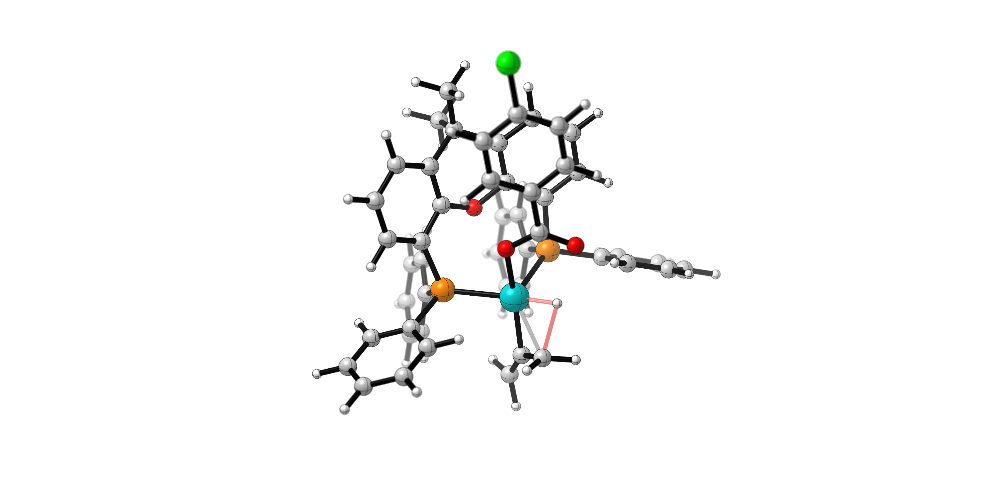 | E/hartree | -3388.750345 |
| --- | --- | --- |
|  | E+zvp/hartree | -3387.990516 |
|  | G/hartree | -3388.076010 |
|  | E(_SP)_/hartree | -3389.809609 |
|  | Imaginary frequency/cm^-1^ | -569.50 |

C 2.2373538 2.8562286 0.3012158

C 1.2071458 1.9161406 0.4276358

C 1.5041918 0.7239656 1.0916538

C 2.7501708 0.4317616 1.6473788

C 3.7597348 1.3825316 1.4844538

C 3.5035308 2.5822696 0.8163878

C 2.0637808 -1.9316224 1.6776188

C 0.8590698 -1.5261314 1.0959398

C -0.0006472 -2.3899094 0.4102918

C 0.3851478 -3.7330484 0.3088608

C 1.5843218 -4.1688094 0.8720778

C 2.4175668 -3.2760474 1.5484778

H 2.0499288 3.7855806 -0.2237152

H 4.7534678 1.1884266 1.8704328

H 4.3007518 3.3087386 0.6958158

H -0.2410132 -4.4329974 -0.2305412

H 1.8729818 -5.2110034 0.7793588

H 3.3496268 -3.6316864 1.9724798

C 2.8704238 -0.8736954 2.4361948

C 2.1986948 -0.6582854 3.8204658

H 2.7304458 0.1186606 4.3786198

H 1.1569958 -0.3466334 3.7089298

H 2.2211198 -1.5862214 4.4006068

C 4.3288928 -1.2893834 2.6542478

H 4.3835798 -2.2125054 3.2373598

H 4.8509638 -1.4380024 1.7072538

H 4.8600288 -0.5239404 3.2258558

O 0.4807058 -0.2029294 1.1828948

P -0.4476192 2.0540986 -0.3446982

P -1.4666132 -1.6962404 -0.4637952

Pd -0.8737142 0.1296356 -1.9338842

C -1.5791032 2.2301986 1.0871248

C -2.9569242 2.1913716 0.8248748

C -1.1406662 2.4040096 2.4052288

C -3.8778582 2.3391116 1.8584508

H -3.2999872 2.0508496 -0.1944672

C -2.0654872 2.5399446 3.4417108

H -0.0779802 2.4407216 2.6195198

C -3.4336252 2.5122856 3.1709388

H -4.9410462 2.3011016 1.6429158

H -1.7145882 2.6748176 4.4609048

H -4.1512882 2.6174236 3.9791198

C -0.4310832 3.7398246 -1.0693932

C -1.0785872 4.8302456 -0.4734102

C 0.2469508 3.9201956 -2.2875162

C -1.0562712 6.0824326 -1.0891922

H -1.6000802 4.7024456 0.4686228

C 0.2763038 5.1780096 -2.8887622

H 0.7680958 3.0797966 -2.7378062

C -0.3796562 6.2596246 -2.2964682

H -1.5650162 6.9202656 -0.6211072

H 0.8099828 5.3104416 -3.8254662

H -0.3627002 7.2355096 -2.7729482

C -2.7355832 -1.4546224 0.8395598

C -2.3864182 -1.1013034 2.1500588

C -4.0952622 -1.5440714 0.4993638

C -3.3757102 -0.8564344 3.1000998

H -1.3461542 -1.0049394 2.4296958

C -5.0814232 -1.2944944 1.4531748

H -4.3821152 -1.8279024 -0.5068152

C -4.7242142 -0.9520134 2.7573498

H -3.0880812 -0.5772594 4.1083678

H -6.1283992 -1.3755504 1.1759148

H -5.4915602 -0.7580414 3.5006828

C -2.0851472 -3.1466994 -1.4006402

C -1.7512782 -3.2346614 -2.7587912

C -2.8180582 -4.1849024 -0.8066372

C -2.1469412 -4.3393324 -3.5133232

H -1.1742122 -2.4333824 -3.2111522

C -3.2179722 -5.2851634 -1.5638062

H -3.0775792 -4.1290554 0.2452368

C -2.8839522 -5.3636534 -2.9179482

H -1.8821642 -4.3974814 -4.5647322

H -3.7883212 -6.0824284 -1.0965322

H -3.1976442 -6.2211484 -3.5056842

H -0.3366862 1.0288176 -3.0891652

C -1.8989832 0.4570696 -3.8849102

H -1.7247502 1.3995066 -4.3970332

H -1.7621202 -0.4311494 -4.4959522

C -2.7288862 0.4264056 -2.7532952

C -3.9735942 0.4706076 -2.3072442

H -4.2103222 0.3950966 -1.2529482

H -4.8075522 0.5759076 -2.9991522

O 1.1533408 -0.5615334 -1.7832212

C 2.0705348 0.2229786 -2.2326912

O 1.8982308 1.2555366 -2.9046102

C 3.4648928 -0.1838804 -1.8356642

C 3.7117508 -1.4485484 -1.2917372

C 4.5156198 0.7279966 -1.9646802

C 4.9933968 -1.8027484 -0.8784172

H 2.8878668 -2.1431634 -1.1860382

C 5.7990568 0.3982776 -1.5349322

H 4.3018708 1.7017006 -2.3900152

C 6.0211778 -0.8665674 -0.9922312

H 5.1934828 -2.7837054 -0.4623162

H 6.6162628 1.1063616 -1.6137262

Cl 7.6331968 -1.2900004 -0.4222082

#### Int-17

| 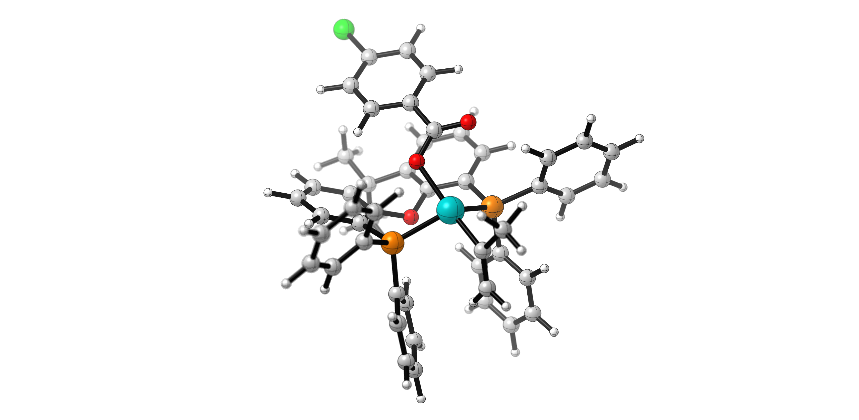 | E/hartree | -3388.807037 |
| --- | --- | --- |
|  | E+zvp/hartree | -3388.041609 |
|  | G/hartree | -3388.128233 |
|  | E(_SP)_/hartree | -3389.866639 |

C 2.3283652 2.7611402 0.2783393

C 1.2620412 1.8602342 0.3929503

C 1.4931602 0.6625832 1.0842953

C 2.7143412 0.3645942 1.6950333

C 3.7582192 1.2786922 1.5460493

C 3.5681752 2.4654422 0.8379283

C 1.9954912 -1.9867278 1.7772593

C 0.8158882 -1.5944178 1.1335763

C -0.0094378 -2.4973948 0.4423913

C 0.3971212 -3.8383148 0.4050973

C 1.5703222 -4.2521788 1.0315723

C 2.3643582 -3.3312608 1.7119573

H 2.1859922 3.6811232 -0.2758497

H 4.7292422 1.0644642 1.9756423

H 4.3923492 3.1615662 0.7216193

H -0.1946218 -4.5609558 -0.1419807

H 1.8676722 -5.2948468 0.9831633

H 3.2791922 -3.6632228 2.1882023

C 2.7815592 -0.9128748 2.5313803

C 2.0602952 -0.6437198 3.8805823

H 2.5702682 0.1554442 4.4275003

H 1.0222802 -0.3422738 3.7187293

H 2.0616182 -1.5485368 4.4964243

C 4.2258442 -1.3377468 2.8197013

H 4.2472412 -2.2396568 3.4366233

H 4.7808182 -1.5257938 1.8978193

H 4.7452282 -0.5596568 3.3846363

O 0.4570742 -0.2573538 1.1726923

P -0.3530768 2.1607792 -0.4214717

P -1.4824348 -1.9258618 -0.5175387

Pd -0.9261508 0.1250042 -1.4411587

C -1.4637218 2.5735642 0.9818803

C -2.7057908 3.1677392 0.7074023

C -1.1446668 2.2588032 2.3095323

C -3.6078218 3.4306262 1.7361163

H -2.9666478 3.4205982 -0.3130337

C -2.0523028 2.5171232 3.3370983

H -0.1834908 1.8223402 2.5493863

C -3.2882618 3.0983872 3.0536743

H -4.5645328 3.8894322 1.5054523

H -1.7860938 2.2709782 4.3610823

H -3.9943348 3.2993282 3.8538653

C -0.1686208 3.7580302 -1.2912237

C -0.3028368 4.9886532 -0.6304107

C 0.1127492 3.7335272 -2.6647637

C -0.1616838 6.1810732 -1.3382397

H -0.5227448 5.0115552 0.4315663

C 0.2534342 4.9311612 -3.3663847

H 0.2633632 2.7776472 -3.1540227

C 0.1131282 6.1533712 -2.7075607

H -0.2671298 7.1306102 -0.8218267

H 0.4750172 4.9069132 -4.4290787

H 0.2192232 7.0837072 -3.2579167

C -2.8258508 -1.7907468 0.7151123

C -2.5817318 -1.0879728 1.9053273

C -4.1355988 -2.1894038 0.4157353

C -3.6255478 -0.8083358 2.7842373

H -1.5876688 -0.7197478 2.1252993

C -5.1749988 -1.9198758 1.3060423

H -4.3502128 -2.6944588 -0.5185957

C -4.9243678 -1.2285658 2.4910953

H -3.4233818 -0.2436688 3.6885193

H -6.1844568 -2.2372878 1.0622803

H -5.7371898 -1.0076438 3.1762903

C -1.9147088 -3.3496888 -1.5769267

C -1.5923358 -3.2475068 -2.9371927

C -2.5176668 -4.5242208 -1.0991707

C -1.8638118 -4.3041118 -3.8069837

H -1.1236798 -2.3377448 -3.3017497

C -2.7933128 -5.5756548 -1.9716197

H -2.7734448 -4.6114938 -0.0482287

C -2.4659848 -5.4668758 -3.3256707

H -1.6087338 -4.2162638 -4.8585237

H -3.2623498 -6.4799158 -1.5955597

H -2.6827108 -6.2873388 -4.0030897

H -2.2470738 1.7776132 -3.5475647

C -2.9197748 0.9660392 -3.2455017

H -3.9441308 1.2564572 -3.5154567

H -2.6528988 0.0873202 -3.8452017

C -2.8173458 0.6864142 -1.7625517

C -3.8469708 0.8138922 -0.9265167

H -3.7669838 0.6365542 0.1382213

H -4.8273038 1.1228082 -1.2935587

O 1.0715812 -0.6222688 -1.5794887

C 1.9740762 0.1388492 -2.1017227

O 1.7722992 1.1648492 -2.7681767

C 3.3831572 -0.2806548 -1.7696787

C 3.6412112 -1.5236308 -1.1824077

C 4.4353942 0.6152802 -1.9744607

C 4.9324922 -1.8682038 -0.7913667

H 2.8172782 -2.2072058 -1.0210657

C 5.7302582 0.2949802 -1.5721917

H 4.2118962 1.5722262 -2.4322087

C 5.9612752 -0.9447328 -0.9775287

H 5.1396222 -2.8312858 -0.3383697

H 6.5491112 0.9924152 -1.7084957

Cl 7.5879072 -1.3526298 -0.4370957

#### Int-18

| 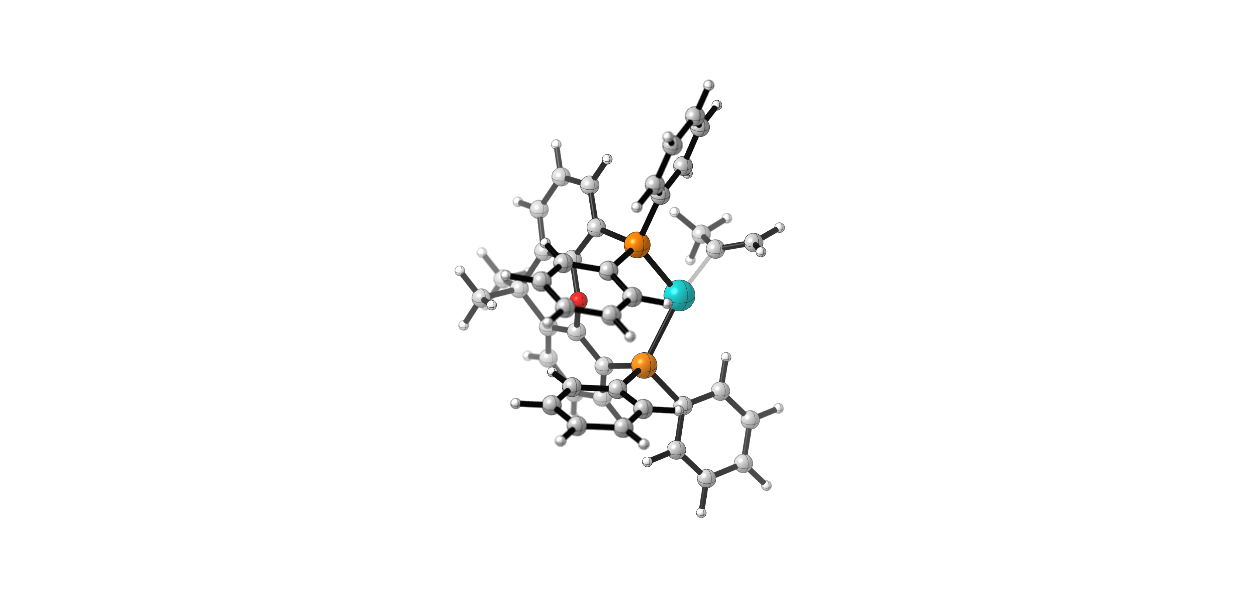 | E/hartree | -2508.733510 |
| --- | --- | --- |
|  | E+zvp/hartree | -2508.063950 |
|  | G/hartree | -2508.140091 |
|  | E(_SP)_/hartree | -2509.580032 |

Pd 0.1320173 -1.1784828 -1.5159266

C 1.6099043 -0.0466278 -3.7892836

H 0.6842543 0.5300482 -3.8944566

H 2.3216433 0.6000802 -3.2631486

C 1.3920423 -1.3311668 -3.0415896

C 1.6831043 -2.5677928 -3.4517936

H 1.5880223 -3.4361998 -2.8061366

H 2.0308793 -2.7544918 -4.4694856

H 2.0012563 -0.2471058 -4.7946616

P 1.8835583 -0.7138538 -0.1382446

C 2.2129343 1.0795072 -0.1838416

C 3.5033063 -1.5327688 -0.3438406

C 1.3843763 -1.1079218 1.5646814

C 3.4499953 1.6516322 -0.5087256

C 1.1305193 1.9390552 0.0456084

C 4.2342353 -1.3898718 -1.5352416

C 4.0426583 -2.2959408 0.7028104

C 1.6757453 -0.2374538 2.6227974

C 0.7704323 -2.3413668 1.8216394

C 3.5584863 3.0360172 -0.6371306

H 4.3152083 1.0195572 -0.6670736

C 1.2067393 3.3259682 -0.0788666

O -0.0575937 1.3435922 0.4046764

C 5.4823233 -1.9907688 -1.6705576

H 3.8334903 -0.8126128 -2.3574156

C 5.2886863 -2.9065668 0.5552384

H 3.4983693 -2.4136238 1.6312354

C 1.3585363 -0.6060228 3.9289394

H 2.1548633 0.7158882 2.4284934

C 0.4706903 -2.7099828 3.1301724

H 0.5276563 -3.0065708 0.9982774

C 2.4484503 3.8607372 -0.4387296

H 4.5155493 3.4769242 -0.8944656

C -0.0485037 4.1397602 0.2543724

C -1.2089147 1.9395052 -0.0776426

C 6.0105783 -2.7553758 -0.6272896

H 6.0393223 -1.8679608 -2.5938006

H 5.6932613 -3.4975338 1.3704984

C 0.7604843 -1.8412308 4.1833144

H 1.5831183 0.0700172 4.7477564

H -0.0103517 -3.6615468 3.3248084

H 2.5600463 4.9323222 -0.5549156

C -1.2632447 3.3278042 -0.2105536

C -0.0130027 5.5351272 -0.3810536

C -0.1272347 4.2829192 1.7989334

C -2.2576707 1.0792292 -0.4173296

H 6.9799823 -3.2303078 -0.7386196

H 0.5139073 -2.1248198 5.2012294

C -2.4537837 3.8670892 -0.7064166

H 0.8449863 6.1040142 -0.0154856

H 0.0426913 5.4816342 -1.4718796

H -0.9029107 6.1056222 -0.1059566

H -1.0345877 4.8246992 2.0810244

H -0.1463067 3.3036712 2.2850344

H 0.7408233 4.8344902 2.1718584

C -3.4399147 1.6585682 -0.8976626

P -1.9591487 -0.7316668 -0.3402596

C -3.5317687 3.0423602 -1.0380746

H -2.5495717 4.9388812 -0.8340246

H -4.2734167 1.0269152 -1.1814736

C -3.3203667 -1.4415488 -1.3280136

C -2.3298737 -1.2210588 1.3798424

H -4.4480187 3.4838832 -1.4154326

C -4.6262897 -1.5845998 -0.8355066

C -3.0391747 -1.8243258 -2.6478486

C -2.2084707 -0.3147748 2.4391264

C -2.7002977 -2.5491908 1.6433114

C -5.6295717 -2.0987678 -1.6555156

H -4.8529027 -1.3022688 0.1872484

C -4.0438137 -2.3364568 -3.4678986

H -2.0280467 -1.7238548 -3.0429576

C -2.4691427 -0.7294048 3.7449304

H -1.9191677 0.7121142 2.2488804

C -2.9713497 -2.9540978 2.9482944

H -2.7989327 -3.2589538 0.8276134

C -5.3403587 -2.4736058 -2.9700876

H -6.6376687 -2.2085058 -1.2686326

H -3.8143617 -2.6323338 -4.4864116

C -2.8570857 -2.0443628 4.0017824

H -2.3757367 -0.0199298 4.5609054

H -3.2753507 -3.9781258 3.1417654

H -6.1241977 -2.8759068 -3.6037566

H -3.0710307 -2.3599238 5.0180144

#### Int-19

| 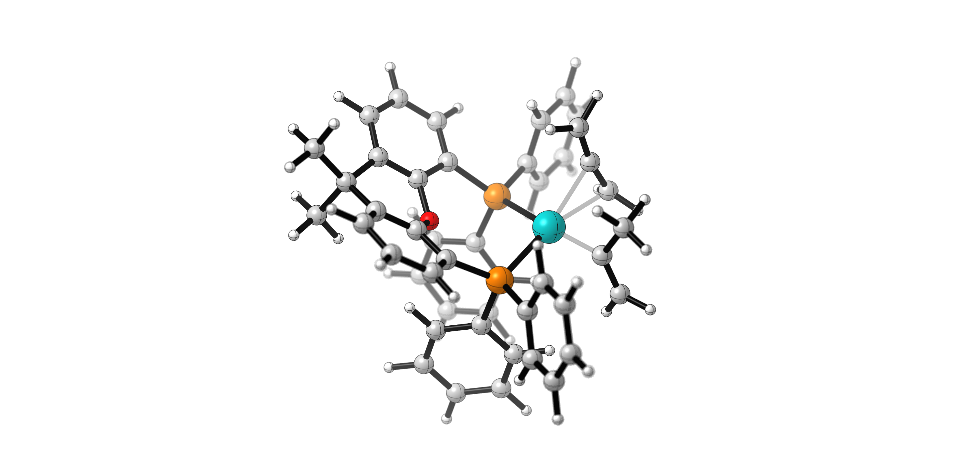 | E/hartree | -2625.430475 |
| --- | --- | --- |
|  | E+zvp/hartree | -2624.701432 |
|  | G/hartree | -2624.780909 |
|  | E(_SP)_/hartree | -2626.322762 |

Pd 0.1579139 -1.7515416 -0.8247010

C 2.0842339 -3.2550576 -2.6745780

H 3.0378169 -3.7915266 -2.7331410

H 1.3336599 -3.8358396 -3.2216440

C 1.6687439 -3.0758276 -1.2407300

C 2.0963239 -3.8607246 -0.2522280

H 1.7267519 -3.7870216 0.7658710

H 2.8451379 -4.6326616 -0.4347460

H 2.1903579 -2.3086766 -3.2078790

C -1.1401201 -3.5480186 -1.5013000

H -2.0070521 -3.5370626 -0.8475910

H -0.4700831 -4.3994156 -1.4370050

C -1.0403831 -2.6734956 -2.5183850

C -1.1007861 -2.0051326 -3.6418070

H -0.5987461 -1.0528916 -3.7779940

H -1.6669781 -2.4060786 -4.4798850

P -1.9517551 -0.5837456 -0.0540320

C -2.4629441 1.0614244 -0.6731590

C -3.4522121 -1.6076436 -0.3297310

C -1.9064381 -0.3809976 1.7607050

C -1.4820321 2.0510984 -0.6180940

C -3.7210431 1.4215294 -1.1722840

C -4.0406441 -2.3372356 0.7134230

C -3.9758161 -1.7351176 -1.6286080

C -2.5184421 0.7008034 2.4053710

C -1.2914981 -1.3832676 2.5236740

C -1.6562721 3.3574474 -1.0695140

O -0.2914101 1.6539344 -0.0510940

C -3.9327151 2.7185914 -1.6408060

H -4.5249351 0.6969184 -1.1997430

C -5.1255771 -3.1787516 0.4609370

H -3.6585421 -2.2466746 1.7232960

C -5.0641571 -2.5699286 -1.8737410

H -3.5330501 -1.1815236 -2.4479900

C -2.5189721 0.7734174 3.7978490

H -2.9988231 1.4790674 1.8223600

C -1.3141811 -1.3186326 3.9150280

H -0.7982611 -2.2143706 2.0272550

C -2.9114651 3.6711804 -1.6008850

C -0.5103241 4.3481414 -0.8537200

C 0.8471859 2.2514254 -0.5343390

H -4.9054071 2.9927904 -2.0352520

C -5.6390901 -3.2984076 -0.8301990

H -5.5726851 -3.7346706 1.2789360

H -5.4622131 -2.6522456 -2.8802660

C -1.9250811 -0.2384256 4.5534030

H -2.9929721 1.6154874 4.2923770

H -0.8397561 -2.0998506 4.4982970

H -3.1067891 4.6708834 -1.9708470

C 0.8094639 3.5780614 -0.9728460

C -0.5781911 5.5248254 -1.8363940

C -0.6174341 4.8857324 0.5997420

C 1.9993509 1.4622954 -0.5219990

H -6.4840691 -3.9515076 -1.0228860

H -1.9344741 -0.1838616 5.6373590

C 2.0156659 4.1329164 -1.4066150

H 0.2227529 6.2410274 -1.6401040

H -0.4988751 5.1882924 -2.8737290

H -1.5166071 6.0712294 -1.7186920

H -1.5607351 5.4230744 0.7349020

H -0.5837081 4.0672934 1.3245360

H 0.2099039 5.5691164 0.8124870

C 3.1984509 2.0723214 -0.9250230

P 1.9097559 -0.2981826 0.0226910

C 3.1983779 3.3926224 -1.3681910

H 2.0429219 5.1567524 -1.7603450

H 4.1256349 1.5167904 -0.9034710

C 3.6153709 -0.8407216 -0.3771840

C 1.8320229 -0.2356386 1.8499540

H 4.1298219 3.8492324 -1.6853280

C 4.5743999 -1.1519156 0.5914790

C 3.9729909 -0.8903326 -1.7335130

C 1.5301399 0.9461764 2.5363670

C 2.0781479 -1.4107106 2.5797180

C 5.8572819 -1.5469616 0.2056770

H 4.3338299 -1.0851466 1.6446080

C 5.2533329 -1.2775596 -2.1154840

H 3.2517639 -0.6046146 -2.4915310

C 1.4884879 0.9539614 3.9304660

H 1.3378919 1.8618414 1.9919210

C 2.0612519 -1.3907866 3.9717830

H 2.3132579 -2.3316256 2.0598640

C 6.1975759 -1.6196536 -1.1439520

H 6.5916849 -1.7910356 0.9666470

H 5.5147229 -1.3114606 -3.1683860

C 1.7638729 -0.2076156 4.6504660

H 1.2488839 1.8744564 4.4527310

H 2.2771279 -2.2994266 4.5251650

H 7.1952619 -1.9281286 -1.4392080

H 1.7442179 -0.1928076 5.7354500

#### TS-13

| 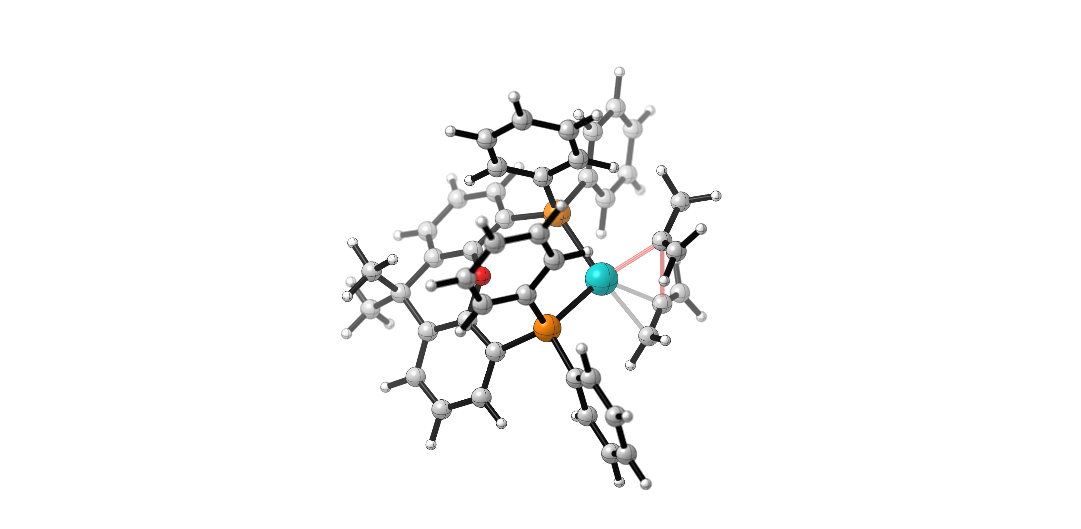 | E/hartree | -2625.415282 |
| --- | --- | --- |
|  | E+zvp/hartree | -2624.686097 |
|  | G/hartree | -2624.763436 |
|  | E(_SP)_/hartree | -2626.307812 |
|  | Imaginary frequency/cm^-1^ | -265.88 |

Pd 0.2086045 -1.6564765 -0.8590040

C 2.9287805 -3.5122635 -0.5459040

H 3.1721905 -4.5728845 -0.4265350

H 3.3158085 -3.1848645 -1.5079060

C 1.4493635 -3.3251475 -0.3991250

C 0.7049065 -4.1023095 0.3977360

H -0.3715725 -4.0222555 0.5005400

H 1.1841715 -4.8683865 1.0080530

H 3.4777695 -2.9463085 0.2096480

C -0.8661755 -3.1188025 -2.0742650

H -1.5690275 -2.5964485 -2.7120490

H -1.2878355 -3.8798875 -1.4260110

C 0.4650065 -3.1454695 -2.4384300

C 1.3946455 -3.2875965 -3.3639980

H 2.4047125 -2.9160575 -3.2541640

H 1.1582595 -3.8388655 -4.2712850

P -1.9652545 -0.6815915 -0.2135180

C -2.6296835 0.9120915 -0.8201620

C -3.3398955 -1.8628765 -0.4857810

C -1.9257075 -0.4564525 1.6032380

C -1.7680315 2.0006095 -0.6878270

C -3.8957755 1.1484485 -1.3706200

C -3.8196395 -2.6612975 0.5610190

C -3.8671365 -2.0410985 -1.7766410

C -2.7247685 0.5086095 2.2314400

C -1.1063815 -1.2858905 2.3797300

C -2.0614555 3.2916145 -1.1203360

O -0.5662395 1.7255985 -0.0711700

C -4.2262215 2.4293475 -1.8148580

H -4.6151475 0.3437265 -1.4540330

C -4.8068535 -3.6185875 0.3199400

H -3.4309265 -2.5318685 1.5645990

C -4.8604255 -2.9898575 -2.0104600

H -3.5110165 -1.4292755 -2.6003660

C -2.6978095 0.6417895 3.6184730

H -3.3649465 1.1536085 1.6395960

C -1.0940905 -1.1591485 3.7678670

H -0.4713585 -2.0230705 1.9007550

C -3.3169735 3.4847595 -1.7050680

C -1.0244735 4.3825795 -0.8427870

C 0.5193825 2.4209785 -0.5490340

H -5.2042725 2.6084115 -2.2487140

C -5.3296695 -3.7847845 -0.9622430

H -5.1723775 -4.2283035 1.1401810

H -5.2655145 -3.1096975 -3.0102820

C -1.8844505 -0.1920475 4.3883220

H -3.3159345 1.3942475 4.0980520

H -0.4510195 -1.8009935 4.3588930

H -3.6009255 4.4670885 -2.0641080

C 0.3625985 3.7434765 -0.9750730

C -1.1948855 5.5872755 -1.7765640

C -1.2012995 4.8449055 0.6295880

C 1.7316575 1.7308605 -0.5694100

H -6.1000525 -4.5267955 -1.1452530

H -1.8662855 -0.0867405 5.4684420

C 1.5080195 4.4009975 -1.4278720

H -0.4685065 6.3675115 -1.5378880

H -1.0721055 5.3053085 -2.8259270

H -2.1834105 6.0346435 -1.6499040

H -2.1938915 5.2828355 0.7702120

H -1.0966145 4.0046465 1.3214410

H -0.4475035 5.5964925 0.8820050

C 2.8655275 2.4374645 -1.0034740

P 1.8105935 -0.0353715 -0.0444580

C 2.7479435 3.7580785 -1.4289240

H 1.4420405 5.4258135 -1.7737240

H 3.8331025 1.9539135 -1.0209160

C 3.4896305 -0.4357075 -0.6530890

C 1.9397795 0.0152785 1.7765500

H 3.6297225 4.2908195 -1.7686240

C 4.6144135 -0.4897705 0.1758060

C 3.6359785 -0.6412585 -2.0333410

C 1.6976525 1.1870395 2.5019070

C 2.2561405 -1.1689485 2.4625300

C 5.8680795 -0.7794205 -0.3682970

H 4.5150425 -0.3129755 1.2408250

C 4.8895005 -0.9147925 -2.5749000

H 2.7649805 -0.5841615 -2.6804190

C 1.7776295 1.1758985 3.8949060

H 1.4524965 2.1071545 1.9846300

C 2.3623575 -1.1677285 3.8506330

H 2.4122735 -2.0900545 1.9129170

C 6.0074425 -0.9956565 -1.7394320

H 6.7363215 -0.8289615 0.2811120

H 4.9957845 -1.0684055 -3.6443410

C 2.1190965 0.0049025 4.5702930

H 1.5821765 2.0876595 4.4500970

H 2.6253795 -2.0832455 4.3715080

H 6.9834385 -1.2188525 -2.1580790

H 2.1931385 0.0032315 5.6531070

#### Int-20

| 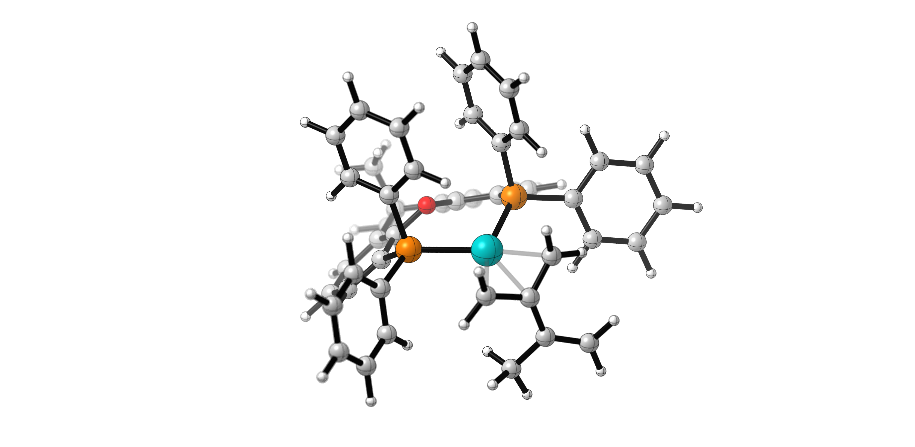 | E/hartree | -2625.512214 |
| --- | --- | --- |
|  | E+zvp/hartree | -2624.778984 |
|  | G/hartree | -2624.858061 |
|  | E(_SP)_/hartree | -2626.404204 |

Pd 0.1618296 -1.7248027 -0.0943538

C -0.4384214 -3.6417017 -3.1612718

C -0.8390684 -3.6693427 -0.2063028

C 0.6748916 -4.0267917 -2.2198868

C 0.4575936 -3.8311127 -0.7614698

H -1.7234944 -3.7702457 -0.8197068

H -0.9902844 -3.8647037 0.8524762

C 1.8261776 -4.5571007 -2.6561778

C 1.5175466 -3.4645147 0.1009662

H -0.7136144 -2.5888007 -3.0295458

H -1.3401004 -4.2384987 -2.9850548

H 2.6120936 -4.8685817 -1.9768838

H 1.9994686 -4.7239467 -3.7143558

H 1.4155896 -3.6394747 1.1696062

H 2.5282526 -3.3805297 -0.2727888

H -0.1365394 -3.7917997 -4.1993238

P 1.9164686 -0.1486037 0.2573202

C 2.0412816 1.4578073 -0.6225338

C 3.5595046 -0.8634077 -0.1229648

C 1.9580446 0.2523063 2.0397832

C 3.2364156 1.9297173 -1.1875598

C 0.8974806 2.2375363 -0.7919628

C 4.6578206 -0.7422177 0.7356752

C 3.7214226 -1.4959887 -1.3664358

C 1.6951716 1.5401123 2.5179412

C 2.1958416 -0.7887797 2.9524652

C 3.2414806 3.1179603 -1.9145568

H 4.1513376 1.3622203 -1.0737718

C 0.8621136 3.4232913 -1.5296078

O -0.2533374 1.7704363 -0.1937838

C 5.8989076 -1.2630077 0.3607582

H 4.5490536 -0.2426517 1.6916852

C 4.9623266 -2.0051487 -1.7405658

H 2.8714076 -1.5998437 -2.0352998

C 1.6750246 1.7841863 3.8917672

H 1.5108416 2.3522543 1.8247702

C 2.1949126 -0.5362147 4.3215952

H 2.4017486 -1.7912267 2.5892242

C 2.0652106 3.8500573 -2.0962608

H 4.1680676 3.4734953 -2.3525488

C -0.4732754 4.1707923 -1.6103008

C -1.4049374 1.9507223 -0.9312078

C 6.0534366 -1.8930977 -0.8738368

H 6.7448916 -1.1703207 1.0343692

H 5.0771716 -2.4932647 -2.7032318

C 1.9298856 0.7520933 4.7937732

H 1.4662526 2.7852843 4.2549412

H 2.3988356 -1.3424867 5.0193802

H 2.0938176 4.7620773 -2.6808888

C -1.5772224 3.1114783 -1.6843908

C -0.5212544 5.1366593 -2.8001038

C -0.6623564 4.9711203 -0.2927988

C -2.3370304 0.9154433 -0.8644768

H 7.0193876 -2.2955177 -1.1615838

H 1.9231676 0.9484843 5.8611672

C -2.7748344 3.2267923 -2.3961838

H 0.2572536 5.8978593 -2.7102558

H -0.3890924 4.6135453 -3.7512808

H -1.4751714 5.6686233 -2.8250398

H -1.6230684 5.4942373 -0.3048538

H -0.6436354 4.3092443 0.5771402

H 0.1371306 5.7096023 -0.1824728

C -3.5404384 1.0773863 -1.5643778

P -1.8872814 -0.5368997 0.1562722

C -3.7480644 2.2264323 -2.3274258

H -2.9610664 4.1058413 -3.0020718

H -4.3031984 0.3100433 -1.5199828

C -3.3610114 -1.6097597 -0.0189408

C -1.9607034 0.0878503 1.8709722

H -4.6777054 2.3448033 -2.8739038

C -3.6355994 -2.1788237 -1.2744638

C -4.1953884 -1.9038867 1.0645352

C -2.7010174 1.2293403 2.2054962

C -1.2756804 -0.6113137 2.8739212

C -4.7323684 -3.0201157 -1.4421758

H -2.9916314 -1.9580367 -2.1212398

C -5.2876704 -2.7588827 0.8949362

H -3.9980294 -1.4664957 2.0366682

C -2.7513674 1.6636483 3.5294802

H -3.2346444 1.7756123 1.4350592

C -1.3378554 -0.1808897 4.1974142

H -0.6821144 -1.4823397 2.6141612

C -5.5590624 -3.3155377 -0.3537658

H -4.9414034 -3.4474127 -2.4179208

H -5.9286044 -2.9830847 1.7416722

C -2.0725114 0.9592913 4.5255942

H -3.3235184 2.5504823 3.7831962

H -0.7975544 -0.7230747 4.9650752

H -6.4103464 -3.9761647 -0.4823298

H -2.1139814 1.3002713 5.5552202

#### Int-21

| 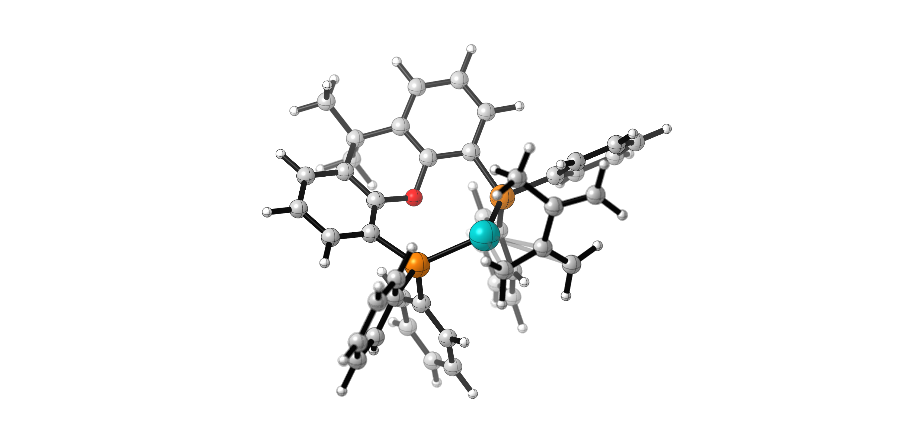 | E/hartree | -2625.647180 |
| --- | --- | --- |
|  | E+zvp/hartree | -2624.919022 |
|  | G/hartree | -2625.000578 |
|  | E(_SP)_/hartree | -2626.517589 |

C -3.7683167 -1.0643877 -1.2464254

C -2.5049977 -0.8875077 -0.6616984

C -1.6132877 -1.9621037 -0.7428004

C -1.8894777 -3.1589047 -1.4106694

C -3.1501017 -3.2856497 -1.9985384

C -4.0845027 -2.2517597 -1.9042514

C 0.5473013 -3.5271847 -1.5093174

C 0.6904953 -2.3252177 -0.8155974

C 1.8771833 -1.5898237 -0.7590334

C 2.9863843 -2.1075877 -1.4394574

C 2.8767553 -3.3005437 -2.1556314

C 1.6701233 -4.0035107 -2.1932564

H -4.4951197 -0.2624097 -1.2031324

H -3.4116547 -4.1933417 -2.5303964

H -5.0645037 -2.3702817 -2.3559414

H 3.9273073 -1.5697647 -1.4148394

H 3.7401603 -3.6888567 -2.6868764

H 1.6108253 -4.9293137 -2.7542624

C -0.8038307 -4.2402427 -1.4002204

C -1.0045707 -5.2686077 -2.5191314

H -1.9615467 -5.7837127 -2.4030394

H -0.9799447 -4.7986627 -3.5064324

H -0.2268507 -6.0355567 -2.4799444

C -0.8538097 -4.9654487 -0.0280774

H -0.0676097 -5.7251567 0.0262116

H -0.7050387 -4.2589787 0.7925116

H -1.8245827 -5.4527067 0.1074046

O -0.3875747 -1.7972417 -0.1340954

P -1.9126707 0.7156973 0.0412126

P 1.8292573 0.0250893 0.1199406

Pd 0.1936123 1.6369243 -0.4441444

C -3.3548657 1.7859713 -0.3593554

C -4.4438847 1.9792643 0.5004156

C -3.3667467 2.3999183 -1.6207214

C -5.5179517 2.7804293 0.1089276

H -4.4529737 1.5030663 1.4746346

C -4.4455237 3.1881913 -2.0164464

H -2.5202997 2.2563143 -2.2849774

C -5.5221597 3.3851083 -1.1487944

H -6.3542937 2.9271013 0.7862576

H -4.4420227 3.6564783 -2.9962964

H -6.3587207 4.0080663 -1.4510234

C -2.0634687 0.4932293 1.8560436

C -2.1844277 -0.7589317 2.4680966

C -1.9801667 1.6410413 2.6627496

C -2.2337677 -0.8609637 3.8588816

H -2.2442927 -1.6550337 1.8614336

C -2.0551587 1.5382243 4.0487736

H -1.8666987 2.6153203 2.1962516

C -2.1802887 0.2842263 4.6521366

H -2.3180727 -1.8398257 4.3212366

H -2.0059547 2.4354713 4.6590836

H -2.2263327 0.2014523 5.7338426

C 3.6028713 0.5118023 0.0589186

C 4.5660823 -0.0037627 0.9376946

C 3.9947203 1.4315283 -0.9215044

C 5.8984903 0.3948863 0.8312816

H 4.2719373 -0.7102237 1.7069366

C 5.3277473 1.8268033 -1.0295274

H 3.2513583 1.8618823 -1.5816154

C 6.2822743 1.3092893 -0.1527874

H 6.6373883 -0.0063097 1.5190096

H 5.6108483 2.5507823 -1.7877724

H 7.3196783 1.6218053 -0.2290764

C 1.6333933 -0.4883757 1.8751466

C 1.8018763 -1.8056777 2.3181666

C 1.3087123 0.5094993 2.8047576

C 1.6502313 -2.1191077 3.6697866

H 2.0516523 -2.5870197 1.6079736

C 1.1724513 0.1972833 4.1549486

H 1.1498923 1.5259093 2.4571776

C 1.3393243 -1.1184597 4.5903696

H 1.7780103 -3.1454237 4.0021966

H 0.9100833 0.9763973 4.8627176

H 1.2187393 -1.3633067 5.6415306

C -0.3747607 3.7149463 -0.8231564

H -0.9422967 3.8745473 -1.7325194

H -0.8851437 4.0484073 0.0781736

C 1.1053903 3.1106443 -3.3439604

H 0.9335603 2.0556183 -3.0973534

H 0.1271813 3.5365893 -3.5935654

H 1.7344323 3.1713553 -4.2358904

C 1.7451463 3.8341353 -2.1818974

C 1.0512013 3.8005573 -0.8564384

C 1.7989833 3.9470693 0.3300926

H 2.8764903 3.8490953 0.3344506

H 1.2930183 4.0722783 1.2812036

C 2.8982523 4.5006343 -2.3478734

H 3.3580383 5.0564333 -1.5384484

H 3.4013993 4.5201243 -3.3103664

#### TS-14

| 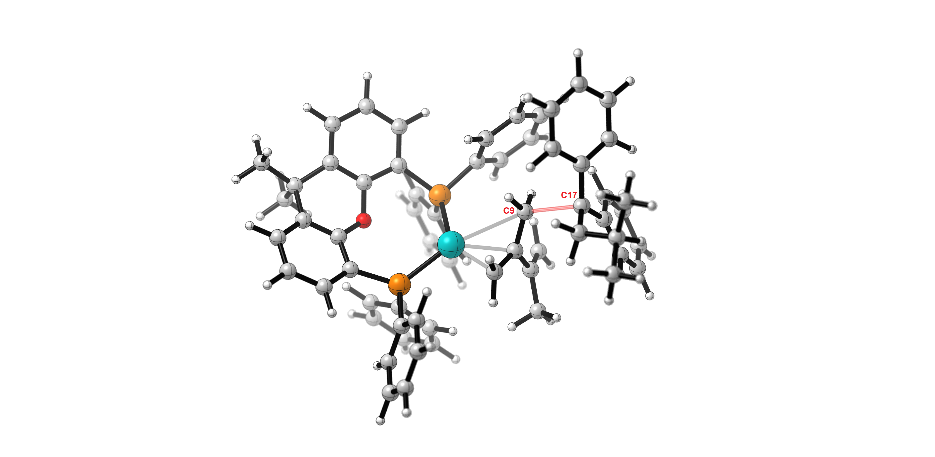  TS with fixed distances between C9-C17 | E/hartree | -3285.020083 |
| --- | --- | --- |
|  | E+zvp/hartree | -3283.975771 |
|  | G/hartree | -3284.074350 |
|  | E(_SP)_/hartree | -3286.162829 |
|  | Imaginary frequency/cm^-1^ | -104.01 |

Pd -0.6133113 0.5556650 -0.1786925

C 1.6666317 2.7550900 2.2474905

C 0.9590337 2.0744270 -0.5292585

C 1.6960107 1.3263400 1.7607985

C 1.5708707 1.0984020 0.2850295

H 0.6877127 3.0483000 -0.1413105

H 1.1179757 2.0371350 -1.6040985

C 1.8509547 0.3228070 2.6359325

C 2.2569377 -0.0252460 -0.3297015

H 0.7069007 3.2394190 2.0343615

H 2.4418687 3.3513580 1.7543295

H 1.8741117 -0.7149600 2.3307925

H 1.9827267 0.5207640 3.6942775

H 1.9903767 -0.2388270 -1.3582695

H 2.4105757 -0.9097180 0.2687505

H 1.8327337 2.7988450 3.3264495

C 4.3937287 0.3450890 -0.6496875

C 4.8157747 0.4059510 0.7745975

C 5.1111327 1.6262020 1.4113545

C 4.7995127 -0.7478770 1.5900135

C 5.3738747 1.6920930 2.7799975

H 5.1220227 2.5465600 0.8412135

C 5.0658887 -0.6832920 2.9511125

H 4.5180917 -1.7003810 1.1571305

C 5.3557547 0.5404520 3.5623725

H 5.5916027 2.6554140 3.2331835

H 5.0252897 -1.5927200 3.5447055

H 5.5592147 0.5917480 4.6277725

C 4.7429927 -0.8625840 -1.4584305

C 5.7640247 -1.7598720 -1.0914005

C 4.0708587 -1.1304150 -2.6694015

C 6.0645847 -2.8813170 -1.8636675

H 6.3412987 -1.5674670 -0.1953855

C 4.3712717 -2.2455220 -3.4451505

H 3.2980727 -0.4513380 -3.0119975

C 5.3650627 -3.1412100 -3.0423155

H 6.8616297 -3.5479390 -1.5456515

H 3.8252147 -2.4167880 -4.3688285

H 5.5990447 -4.0142960 -3.6438475

C 4.3644267 1.6565850 -1.4163645

H 3.7990197 2.3882340 -0.8286885

H 3.7892107 1.5250680 -2.3402735

C 5.7354337 2.2691310 -1.8003425

H 6.3865647 2.2374090 -0.9179695

C 6.4377407 1.4959580 -2.9239325

H 6.6620387 0.4676370 -2.6355105

H 5.8085857 1.4575700 -3.8215455

H 7.3791547 1.9857550 -3.1970585

C 5.5440077 3.7382030 -2.1981145

H 5.1011267 4.3202980 -1.3820795

H 6.4964247 4.2061380 -2.4694235

H 4.8749657 3.8204010 -3.0638745

P -2.6190593 1.7275070 -0.3341585

C -4.0925723 1.1643090 -1.2893285

C -2.5289923 3.4919180 -0.8487355

C -3.2403273 1.8614130 1.3919415

C -4.8281213 1.9851530 -2.1565335

C -4.4386823 -0.1907460 -1.2472865

C -3.3044203 4.5019610 -0.2620025

C -1.6556813 3.8234690 -1.8938655

C -4.4323443 1.2788840 1.8338115

C -2.4236203 2.5276920 2.3208565

C -5.8395773 1.4477250 -2.9512675

H -4.5947303 3.0413080 -2.2196305

C -5.4441503 -0.7624650 -2.0313215

O -3.7216043 -0.9877970 -0.3783625

C -3.2028263 5.8188080 -0.7110175

H -3.9851643 4.2559770 0.5461805

C -1.5625183 5.1381160 -2.3499465

H -1.0441433 3.0434990 -2.3367235

C -4.7996143 1.3584840 3.1785325

H -5.0781423 0.7644800 1.1318955

C -2.8011573 2.6241780 3.6566015

H -1.4900113 2.9703830 1.9884845

C -6.1413243 0.0853850 -2.8957885

H -6.3960183 2.0944900 -3.6224885

C -5.7272753 -2.2543240 -1.8339515

C -3.4324143 -2.2573820 -0.8347275

C -2.3333423 6.1390640 -1.7558545

H -3.8045303 6.5947030 -0.2466345

H -0.8819313 5.3824560 -3.1601745

C -3.9908553 2.0334060 4.0913155

H -5.7233723 0.8935290 3.5098395

H -2.1623213 3.1502090 4.3601905

H -6.9270513 -0.3122400 -3.5279145

C -4.3805853 -2.9440480 -1.5973635

C -6.4845113 -2.8587740 -3.0220425

C -6.5843043 -2.4192090 -0.5495515

C -2.1801833 -2.7791280 -0.4934885

H -2.2543263 7.1651550 -2.1025355

H -4.2815983 2.0963690 5.1356205

C -4.0448093 -4.2297350 -2.0298705

H -7.4509403 -2.3656650 -3.1539095

H -5.9161863 -2.7646430 -3.9517625

H -6.6927643 -3.9173650 -2.8476075

H -6.7801693 -3.4793130 -0.3601125

H -6.0678763 -2.0061140 0.3205055

H -7.5405513 -1.8988960 -0.6636835

C -1.8934563 -4.0826440 -0.9210715

P -1.0251103 -1.6991070 0.4582085

C -2.8155273 -4.7957830 -1.6871855

H -4.7455113 -4.8005060 -2.6285285

H -0.9412563 -4.5313270 -0.6645515

C 0.4735757 -2.7628300 0.5591105

C -1.7382313 -1.8011260 2.1484145

H -2.5750953 -5.8011750 -2.0185785

C 1.0145777 -3.1788970 1.7823225

C 1.1964867 -3.0137510 -0.6206765

C -2.5152083 -2.8871410 2.5741525

C -1.4856463 -0.7465480 3.0354365

C 2.2595497 -3.8123370 1.8247335

H 0.4713737 -2.9936450 2.7024655

C 2.4307307 -3.6567740 -0.5798055

H 0.8041177 -2.6683320 -1.5730525

C -3.0337253 -2.9147510 3.8682035

H -2.7164853 -3.7069220 1.8920965

C -1.9963663 -0.7822820 4.3322765

H -0.8919593 0.0975470 2.7030035

C 2.9702217 -4.0500960 0.6480825

H 2.6736907 -4.1150290 2.7821575

H 2.9916617 -3.8122610 -1.4951135

C -2.7741513 -1.8627080 4.7493345

H -3.6396723 -3.7573680 4.1888015

H -1.8027033 0.0452750 5.0062365

H 3.9453177 -4.5260800 0.6818785

H -3.1812543 -1.8842690 5.7561245

#### Int-22

| 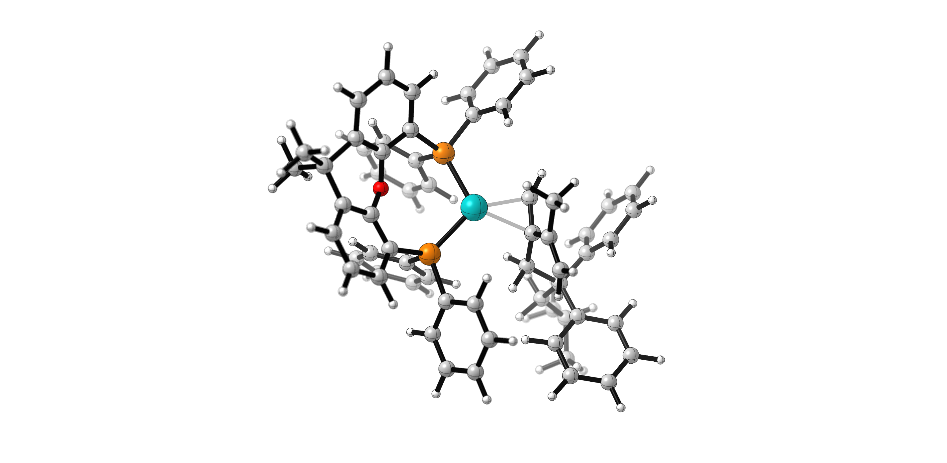 | E/hartree | -3285.060169 |
| --- | --- | --- |
|  | E+zvp/hartree | -3284.011963 |
|  | G/hartree | -3284.110401 |
|  | E(_SP)_/hartree | -3286.201076 |

Pd -0.6384434 0.4374850 0.7469534

C 0.9213086 1.0218430 3.7857454

C 0.9409046 1.9217430 1.0387174

C 1.7286306 0.2773410 2.7435284

C 1.5768696 0.7019930 1.3338364

H 0.6662606 2.6216430 1.8176794

H 1.1143826 2.3852960 0.0716754

C 2.5571886 -0.7062450 3.1426954

C 2.3492276 0.0203760 0.2117914

H -0.1453274 1.0203050 3.5337404

H 1.2364926 2.0696220 3.8564934

H 3.1812136 -1.2623440 2.4595414

H 2.6276326 -0.9741460 4.1930224

H 1.8277546 0.2989960 -0.7062056

H 2.2444666 -1.0604030 0.3025944

H 1.0463896 0.5656320 4.7705964

C 3.8743026 0.3626330 -0.0625496

C 4.1141186 1.8190890 0.3426304

C 4.1127516 2.8683550 -0.5836056

C 4.2220616 2.1568950 1.7025074

C 4.2176316 4.2006740 -0.1752286

H 4.0203276 2.6625650 -1.6421006

C 4.3293236 3.4819100 2.1150424

H 4.1990096 1.3697870 2.4464424

C 4.3272086 4.5158180 1.1766044

H 4.2134096 4.9896130 -0.9219456

H 4.4075946 3.7069470 3.1750144

H 4.4087846 5.5505550 1.4960524

C 4.8353726 -0.6200690 0.6268374

C 6.0607096 -0.2131470 1.1694134

C 4.5682056 -1.9993460 0.5953104

C 6.9684766 -1.1392020 1.6871504

H 6.3154216 0.8392730 1.1847464

C 5.4669556 -2.9262570 1.1168744

H 3.6508136 -2.3713070 0.1541684

C 6.6755586 -2.5015340 1.6708794

H 7.9095796 -0.7887360 2.1013344

H 5.2181706 -3.9827690 1.0829994

H 7.3794866 -3.2218870 2.0767994

C 4.0564546 0.1373490 -1.5984706

H 3.3487136 0.7952920 -2.1147896

H 3.7171546 -0.8821740 -1.8197576

C 5.4718086 0.3394380 -2.1929436

H 6.0174156 1.0417160 -1.5504766

C 6.2817476 -0.9634630 -2.2723716

H 6.4327606 -1.4198270 -1.2942496

H 5.7667566 -1.6935870 -2.9092146

H 7.2665426 -0.7760010 -2.7145736

C 5.3699666 0.9630420 -3.5928186

H 4.8659636 1.9353050 -3.5702476

H 6.3612416 1.1100980 -4.0344966

H 4.8007016 0.3102060 -4.2662076

P -1.0585014 -1.7770860 0.0621534

C -2.5710224 -2.7222890 0.5442734

C 0.2428096 -3.0410780 0.3824644

C -1.1210554 -1.7515690 -1.7787006

C -2.5488124 -4.0364750 1.0326694

C -3.8029324 -2.0597850 0.5586444

C 0.6654476 -3.9894990 -0.5608946

C 0.8339056 -3.0360600 1.6536464

C -2.2934724 -1.9685530 -2.5103276

C 0.0589496 -1.4298010 -2.4706796

C -3.7084884 -4.6241020 1.5363764

H -1.6170834 -4.5895160 1.0363154

C -4.9813904 -2.6125460 1.0672154

O -3.8249524 -0.7795880 0.0434174

C 1.6636086 -4.9091610 -0.2370106

H 0.2167386 -4.0063340 -1.5479076

C 1.8236506 -3.9628810 1.9799264

H 0.5440846 -2.2787480 2.3731154

C -2.2859054 -1.8695960 -3.9025956

H -3.2152034 -2.2164770 -1.9985266

C 0.0691066 -1.3511910 -3.8604536

H 0.9751396 -1.2571160 -1.9171176

C -4.9118964 -3.9161600 1.5650124

H -3.6737294 -5.6400940 1.9172314

C -6.2525264 -1.7607860 0.9880934

C -4.6267984 0.1199960 0.7147464

C 2.2413416 -4.9011480 1.0349404

H 1.9866506 -5.6356270 -0.9770296

H 2.2842196 -3.9320810 2.9621284

C -1.1069634 -1.5681530 -4.5822826

H -3.2066444 -2.0307280 -4.4551636

H 0.9943606 -1.1150240 -4.3785026

H -5.7979044 -4.3883000 1.9739964

C -5.8438574 -0.3075690 1.2481704

C -7.3283854 -2.2409790 1.9690474

C -6.8081254 -1.8516580 -0.4590046

C -4.1435634 1.4281270 0.8182654

H 3.0177936 -5.6182910 1.2847134

H -1.1041504 -1.4971670 -5.6658256

C -6.6132074 0.6450010 1.9230744

H -7.6221294 -3.2698090 1.7459904

H -6.9777984 -2.1959730 3.0040534

H -8.2301354 -1.6297760 1.8805834

H -7.7027864 -1.2290780 -0.5599216

H -6.0670664 -1.5059400 -1.1841676

H -7.0700814 -2.8868630 -0.6995356

C -4.9529384 2.3591960 1.4822984

P -2.4593234 1.7697530 0.1529784

C -6.1730154 1.9658200 2.0336494

H -7.5640824 0.3625900 2.3605644

H -4.6189404 3.3859360 1.5774364

C -2.2973034 3.5621340 0.5347054

C -2.7478844 1.7586160 -1.6609556

H -6.7876694 2.6947040 2.5527384

C -2.5998394 4.5779980 -0.3802066

C -1.8349074 3.9121580 1.8132114

C -4.0217144 1.8559150 -2.2348126

C -1.6273604 1.6505940 -2.4967486

C -2.4396274 5.9184310 -0.0232626

H -2.9596624 4.3212780 -1.3707246

C -1.6911524 5.2501540 2.1738904

H -1.5832904 3.1262400 2.5196444

C -4.1708154 1.8481080 -3.6223726

H -4.8956454 1.9381740 -1.5968116

C -1.7768804 1.6593050 -3.8813256

H -0.6442054 1.5370280 -2.0508576

C -1.9887314 6.2575810 1.2526244

H -2.6709504 6.6978750 -0.7435316

H -1.3355394 5.5069460 3.1674124

C -3.0493764 1.7546700 -4.4472686

H -5.1634844 1.9184930 -4.0579366

H -0.9028754 1.5636210 -4.5167016

H -1.8646954 7.3007980 1.5275094

H -3.1667764 1.7470190 -5.5269266

#### TS-15

| 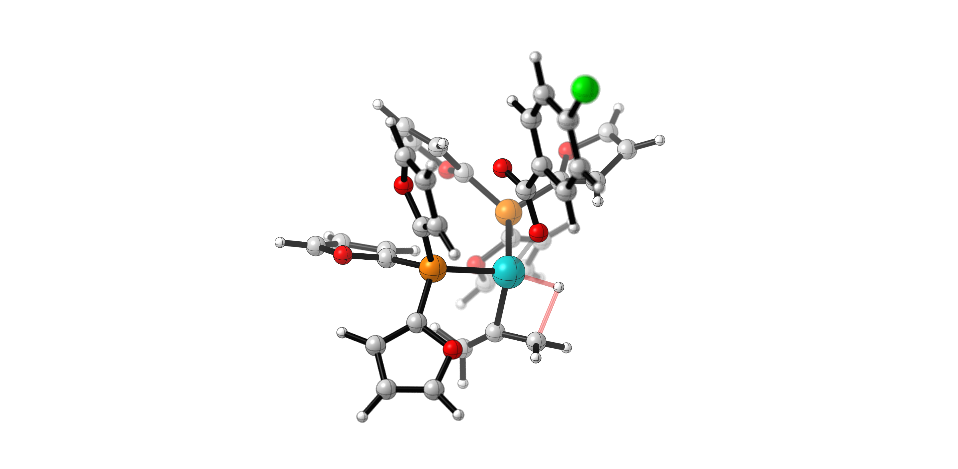 | E/hartree | -3184.518078 |
| --- | --- | --- |
|  | E+zvp/hartree | -3183.988603 |
|  | G/hartree | -3184.073899 |
|  | E(_SP)_/hartree | -3185.534299 |
|  | Imaginary frequency/cm^-1^ | -761.05 |

P 1.2655000 -1.7625763 -0.1531121

P 0.2177100 1.9211787 0.0134209

Pd 0.4213620 0.1206407 -1.5404521

H 0.4454630 -0.7206943 -2.8731041

C 1.1993780 0.6947197 -3.5445351

H 1.5221460 -0.0510733 -4.2668401

H 0.5061830 1.4417837 -3.9217311

C 2.0353700 0.9986417 -2.4507361

C 3.1527930 1.5824777 -2.0593581

H 3.4603910 1.6020527 -1.0219131

H 3.8009650 2.0702037 -2.7854631

O -1.7001900 -0.3433683 -1.4721111

C -2.1943120 -0.7648133 -0.3590241

O -1.5461410 -1.1009283 0.6462989

C -3.6994680 -0.8319603 -0.3127471

C -4.4801760 -0.3662913 -1.3749161

C -4.3262470 -1.3262753 0.8352359

C -5.8714100 -0.3844073 -1.2948881

H -3.9808910 0.0161947 -2.2577131

C -5.7146660 -1.3557123 0.9314039

H -3.7002750 -1.6736713 1.6490339

C -6.4728690 -0.8785523 -0.1380531

H -6.4846700 -0.0214273 -2.1120761

H -6.2081040 -1.7367663 1.8185109

Cl -8.2297200 -0.8988393 -0.0231241

C 1.4674650 2.1116167 1.2909429

C 2.5860720 1.3800857 1.5857299

O 1.3342830 3.1365377 2.2038939

C 3.1757850 1.9825317 2.7414659

H 2.9441640 0.5118077 1.0555669

C 2.3774490 3.0370777 3.0708799

H 4.0668780 1.6600757 3.2589539

H 2.4058050 3.7759167 3.8567829

C 0.2576500 3.5208767 -0.8358441

C 0.6440780 4.7853527 -0.4861211

O -0.1620190 3.5162037 -2.1468681

C 0.4552490 5.6074787 -1.6424781

H 1.0118150 5.0805197 0.4846009

C -0.0369520 4.7882917 -2.6145001

H 0.6589380 6.6643727 -1.7345521

H -0.3370140 4.9430697 -3.6390551

C -1.3338220 2.0107817 0.9385459

C -2.6004090 2.3412737 0.5415499

O -1.3592450 1.5100427 2.2116829

C -3.4626160 2.0241247 1.6394519

H -2.8819720 2.7359147 -0.4230601

C -2.6603530 1.5207567 2.6186049

H -4.5358430 2.1357857 1.6786009

H -2.8411400 1.1508417 3.6151989

C 1.4145810 -1.7199183 1.6559689

C 0.5865760 -1.2596523 2.6449419

O 2.6319760 -2.0731493 2.2026279

C 1.3260920 -1.3418593 3.8659949

H -0.4189030 -0.9074013 2.4869909

C 2.5551720 -1.8343413 3.5391219

H 0.9870740 -1.0646663 4.8537529

H 3.4417980 -2.0680803 4.1081189

C 2.9820350 -2.1487773 -0.6053941

C 3.5981340 -3.1938213 -1.2342651

O 3.8915780 -1.1366933 -0.4045161

C 4.9663570 -2.8078263 -1.4292331

H 3.1329750 -4.1290453 -1.5046421

C 5.0890510 -1.5579853 -0.9047771

H 5.7498220 -3.3905573 -1.8915991

H 5.9092320 -0.8645723 -0.8066301

C 0.4630690 -3.3195523 -0.5678791

C -0.6031790 -3.5619793 -1.3908891

O 0.8982450 -4.5039383 -0.0145191

C -0.8388680 -4.9728953 -1.3503101

H -1.1575200 -2.8057983 -1.9271281

C 0.0956720 -5.4889123 -0.5015451

H -1.6064560 -5.5238543 -1.8738471

H 0.3131170 -6.4865943 -0.1523241

#### Int-23

| 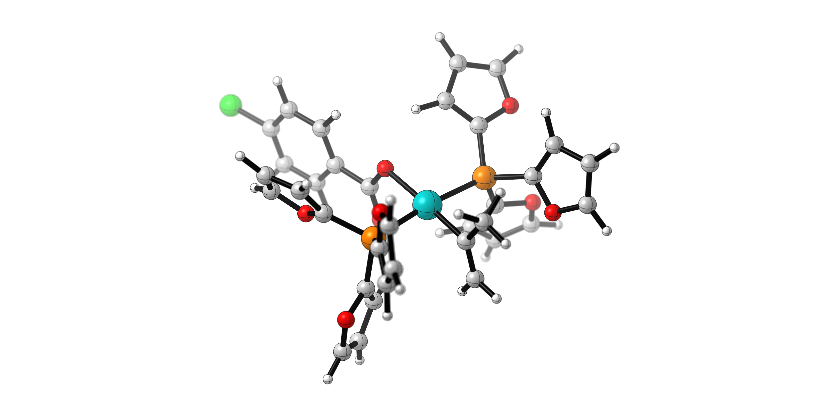 | E/hartree | -3184.598267 |
| --- | --- | --- |
|  | E+zvp/hartree | -3184.062758 |
|  | G/hartree | -3184.150000 |
|  | E(_SP)_/hartree | -3185.611144 |

P 1.9337166 -1.7111989 -0.0939525

P -0.5455944 2.2270981 -0.0905535

Pd 0.6009896 0.2073831 -0.1546235

H 3.3733236 0.7868691 -1.6364125

C 3.0700226 1.6736461 -1.0700145

H 3.9763866 2.2298161 -0.7971645

H 2.4790476 2.2945131 -1.7497055

C 2.2779076 1.2983571 0.1523435

C 2.6356586 1.6478841 1.3904985

H 2.0565186 1.3747981 2.2670225

H 3.5472366 2.2216221 1.5685735

O -1.2216334 -0.8599599 -0.5073585

C -1.9291494 -1.0724379 0.5496255

O -1.5643164 -0.8646629 1.7213735

C -3.3205874 -1.5863989 0.2881725

C -3.7864724 -1.7868879 -1.0157085

C -4.1821524 -1.8119629 1.3658845

C -5.0932054 -2.2106099 -1.2467095

H -3.1193784 -1.5836959 -1.8449185

C -5.4930924 -2.2325259 1.1538425

H -3.8047114 -1.6405289 2.3675955

C -5.9341064 -2.4274139 -0.1552735

H -5.4621634 -2.3637749 -2.2546115

H -6.1673674 -2.4065279 1.9848095

Cl -7.5901434 -2.9516629 -0.4366395

C -0.7483894 2.7933281 1.5985555

C -0.8318704 2.0486711 2.7428755

O -0.9719044 4.1225351 1.8828775

C -1.1116654 2.9704821 3.8009695

H -0.7550734 0.9711361 2.7809775

C -1.1796254 4.2050421 3.2258505

H -1.2441134 2.7413701 4.8481315

H -1.3537204 5.1978241 3.6112175

C 0.2125736 3.6202641 -0.9477495

C 0.8419216 4.7618181 -0.5409175

O 0.3207326 3.4917071 -2.3137155

C 1.3640566 5.3813881 -1.7229135

H 0.9137036 5.1119761 0.4761525

C 1.0180716 4.5752741 -2.7641955

H 1.9183576 6.3064511 -1.7825775

H 1.1731516 4.6208511 -3.8304665

C -2.2185434 2.2216961 -0.7845295

C -2.7627104 2.6575781 -1.9621775

O -3.1606044 1.5349311 -0.0658465

C -4.1251314 2.2123231 -1.9645695

H -2.2494124 3.2192761 -2.7271395

C -4.3094684 1.5367781 -0.7951525

H -4.8635574 2.3772841 -2.7353785

H -5.1427524 1.0153061 -0.3521755

C 1.9993566 -2.4017169 1.5630715

C 1.1266656 -2.2713149 2.6105795

O 3.0274946 -3.2495479 1.9055035

C 1.6486266 -3.0862229 3.6647695

H 0.2130816 -1.6903059 2.5869555

C 2.7961726 -3.6469339 3.1842135

H 1.2258726 -3.2306329 4.6483095

H 3.5316376 -4.3140509 3.6069125

C 3.6696866 -1.6010049 -0.5770585

C 4.3323746 -1.9564369 -1.7216345

O 4.5226506 -0.9388279 0.2640895

C 5.6755676 -1.4833839 -1.5745135

H 3.9125306 -2.4982909 -2.5549535

C 5.7325196 -0.8774369 -0.3544095

H 6.4862026 -1.5888249 -2.2804335

H 6.5168196 -0.3873349 0.2004345

C 1.3563326 -3.0216489 -1.1849085

C 0.2090836 -3.1263929 -1.9260415

O 2.0967146 -4.1787089 -1.3076975

C 0.2442276 -4.4151509 -2.5463225

H -0.5652444 -2.3747339 -1.9649515

C 1.4045206 -5.0037519 -2.1367065

H -0.4975794 -4.8445689 -3.2036265

H 1.8639226 -5.9598029 -2.3357835

#### Int-24

| 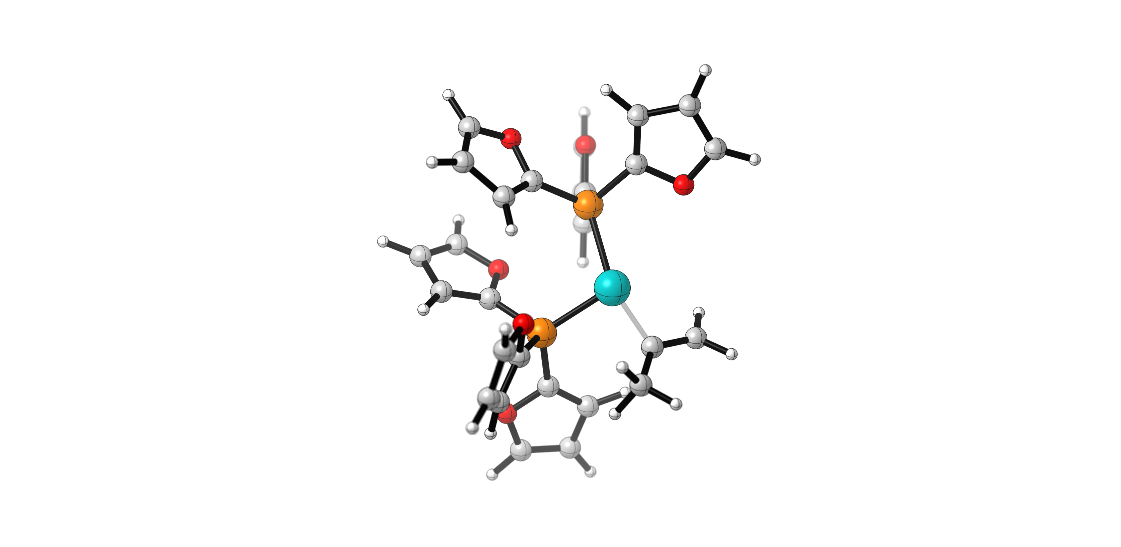 | E/hartree | -2304.516015 |
| --- | --- | --- |
|  | E+zvp/hartree | -2304.076481 |
|  | G/hartree | -2304.151268 |
|  | E(_SP)_/hartree | -2305.315398 |

P 2.0034890 0.2591593 0.0521791

P -1.6646930 -0.1295557 0.1422811

Pd -0.0768870 1.4541853 0.0986401

C -2.6355670 3.0638073 -0.8306369

H -2.4482030 2.6921253 -1.8416569

H -3.4709510 2.4857173 -0.4219009

C -1.4328010 2.9251953 0.0374911

C -0.8169840 3.8571623 0.7701071

H -0.0023670 3.6231523 1.4628961

H -1.0619040 4.9174613 0.6850621

C 2.0990580 -1.1277707 -1.0802729

C 1.3017530 -1.4760417 -2.1384529

O 3.1455580 -2.0131117 -0.9759019

C 1.8873790 -2.6445587 -2.7192169

H 0.4068060 -0.9627137 -2.4578789

C 2.9954170 -2.9249337 -1.9749119

H 1.5286540 -3.2009637 -3.5724019

H 3.7447400 -3.7000517 -2.0186299

C 2.6519190 -0.3006647 1.6282461

C 2.0630720 -0.4248637 2.8604521

O 3.9788220 -0.6440267 1.7147251

C 3.0814520 -0.8789857 3.7543921

H 1.0258450 -0.2296937 3.0875681

C 4.2173660 -0.9927067 3.0054861

H 2.9829190 -1.0855747 4.8097321

H 5.2298350 -1.2889507 3.2324401

C 3.2292260 1.4137713 -0.5798989

C 4.3670330 1.3098513 -1.3304759

O 2.9936580 2.7380633 -0.2731249

C 4.8680540 2.6397093 -1.4953179

H 4.7933620 0.3919273 -1.7070839

C 4.0025720 3.4613783 -0.8360679

H 5.7558670 2.9405813 -2.0316649

H 3.9599790 4.5278453 -0.6801149

C -1.0232000 -1.7950577 0.2638941

C -1.2336340 -2.9415337 -0.4563689

O -0.0266050 -1.9994597 1.1821561

C -0.3124070 -3.9091197 0.0516501

H -1.9546720 -3.0709727 -1.2495659

C 0.3962510 -3.2849187 1.0367561

H -0.1925190 -4.9300307 -0.2785979

H 1.1909890 -3.5909587 1.6984801

C -2.8324290 0.0873543 1.4786481

C -2.9906770 1.0720033 2.4196801

O -3.7709550 -0.8996177 1.6757441

C -4.0930950 0.6758453 3.2378761

H -2.3939040 1.9678343 2.5027621

C -4.5243510 -0.5206017 2.7435361

H -4.5041360 1.2087343 4.0822881

H -5.3137300 -1.2004637 3.0249671

C -2.6593270 -0.1950537 -1.3466659

C -3.9964890 -0.3664347 -1.5831479

O -1.9974810 0.0091923 -2.5352709

C -4.1724320 -0.2670877 -2.9994719

H -4.7533210 -0.5471557 -0.8346649

C -2.9346700 -0.0402597 -3.5248779

H -5.0983250 -0.3523197 -3.5486939

H -2.5645420 0.1012223 -4.5282689

H -2.9478190 4.1129353 -0.8889279

#### Int-25

| 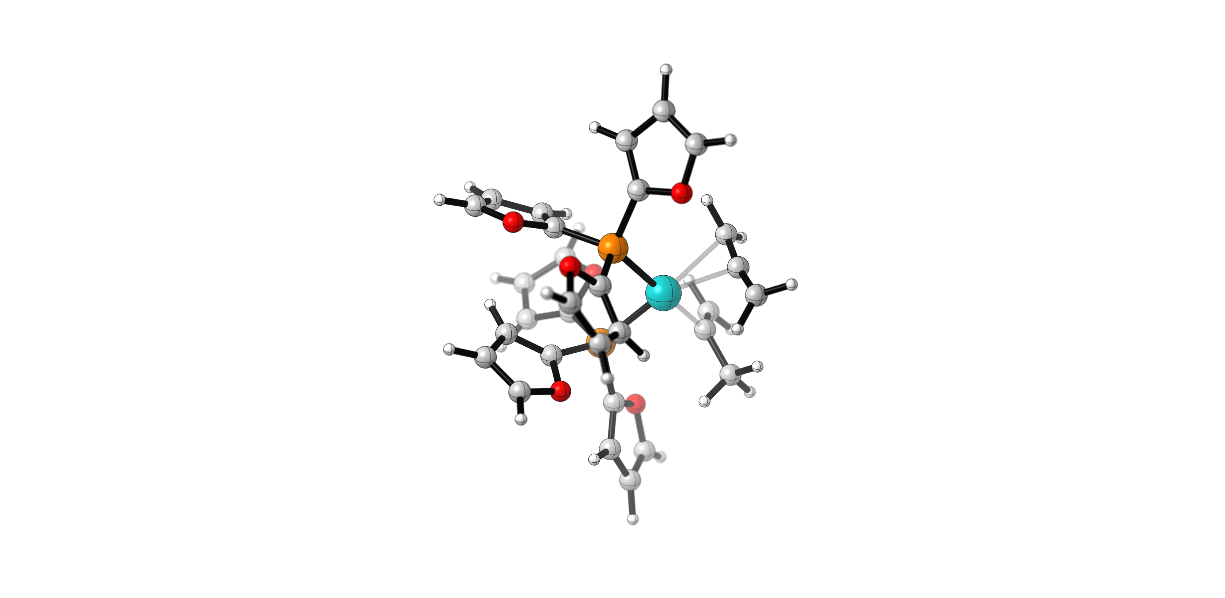 | E/hartree | -2421.223730 |
| --- | --- | --- |
|  | E+zvp/hartree | -2420.724905 |
|  | G/hartree | -2420.800466 |
|  | E(_SP)_/hartree | -2422.069483 |

P 1.8378150 -0.0098469 -0.1138852

P -1.6793550 0.3106881 0.0427248

Pd -0.1477800 -1.4002059 -0.3020392

C -2.3015830 -3.2042229 0.8132728

H -1.5529300 -3.8829279 1.2329518

H -2.5172230 -2.4450499 1.5731488

C -1.8195590 -2.5820279 -0.4646602

C -2.3701670 -2.7754659 -1.6622852

H -2.0161020 -2.2821289 -2.5616242

H -3.2315110 -3.4353369 -1.7761742

C 3.3874260 -0.7098219 -0.7232842

C 4.1670240 -0.4409589 -1.8164302

O 3.8842800 -1.8006569 -0.0545832

C 5.2148380 -1.4176709 -1.8157232

H 4.0169940 0.3618401 -2.5226582

C 4.9975780 -2.2104149 -0.7291342

H 6.0237400 -1.5062079 -2.5256852

H 5.5144820 -3.0584489 -0.3082622

C 2.1718100 0.4199731 1.5909098

C 1.5814180 -0.0329969 2.7427238

O 3.2433190 1.2181741 1.9072178

C 2.3204910 0.5283691 3.8291528

H 0.7153730 -0.6749509 2.7978888

C 3.3121480 1.2758561 3.2612688

H 2.1425130 0.3900331 4.8852808

H 4.1101520 1.8793891 3.6655598

C 1.7589770 1.5199821 -1.0565962

C 1.1213700 1.8057161 -2.2353312

O 2.4394590 2.6278431 -0.6192602

C 1.4158200 3.1727161 -2.5363672

H 0.5086130 1.1255531 -2.8055302

C 2.2126540 3.6197221 -1.5212132

H 1.0785230 3.7410811 -3.3903132

H 2.6831200 4.5665141 -1.3056632

C -3.2731420 -0.0228969 0.8191298

C -3.7570870 0.1507301 2.0910588

O -4.2086480 -0.6621769 0.0497518

C -5.0715820 -0.4119489 2.1002178

H -3.2386260 0.6245851 2.9091628

C -5.2923310 -0.8876259 0.8411568

H -5.7582910 -0.4481009 2.9327918

H -6.1275810 -1.3740959 0.3624278

C -1.0678010 1.6957871 1.0219478

C -0.4911310 2.8944941 0.6998018

O -1.0197680 1.5092601 2.3830038

C -0.0690120 3.4848241 1.9339118

H -0.3729110 3.3005521 -0.2925662

C -0.4121190 2.6084871 2.9174718

H 0.4212880 4.4380221 2.0629068

H -0.3050270 2.6092491 3.9900918

C -2.0658370 1.0820841 -1.5315422

C -2.6535150 2.2686931 -1.8803722

O -1.7508370 0.3717691 -2.6657162

C -2.6962910 2.2979121 -3.3084022

H -3.0111280 3.0240331 -1.1963362

C -2.1416660 1.1248871 -3.7305142

H -3.0965710 3.0823951 -3.9334952

H -1.9679120 0.6896421 -4.7022382

H -3.2216050 -3.7755729 0.6398728

C 1.0591290 -3.1412359 -1.2750162

H 1.8775540 -2.6357859 -1.7828602

H 0.2960960 -3.6190089 -1.8821972

C 1.1165130 -3.3668319 0.0437248

C 1.2988860 -3.7518999 1.2780928

H 0.9036040 -3.2023879 2.1251828

H 1.8736210 -4.6519459 1.4841558

#### TS-16

| 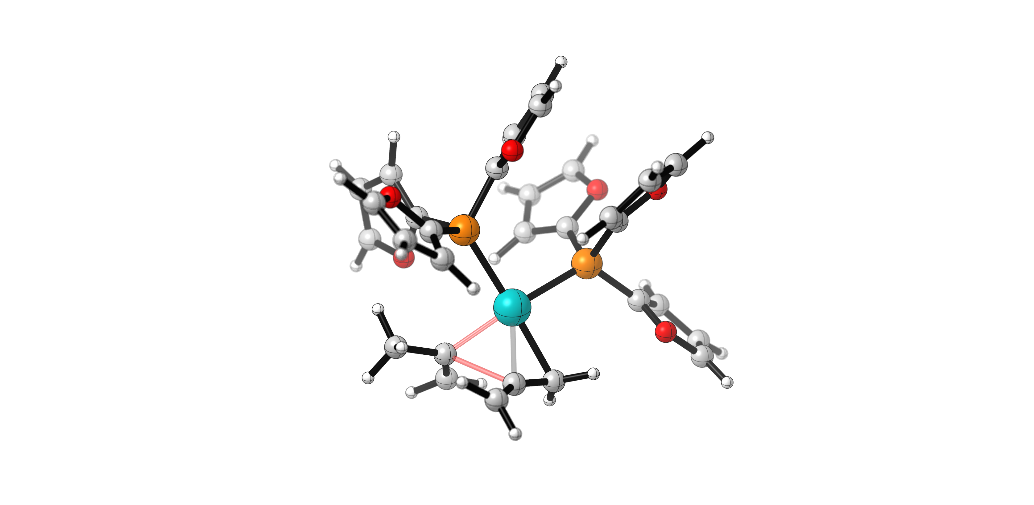 | E/hartree | -2421.208226 |
| --- | --- | --- |
|  | E+zvp/hartree | -2420.709319 |
|  | G/hartree | -2420.783907 |
|  | E(_SP)_/hartree | -2422.054472 |
|  | Imaginary frequency/cm^-1^ | -289.53 |

P 1.9304702 0.0432356 0.0069972

P -1.4912968 -0.3882764 -0.0271408

Pd 0.0192372 1.4349596 -0.0273778

C -2.8855208 2.8052496 0.2766122

H -3.0415368 2.8793306 -0.7999488

H -3.3732898 1.8800666 0.5973172

C -1.4402078 2.7991796 0.6669892

C -0.9841698 3.3455856 1.8006212

H 0.0610792 3.3663706 2.0878882

H -1.6820468 3.7809986 2.5155712

C 3.5475682 0.7977926 0.2493912

C 4.4552692 0.7652436 1.2723502

O 4.0084982 1.6119046 -0.7578208

C 5.5447822 1.6046326 0.8729732

H 4.3636782 0.1996456 2.1873022

C 5.2251542 2.0875206 -0.3598128

H 6.4464702 1.8108336 1.4302882

H 5.7239162 2.7407716 -1.0584048

C 2.1302502 -1.0063564 -1.4268548

C 1.5435692 -0.9280684 -2.6635938

O 3.1124862 -1.9661174 -1.4402158

C 2.1910072 -1.9039694 -3.4824588

H 0.7440082 -0.2587354 -2.9439718

C 3.1278582 -2.5003054 -2.6884628

H 1.9882452 -2.1272954 -4.5193148

H 3.8493042 -3.2855844 -2.8536368

C 1.7846122 -0.9788344 1.4770602

C 0.9956922 -0.8237564 2.5889232

O 2.5577212 -2.1039244 1.6116672

C 1.2919132 -1.9247404 3.4524912

H 0.2823642 -0.0304604 2.7586542

C 2.2403252 -2.6672394 2.8082642

H 0.8575902 -2.1324664 4.4190762

H 2.7702382 -3.5741934 3.0552262

C -2.7340278 -0.1119914 -1.2934208

C -2.7048708 0.6581406 -2.4257658

O -3.8966218 -0.8382444 -1.2564008

C -3.9244258 0.3983436 -3.1253458

H -1.9049618 1.3240596 -2.7146958

C -4.6064938 -0.5107474 -2.3697098

H -4.2500928 0.8317056 -4.0593078

H -5.5654548 -0.9941124 -2.4742058

C -0.9144138 -2.0481384 -0.4473758

C -0.3115168 -3.0565864 0.2583902

O -0.9710988 -2.3853404 -1.7765298

C 0.0220142 -4.0720954 -0.6920888

H -0.1358968 -3.0753634 1.3227642

C -0.3978738 -3.6125824 -1.9051008

H 0.5015892 -5.0186384 -0.4918518

H -0.3736068 -4.0160794 -2.9045528

C -2.3258888 -0.7107734 1.5297192

C -3.0106388 -1.7931484 2.0170282

O -2.2909508 0.2792196 2.4847842

C -3.4233338 -1.4529404 3.3417042

H -3.1904918 -2.7163504 1.4872602

C -2.9619628 -0.1893034 3.5709992

H -3.9888738 -2.0661954 4.0275722

H -3.0236198 0.4851986 4.4108222

H -3.4001108 3.6464466 0.7528082

C 1.2252242 3.2172896 -0.3900688

H 2.0574362 2.9471186 -1.0316758

H 1.4843672 3.6549736 0.5699382

C -0.0138528 3.4517476 -0.9544878

C -0.7504338 3.9693916 -1.9185218

H -1.7631978 3.6536956 -2.1311978

H -0.3511608 4.7983046 -2.4981358

#### Int-26

| 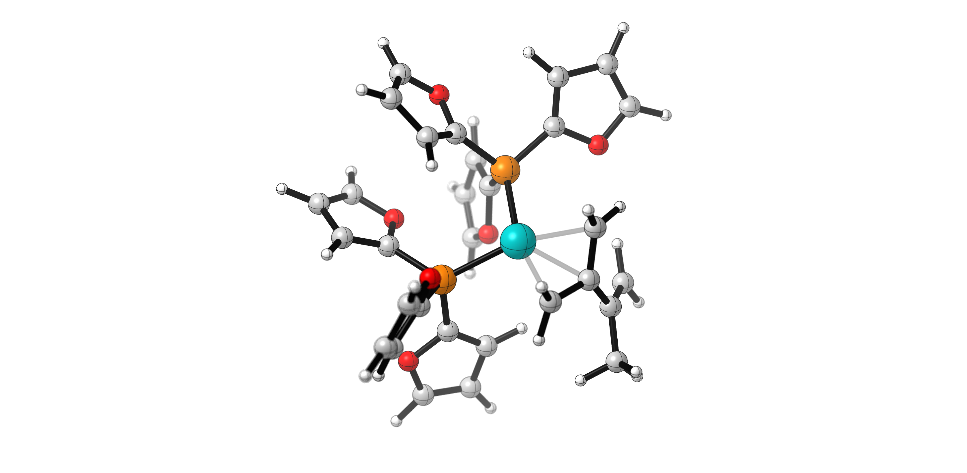 | E/hartree | -2421.299462 |
| --- | --- | --- |
|  | E+zvp/hartree | -2420.796354 |
|  | G/hartree | -2420.872684 |
|  | E(_SP)_/hartree | -2422.142322 |

P 1.9141535 -0.2648756 -0.1917171

P -1.6617575 -0.5092076 -0.1130561

Pd 0.0297225 0.8685954 -0.9170041

C -2.9470355 -0.7671616 -1.3441261

C -4.3060235 -0.9164116 -1.2955871

O -2.5172765 -0.8497996 -2.6507141

C -4.7439465 -1.1003236 -2.6461401

H -4.9096115 -0.9005176 -0.4006261

C -3.6236215 -1.0546236 -3.4211771

H -5.7577055 -1.2448086 -2.9892701

H -3.4470515 -1.1421566 -4.4818261

C -2.4847865 0.2948834 1.2606119

C -2.1837575 1.4530764 1.9288989

O -3.5888395 -0.2955156 1.8295149

C -3.1629705 1.5908794 2.9626669

H -1.3503265 2.1058544 1.7126659

C -3.9852645 0.5072384 2.8544769

H -3.2425795 2.3871584 3.6879039

H -4.8541865 0.1755824 3.4016299

C -1.3209965 -2.1989326 0.4058779

C -1.8124675 -3.4135256 0.0064059

O -0.2941225 -2.3854746 1.2946869

C -1.0426765 -4.4058606 0.6928219

H -2.6242775 -3.5731796 -0.6872461

C -0.1391795 -3.7280626 1.4564849

H -1.1534825 -5.4779266 0.6257489

H 0.6390805 -4.0269016 2.1405309

C 1.9017975 -0.4155146 1.6001619

C 2.6914045 -1.0604956 2.5135399

O 0.8267715 0.1541804 2.2398359

C 2.0625515 -0.8883686 3.7862369

H 3.6018955 -1.5982136 2.2939839

C 0.9378985 -0.1484596 3.5592999

H 2.4057945 -1.2608066 4.7402359

H 0.1552715 0.2357444 4.1944289

C 3.5403165 0.4033024 -0.5752981

C 4.6707625 -0.1155026 -1.1444091

O 3.7501125 1.7217274 -0.2419791

C 5.6375065 0.9414194 -1.1635281

H 4.7988205 -1.1295426 -1.4912171

C 5.0316035 2.0251424 -0.6045561

H 6.6479305 0.8933584 -1.5415751

H 5.3501565 3.0350254 -0.3989521

C 2.0010245 -1.9294076 -0.8475771

C 1.2280185 -2.5646916 -1.7825851

O 2.9485545 -2.7999316 -0.3604401

C 1.7252395 -3.9021936 -1.8815001

H 0.4002655 -2.1281156 -2.3221621

C 2.7623295 -3.9885006 -1.0007921

H 1.3561085 -4.6910036 -2.5196661

H 3.4385305 -4.7820486 -0.7228201

C -1.5043975 4.7306714 -0.0337701

C -1.3644345 2.3165504 -1.8201851

C -0.2403395 3.9300264 -0.2225811

C -0.2003245 3.0095414 -1.4017001

H -2.3185435 2.5039874 -1.3427811

H -1.4153345 1.8870774 -2.8171181

C 0.7937785 4.0184264 0.6262409

C 1.0193295 2.5280364 -1.9447471

H -2.3462305 4.0861744 0.2464789

H -1.7898205 5.2506774 -0.9541411

H 1.6895795 3.4154594 0.5219319

H 0.7600115 4.6994764 1.4704839

H 1.0256775 2.1116334 -2.9493851

H 1.9684315 2.9048794 -1.5870411

H -1.3770585 5.4722784 0.7572099

#### Int-27

| 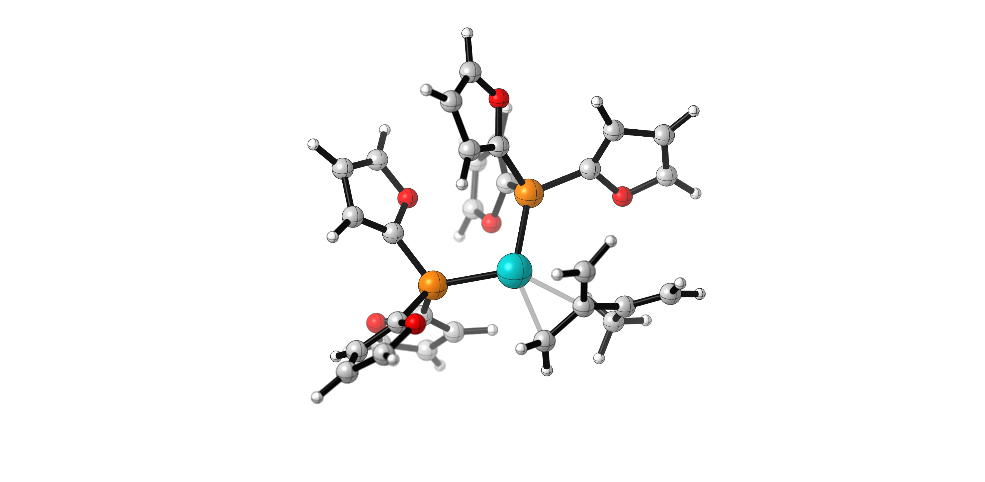 | E/hartree | -2421.435534 |
| --- | --- | --- |
|  | E+zvp/hartree | -2420.937069 |
|  | G/hartree | -2421.018314 |
|  | E(_SP)_/hartree | -2422.258455 |

P 1.5561099 -0.6119805 -0.0482005

P -2.0407851 0.2274675 -0.0656815

Pd 0.0533679 1.1428545 -0.2839955

C -3.4239781 0.9012035 -1.0273445

C -4.7830241 0.9460485 -0.8675395

O -3.0836401 1.5390685 -2.2000035

C -5.3099061 1.6478645 -1.9985105

H -5.3267871 0.5212125 -0.0375255

C -4.2362031 1.9793915 -2.7711465

H -6.3455661 1.8762865 -2.2046195

H -4.1254611 2.5060635 -3.7061525

C -2.6719261 0.3086855 1.6250755

C -2.1110461 0.8717365 2.7384335

O -3.8578601 -0.3048135 1.9701585

C -2.9968071 0.5999695 3.8304515

H -1.1661451 1.3937655 2.7568085

C -4.0342971 -0.1132775 3.3060035

H -2.8774121 0.8964625 4.8624405

H -4.9321821 -0.5385585 3.7274585

C -2.2318721 -1.5344115 -0.4684015

C -2.9658621 -2.2343125 -1.3883935

O -1.3481731 -2.3839445 0.1487325

C -2.5066121 -3.5919495 -1.3308205

H -3.7416511 -1.8260715 -2.0184735

C -1.5281721 -3.6237935 -0.3832525

H -2.8614341 -4.4269605 -1.9173435

H -0.8896891 -4.3962225 0.0146445

C 1.3026879 -1.6613675 1.4104995

C 1.7170099 -2.9054575 1.8049395

O 0.3960059 -1.1769485 2.3225155

C 1.0200779 -3.2061795 3.0184645

H 2.4226999 -3.5263805 1.2740415

C 0.2320629 -2.1225095 3.2804995

H 1.0960379 -4.1051195 3.6132165

H -0.4670431 -1.8758025 4.0641825

C 3.3447399 -0.3119205 -0.0351935

C 4.3852049 -0.6486935 -0.8580045

O 3.7842549 0.5693585 0.9224645

C 5.5329229 0.0640595 -0.3772645

H 4.3347759 -1.3294065 -1.6938375

C 5.1107639 0.7820515 0.7004715

H 6.5342869 0.0400285 -0.7820615

H 5.5978019 1.4589185 1.3841655

C 1.4278699 -1.7944435 -1.4062405

C 0.6264659 -1.7624455 -2.5142895

O 2.1493579 -2.9705825 -1.3979595

C 0.8597279 -2.9800675 -3.2303015

H -0.0576261 -0.9644615 -2.7612295

C 1.7876149 -3.6707755 -2.5098655

H 0.3939299 -3.2973855 -4.1516145

H 2.2748679 -4.6243805 -2.6434795

C 1.7625159 3.2990255 1.7117325

C -0.2890921 3.3443435 -0.5047855

C 2.1248849 3.4854355 0.2587115

C 1.0974609 3.1566945 -0.7792795

H -0.6023491 3.8437245 0.4052645

H -0.9798611 3.4262025 -1.3388495

C 3.3336369 3.9450115 -0.0931275

C 1.5135849 2.7021085 -2.0518105

H 1.5010099 2.2531905 1.9086125

H 0.8952219 3.9065235 1.9936245

H 3.6041379 4.1119495 -1.1298215

H 4.0840449 4.1785615 0.6562995

H 0.7997869 2.6096525 -2.8627115

H 2.5390719 2.4058535 -2.2307535

H 2.5964599 3.5798475 2.3599725

#### TS-17

| 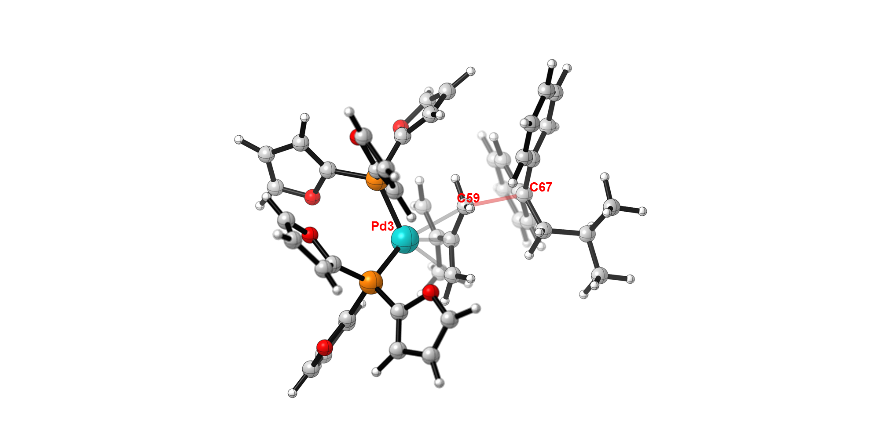  TS with fixed distances between  Pd3 and C59 as well as between C59 and C67 | E/hartree | -3080.799854 |
| --- | --- | --- |
|  | E+zvp/hartree | -3079.985708 |
|  | G/hartree | -3080.083120 |
|  | E(_SP)_/hartree | -3081.895943 |
|  | Imaginary frequency/cm^-1^ | -219.36 |

P 1.5552985 1.9530458 0.5156255

P 3.4159415 -1.1471952 -0.1872025

Pd 1.3495065 -0.3848412 0.4892185

C 3.4634995 -2.1801952 -1.6750605

C 4.1631285 -3.2864352 -2.0727215

O 2.5761925 -1.8218282 -2.6677945

C 3.6853005 -3.6305702 -3.3791675

H 4.9293585 -3.7812592 -1.4959285

C 2.7309565 -2.7090872 -3.6897695

H 4.0091755 -4.4542222 -3.9986625

H 2.0954725 -2.5528962 -4.5473795

C 4.1778995 -2.2136202 1.0508065

C 3.7094895 -2.5788152 2.2843865

O 5.4241375 -2.7677732 0.8510755

C 4.7134095 -3.4088252 2.8780715

H 2.7618675 -2.2712172 2.7022845

C 5.7242615 -3.4883052 1.9667415

H 4.6860675 -3.8781032 3.8505745

H 6.6800415 -3.9893842 1.9590185

C 4.7133285 0.0475148 -0.6078485

C 5.5416455 0.2274598 -1.6827065

O 4.7850465 1.1478208 0.2100325

C 6.1572125 1.5108248 -1.5152545

H 5.6829695 -0.4723052 -2.4926805

C 5.6614325 2.0228078 -0.3531555

H 6.8688155 1.9854928 -2.1750235

H 5.8115925 2.9413748 0.1914725

C 2.6741195 2.7435708 1.6986075

C 3.2511355 3.9803598 1.8107835

O 3.1447825 1.9291238 2.7008155

C 4.1302245 3.9229368 2.9381195

H 3.0688065 4.8162148 1.1528705

C 4.0226545 2.6558718 3.4357235

H 4.7504685 4.7168698 3.3285625

H 4.4740145 2.1434778 4.2708345

C 0.0622675 2.9862108 0.5830305

C -0.7728785 3.4383908 -0.4031555

O -0.5628445 3.1040618 1.8026415

C -1.9779045 3.8692598 0.2417995

H -0.5670585 3.4391028 -1.4618915

C -1.7968845 3.6499018 1.5733795

H -2.8621705 4.2602588 -0.2383675

H -2.4014295 3.8204578 2.4499605

C 2.1882895 2.4979768 -1.0889465

C 2.3741485 1.8045848 -2.2545695

O 2.5348355 3.8183038 -1.2840245

C 2.8675915 2.7417378 -3.2186595

H 2.1884195 0.7504638 -2.3962065

C 2.9475435 3.9402698 -2.5750725

H 3.1333765 2.5436378 -4.2466225

H 3.2647645 4.9268508 -2.8759305

C -0.7986245 -2.5098372 3.2508375

C 0.0948445 -2.2189842 0.4580525

C -0.8276785 -1.1685182 2.5581095

C -0.6059685 -1.1665612 1.0791905

H 0.4457315 -3.0704762 1.0281995

H -0.0004955 -2.3846112 -0.6101105

C -1.0453385 -0.0493992 3.2619045

C -1.1496215 -0.1226152 0.2286405

H 0.2010705 -2.9608632 3.2183145

H -1.4864035 -3.2140602 2.7721485

H -1.0424255 0.9338268 2.8103145

H -1.2424445 -0.0959112 4.3276655

H -0.7940375 -0.0802902 -0.7935325

H -1.3602185 0.8410838 0.6653825

H -1.0835045 -2.4069022 4.3002965

C -3.2094835 -0.4922232 -0.3205175

C -3.7861865 -0.5837542 1.0429435

C -4.0380135 -1.8245192 1.6610535

C -3.9647455 0.5682498 1.8429835

C -4.4376315 -1.9104782 2.9955035

H -3.9089185 -2.7433992 1.1029585

C -4.3706855 0.4827648 3.1673615

H -3.7262895 1.5407878 1.4290675

C -4.6090965 -0.7609102 3.7614665

H -4.6139795 -2.8888052 3.4345775

H -4.4822325 1.3931248 3.7508075

H -4.9207465 -0.8278062 4.7994425

C -3.5146665 0.7093708 -1.1548685

C -4.6478815 1.5194378 -0.9424815

C -2.6866235 1.0573678 -2.2422505

C -4.9153475 2.6298318 -1.7414435

H -5.3400745 1.2614398 -0.1499715

C -2.9490055 2.1669668 -3.0409005

H -1.8208505 0.4467648 -2.4733005

C -4.0617695 2.9722718 -2.7914375

H -5.8053735 3.2227748 -1.5479415

H -2.2799085 2.4019818 -3.8645155

H -4.2685575 3.8379528 -3.4134445

C -3.0674905 -1.7928872 -1.0969945

H -2.5295335 -2.5139772 -0.4711105

H -2.4227865 -1.6247562 -1.9686455

C -4.3763285 -2.4507282 -1.6016445

H -5.1027275 -2.4430662 -0.7792605

C -4.9990765 -1.6971262 -2.7838295

H -5.2758335 -0.6755232 -2.5183655

H -4.2937255 -1.6407912 -3.6221555

H -5.8986355 -2.2124022 -3.1391415

C -4.1034755 -3.9117232 -1.9814695

H -3.7171415 -4.4821582 -1.1292395

H -5.0121575 -4.4092652 -2.3373475

H -3.3572225 -3.9700232 -2.7839365

#### Int-28

| 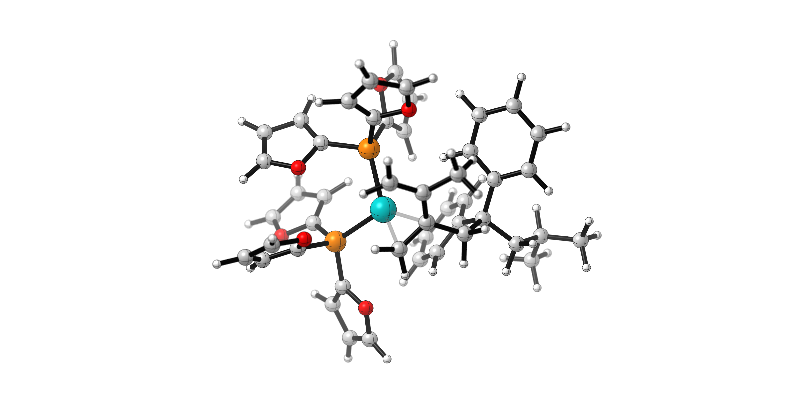 | E/hartree | -3080.848441 |
| --- | --- | --- |
|  | E+zvp/hartree | -3080.029798 |
|  | G/hartree | -3080.121642 |
|  | E(_SP)_/hartree | -3081.935603 |

P 2.8577982 -1.0831308 -0.3227396

P 0.9493762 2.0257902 0.3991434

Pd 0.8151142 -0.0538558 -0.6339006

C -0.8042438 -0.1953658 -2.3469016

C 0.3578712 -0.9256398 -2.6147986

C -2.0463868 -1.0343698 -2.0156106

H 1.1420622 -0.5179318 -3.2428666

H -2.8545208 -0.7304398 -2.6872566

H -1.8097888 -2.0606758 -2.3095146

H 0.3582362 -2.0071148 -2.5360596

C -2.7184818 -1.1364258 -0.5962046

C -3.4117158 0.1901092 -0.2693916

C -4.7365668 0.4488442 -0.6500116

C -2.7065658 1.2249952 0.3561884

C -5.3341528 1.6843922 -0.3985206

H -5.3171978 -0.3109488 -1.1576046

C -3.2998178 2.4566522 0.6246954

H -1.6744618 1.0625512 0.6279194

C -4.6212648 2.6939042 0.2487274

H -6.3623918 1.8527562 -0.7059826

H -2.7139538 3.2304722 1.1082884

H -5.0895988 3.6521992 0.4540124

C -1.7565088 -1.5811438 0.5142954

C -0.7098798 -2.4780148 0.2448704

C -2.0120338 -1.2799038 1.8621514

C 0.0510532 -3.0428488 1.2716954

H -0.5034448 -2.7839368 -0.7705256

C -1.2567188 -1.8426498 2.8910054

H -2.8214758 -0.6064798 2.1143764

C -0.2129338 -2.7222978 2.6013734

H 0.8440242 -3.7394638 1.0265424

H -1.4956158 -1.5988638 3.9227884

H 0.3765592 -3.1624848 3.3999544

C -3.7589578 -2.2978238 -0.7621536

H -3.1967708 -3.1884828 -1.0644946

H -4.3970118 -2.0563198 -1.6192166

C -4.6381228 -2.6561838 0.4605434

H -4.6567368 -1.7961118 1.1400634

C -4.0854568 -3.8668838 1.2281374

H -4.6885028 -4.0718598 2.1196354

H -4.1158788 -4.7621118 0.5946584

H -3.0528028 -3.7158628 1.5464834

C -6.0845748 -2.9353588 0.0282664

H -6.1225158 -3.7631368 -0.6903186

H -6.7068078 -3.2127068 0.8860014

H -6.5403478 -2.0601938 -0.4459986

C -0.9518998 1.1838542 -2.9290706

C 0.1031222 1.9299562 -3.2992356

H -0.0398968 2.9054742 -3.7504496

H 1.1268652 1.6229952 -3.1157216

C -2.3432138 1.7271312 -3.1584526

H -2.8997108 1.8175542 -2.2236916

H -2.2882328 2.7169352 -3.6175456

H -2.9313838 1.0832572 -3.8235616

C 3.7126462 -0.8002678 1.2473024

C 3.2288462 -0.2723838 2.4124334

O 5.0376322 -1.1513298 1.4235774

C 4.3055322 -0.2892818 3.3565004

H 2.2256902 0.0936892 2.5652394

C 5.3735472 -0.8271568 2.7043894

H 4.2853832 0.0629592 4.3773954

H 6.3944292 -1.0313298 2.9885114

C 2.9632702 -2.9000138 -0.3745496

C 3.5863152 -3.8490558 0.3907434

O 2.1825752 -3.5230618 -1.3210116

C 3.1676802 -5.1250798 -0.1092826

H 4.2626762 -3.6519748 1.2079834

C 2.3218622 -4.8678298 -1.1464466

H 3.4598672 -6.0993788 0.2549474

H 1.7681422 -5.4904418 -1.8315046

C 4.1202722 -0.6059498 -1.5451916

C 5.4783432 -0.7530908 -1.6436586

O 3.6676132 0.1229102 -2.6200016

C 5.8846112 -0.0707838 -2.8350206

H 6.1011872 -1.2774218 -0.9356506

C 4.7438582 0.4382522 -3.3847976

H 6.8860102 0.0206962 -3.2304976

H 4.5353902 1.0099022 -4.2756346

C 2.6857352 2.4969972 0.6887844

C 3.3458142 3.1560142 1.6901944

O 3.6022052 2.0125942 -0.2112566

C 4.7451932 3.0600982 1.3956204

H 2.8814382 3.6446212 2.5329924

C 4.8421432 2.3559012 0.2326214

H 5.5643422 3.4579732 1.9768204

H 5.6619602 2.0198232 -0.3820206

C 0.2774682 2.3968732 2.0427214

C -0.2279468 1.5514712 2.9930464

O 0.2295602 3.6927102 2.5143674

C -0.6050828 2.3617212 4.1120134

H -0.3357808 0.4834532 2.8822854

C -0.3090248 3.6462992 3.7645744

H -1.0480998 2.0320632 5.0405064

H -0.4238568 4.5971852 4.2619014

C 0.4332502 3.5046642 -0.5283736

C 1.1256622 4.5152502 -1.1380216

O -0.9032158 3.6486362 -0.7825696

C 0.1529412 5.3309732 -1.8047616

H 2.1950352 4.6585272 -1.1052676

C -1.0584398 4.7595792 -1.5529656

H 0.3358902 6.2216872 -2.3882096

H -2.0741048 4.9995042 -1.8247926

#### TS-18

| 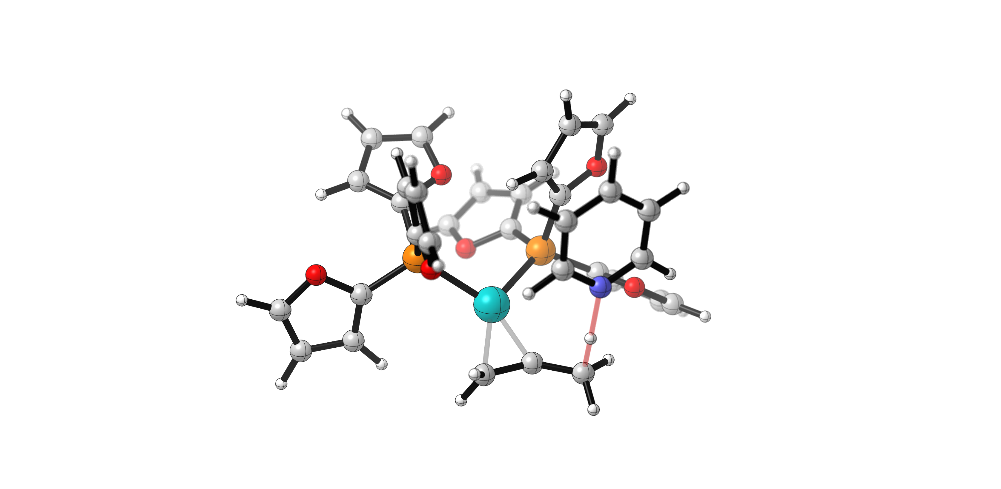 | E/hartree | -2552.822853 |
| --- | --- | --- |
|  | E+zvp/hartree | -2552.296915 |
|  | G/hartree | -2552.376586 |
|  | E(_SP)_/hartree | -2553.722623 |
|  | Imaginary frequency/cm^-1^ | -202.70 |

P 1.2942196 1.2252072 -0.4002759

P -2.2317504 0.0136652 -0.2702559

Pd -0.2719914 -0.0424088 -1.6094869

C -2.4266594 -1.5667198 0.5951271

C -2.6924604 -1.9017508 1.8945441

O -2.2526824 -2.7100438 -0.1507339

C -2.6951664 -3.3339098 1.9554961

H -2.8856814 -1.2069718 2.6981801

C -2.4329914 -3.7701518 0.6911761

H -2.8873424 -3.9502608 2.8215811

H -2.3592234 -4.7471938 0.2391561

C -3.7448464 0.1971742 -1.2203579

C -3.9448884 0.5534252 -2.5277269

O -4.9599214 -0.0159708 -0.6108689

C -5.3593894 0.5647332 -2.7388199

H -3.1690074 0.7820352 -3.2427959

C -5.9215084 0.2111602 -1.5479859

H -5.8837124 0.7993482 -3.6532049

H -6.9397574 0.0739362 -1.2183349

C -2.4676634 1.1902692 1.0758561

C -3.5546674 1.8319572 1.6084581

O -1.3293824 1.6041652 1.7235531

C -3.0557704 2.6911342 2.6386251

H -4.5800434 1.6986272 1.2993431

C -1.7037144 2.5127712 2.6632851

H -3.6295144 3.3534342 3.2699631

H -0.9024084 2.9366912 3.2467431

C 0.7544386 2.9040772 -0.0253479

C 1.2234796 3.8995582 0.7900551

O -0.4470214 3.2868722 -0.5728889

C 0.2597916 4.9550352 0.7418901

H 2.1423636 3.8676622 1.3570601

C -0.7300594 4.5275272 -0.0952819

H 0.2991056 5.9021222 1.2596721

H -1.6522224 4.9656302 -0.4430199

C 2.9169766 1.5502722 -1.1316659

C 3.5438966 2.6888632 -1.5548719

O 3.7141106 0.4588452 -1.3948529

C 4.8041706 2.2837212 -2.1046849

H 3.1561486 3.6925382 -1.4730319

C 4.8588056 0.9305742 -1.9797359

H 5.5652366 2.9218562 -2.5288709

H 5.5968896 0.1863492 -2.2350719

C 1.7275076 0.5203482 1.1997481

C 1.0941486 -0.4020158 1.9874881

O 2.8296156 1.0135122 1.8623771

C 1.8436366 -0.4850538 3.2030051

H 0.1970836 -0.9453128 1.7346271

C 2.8815206 0.3904152 3.0707051

H 1.6382716 -1.1135858 4.0569471

H 3.7067016 0.6683142 3.7081991

C -0.7152624 -1.3116248 -3.2665999

C 0.6311276 -1.0219138 -3.0673399

H -1.2558934 -0.7580308 -4.0344589

H -1.1087554 -2.3064288 -3.0604319

C 1.9196696 -1.5084018 -3.3132249

H 2.7333436 -0.8090468 -3.1172909

H 2.0631736 -2.1541648 -4.1818249

C 3.3218796 -2.6365868 -0.1171009

C 3.4327356 -3.0646088 1.2025451

C 2.3142856 -3.6256938 1.8175941

C 1.1227446 -3.7276338 1.1002791

C 1.1010486 -3.2776248 -0.2168109

H 2.0099786 -2.1527058 -2.2726169

H 4.1554376 -2.1778978 -0.6386219

H 4.3709476 -2.9516248 1.7334231

H 2.3680186 -3.9682938 2.8458661

H 0.2217426 -4.1326558 1.5462611

H 0.1941596 -3.3202078 -0.8116699

N 2.1829476 -2.7573318 -0.8106659

#### Int-29

| 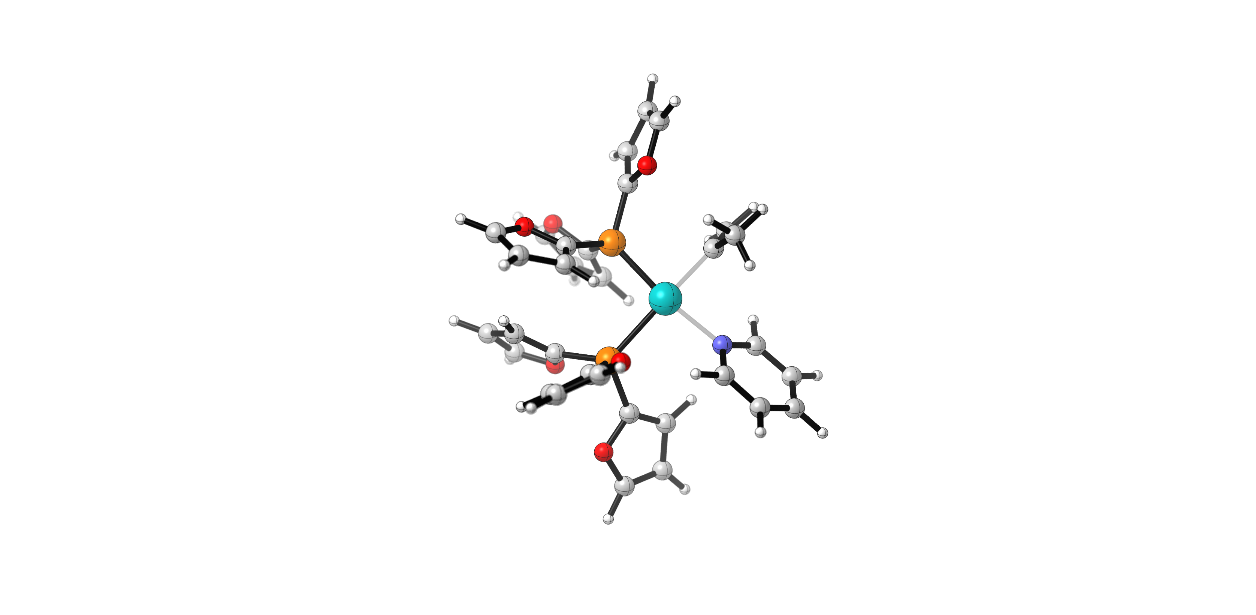 | E/hartree | -2552.886492 |
| --- | --- | --- |
|  | E+zvp/hartree | -2552.353917 |
|  | G/hartree | -2552.431636 |
|  | E(_SP)_/hartree | -2553.789183 |

P 1.9562886 -0.2299508 -0.0066374

P -1.2636574 1.0565232 0.0325936

Pd -0.1543714 -1.1121218 -0.0239384

C 0.6634936 -3.6976208 1.2274736

H 1.2285236 -3.1735328 2.0047516

H -0.3685904 -3.7963478 1.5822606

C 0.7264436 -2.9466388 -0.0702734

C 1.2286416 -3.4324688 -1.2061264

H 1.3033886 -2.8461608 -2.1183664

H 1.6150896 -4.4516588 -1.2563284

C 2.1268916 0.9688632 1.3222046

C 1.4557046 1.0913242 2.5107446

O 3.1170406 1.9156022 1.2490786

C 2.0540496 2.1905912 3.2026166

H 0.6364206 0.4734812 2.8429626

C 3.0513156 2.6508782 2.3909356

H 1.7783516 2.5800692 4.1711856

H 3.7724806 3.4488492 2.4772106

C 2.2599756 0.6059142 -1.5615424

C 1.5524416 0.5823322 -2.7349534

O 3.4145066 1.3307902 -1.7333634

C 2.3085336 1.3402892 -3.6822784

H 0.6048726 0.0885232 -2.8905064

C 3.4218226 1.7691872 -3.0199974

H 2.0556956 1.5376562 -4.7133414

H 4.2722066 2.3688322 -3.3055344

C 3.4018026 -1.2844928 0.1461446

C 4.4502856 -1.5692248 -0.6845774

O 3.4824086 -2.0472268 1.2827576

C 5.2254316 -2.5731398 -0.0214024

H 4.6458646 -1.1153288 -1.6437224

C 4.5956546 -2.8246918 1.1608826

H 6.1332056 -3.0367048 -0.3780924

H 4.8024066 -3.4805978 1.9917886

C -0.4045454 2.5208862 -0.5851684

C 0.4961486 3.3996232 -0.0461354

O -0.5297584 2.7516352 -1.9319754

C 0.9479686 4.2260202 -1.1251944

H 0.7977306 3.4528872 0.9884086

C 0.2972256 3.7903612 -2.2397414

H 1.6560636 5.0393442 -1.0685754

H 0.2984646 4.0975562 -3.2731634

C -2.8176344 1.1102242 -0.8726434

C -3.3707374 0.2740672 -1.8077534

O -3.6316014 2.2037862 -0.6981994

C -4.5930614 0.8828432 -2.2301634

H -2.9539224 -0.6597508 -2.1507694

C -4.6983674 2.0457962 -1.5244004

H -5.2955274 0.5054652 -2.9583064

H -5.4383394 2.8305242 -1.4921784

C -1.6653434 1.5812612 1.7094116

C -1.8645634 2.7894332 2.3170346

O -1.7794904 0.5732582 2.6458516

C -2.1088154 2.5204162 3.7012836

H -1.8342724 3.7514092 1.8286096

C -2.0497774 1.1668802 3.8453376

H -2.3074354 3.2411802 4.4807856

H -2.1730414 0.4985332 4.6831466

H 1.0891076 -4.7033518 1.1181746

C -2.9750704 -1.9616108 0.8435206

C -2.2629284 -3.0584598 -1.0723784

C -4.2109794 -2.5961728 0.7953176

H -2.7333404 -1.2537758 1.6267836

C -3.4808184 -3.7180278 -1.1950714

H -1.4470344 -3.2322348 -1.7631074

C -4.4748474 -3.4846288 -0.2457314

H -4.9495234 -2.3871448 1.5608246

H -3.6356974 -4.4063958 -2.0181654

H -5.4335184 -3.9883248 -0.3135144

N -2.0173384 -2.1853088 -0.0767614

#### Int-30

| 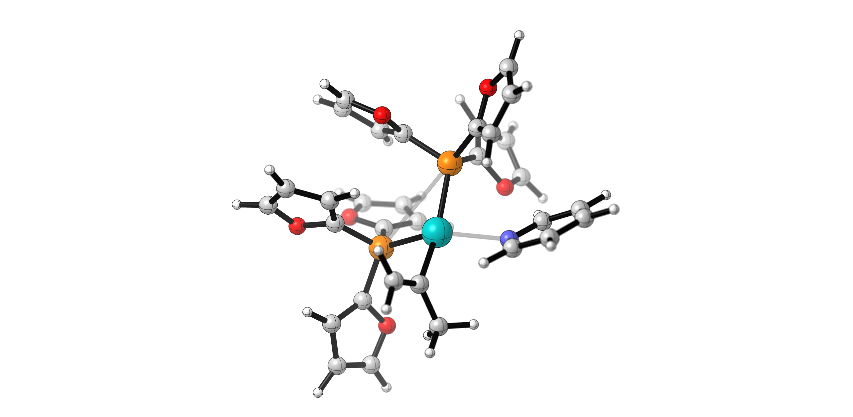 | E/hartree | -2553.008735 |
| --- | --- | --- |
|  | E+zvp/hartree | -2552.480380 |
|  | G/hartree | -2552.561676 |
|  | E(_SP)_/hartree | -2553.892205 |

P -2.0190730 0.2701309 0.2192276

P 1.2650380 -1.0090091 -0.0695934

Pd 0.1583540 1.0734299 -0.0788724

C -0.9025310 3.9276489 0.3526966

H -1.6646240 3.5525359 1.0447196

H -0.0042730 4.1097589 0.9561746

C -0.6232730 2.9222859 -0.7364424

C -0.8208010 3.2394309 -2.0236024

H -0.6513380 2.5363239 -2.8361044

H -1.1801710 4.2311639 -2.3247904

C -2.2744430 -1.1517971 1.3178836

C -1.5562460 -1.5089541 2.4283376

O -3.2545620 -2.0909571 1.0888096

C -2.1161470 -2.7352571 2.9081876

H -0.7190910 -0.9626851 2.8329636

C -3.1362620 -3.0413161 2.0542176

H -1.7935690 -3.3108651 3.7632916

H -3.8404190 -3.8574321 1.9991616

C -2.5773250 -0.3186241 -1.3906564

C -1.9396490 -0.2808251 -2.6011574

O -3.8276820 -0.8763061 -1.5493614

C -2.8369240 -0.8513911 -3.5580024

H -0.9471840 0.1097859 -2.7635954

C -3.9605850 -1.1926791 -2.8637044

H -2.6678030 -0.9859321 -4.6163974

H -4.8983130 -1.6473701 -3.1443004

C -3.3808420 1.3778799 0.6609826

C -4.3033940 2.0688359 -0.0754784

O -3.4284360 1.7950389 1.9702746

C -4.9689950 2.9550789 0.8326936

H -4.4786460 1.9547419 -1.1339234

C -4.4032080 2.7465449 2.0540806

H -5.7643590 3.6492979 0.6031406

H -4.5716700 3.1593009 3.0362246

C 0.3822430 -2.3463661 -0.9282224

C -0.5513730 -3.2702801 -0.5409824

O 0.4881520 -2.3463951 -2.2972424

C -1.0420210 -3.8805241 -1.7412644

H -0.8572620 -3.4805291 0.4719136

C -0.3817950 -3.2840561 -2.7712204

H -1.7903180 -4.6555911 -1.8181784

H -0.4049160 -3.4016231 -3.8426914

C 2.8837410 -1.0539161 -0.8767814

C 3.5589130 -0.0770521 -1.5583094

O 3.6377280 -2.2072431 -0.8639874

C 4.7948790 -0.6543701 -1.9901804

H 3.2068040 0.9301599 -1.7198734

C 4.7857780 -1.9425451 -1.5418564

H 5.5802440 -0.1745471 -2.5556594

H 5.4868980 -2.7597211 -1.6167734

C 1.5922350 -1.8581231 1.5051736

C 1.7298320 -3.1614481 1.8956716

O 1.7015850 -1.0426331 2.6124616

C 1.9261390 -3.1547131 3.3137036

H 1.6856610 -4.0170691 1.2397936

C 1.9041100 -1.8461821 3.6940456

H 2.0680360 -4.0092721 3.9593756

H 2.0127870 -1.3408401 4.6409066

H -1.2531230 4.8927069 -0.0453134

C 3.1203560 1.7519879 1.3418826

C 2.5854650 3.2333609 -0.3410804

C 4.4624000 2.1251869 1.3104466

H 2.7648460 0.9900779 2.0275626

C 3.9048140 3.6702039 -0.4364134

H 1.8007280 3.6249789 -0.9830044

C 4.8660350 3.1003329 0.4000276

H 5.1708160 1.6539929 1.9839316

H 4.1681280 4.4389819 -1.1556614

H 5.9051190 3.4103389 0.3432746

N 2.1947850 2.2849189 0.5274416

#### Int-31

| 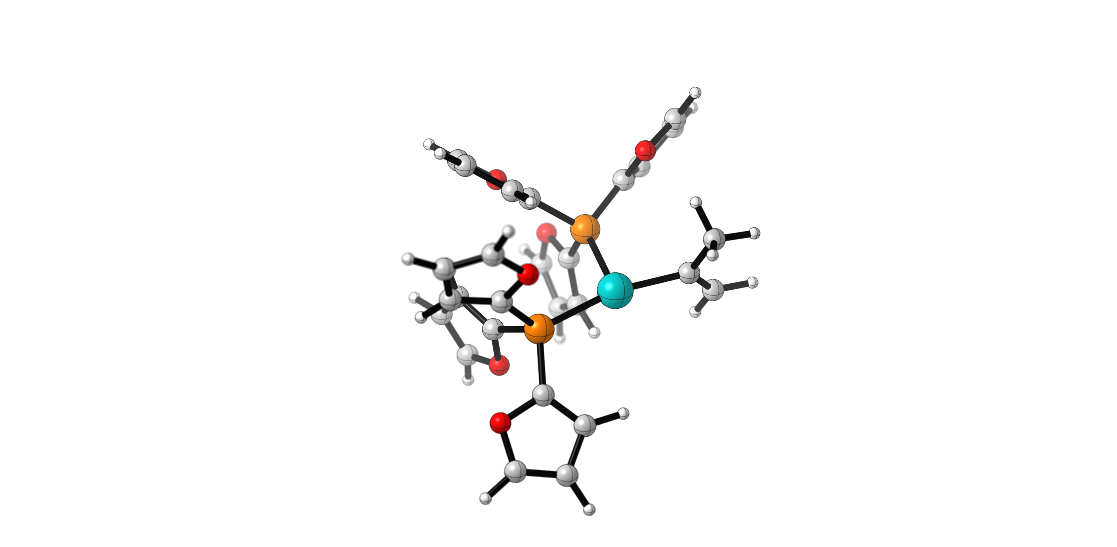 | E/hartree | -2304.682162 |
| --- | --- | --- |
|  | E+zvp/hartree | -2304.244040 |
|  | G/hartree | -2304.319039 |
|  | E(_SP)_/hartree | -2305.458712 |

P 1.6013588 -0.0120778 0.0705928

P -1.9474612 -0.4332128 0.0685938

Pd 0.0073408 -1.7272378 -0.0137402

C 1.8039338 -3.9692878 1.0174668

H 2.2779178 -3.2846358 1.7299478

H 0.9318678 -4.4006978 1.5230038

C 1.4060448 -3.2285578 -0.2355182

C 1.9297458 -3.5395348 -1.4294572

H 1.6961868 -2.9843928 -2.3354492

H 2.6214908 -4.3798768 -1.5514502

C 1.2168538 1.3047862 1.2509218

C 0.4795968 1.2340782 2.4037338

O 1.6500038 2.5968942 1.0633468

C 0.4465788 2.5546472 2.9535278

H 0.0075268 0.3456112 2.7928878

C 1.1664038 3.3368332 2.0969968

H -0.0537312 2.8737792 3.8558798

H 1.4186378 4.3860282 2.0816208

C 1.6465828 0.7940752 -1.5379872

C 1.0434908 0.4140022 -2.7070882

O 2.4028948 1.9246812 -1.7593812

C 1.4416558 1.3605662 -3.7032282

H 0.3903108 -0.4383158 -2.8202262

C 2.2602598 2.2499842 -3.0718692

H 1.1550388 1.3755972 -4.7445122

H 2.7933308 3.1301152 -3.3972092

C 3.3552058 -0.3508218 0.3472068

C 4.4941158 -0.1640258 -0.3872162

O 3.6537198 -1.0504378 1.4914128

C 5.5579978 -0.7807798 0.3474958

H 4.5593758 0.3581842 -1.3290072

C 4.9931798 -1.2980248 1.4741968

H 6.6010748 -0.8291758 0.0706758

H 5.3800378 -1.8417318 2.3215828

C -1.8487762 1.1845502 -0.7567212

C -1.4353112 2.4278832 -0.3598372

O -2.0131732 1.1681672 -2.1196262

C -1.3496302 3.2261232 -1.5472542

H -1.2102722 2.7317422 0.6505538

C -1.7079042 2.4150432 -2.5800032

H -1.0574982 4.2637912 -1.6140122

H -1.7981442 2.5559082 -3.6450202

C -3.3886152 -1.1689068 -0.7278112

C -3.5266412 -2.3918568 -1.3280392

O -4.5773242 -0.4804598 -0.8037902

C -4.8748092 -2.4650408 -1.8026152

H -2.7468232 -3.1351648 -1.4128572

C -5.4597112 -1.2822798 -1.4570242

H -5.3421752 -3.2851428 -2.3276082

H -6.4501742 -0.8763118 -1.5950712

C -2.5506262 0.0960332 1.6923568

C -3.2709042 1.1621712 2.1582018

O -2.1964922 -0.7107248 2.7500908

C -3.3618502 1.0106222 3.5781758

H -3.6765652 1.9568642 1.5511438

C -2.6968422 -0.1414268 3.8797688

H -3.8600362 1.6673842 4.2764828

H -2.5024132 -0.6723828 4.7984898

H 2.5141448 -4.7836228 0.8042768

#### Int-32

| 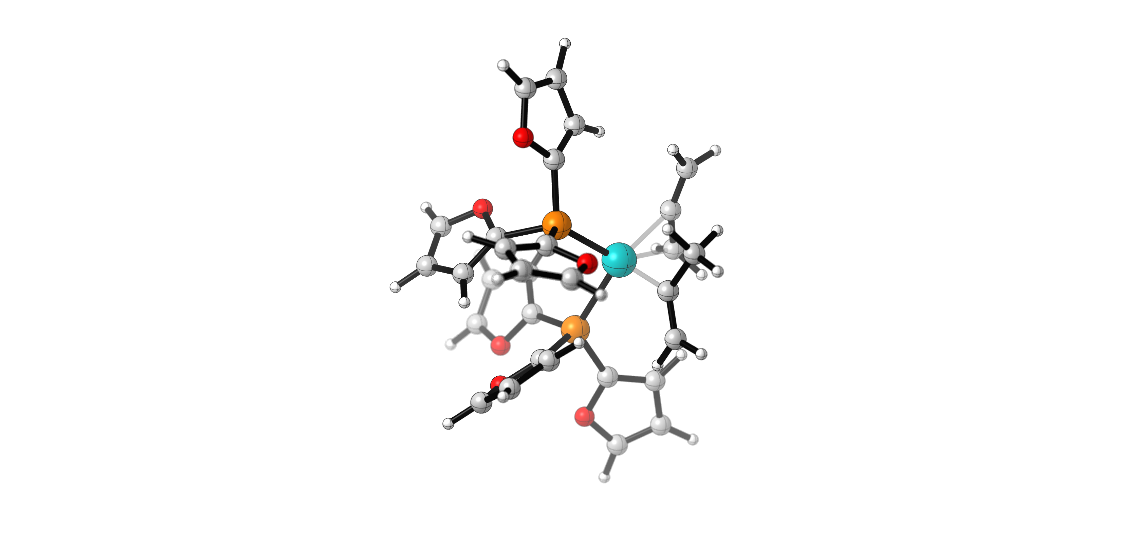 | E/hartree | -2421.369964 |
| --- | --- | --- |
|  | E+zvp/hartree | -2420.873644 |
|  | G/hartree | -2420.949785 |
|  | E(_SP)_/hartree | -2422.192548 |

P 1.8645468 0.0181507 -0.1846678

P -1.7040872 -0.1938193 -0.0809808

Pd -0.0351472 1.3973287 -0.6972018

C -1.3087862 3.5439197 1.2028022

H -1.4601412 4.2024417 0.3426712

H -2.1848042 2.8915507 1.2619922

C -0.0475302 2.7485477 1.0503842

C 1.0039088 2.8691267 1.8595642

H 1.9269328 2.3123247 1.7379002

H 0.9789218 3.5637077 2.7067952

C 3.4906218 0.8008417 -0.1052708

C 3.8500528 2.1097617 -0.2894338

O 4.6111288 0.0543837 0.1801652

C 5.2683718 2.1764327 -0.1110508

H 3.1676758 2.9167107 -0.5069048

C 5.6716828 0.9044197 0.1711492

H 5.8978728 3.0515047 -0.1798898

H 6.6320328 0.4592247 0.3812292

C 2.0828868 -1.2418483 -1.4582408

C 1.4773398 -1.3318473 -2.6843448

O 3.0279898 -2.2339163 -1.3423028

C 2.0762568 -2.4421163 -3.3592808

H 0.6883728 -0.6848203 -3.0379118

C 3.0036728 -2.9487713 -2.4969808

H 1.8437748 -2.8128943 -4.3468778

H 3.6934728 -3.7770153 -2.5505928

C 1.7550298 -0.9021683 1.3672062

C 1.0247748 -0.6047033 2.4881932

O 2.4132068 -2.0957773 1.5492072

C 1.2357778 -1.6786193 3.4095332

H 0.4166538 0.2771137 2.6189162

C 2.0795678 -2.5525613 2.7859672

H 0.8072128 -1.7849753 4.3951872

H 2.5196238 -3.4954933 3.0719442

C -3.3009452 -0.1465723 -0.9320598

C -3.6608592 0.4371827 -2.1166468

O -4.3561302 -0.8857053 -0.4455038

C -5.0133392 0.0450617 -2.3740018

H -3.0246582 1.0647557 -2.7213468

C -5.3804292 -0.7502823 -1.3290648

H -5.6274072 0.3238987 -3.2178338

H -6.2945912 -1.2622433 -1.0703338

C -1.2068542 -1.9147193 -0.4273478

C -0.5479292 -2.8840693 0.2809892

O -1.3644972 -2.3265383 -1.7283668

C -0.2914652 -3.9560163 -0.6348238

H -0.2773462 -2.8373273 1.3239022

C -0.8047362 -3.5655673 -1.8333098

H 0.2122448 -4.8872983 -0.4215358

H -0.8542332 -4.0205653 -2.8093068

C -2.1762882 -0.4010373 1.6579142

C -2.6347392 -1.4532013 2.4051482

O -2.0295522 0.6979847 2.4687102

C -2.7678022 -0.9794833 3.7481032

H -2.8425422 -2.4435223 2.0312552

C -2.3896372 0.3312807 3.7259142

H -3.1032962 -1.5384263 4.6095722

H -2.3245462 1.1040797 4.4756682

H -1.2792762 4.1615477 2.1139682

C 0.6460848 2.8809967 -2.1473818

H 1.1310988 2.3715827 -2.9772528

H 1.2416298 3.6338327 -1.6366668

C -0.7314692 2.8940637 -2.0189888

C -1.9055642 3.4340687 -2.3022908

H -2.8229992 3.1054897 -1.8264998

H -1.9867892 4.2506017 -3.0194578

#### TS-19

| 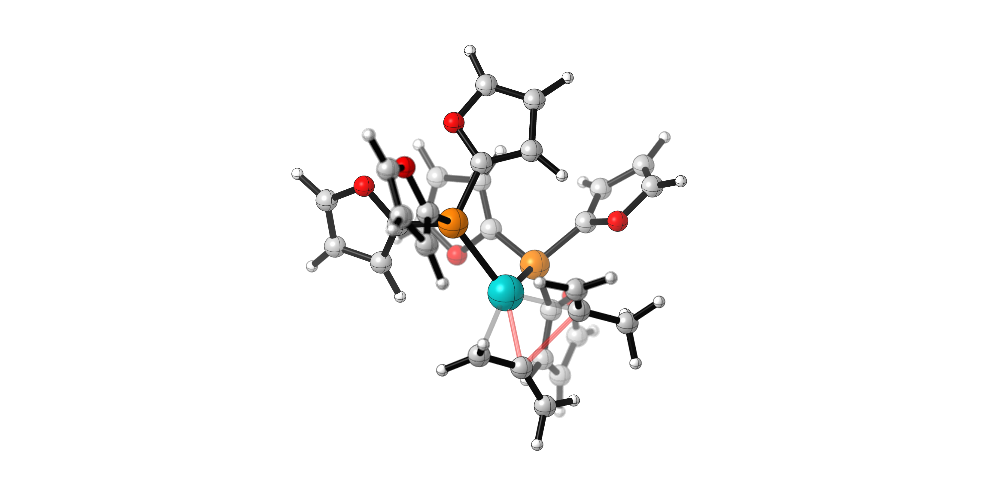 | E/hartree | -2421.351652 |
| --- | --- | --- |
|  | E+zvp/hartree | -2420.856398 |
|  | G/hartree | -2420.932935 |
|  | E(_SP)_/hartree | -2422.175331 |
|  | Imaginary frequency/cm^-1^ | -426.40 |

P 1.9272078 0.0862478 -0.1694230

P -1.5948622 -0.3657382 -0.0931770

Pd -0.0433642 1.3402978 -0.6071600

C -2.1722442 3.2393178 1.1623740

H -2.6757712 4.0380618 0.6084420

H -2.7551562 2.3265908 1.0221480

C -0.7601842 3.0888788 0.6910130

C 0.3060548 3.5948368 1.3137490

H 1.3152118 3.5186008 0.9206800

H 0.2044138 4.1107888 2.2735020

C 3.5125248 0.9431558 -0.0075530

C 3.8721648 2.2319888 -0.3006600

O 4.6049678 0.2669578 0.4854060

C 5.2599778 2.3585228 0.0254700

H 3.2175898 2.9878708 -0.7042820

C 5.6471088 1.1394798 0.4978710

H 5.8797048 3.2375638 -0.0747280

H 6.5819478 0.7493678 0.8698380

C 2.2449248 -1.1254082 -1.4682430

C 1.6400188 -1.2437312 -2.6918020

O 3.2685578 -2.0391362 -1.3732520

C 2.3206918 -2.2925512 -3.3872700

H 0.8024568 -0.6519482 -3.0295690

C 3.2927468 -2.7358522 -2.5392010

H 2.1122868 -2.6651972 -4.3795550

H 4.0473238 -3.5040672 -2.6111070

C 1.8589238 -0.8844242 1.3560290

C 1.1340478 -0.6338412 2.4911240

O 2.5564678 -2.0583432 1.5059820

C 1.3898538 -1.7183772 3.3887990

H 0.4851458 0.2144108 2.6430150

C 2.2531468 -2.5513872 2.7369520

H 0.9757578 -1.8598672 4.3762070

H 2.7249258 -3.4864242 2.9969490

C -3.1854102 -0.3411702 -0.9520460

C -3.5925152 0.3891338 -2.0357540

O -4.1834532 -1.2181272 -0.5871570

C -4.9153382 -0.0545322 -2.3564640

H -3.0054532 1.1486278 -2.5300440

C -5.2188572 -1.0238772 -1.4465740

H -5.5521352 0.3034978 -3.1521980

H -6.0913242 -1.6362322 -1.2771890

C -1.0267752 -2.0521762 -0.4793510

C -0.3317612 -3.0046022 0.2170520

O -1.1439202 -2.4381142 -1.7920960

C -0.0075842 -4.0392912 -0.7200620

H -0.0811192 -2.9688562 1.2656460

C -0.5214012 -3.6452022 -1.9172380

H 0.5383348 -4.9498152 -0.5213750

H -0.5340732 -4.0784182 -2.9042940

C -2.0683322 -0.6400522 1.6388150

C -2.5504792 -1.7139632 2.3379790

O -1.9050032 0.4207448 2.4987780

C -2.6844132 -1.2966832 3.6996920

H -2.7733912 -2.6831312 1.9194220

C -2.2814922 0.0060838 3.7368630

H -3.0370182 -1.8855962 4.5339640

H -2.2099402 0.7441638 4.5203830

H -2.2001532 3.4950718 2.2297890

C 0.5859618 3.0467988 -1.8784360

H 0.8853798 2.7585238 -2.8827020

H 1.2978138 3.6466928 -1.3203930

C -0.7673942 3.0800098 -1.5384150

C -1.9608792 3.5135448 -1.9692480

H -2.8932442 3.1651338 -1.5426290

H -2.0191952 4.2951678 -2.7232870

#### TS-20

| 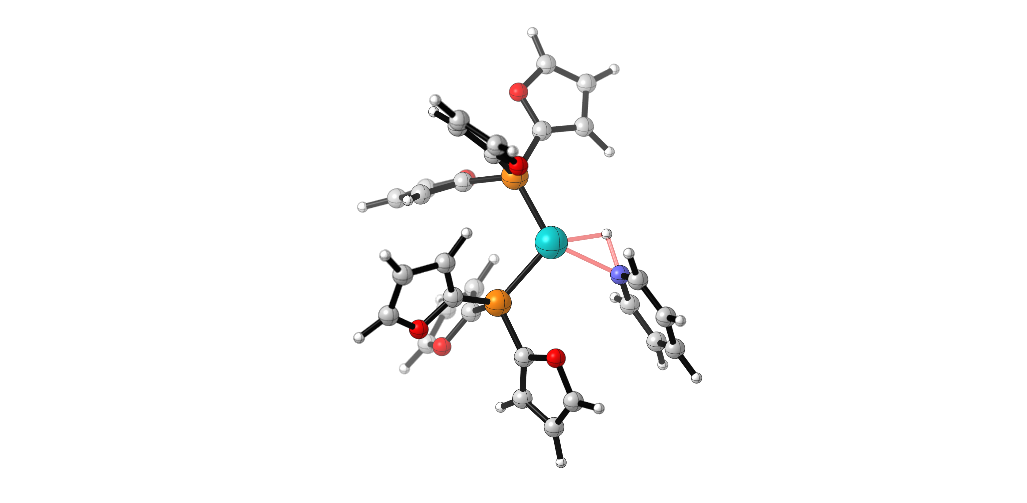 | E/hartree | -2436.100063 |
| --- | --- | --- |
|  | E+zvp/hartree | -2435.633464 |
|  | G/hartree | -2435.711428 |
|  | E(_SP)_/hartree | -2436.960614 |
|  | Imaginary frequency/cm^-1^ | -1078.26 |

P -1.3738417 0.8149863 0.0259367

P 2.1843743 -0.5743447 -0.0196073

Pd -0.0133767 -1.1472447 -0.1309173

C -3.1414487 0.4338693 0.0479097

C -4.2378247 0.8680213 -0.6470173

O -3.5149957 -0.5764027 0.9103837

C -5.3506147 0.0953923 -0.1834653

H -4.2444787 1.6604343 -1.3794283

C -4.8589107 -0.7558127 0.7601797

H -6.3796617 0.1785563 -0.5013153

H -5.3079637 -1.4999017 1.3984757

C -1.2257427 1.8591983 -1.4250943

C -0.3984267 1.7426713 -2.5112023

O -1.9998807 2.9898653 -1.5562133

C -0.6757987 2.8597413 -3.3597573

H 0.3352643 0.9657743 -2.6647913

C -1.6507427 3.5781453 -2.7327033

H -0.2090117 3.0966523 -4.3041493

H -2.1773267 4.4879243 -2.9765213

C -1.0740877 1.8761023 1.4431017

C -0.2029727 1.7198923 2.4920607

O -1.7534057 3.0621733 1.5814317

C -0.3513807 2.8759413 3.3200667

H 0.4520133 0.8771103 2.6493437

C -1.3008787 3.6510773 2.7189727

H 0.1743503 3.0968733 4.2371017

H -1.7456147 4.6033153 2.9639377

C 3.4416633 -1.6084887 -0.7747203

C 3.3516203 -2.8017447 -1.4419163

O 4.7550443 -1.2105617 -0.7167503

C 4.6830803 -3.1615407 -1.8172623

H 2.4399773 -3.3462237 -1.6380493

C 5.4878053 -2.1624767 -1.3522233

H 4.9975973 -4.0411907 -2.3588473

H 6.5522713 -1.9892087 -1.3897853

C 2.5407063 1.0662473 -0.6941533

C 2.4206413 2.3357313 -0.1950963

O 2.8485423 1.1200513 -2.0303573

C 2.6894963 3.2255793 -1.2844033

H 2.1622763 2.5996083 0.8189417

C 2.9427033 2.4398813 -2.3677583

H 2.6969603 4.3050843 -1.2563813

H 3.2063243 2.6426853 -3.3936783

C 2.6814143 -0.3362757 1.6977787

C 3.6312553 0.4252553 2.3234627

O 1.9748913 -1.0539307 2.6396887

C 3.5057083 0.1699533 3.7249607

H 4.3312863 1.0853843 1.8340467

C 2.4927263 -0.7332317 3.8585867

H 4.0960233 0.5971093 4.5222437

H 2.0415863 -1.2263917 4.7053427

C -2.2261857 -2.8462697 -1.4432573

C -1.8807777 -3.4594537 0.8028267

C -3.5125757 -3.3551137 -1.4136943

H -1.7903627 -2.3848537 -2.3212113

C -3.1551247 -3.9955417 0.8823517

H -1.1803357 -3.4617247 1.6297807

C -3.9899117 -3.9340647 -0.2348563

H -4.1302467 -3.2924437 -2.3017083

H -3.4846317 -4.4487597 1.8100017

H -4.9927507 -4.3450477 -0.1945753

H -0.1430637 -2.7775537 -0.4478133

N -1.4432467 -2.8804867 -0.3382313

#### Int-33

| 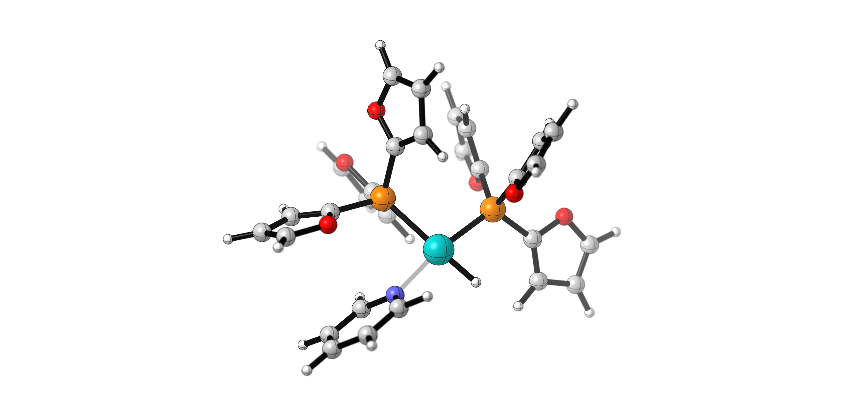 | E/hartree | -2436.153036 |
| --- | --- | --- |
|  | E+zvp/hartree | -2435.682899 |
|  | G/hartree | -2435.755926 |
|  | E(_SP)_/hartree | -2437.010052 |

P -1.1435377 -0.7627765 -0.0829605

P 2.0060773 0.7657045 0.0792965

Pd -0.1595057 1.4084665 0.2576685

C -2.9424317 -0.7952695 -0.2241285

C -3.9409797 -1.4489155 0.4470015

O -3.4906977 0.0752315 -1.1365145

C -5.1760527 -0.9566465 -0.0828975

H -3.8053927 -2.1992865 1.2108715

C -4.8447667 -0.0406265 -1.0367775

H -6.1736297 -1.2553735 0.2035615

H -5.4185717 0.5794165 -1.7071735

C -0.8544997 -1.8428345 1.3147345

C -0.3370847 -1.5537745 2.5510045

O -1.2621427 -3.1552495 1.2814565

C -0.4219067 -2.7558575 3.3205285

H 0.0707733 -0.6023835 2.8590025

C -0.9863377 -3.6894765 2.5011305

H -0.1039647 -2.9031375 4.3419545

H -1.2432057 -4.7300895 2.6258395

C -0.5060147 -1.5773245 -1.5507835

C 0.0671183 -1.0395995 -2.6745055

O -0.5519977 -2.9439575 -1.6625355

C 0.4044943 -2.1396315 -3.5232825

H 0.2352773 0.0097405 -2.8616615

C 0.0104593 -3.2654915 -2.8579435

H 0.8754843 -2.0953205 -4.4939155

H 0.0516473 -4.3187015 -3.0890155

C 3.1339763 1.6819385 1.1249985

C 2.9637603 2.7380365 1.9820615

O 4.4507733 1.2898595 1.1262745

C 4.2486793 3.0145305 2.5423365

H 2.0329703 3.2472215 2.1799065

C 5.1069593 2.1096025 1.9883935

H 4.4978373 3.7829835 3.2588205

H 6.1650533 1.9272625 2.0962305

C 2.4042193 -0.9486355 0.4687525

C 2.3994533 -2.1180545 -0.2464705

O 2.6386953 -1.2177425 1.7942735

C 2.6553053 -3.1660365 0.6928035

H 2.2369203 -2.2176605 -1.3083075

C 2.7903593 -2.5655155 1.9091445

H 2.7348863 -4.2221885 0.4828175

H 2.9946593 -2.9312325 2.9026695

C 2.5802523 0.8906155 -1.6184665

C 3.5869673 0.2854775 -2.3223975

O 1.9039953 1.7650925 -2.4371955

C 3.5270613 0.8097805 -3.6507585

H 4.2812533 -0.4429905 -1.9318355

C 2.4939693 1.7008335 -3.6628035

H 4.1724533 0.5615845 -4.4803905

H 2.0742873 2.3447815 -4.4198075

C -2.9379027 1.9332455 1.3734435

C -2.4525937 3.2416535 -0.4796745

C -4.2380707 2.4192055 1.4284215

H -2.5809967 1.1952035 2.0818975

C -3.7370617 3.7735095 -0.4891975

H -1.7080867 3.5369645 -1.2092035

C -4.6480347 3.3543965 0.4787815

H -4.9109697 2.0626325 2.1992735

H -4.0088407 4.5029945 -1.2435275

H -5.6579517 3.7508475 0.4953225

H 0.5087453 2.8102505 0.4511025

N -2.0615927 2.3327185 0.4323565

#### Int-34

| 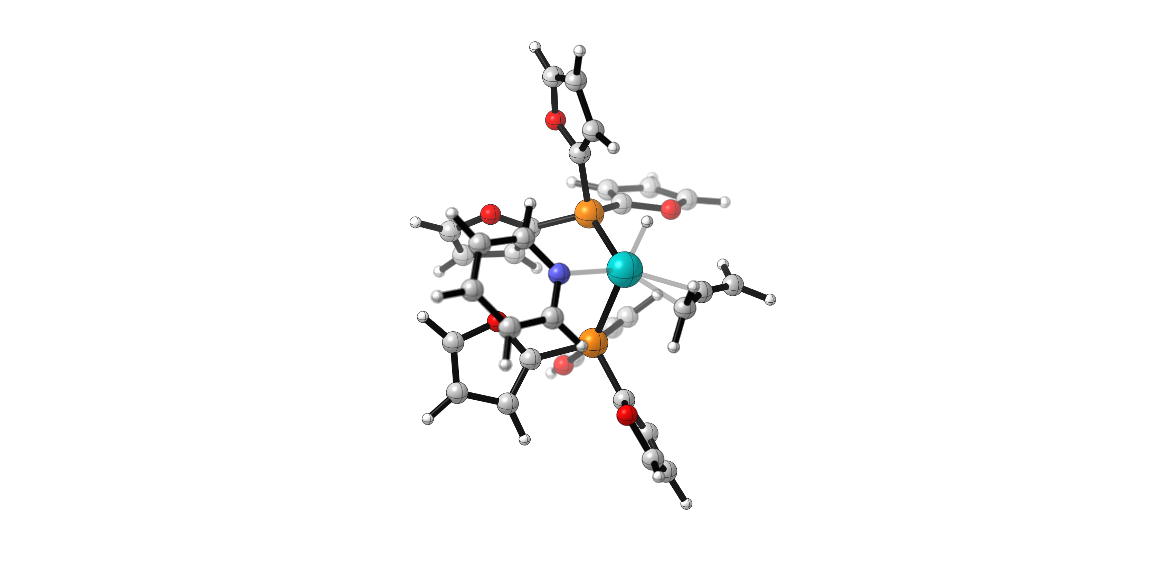 | E/hartree | -2552.833522 |
| --- | --- | --- |
|  | E+zvp/hartree | -2552.305183 |
|  | G/hartree | -2552.382045 |
|  | E(_SP)_/hartree | -2553.734717 |

P -1.9547536 -0.1603670 -0.3000519

P 1.5663604 -0.6112370 0.0533171

Pd -0.0288506 0.0687290 -1.6535119

H -1.0776556 0.4053310 -2.7341919

C 1.3122284 -0.3142140 -3.3570049

H 1.0141724 0.3409800 -4.1711999

H 2.3352394 -0.2224730 -3.0067659

C 0.5783834 -1.4425160 -3.0884439

C 0.1590884 -2.6797050 -3.2232739

H -0.6339156 -3.0850190 -2.6091539

H 0.5977984 -3.3172270 -3.9886499

C 1.0363894 -2.0101830 1.0521491

C 0.2082874 -3.0639970 0.7680671

O 1.4883964 -2.1131740 2.3457781

C 0.1382044 -3.8585100 1.9548181

H -0.2878766 -3.2442370 -0.1716959

C 0.9281424 -3.2330080 2.8769001

H -0.4279276 -4.7666340 2.0986051

H 1.1863634 -3.4456820 3.9027821

C 3.1847554 -1.1599420 -0.5253979

C 3.8887804 -2.3227340 -0.3843179

O 3.8962594 -0.2797740 -1.3120499

C 5.1082014 -2.1542340 -1.1181129

H 3.5760824 -3.1884970 0.1799421

C 5.0630184 -0.9046420 -1.6561019

H 5.9090254 -2.8708900 -1.2243239

H 5.7370594 -0.3317730 -2.2733949

C 2.0475534 0.6687720 1.2224781

C 3.2516044 1.0124480 1.7780971

O 1.0869184 1.5675230 1.6109481

C 3.0173514 2.1874970 2.5583131

H 4.1850114 0.4865560 1.6442101

C 1.6910684 2.4761640 2.4206371

H 3.7397924 2.7385800 3.1419871

H 1.0506164 3.2492310 2.8127341

C -1.8020736 0.3074940 1.4396531

C -1.4550226 -0.3636380 2.5822161

O -1.9610276 1.6413140 1.7147251

C -1.4114476 0.6147130 3.6264781

H -1.2647656 -1.4221520 2.6693231

C -1.7247886 1.8072810 3.0470241

H -1.1840816 0.4442320 4.6683411

H -1.8415646 2.8120750 3.4216201

C -2.6415986 -1.8178890 -0.1277309

C -3.3947426 -2.4333680 0.8359421

O -2.4366446 -2.6866120 -1.1738269

C -3.6599086 -3.7575640 0.3659611

H -3.7201256 -1.9870850 1.7628731

C -3.0613426 -3.8561180 -0.8549479

H -4.2292116 -4.5264340 0.8672271

H -2.9956936 -4.6399210 -1.5933939

C -3.3305476 0.8607790 -0.8480579

C -3.5295676 1.6372290 -1.9609759

O -4.4520996 0.8987730 -0.0554949

C -4.8443736 2.1863480 -1.8459009

H -2.8212666 1.7917230 -2.7612189

C -5.3533746 1.7048280 -0.6751559

H -5.3411566 2.8452140 -2.5423699

H -6.2962756 1.8338220 -0.1664749

C 1.7650904 2.7131280 -1.2702559

C -0.4280446 3.2181780 -0.7647969

C 2.2017874 3.9613900 -0.8389769

H 2.4685554 1.9770400 -1.6438449

C -0.0791686 4.4919300 -0.3229839

H -1.4580866 2.8846470 -0.7389149

C 1.2612494 4.8705400 -0.3552629

H 3.2564664 4.2085100 -0.8825399

H -0.8485386 5.1646200 0.0398721

H 1.5682044 5.8535280 -0.0125319

N 0.4740164 2.3403050 -1.2297219

#### TS-21

| 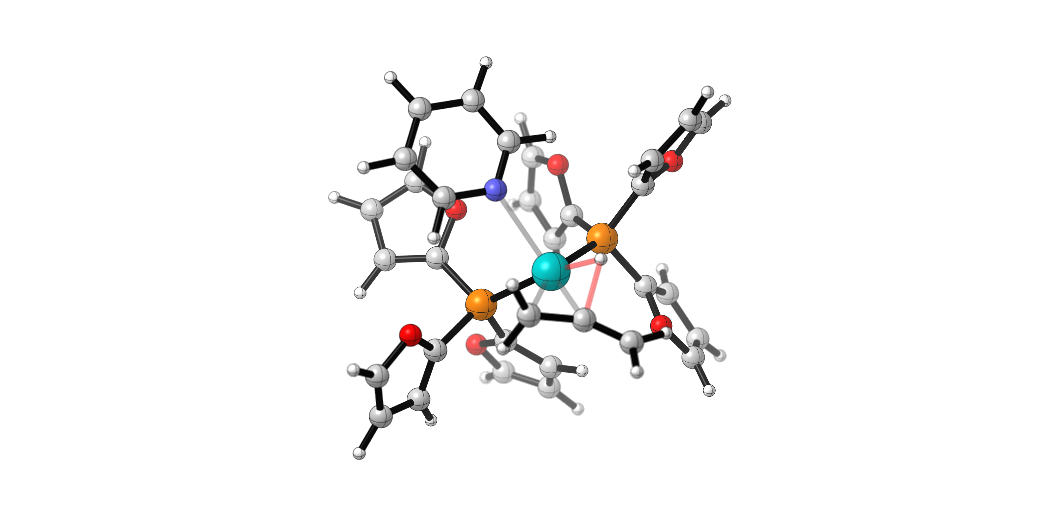 | E/hartree | -2552.825208 |
| --- | --- | --- |
|  | E+zvp/hartree | -2552.299054 |
|  | G/hartree | -2552.377145 |
|  | E(_SP)_/hartree | -2553.728848 |
|  | Imaginary frequency/cm^-1^ | -544.94 |

P -1.9440274 -0.0882823 -0.1101363

P 1.5466126 -0.7277913 0.0262407

Pd -0.1178784 -0.0769783 -1.5604983

H -1.1950374 0.1069577 -2.6964243

C 1.0893456 -0.4752663 -3.3443813

H 1.3522476 0.4980687 -3.7519563

H 1.9100286 -1.1390623 -3.0942573

C -0.1702894 -1.0010343 -3.5549803

C -1.0403164 -1.7776343 -4.1730963

H -2.0357074 -1.9517903 -3.7845053

H -0.7578754 -2.2584423 -5.1065313

C 1.0182856 -2.1339813 1.0159037

C 0.1127926 -3.1284263 0.7531167

O 1.5557436 -2.3166953 2.2677187

C 0.0833146 -3.9684113 1.9101587

H -0.4620714 -3.2367833 -0.1533713

C 0.9720536 -3.4270903 2.7946697

H -0.5216604 -4.8500673 2.0606617

H 1.2904466 -3.6973643 3.7896657

C 3.1346456 -1.2973153 -0.6140693

C 3.8797756 -2.4270743 -0.4176783

O 3.7798696 -0.4658913 -1.5020103

C 5.0555146 -2.2866543 -1.2248423

H 3.6231556 -3.2509853 0.2312817

C 4.9454276 -1.0846313 -1.8552293

H 5.8702206 -2.9893533 -1.3181183

H 5.5694456 -0.5470003 -2.5518143

C 2.0934986 0.5207697 1.2002057

C 3.3077126 0.7757487 1.7809167

O 1.1886516 1.4776397 1.5837177

C 3.1394486 1.9548467 2.5715417

H 4.2052846 0.1888187 1.6550007

C 1.8384386 2.3343007 2.4139447

H 3.8872076 2.4507437 3.1725667

H 1.2468786 3.1486227 2.7988787

C -1.7125894 0.3107677 1.6332007

C -1.3386424 -0.4184323 2.7312287

O -1.8057064 1.6373487 1.9640727

C -1.2071344 0.5159387 3.8073027

H -1.1839514 -1.4858563 2.7678307

C -1.5005564 1.7413967 3.2879787

H -0.9377104 0.2970197 4.8298717

H -1.5596044 2.7342467 3.7055027

C -2.7224654 -1.7078173 0.0033797

C -3.4559914 -2.3521373 0.9627277

O -2.5849774 -2.5239083 -1.0964383

C -3.7832174 -3.6393183 0.4321367

H -3.7249464 -1.9499363 1.9274057

C -3.2355184 -3.6881953 -0.8158333

H -4.3562434 -4.4178013 0.9140047

H -3.2216564 -4.4334883 -1.5957153

C -3.2395904 1.0472347 -0.6196243

C -3.3850464 1.8341317 -1.7340043

O -4.3438684 1.1805427 0.1849847

C -4.6478864 2.4916697 -1.6062363

H -2.6719944 1.9300827 -2.5395183

C -5.1814434 2.0579197 -0.4274923

H -5.0952144 3.1896637 -2.2980903

H -6.1036574 2.2678287 0.0920077

C 1.9362496 2.5675337 -1.3400633

C -0.1735634 3.2633197 -0.7411393

C 2.5165596 3.7278677 -0.8346843

H 2.5509306 1.7874347 -1.7789113

C 0.3166286 4.4594087 -0.2211463

H -1.2337064 3.0382557 -0.7062073

C 1.6894266 4.6952417 -0.2646913

H 3.5911026 3.8634647 -0.8866453

H -0.3677684 5.1824057 0.2095097

H 2.1075406 5.6129557 0.1367867

N 0.6150266 2.3307027 -1.2957833

#### Int-35

| 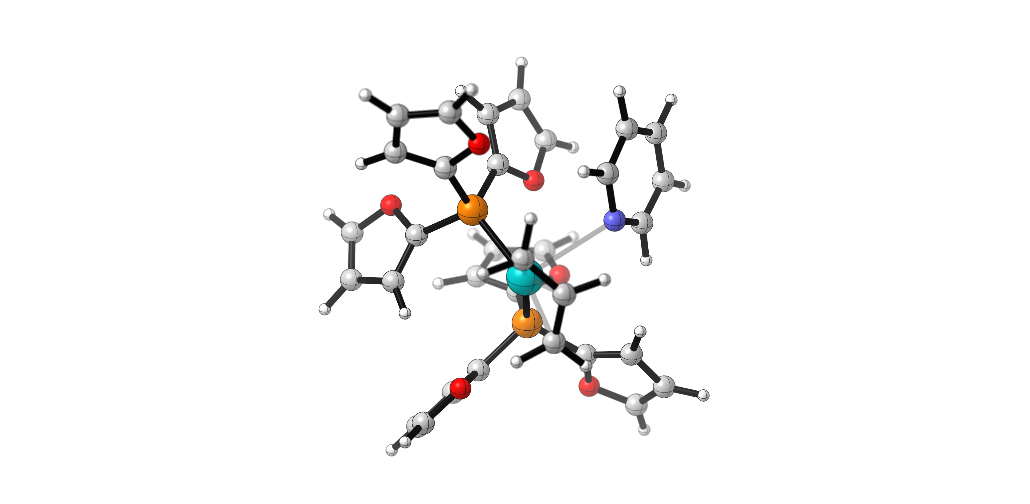 | E/hartree | -2552.890823 |
| --- | --- | --- |
|  | E+zvp/hartree | -2552.358009 |
|  | G/hartree | -2552.436364 |
|  | E(_SP)_/hartree | -2553.792736 |

P -1.9462569 -0.2264465 -0.1946154

P 1.5479921 -0.7887625 -0.1297084

Pd -0.1088199 -0.1175895 -1.6107044

H 0.0490141 1.5455375 -3.8777914

C 1.1222281 -0.2725605 -3.4409524

H 2.0950841 0.2030355 -3.4613044

H 1.1244311 -1.3544155 -3.5515134

C -0.0369929 0.4702285 -3.7425014

C -1.3049429 -0.0840515 -3.4964874

H -2.1923069 0.5326115 -3.5691484

H -1.4662059 -1.1560165 -3.5724644

C 1.0540841 -2.3061135 0.7048336

C 0.1528271 -3.2779365 0.3554866

O 1.6250031 -2.6197385 1.9150006

C 0.1627811 -4.2419225 1.4118766

H -0.4466339 -3.2937085 -0.5413704

C 1.0682881 -3.7902685 2.3289496

H -0.4279779 -5.1434125 1.4778816

H 1.4157571 -4.1651395 3.2792916

C 3.1556861 -1.2430055 -0.8086544

C 3.9456791 -2.3522505 -0.6802004

O 3.7778771 -0.3214465 -1.6183424

C 5.1256871 -2.1040465 -1.4538924

H 3.7157591 -3.2307165 -0.0960974

C 4.9729751 -0.8638115 -1.9955464

H 5.9704791 -2.7638145 -1.5851574

H 5.5836271 -0.2509975 -2.6400004

C 2.0476651 0.3418145 1.1741716

C 3.2440691 0.5300895 1.8150036

O 1.1119911 1.2091585 1.6758036

C 3.0305191 1.5701305 2.7715996

H 4.1566951 -0.0147325 1.6248956

C 1.7237861 1.9408135 2.6428606

H 3.7534621 1.9847655 3.4583956

H 1.1062091 2.6758915 3.1314256

C -1.7372649 -0.0308415 1.5878846

C -1.3563779 -0.8742625 2.5983036

O -1.8793939 1.2428785 2.0733466

C -1.2698759 -0.0717815 3.7800486

H -1.1704389 -1.9337195 2.5109356

C -1.5957849 1.1975825 3.4049496

H -1.0081869 -0.4029315 4.7740906

H -1.6927399 2.1311945 3.9365546

C -2.7556099 -1.8323295 -0.2743384

C -3.5140319 -2.5649355 0.5977646

O -2.5900159 -2.5390145 -1.4458384

C -3.8333379 -3.7909835 -0.0657974

H -3.8036539 -2.2589545 1.5914266

C -3.2533729 -3.7207475 -1.2978284

H -4.4227269 -4.6089415 0.3216316

H -3.2238399 -4.3855425 -2.1470214

C -3.2028999 0.9984705 -0.5799794

C -3.2148669 2.0572535 -1.4512584

O -4.3922729 0.9519515 0.1040746

C -4.4827259 2.6994715 -1.2951924

H -2.4023339 2.3465525 -2.1003994

C -5.1519509 1.9860145 -0.3434904

H -4.8455859 3.5697115 -1.8215644

H -6.1313559 2.0761955 0.1003166

C 1.7301221 2.7459035 -1.1671714

C -0.2552219 3.4291845 -0.2457854

C 2.4576951 3.6681545 -0.4188654

H 2.2407401 2.0671105 -1.8446104

C 0.3811541 4.3957175 0.5312246

H -1.3300519 3.2944365 -0.1822374

C 1.7668401 4.5160855 0.4459406

H 3.5368491 3.7161655 -0.5141464

H -0.2012829 5.0327665 1.1886126

H 2.2984531 5.2521855 1.0407276

N 0.3958261 2.6188235 -1.0933314

#### Int-36

| 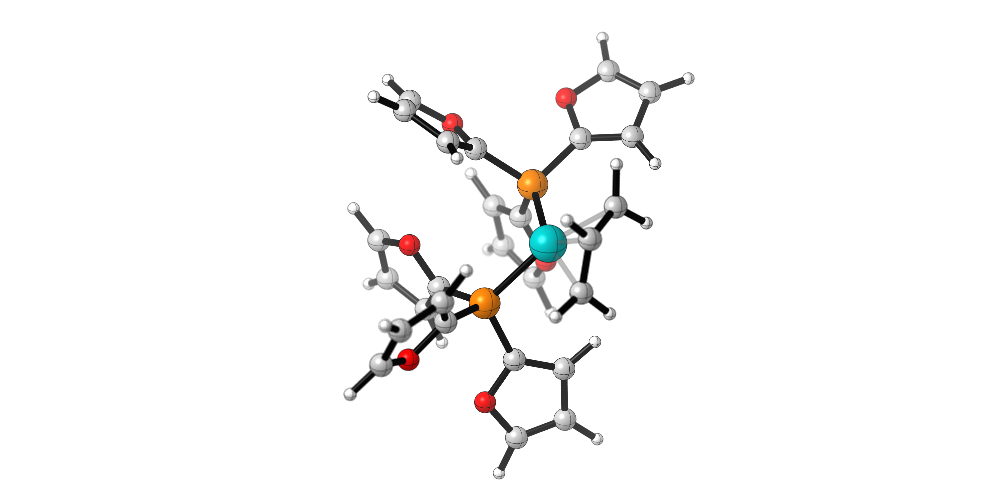 | E/hartree | -2304.553259 |
| --- | --- | --- |
|  | E+zvp/hartree | -2304.111447 |
|  | G/hartree | -2304.183164 |
|  | E(_SP)_/hartree | -2305.352510 |

P 1.8136424 0.2386111 -0.0274313

P -1.6929876 0.1688891 -0.0278273

Pd 0.0372504 1.7353591 -0.1762423

C 3.4427804 0.8774751 0.3884447

C 3.8690694 2.0188881 1.0172757

O 4.5185374 0.0592521 0.1448177

C 5.2863134 1.9012751 1.1615037

H 3.2495424 2.8370171 1.3489397

C 5.6227854 0.6982191 0.6141467

H 5.9598474 2.6175161 1.6079127
[truncated: 137,088 more chars]
